# From Prediction to Practice: A Systematic Review of the Decision Support Gap in Artificial Intelligence and Machine Learning for Environmentally Sustainable Livestock Production

**DOI:** 10.3390/ani16213502

**Published:** 2026-11-05

**Authors:** Amir Mohammad Shadjou, Saghar Mohsenian, Parham Vatankhah, Farzam Hosseinpour Vardin, Uttama Deb, Fatemeh Taghvaei, Dongyi Wang, Marty Matlock

**Affiliations:** 1 Department of Biological and Agricultural Engineering, University of Arkansas, Fayetteville, AR 72701, USA; ashadjou@uark.edu (A.M.S.); farzamh@uark.edu (F.H.V.); taghvaei@uark.edu (F.T.); dongyiw@uark.edu (D.W.); 2 Environmental Dynamics Ph.D. Program, Graduate School and International Education, University of Arkansas, Fayetteville, AR 72701, USA; sagharm@uark.edu (S.M.); udeb@uark.edu (U.D.); 3 School of Biomedical Engineering, College of Science and Engineering, Flinders University, Adelaide, SA 5042, Australia; parham.vatankhah@flinders.edu.au

**Keywords:** machine learning, livestock sustainability, decision support systems, hybrid modeling, greenhouse gas emissions, systematic review, technology adoption

## Abstract

Artificial intelligence (AI) and machine learning (ML) are promoted as tools for improving the environmental sustainability of livestock production, but it remains unknown whether the models reported in this literature are used to support real-world decisions. We systematically reviewed studies that applied AI or ML to environmental sustainability outcomes in livestock production. Four databases were searched in January 2026, and 165 of 1994 screened records were included. The protocol was registered, but the main outcome was defined after data extraction, so the analysis is exploratory. A study counted as delivering decision support only if it named a specific decision and used its model to compare options or recommend an action. The authors of 96 studies (58.2%) stated that their work could support decisions, but only 26 studies (15.8%) met our definition. This gap appeared in 10 of 11 application areas. The gap held under both a broader and a stricter definition, and when a second coder repeated the coding. Only 30 studies (18.2%) tested their model outside the setting where it was developed, and 17 (10.3%) included an optimization step, which turns a prediction into a choice. Linking ML with mechanistic farm models is a promising approach that still needs testing. We also describe the main components a practical decision tool would need.

## Simple Summary

Artificial intelligence (AI) and machine learning (ML) are being increasingly incorporated in livestock research. They can predict things such as emissions, pasture conditions, or animal traits, but a prediction alone does not inform a farmer, manager, or policymaker how to make decisions. We reviewed 165 studies that applied AI or ML to livestock sustainability. Authors of 96 studies explicitly said that their work was relevant to decision making, and authors of another 59 made a partial claim. We then looked separately at whether the studies actually offered decision support. To meet our definition, a study had to identify a specific decision (on a farm, in a manure treatment process such as a digester, or in a policy program) and use the model to compare options or recommend an action. Only 26 studies met this definition. Most studies produced useful predictions or classifications but did not show how the results could guide a specific choice. Fewer than one in five reported external validation. This pattern appeared in 10 of the 11 application domains. Future research should connect model results to realistic options and test whether using them improves outcomes in practice.

## 1. Introduction

### 1.1. Livestock Sustainability as a Farm Level Decision Problem

Demand for animal source food continues to rise with population growth, urbanization, and income [1], and this production carries a substantial environmental burden. On a life cycle basis, livestock agrifood systems emitted an estimated 6.2 Gt carbon dioxide equivalent (CO_2_-eq) in 2015, about 12% of anthropogenic greenhouse gas (GHG) emissions, with enteric fermentation as the single largest source, followed by feed production [2]. An earlier assessment by the Food and Agriculture Organization of the United Nations (FAO) estimated 7.1 Gt CO_2_-eq, or 14.5%, for 2005 [3]. The two figures are not directly comparable, because the newer estimate uses updated emission factors and global warming potentials [2]. In Europe, livestock accounts for approximately two thirds of agricultural land use and around three quarters of diffuse nitrogen and phosphorus losses to water [4,5]. Reducing these pressures is the objective of sustainable intensification, i.e., producing more from existing agricultural land while reducing environmental impact per unit of output [6,7].

On a farm, sustainability depends on a series of practical choices: how many animals to keep or replace, what to feed them, how to store or treat manure, how to manage grazing, and where to invest. These choices affect one another and can influence environmental outcomes. Decision-support tools are intended to help farmers weigh those choices.

The artificial intelligence (AI) and machine learning (ML) studies in this review use many kinds of information, including farm records, animal measurements, sensor readings, images, and satellite data. The models use those data to estimate a value, sort observations into groups, or identify patterns. For example, a model may estimate pasture biomass, methane emissions, or energy use; identify an animal condition or land-cover class; or group similar farms before estimating their emissions, as in a study of Indonesian smallholder dairy farms [8]. In each case, the model turns information into a result that may be easier to use. Whether that result is then used to make a management decision is a separate question examined later in this review.

### 1.2. Prior Reviews and the Unexamined Step

Earlier reviews of AI and ML in livestock farming describe which methods are used, what they predict, and how accurate the models are. They show that much of the work focuses on identifying animals, monitoring health and behavior, and predicting production. Accuracy is often reported as strong [9,10,11,12], while environmental sustainability receives less attention [13]. However, these reviews do not ask whether accurate predictions help someone make a decision. To check this, we searched OpenAlex for reviews of AI or ML in livestock farming published since 2015, added the reviews found in our own search and two further reviews identified after it, and read each abstract against a written rule (Supplementary Section S11, Table S26). Of the 117 reviews, 106 describe methods, tasks, data or accuracy without asking what became of the models, and 10 look further at deployment, readiness or adoption. Among them, an umbrella review of 112 reviews of computer vision in precision livestock farming found that reported model performance often exceeds the evidence for use on farms [14], and a review published after our search found evidence of on-farm deployment and measurable management impact in fewer than 5% of computer vision studies [15]. None of these reviews asks whether the models were used to compare management options or to recommend an action, which is the question of this review. Research on agricultural decision support describes a continuum from better information through advice to action [16,17,18]; Section 2.7 explains which part of that continuum this review measures. A separate line of work uses farm models to represent how biological, economic, and environmental parts of a farm interact. These models can estimate environmental impacts and compare management options [19,20]. They are easier to interpret because they are based on known farm processes, but they need local data and can be slow to run. In crop science, researchers have combined these farm models with AI and ML so that each approach can contribute what it does best [21,22].

The key unanswered question is whether accurate livestock AI predictions become tools that people can use to make decisions. Farm decision-support systems have existed for many years, but many have not been widely adopted because they do not always fit farmers’ needs or work easily with farm data [16,17,23]. Dairy systems show the same problem. Tools may have clear potential but still be difficult to use, integrate, or show a clear benefit [24]. A research field can therefore produce many accurate models without producing many tools that help users choose between real options.

### 1.3. Objectives and Research Questions

This review has two aims. The first is to describe how AI and ML have been used to study sustainability in livestock systems: the methods used, the sustainability outcomes examined, the species and scales covered, and the quality of the studies. The second is to ask whether studies that say they support decisions actually give farmers, processors, or policy makers information they can use to choose between options. For the second aim, we measure the gap between studies that claim to support decisions and studies that do so under a written definition. We then check whether the gap is present in each application area and whether it changes when lower-quality studies are excluded. Finally, we describe what the studies that do not provide decision support have in common, and what approaches the studies that do provide it use.

## 2. Materials and Methods

### 2.1. Protocol and Registration

This review was conducted and reported in accordance with the Preferred Reporting Items for Systematic Reviews and Meta-Analyses (PRISMA) 2020 statement [25]. Differences in species, algorithms, and outcomes precluded pooling, so we used narrative synthesis following the Synthesis Without Meta-analysis (SWiM) guideline [26]; Supplementary Table S25 lists the nine SWiM items, where each is addressed in this review, and how the three items written for effect sizes were adapted to a synthesis of study counts. It was registered on the Open Science Framework in September 2025 (registration ysw5q, https://doi.org/10.17605/OSF.IO/YSW5Q) before the database searches on 9 January 2026. Before searching and screening, the registered information sources were amended because Scopus and AGRICOLA were unavailable; searches were conducted in Web of Science, CAB Abstracts, IEEE Xplore, and PubMed. We did not run an additional search in Google Scholar. Eligibility was restricted to peer-reviewed journal articles, and conference papers were excluded during screening before data extraction. During full-text screening, before data extraction and analysis, the scope was narrowed from farm-system modeling, life-cycle assessment, and artificial intelligence or optimization to artificial intelligence and machine-learning applications only, so that each stream could be reported in a separate article. Before final analysis, following this scope amendment, the registered inferential and meta-analytic analysis plan was replaced with the descriptive narrative synthesis reported here. The central endpoint, whether a study delivered functional decision support, was not among the registered hypotheses; it emerged after data extraction during cross-tabulation and is therefore reported as exploratory. The completed PRISMA 2020 checklist accompanies this submission, and full details of the protocol amendments are provided in Supplementary Section S2.

### 2.2. Eligibility Criteria

Eligible studies met seven criteria: application of an AI or ML method as a core rather than peripheral methodology; a livestock species as the study subject; measurement, prediction, or assessment of at least one environmental, resource efficiency, or integrated sustainability outcome; original research; publication as a peer reviewed journal article; English language publication; and full text availability. Eligible livestock systems included cattle, pigs, poultry, sheep, goats, and mixed-species systems. Seven codes, E1 to E7, were applied in a fixed hierarchy, with only the first applicable reason recorded for each study (Table 1); E6 records reports whose full text could not be retrieved and is counted separately from assessed reports, following PRISMA 2020, so six codes (E1 to E5 and E7) are exclusion reasons.

Two boundary rules substantially affected inclusion. Studies were excluded if removing machine learning would leave their contribution substantially unchanged (E1), and livestock had to be the subject of the study rather than its setting (E2). For E2 the boundary is the farm gate. A study is in scope when the quantity modelled with the AI or ML method, or the decision that model supports, concerns live animals or herds, the farm or grazing system that keeps them, manure, slurry or litter at any stage of treatment or use, or inventories and maps of livestock populations and their emissions. A study is out of scope when both the model and the decision concern only a stage after the animal has become a food product, such as slaughter, meat or dairy processing, packing, retail or consumption. A study that covers farms together with processing sites stays in scope when the farm stage is part of what is modelled or decided. This rule was written down and every included study was checked against it (Supplementary Section S2, amendment 9). One study, which chose the product mix, fuel and refrigerant of a poultry processing plant [27], failed the rule and was excluded under E2. The process level decisions of Section 2.7 therefore concern digesters, composters and other manure treatment steps, not food processing plants.

For E1, a written rule was applied to every included study. A study counts as applying AI or ML when its core method passes three tests. First, it learns from data, meaning that an algorithm fits the model’s parameters or structure from observations, not from hand-set values, expert rules or physical equations alone. Second, it produces outputs for cases beyond those it was fitted on, by predicting, classifying, clustering, detecting or choosing actions. Third, it is central to the study, not a peripheral step. Optimization algorithms on their own, such as particle swarm or genetic algorithms, do not count; they count only when used to fit or tune a model that passes the first two tests. Statistical models such as regressions and mixed models count only when they are built and judged as predictive models with held-out validation. Fuzzy systems count when their rules or membership functions are learned from data. Mechanistic models never count on their own and make a study Hybrid only when coupled with a model that passes the tests. Applied to all 165 studies, the rule excluded none and moved two studies from an Other category to Traditional ML, namely a projection model fitted by particle swarm optimization and tested on held-out years [28], and a matrix completion model that predicts counterfactual farm outcomes [29]. These two and a changepoint and growth-curve simulation study [30] pass only through the clause on statistical models and are the weakest inclusions; Supplementary Section S2 (amendment 10) records the rule and its application, and there is no Other category.

The same eligibility threshold was applied to every species. The registered protocol allowed an exception to E3 for goat studies; it was not applied, and the four goat studies that measured no sustainability outcome were excluded under E3 (Supplementary Section S2, amendment 6). The analysis workbook records the four exclusions.

### 2.3. Information Sources and Search

On 9 January 2026, we searched four databases: Web of Science Core Collection, CAB Abstracts via EBSCO, IEEE Xplore, and PubMed. Three sources named at registration were not searched. Scopus was not accessible to the team through an institutional subscription. AGRICOLA, which is freely accessible, was not searched because its agricultural coverage was judged to be largely duplicated by CAB Abstracts; that judgement was not tested. Google Scholar was dropped after trial searches returned several hundred thousand records that could not be exported or screened reproducibly. IEEE Xplore and PubMed were added (Supplementary Section S2, amendment 2). The consequence for coverage was tested in two ways. First, the search was re-run in OpenAlex, a multidisciplinary index broader than any of the four databases, and a random sample of the additional records was screened (Supplementary Section S12). Second, the reference lists of the 165 included studies and the papers citing them were screened in the same way (Supplementary Section S15). The search linked three concept blocks with Boolean AND: an AI and ML block, a livestock species block, and a sustainability outcome block. It used free text terms adapted to each database’s syntax, without controlled vocabulary. The base search string, adapted to the syntax of each database, was as follows:

Block 1 (AI and ML): (“artificial intelligence” OR “machine learning” OR “deep learning” OR “neural network*” OR “random forest” OR “support vector machine” OR “gradient boosting” OR “XGBoost” OR “reinforcement learning”).

AND Block 2 (livestock): (livestock OR dairy OR cattle OR cow* OR bovine OR goat* OR caprine OR sheep OR ovine OR pig* OR swine OR porcine OR poultry OR chicken* OR “farm animal*”).

AND Block 3 (sustainability): (sustainab* OR “life cycle assessment” OR “life cycle analysis” OR LCA OR “carbon footprint” OR “greenhouse gas” OR GHG OR methane OR “environmental impact” OR “water footprint” OR “water use” OR “energy use” OR “economic viability” OR “profitability” OR “production efficiency”).

Search results were restricted to English-language journal articles published from January 2010 through the 2026 search date. The 2010 lower date limit defines the review’s search window; it was not intended to mark the historical beginning of artificial intelligence and machine-learning research in livestock sustainability. The string executed in each database, with its platform, search field, limits and record count, is reported in Supplementary Section S1. The strategy was tested in four ways. Every included study was checked against the exported record sets. The search terms were matched against the text of the included studies. An expanded string with additional species, method and outcome terms was re-run in two databases, and a random sample of the additional records was screened. Finally, the strategy was self-assessed against the Peer Review of Electronic Search Strategies (PRESS) checklist and reviewed independently by a university librarian (Supplementary Section S12). The corpus is therefore a reproducible but not exhaustive sample of the eligible literature.

### 2.4. Study Selection Process

Records were deduplicated and screened in two stages using the Rayyan screening platform (Rayyan Systems Inc., Cambridge, MA, USA). At the first stage, two reviewers independently screened the titles and abstracts of all 1994 unique records against the eligibility criteria. The platform requires both decisions to agree before a record moves on, so conflicts were settled by discussion inside Rayyan, and the number of initial conflicts was not recorded. At the second stage, the 308 retrieved full texts were divided among five reviewers, and each full text was assessed by only one of them against a written screening guideline, with the decision and the exclusion code from the E1 to E7 hierarchy recorded in a shared template. Each full text was screened by one reviewer only. Every included study was then checked a second time against the eligibility criteria under written rules (Supplementary Section S2, amendments 6 and 9); this check covered the inclusions but not the full text exclusions. Screening decisions and their rationales were logged for audit.

### 2.5. Data Extraction and Canonical Mapping

We read each study in full and extracted 47 fields. These cover study identification, geographic and system context, species and production system, ML methods and validation, sustainability outcomes and units, application domain, decision support framing, practical readiness, self-reported findings and limitations, and a structured quality assessment. Five reviewers divided the studies between them and extracted the data using a shared written guide. Each study was extracted by one reviewer; no study was extracted twice. One author then checked every extracted value against written cleaning rules and recorded each change in a cleaning log (Supplementary Section S13). Some fields were coded a second time. The functional flags F1 to F6 and the decision support claim were coded by a second coder (Section 2.7; Supplementary Section S14). The input structure variable was coded a second time for every study (Supplementary Section S13). Three quality dimensions were rated a second time on a random sample of 40 studies (Section 2.6), and the stated transportability target was coded a second time on the same 40 studies (Section 3.6; Supplementary Table S23). All other fields, including application domain, practical readiness and the external validation field, were coded once and were not independently checked (Section 4.5). Values not found in a study were recorded as “not reported” rather than inferred. The full field list and definitions are provided in Supplementary Section S5.

Free text entries were mapped to canonical categories: nine algorithm families, five method categories, eleven application domains, and seven outcome unit families. Three kinds of rule were used. Species, geographic scope, production system, method category, validation method, transparency and reproducibility were recoded to the written value sets of the codebook. Algorithm family, application domain, outcome unit family and sample size bin were assigned through lookup tables with one row per study. Country was taken as the first country listed, with sub-national detail removed; studies naming several countries are counted under the first, and regional or multi-country labels are kept as labels. The structure of the model inputs was coded by hand for every study from the extracted input, data source and algorithm fields, under written definitions. Every change was recorded in an auditable cleaning log of 279 entries. Of these, 22 are judgement overrides, in which the cleaning reviewer departed from an extracted value on the basis of the study rather than of a written rule. The rest are format normalizations, rule based collapses, and coding or audit entries. The rules, the keyword lists, every override, and a recount of countries under alternative conventions are given in Supplementary Section S13. The mapping code is released with the analysis materials, and all reported counts use the canonical variables. Definitions of the technical terms used in this review are provided in Supplementary Table S17.

### 2.6. Methodological Quality and Reporting Assessment

Risk-of-bias instruments for prediction models are designed for clinical prediction in human participants. Many of their signaling questions (participant selection, outcome definition, predictor timing) have no counterpart in livestock sustainability studies, so risk of bias in the formal sense was not assessed. Instead, methodological quality and reporting were assessed on three dimensions tailored to ML research, each with three ordered levels: data quality (sample size and data collection reporting), model transparency (reporting of architecture, hyperparameters and training procedures), and reproducibility (code and data sharing versus description alone). Each dimension has written signaling questions (Supplementary Table S7). For example, data quality is rated High when the sample size, the data collection and the data handling are all reported. Two further items, an external validity label and an overall confidence rating, are reported descriptively only and do not enter the quality summary. The external validity label has two levels and so cannot be mapped onto the three-level scale; the overall confidence rating summarizes the three dimensions and would count them twice. These dimensions follow emerging standards for reporting and reproducibility in machine learning-based research [31,32,33]. Those standards emphasize transparent documentation and warn that leakage and incomplete reporting can produce optimistic and irreproducible results. Each dimension targets one of these failure modes. Small or poorly described datasets can inflate apparent accuracy. Unreported architecture, hyperparameters and training procedures prevent replication and conceal leakage. Unshared code and data prevent independent verification.

The three dimensions were combined by rule into a composite quality rating. The rating was ‘Low concern’ if all three received the most favorable level, ‘High concern’ if at least two received the least favorable level, and ‘Some concerns’ otherwise. Study quality was summarized descriptively and used in the sensitivity analysis (Section 3.7.6); no study was excluded on quality grounds. To test whether the ratings can be reproduced, a second reviewer rated a random sample of 40 studies (24%) on the three dimensions from the full texts. The second reviewer used the signaling questions and did not see the first rating. Percent agreement and Cohen’s kappa are reported in Section 3.6 and Supplementary Table S7, which also gives the full distribution of all ratings.

Whether a model can be expected to work somewhere else, its transportability, was recorded with three separate items instead of a single label. The first is the scope of the data: single farm, several farms, regional, national or international. The second is whether the model was tested on data from outside the training context, that is, on a different farm, herd, region or trial, or on an independent dataset. Testing on a later time period from the same source was counted as external testing and recorded as a separate basis (Supplementary Table S16, flag VAL_external). The third item is the target that the authors themselves state for the model. It was coded from the abstract, discussion and stated limitations into four categories: the studied setting only (including the mapped area of a mapping study); a named wider population such as a region, country, breed or production system; a general claim of applicability without a named population; or no statement. The coding rules were written down before coding (Supplementary Section S2, amendment 8). For the studies that claim applicability beyond their own setting, external validity is then reported as the share whose model was actually tested outside the training context (Supplementary Tables S23 and S24).

### 2.7. Operational Definitions of Claimed and Delivered Decision Support

This review distinguishes between claimed and delivered decision support. The agricultural decision support literature recognizes two broad families of tools: those that improve the information available to a decision maker, and those that use a model to compare or recommend actions [16,17]. We measured the second family only. The endpoint reported here is therefore action-oriented, model-based decision support, and we use “delivered decision support” as shorthand for it throughout. This is a deliberately strict construct. A study can inform a decision without meeting this definition, for example by supplying a reliable emission estimate that a producer then acts on. We chose the strict form because the question of this review is whether a trained model is used to choose between actions, which is the step that separates a decision tool from a prediction. Studies that improve the information base without comparing or recommending actions are counted under the broader sensitivity definition reported in Section 3.7.6. The distinction between claimed and delivered support matters because studies may claim decision support without using the model to compare management options, recommend an action, or guide a specific decision. Decision support claims were identified using the extracted “decision support focus” field. This field captures whether the authors explicitly intended the study, model, or analytical output to support decisions by producers, managers, planners, or other users. Each study was coded as ‘Yes’, ‘Partial’, or ‘No’. This variable records what the authors intended, without evaluating whether their studies provided decision support in practice. A second coder independently coded this field for all 165 studies (Supplementary Section S14).

Delivered decision support was assessed separately based on what the model or analytical output actually enabled users to do. Each study was assessed against a set of functional criteria using its full text and extracted information, rather than relying on the author’s terminology. Six flags represented these criteria (F1 to F6; Table 2). F1 to F3 correspond to what Ruchay et al. [18] call advisory decision support, in which model outputs are converted into a recommendation that a person approves and acts on. F4 to F6 record whether that recommendation was made accessible, used in operation, and shown to change an outcome, which their readiness scale treats as later, supervised stages. Closed-loop control, the highest level of that scale, lies outside the scope of this review.

The assessment covered decisions at three levels. Farm-level decisions included feeding strategy, grazing management, stocking rate, housing or environmental control, and technology adoption. Process-level decisions included operating conditions, feedstock ratios, pretreatment methods, or the configuration of systems such as digesters, composting processes, or manure treatment systems; decisions inside food processing plants are outside the scope of the review (Section 2.2). Policy or program-level decisions included choices such as selecting target farms or geographic areas and choosing interventions, pathways, or programs. The model’s demonstration scale (e.g., laboratory, pilot, or commercial) was recorded separately and did not determine whether decision support was delivered.

Three functional criteria were used to determine the primary decision support classification. F1 required identifying a specific decision and an adjustable variable or management lever. For example, feed allocation could be the decision and feeding rate the adjustable lever. F2 required the study to use the model, or an analysis directly based on model outputs, to prospectively compare at least two alternative settings of that lever. F3 required using the model output to identify or recommend a preferred setting, target, strategy, or control rule.

A study delivered decision support if F1 and at least one of F2 or F3 were coded ‘Yes’. Thus, predicting an outcome was insufficient. The model had to support an identifiable decision by comparing alternative actions or identifying a preferred action.

Several types of analysis did not qualify for F2. Experimental treatment comparisons did not qualify if the model served only as a measurement or prediction tool. Similarly, retrospective analyses of past decisions did not qualify as prospective decision support. Scenario analyses also did not qualify if they varied only external or exogenous conditions, such as weather or market, without changing a variable under the decision-maker’s control.

Studies were coded ‘No’ if no specific decision was identified (F1 = ‘No’) or if neither alternative actions nor a preferred action were determined (F2 = ‘No’ and F3 = ‘No’), and ‘Unclear’ when the available information was insufficient to assess the criteria. ‘Unclear’ studies were reported separately, treated as ‘No’ in the primary estimate of delivered decision support, and treated as ‘Yes’ in a sensitivity analysis estimating the upper bound.

Two alternative definitions, one broader and one stricter, were written down and dated before being applied to any study, and were used as a sensitivity analysis (Section 3.7.6; Supplementary Section S2, amendment 7).

To test whether the coding can be reproduced, a second coder independently coded all 165 studies on F1 to F6 from the full texts under the written rubric, blind to the first coding. Disagreements were not reconciled. The first coding, which was checked against every full text, is the analysis coding. Every disagreement is listed in Supplementary Table S36, and the counts under the second coding are reported alongside the headline count.

Three additional criteria characterized later stages of decision support implementation but were excluded from the primary ‘Yes’/‘No’ classification. F4 recorded whether the study provided an accessible tool, interface, software system, or other mechanism allowing intended users to interact with the model or its outputs. F5 recorded whether the model or decision support output was evaluated in an operational setting. F6 recorded whether the study measured the consequences of decisions based on the model or its outputs. These criteria were analyzed separately because recommending actions, providing tools, evaluating their use, and demonstrating decision outcomes represent distinct stages from model development to practical decision support.

### 2.8. Synthesis and Software

Counts and cross-tabulations were computed from the frozen analysis workbook, using mutually exclusive categories for distributions and non-exclusive flags for capabilities, and all figures were produced in R version 4.5.0 (R Foundation for Statistical Computing, Vienna, Austria) [34] using the ggplot2 package (version 3.5.2) [35]. Percentages use the full corpus denominator of 165 unless stated otherwise; temporal analyses use 164, excluding the single study published in 2026, a year only partly covered by the search. Confidence intervals (CI) for proportions use the Wilson method. Because the claim rate and the delivery rate are measured on the same 165 studies, the CI for their difference uses the Wald interval for paired proportions.

## 3. Results

### 3.1. Study Selection Results

**Figure 1:**
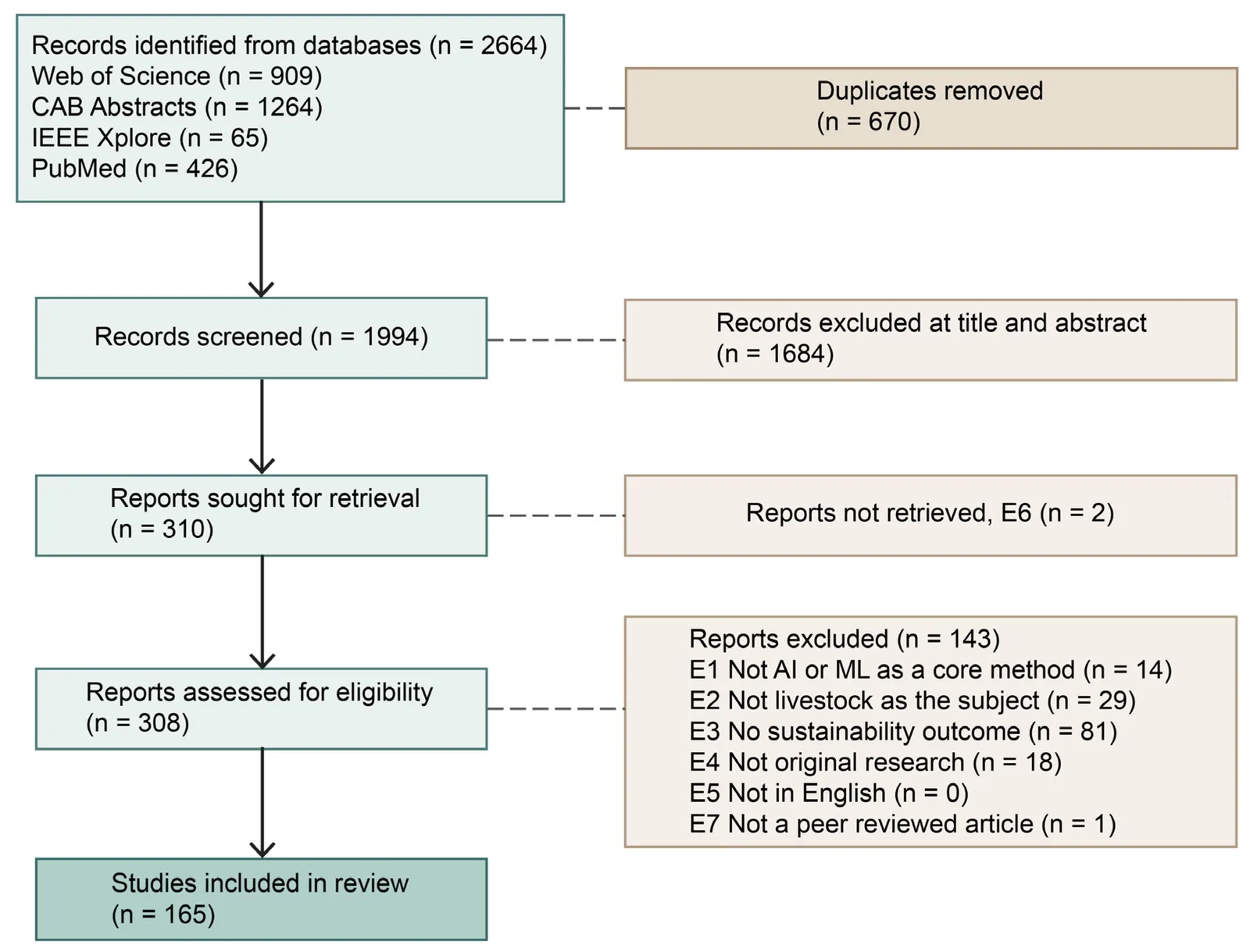
PRISMA 2020 flow diagram for identification, screening and inclusion of studies.

The database search returned 2664 records: 909 from Web of Science, 1264 from CAB Abstracts, 65 from IEEE Xplore, and 426 from PubMed. After removal of 670 duplicates, 1994 unique records were screened at title and abstract, of which 1684 were excluded. Full texts were sought for 310 records; 2 could not be retrieved and 308 were assessed. Of these, 143 were excluded with reasons recorded, most commonly no measured sustainability outcome (81), followed by not livestock (29), not original research (18), not AI or ML (14), and not a peer reviewed journal article (1). The final corpus comprised 165 studies (Figure 1).

### 3.2. Corpus Profile

**Figure 2:**
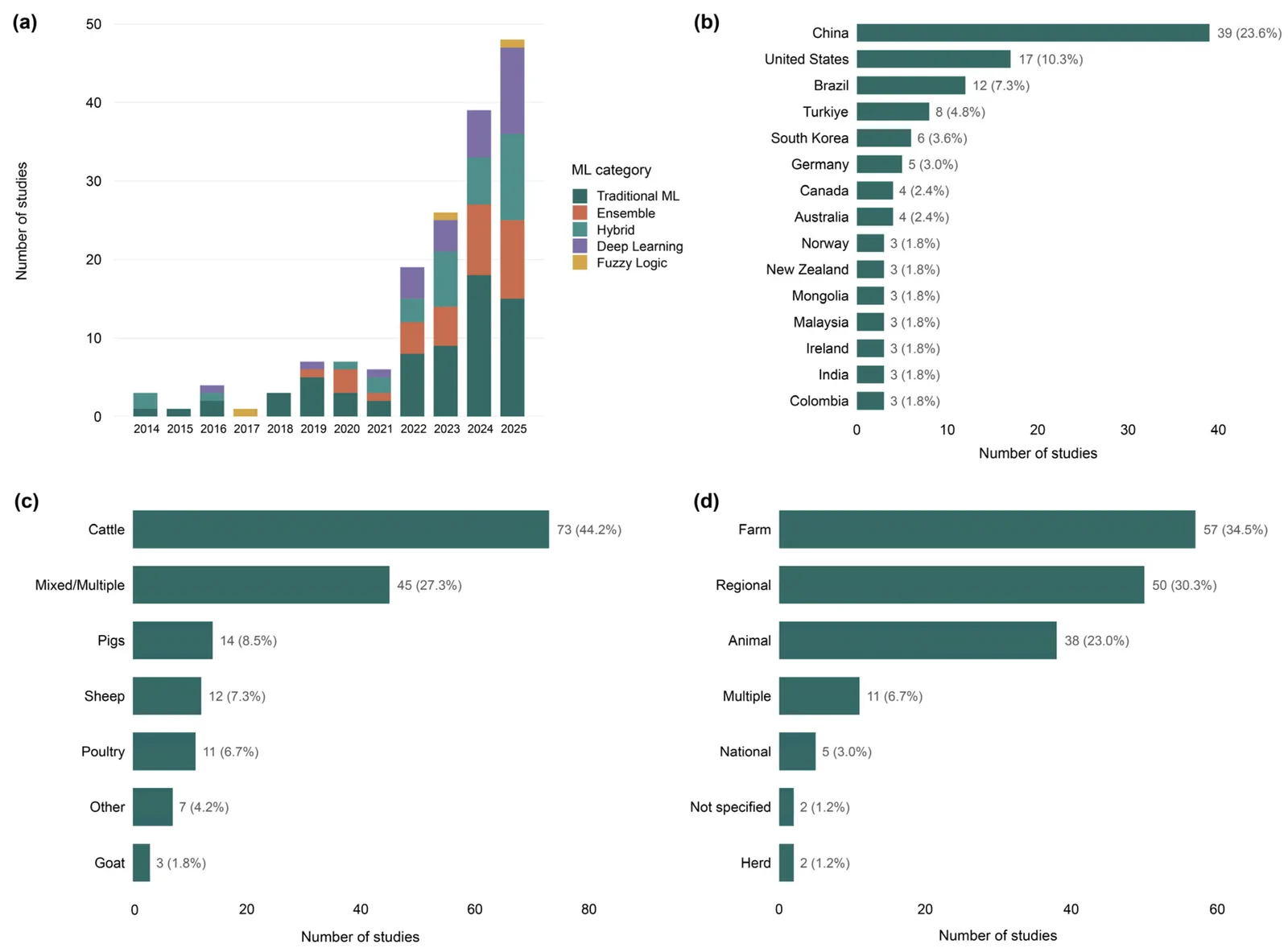
Profile of the included corpus. (**a**) Publication year by machine learning category, 2014 to 2025 (*n* = 164; the 2026 indexed study is excluded because coverage of that year is incomplete at the search date). (**b**) Number of studies by first-listed country, fifteen most frequent. (**c**) Primary species. (**d**) Measurement level at which outcomes are reported.

Within the 2010–2026 search window, publication activity was concentrated in the later years (Supplementary Table S20, Figure 2a); no trend model was fitted. Out of 164 dated studies, 12 (7.3%) appeared between 2013 and 2018, whereas 132 (80.5%) were published between 2022 and 2025, with the largest single year being 2025 (48 studies). Counted by the first country listed for each study, the corpus spans 43 countries and 7 regional or multi-country labels; 10 studies name more than one country. China leads with 39 studies (23.6%), followed by the United States (17, 10.3%), Brazil (12, 7.3%), Türkiye (8, 4.8%), and South Korea (6, 3.6%); the same five lead in the same order when each study is shared equally among the countries it names (Supplementary Table S34), and the geographic distribution is mapped in Supplementary Figure S5. Most studies operate at single farm (56, 33.9%) or regional (50, 30.3%) scale, and the most common study design is modeling (78, 47.3%).

National research output concentrates in particular domains. For example, 7 out of 8 Turkish studies address manure and waste to energy; all 4 Australian studies address grazing and pasture management; 7 of 12 Brazilian studies address grazing; and of 17 United States studies, 8 address manure and 3 address spatial detection of animal feeding operations. The 165 studies are distributed across 99 distinct journals spanning multiple disciplines, with no single disciplinary home; 70 journals contribute exactly one study each. Possible reasons for these patterns are considered in Section 4.2.

### 3.3. Methodological Landscape

**Figure 3:**
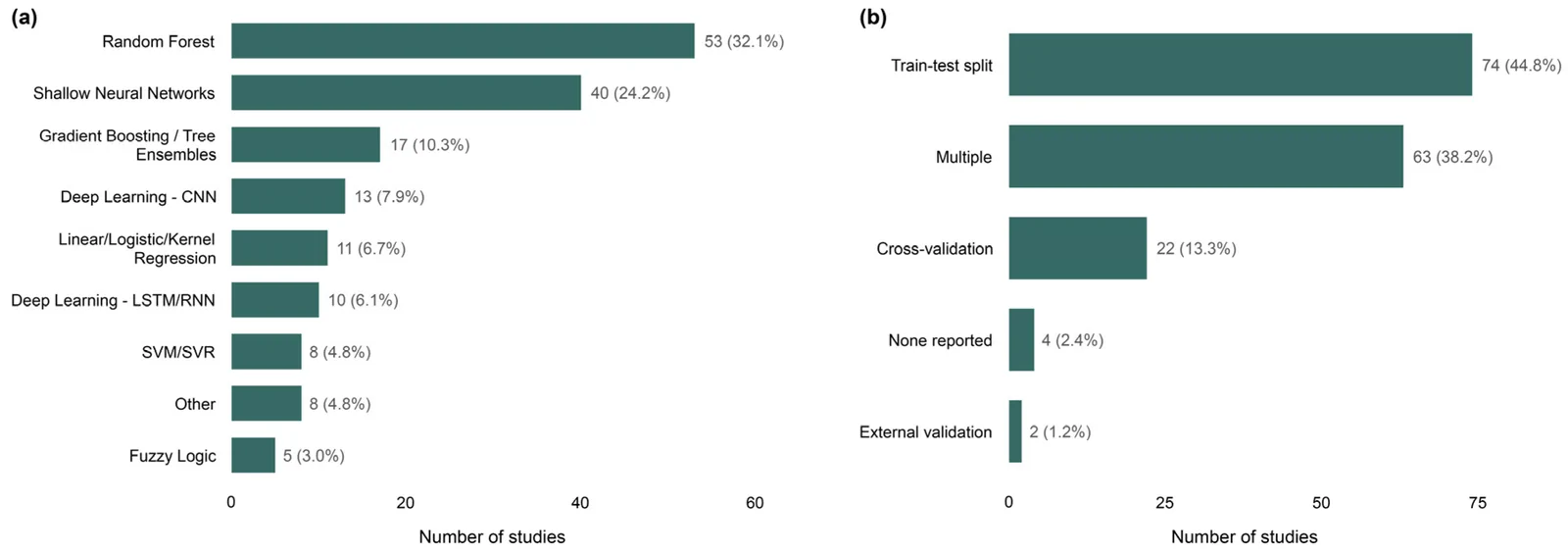
Methodological landscape of the corpus. (**a**) Algorithm families, showing that random forests and shallow neural networks together account for more than half the corpus. (**b**) Reported validation strategy in the canonical coding, in which combinations of strategies collapse to Multiple and 2 (1.2%) are coded as external validation; counted as non-exclusive flags, some form of external validation is reported by 30 (18.2%) (Supplementary Figure S3).

Traditional ML is the most frequent family (67 studies, 40.6%), with ensemble methods at 34 studies (20.6%), hybrid methods at 33 (20.0%) and deep learning at 28 (17.0%); random forests and shallow neural networks together account for more than half the corpus (Figure 3; Supplementary Table S18).

Prediction is the predominant purpose. Counted as non-exclusive flags, a prediction task appears in 127 studies (77.0%), classification in 40 (24.2%), an optimization step in 17 (10.3%), and decision support, as a label, in 7 (4.2%); optimization is the sole purpose of only 7 studies (4.2%). This distribution is revisited in Section 3.7.

**Figure 4:**
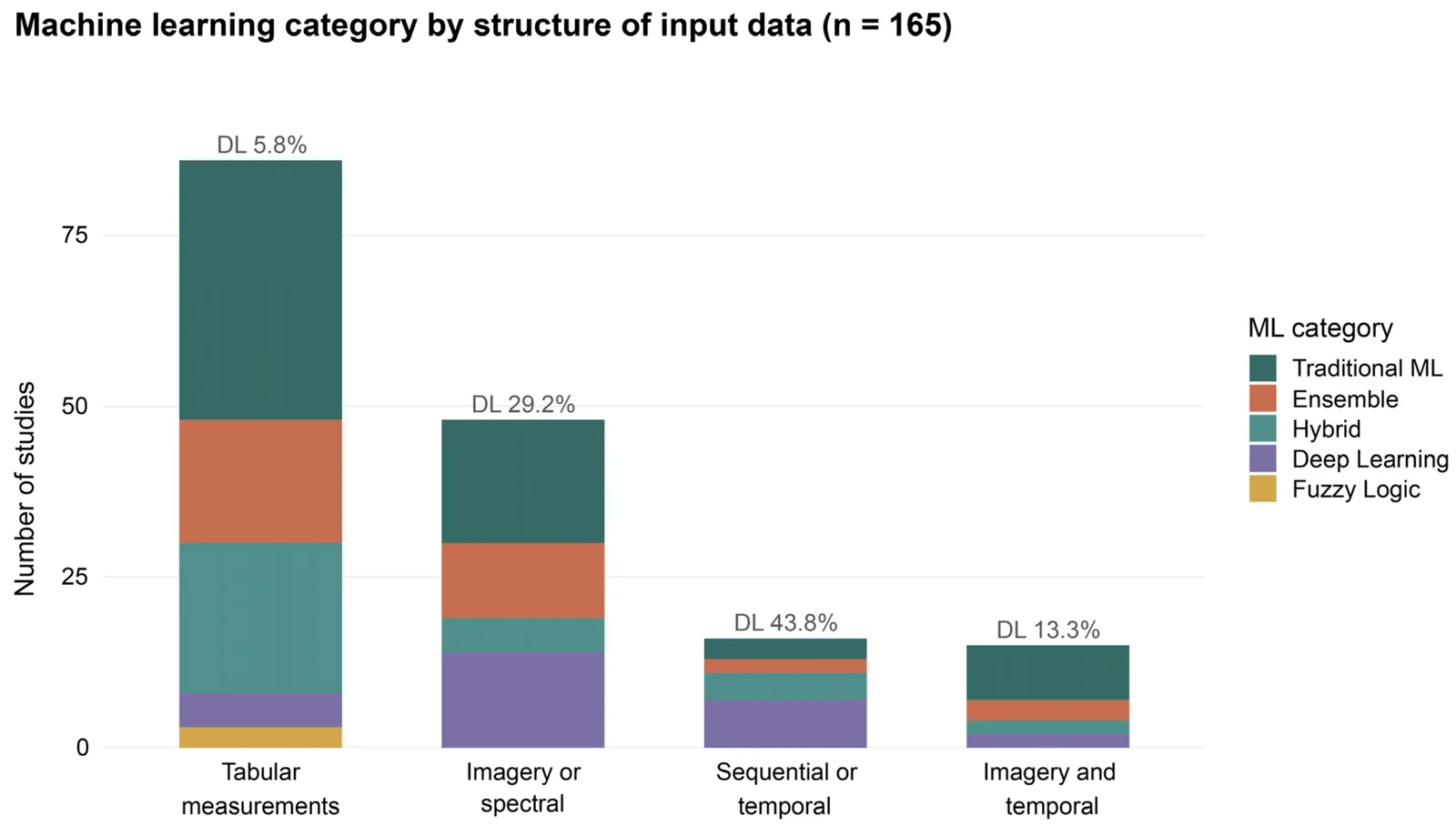
Machine learning category by structure of input data (*n* = 165). Deep learning accounts for 29.2% of studies with imagery or spectral inputs against 5.8% of studies with tabular inputs, whereas the deep learning share is similar across the three largest application domains.

Method family varies with the structure of the input data more than with the application domain. The share of studies using deep learning is similar across the three largest application domains, at 16% for grazing and pasture management, 13% for manure and waste to energy, and 17% for enteric fermentation. Classifying studies by the structure of their input data produces a different picture (Supplementary Table S21, Figure 4). Deep learning accounts for 14 of 48 studies (29.2%) with imagery or spectral inputs, against 5 of 86 (5.8%) with tabular inputs. At algorithm family level the separation is sharper. Twelve of 13 convolutional neural network (CNN) studies (92.3%) use imagery, whereas 34 of 40 shallow neural network studies (85.0%) use tabular measurements. Input structure was coded by hand for every study (Supplementary Section S13).

Across the four publication-period bins, traditional machine learning declined from 58% of studies in 2014–2018 to 38% in both 2022–2023 and 2024–2026, whereas deep learning increased from 8% to 18% and 19%, respectively. Ensemble methods accounted for 20% and 23% of studies in the two later period bins (Supplementary Table S2).

### 3.4. Sustainability Outcomes and Application Domains

**Figure 5:**
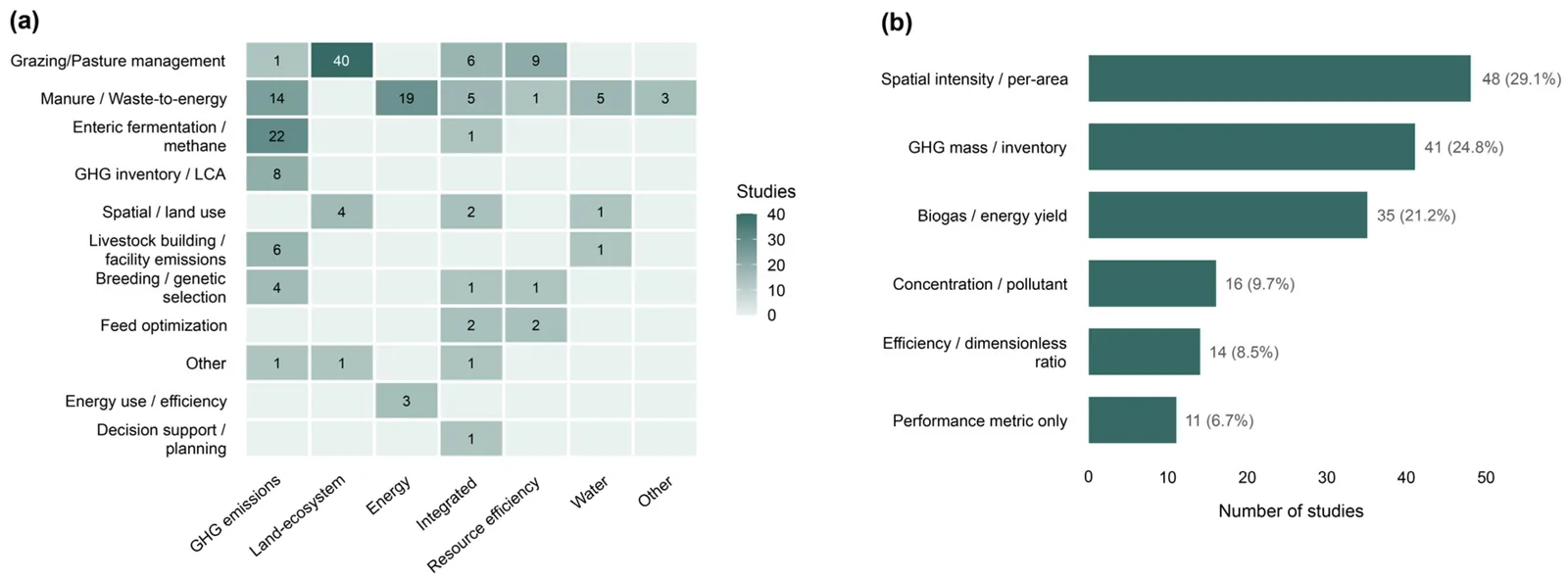
Sustainability outcomes across the corpus. (**a**) Canonical application domain by primary sustainability category. (**b**) Outcome unit families, showing the predominance of spatial or per area intensities and physical mass quantities.

Greenhouse gas emissions are the most common primary sustainability category (56 studies, 33.9%), followed by land and ecosystem outcomes (45, 27.3%), energy (22, 13.3%), integrated economic environmental assessment (19, 11.5%), resource efficiency (13, 7.9%), and water (7, 4.2%). Mapped onto canonical application domains, the corpus concentrates in grazing and pasture management (56, 33.9%) and manure and waste to energy (47, 28.5%), with enteric fermentation and methane third (23, 13.9%). The remaining eight domains are individually small, and decision support and planning is the smallest, with 1 study (0.6%). Domains and their representative studies are summarized in Table 3, with distributions in Figure 5 and the method mix by domain in Supplementary Figures S6 and S7.

#### 3.4.1. Grazing and Pasture Management

The largest cluster addresses forage rather than animals. Over-grazing degrades land and under-grazing wastes it, and distinguishing the two requires knowing standing biomass. Satellite sensors measure reflectance in bands including the red edge and near infrared, and the learning problem is to relate those bands to measured biomass.

Most studies in this cluster relate satellite or drone reflectance to field measured biomass or forage quality with random forests or support vector regression, and performance is generally good; Irisarri et al. [37], for example, predicted four forage quality attributes across United Kingdom grasslands from Sentinel-2 imagery at R^2^ between 0.77 and 0.86. A second group applies the same methods at national and continental scale to produce multi decade maps of pasture extent, grazing intensity, and rangeland condition [38,39,40]; their outputs are maps and inventories. Recent studies use finer resolution, longer records, and visible proxies for what cannot be observed directly, such as silage bags as a marker of intensification [67]. Few take a step from the map to a grazing decision (Section 3.7).

#### 3.4.2. Manure and Waste to Energy

Anaerobic digestion converts manure into methane-rich biogas, transforming a waste stream and emission source into an energy product. Yield depends on feedstock composition, carbon to nitrogen ratio, temperature, pH, retention time, mixing, and the state of an unobservable microbial community. Mechanistic models exist but are complex, making the domain well suited to learned approximation.

Most studies in this cluster predict biogas or methane yield from feedstock and operating variables at bench scale with neural networks or gradient boosting, typically reporting R^2^ above 0.9, and a smaller group forecasts regional or national biogas potential from livestock statistics [63,68]. What distinguishes this domain is that prediction is more often followed by a search over operating conditions. For example, Long et al. [42] optimized temperature and pH over a model of a commercial scale digester, Zou et al. [44] generated a Pareto front between methane yield and energy efficiency for a microbial electrolysis cell digester, and Tryhuba et al. [43] combined waste prediction with multi-objective optimization of farm configuration and rations. Chen et al. [69] went furthest, using real time camera-based weight estimation to drive precision feeding on a commercial farm and measuring a 7.8% reduction in feed consumption and a 5.1% reduction in carbon footprint per kilogram of live pig. Most of these optimization studies are at a laboratory scale.

#### 3.4.3. Enteric Fermentation and Methane

Enteric fermentation is the most difficult quantity in this corpus to measure. Three predictor classes appear.

Diet composition is the longest established predictor class. Networks trained on carbohydrate fractions or total mixed ration composition consistently outperform the linear equations conventionally used, and Ellis et al. [46] reported a concordance correlation of 0.917 for support vector regression against 0.42 to 0.70 for the best conventional equations. Milk mid infrared spectra form a second class with an existing measurement infrastructure, since spectra are already collected for payment and recording [47,61]. The rumen microbiome is the newest and least settled class, adding modest accuracy to conventional predictors and, in the largest multi country dataset, explaining about half the variation in individual emissions [48]. The outputs are estimates of an emission rather than choices about a diet, and few are evaluated on farms outside the training data; Choo et al. [8], evaluated across unseen farms, is an exception.

#### 3.4.4. Remaining Domains

The eight remaining domains are individually small. Spatial detection studies locate animal feeding operations from aerial imagery for inventory and regulatory purposes and are among the few to test transferability, with detection accuracy declining sharply between landscapes [56,70]. Housing studies predict ammonia and methane concentrations from in barn sensors; they include the one housing study that converts a prediction into a control action, a fuzzy ventilation controller driven by a deep learning forecast [55], and the only studies that specify how many days of measurement a practitioner needs for a stable estimate [52,53]. Energy, breeding, feed, and water studies each contribute a handful of papers; the energy studies are notable for reporting an economic consequence of their recommendations, which is otherwise rare [64,65], and the one study that couples a learned surrogate to a life cycle model to search a large scenario space is a grazing study whose search targets breeding goals [71].

### 3.5. Species Coverage

**Figure 6:**
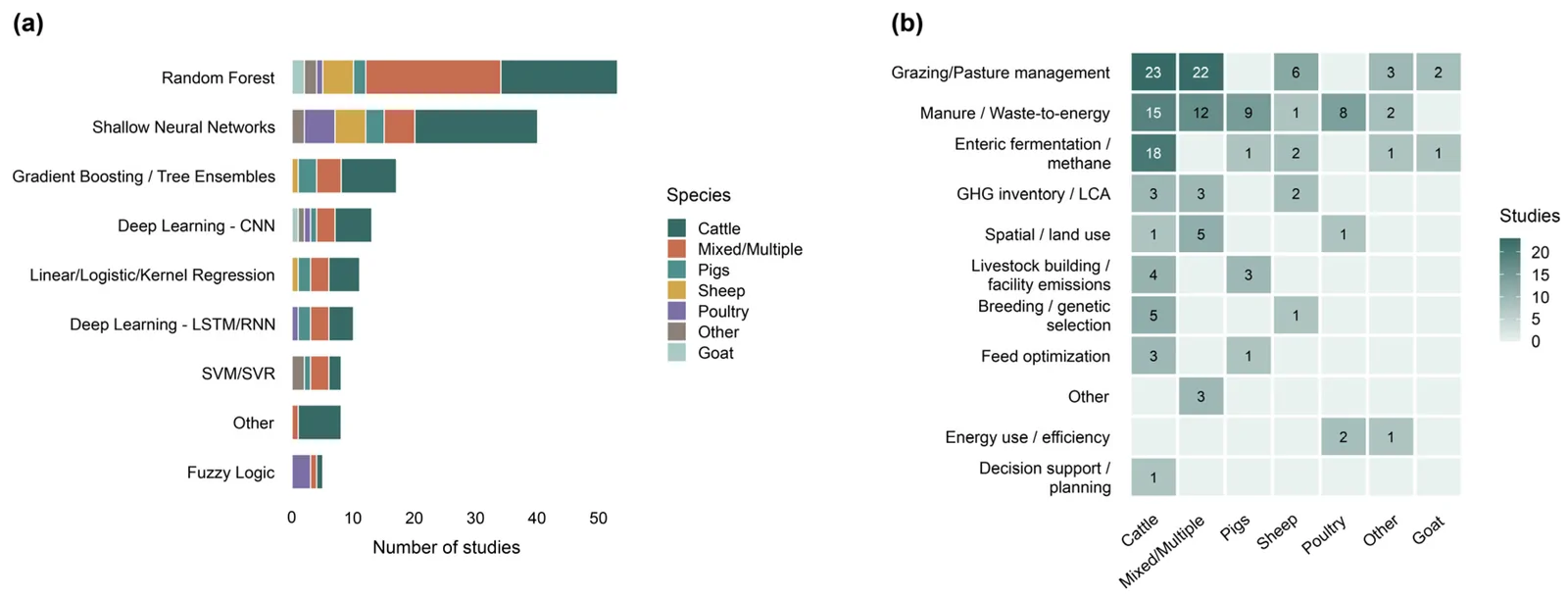
Species coverage across the corpus. (**a**) Species representation within algorithm families. (**b**) Canonical application domains by primary species.

Cattle account for 73 studies (44.2%) and mixed or multiple species systems for 45 (27.3%), with pigs (14, 8.5%), poultry (11, 6.7%), sheep (12, 7.3%), other species (7, 4.2%), and goats (3, 1.8%) accounting for the remainder. Species distribution across methods and application domains is shown in Figure 6.

Species and application domains were coupled in this corpus. Out of 14 pig studies, 9 address manure and waste-to-energy and 3 address housing emissions; of 11 poultry studies, 8 address manure. Cattle studies are distributed across grazing (23), enteric fermentation (18), and manure (15); sheep studies concentrate in grazing (6) and goat studies in grazing (2). Possible reasons are considered in Section 4.2.

### 3.6. Methodological Quality

**Figure 7:**
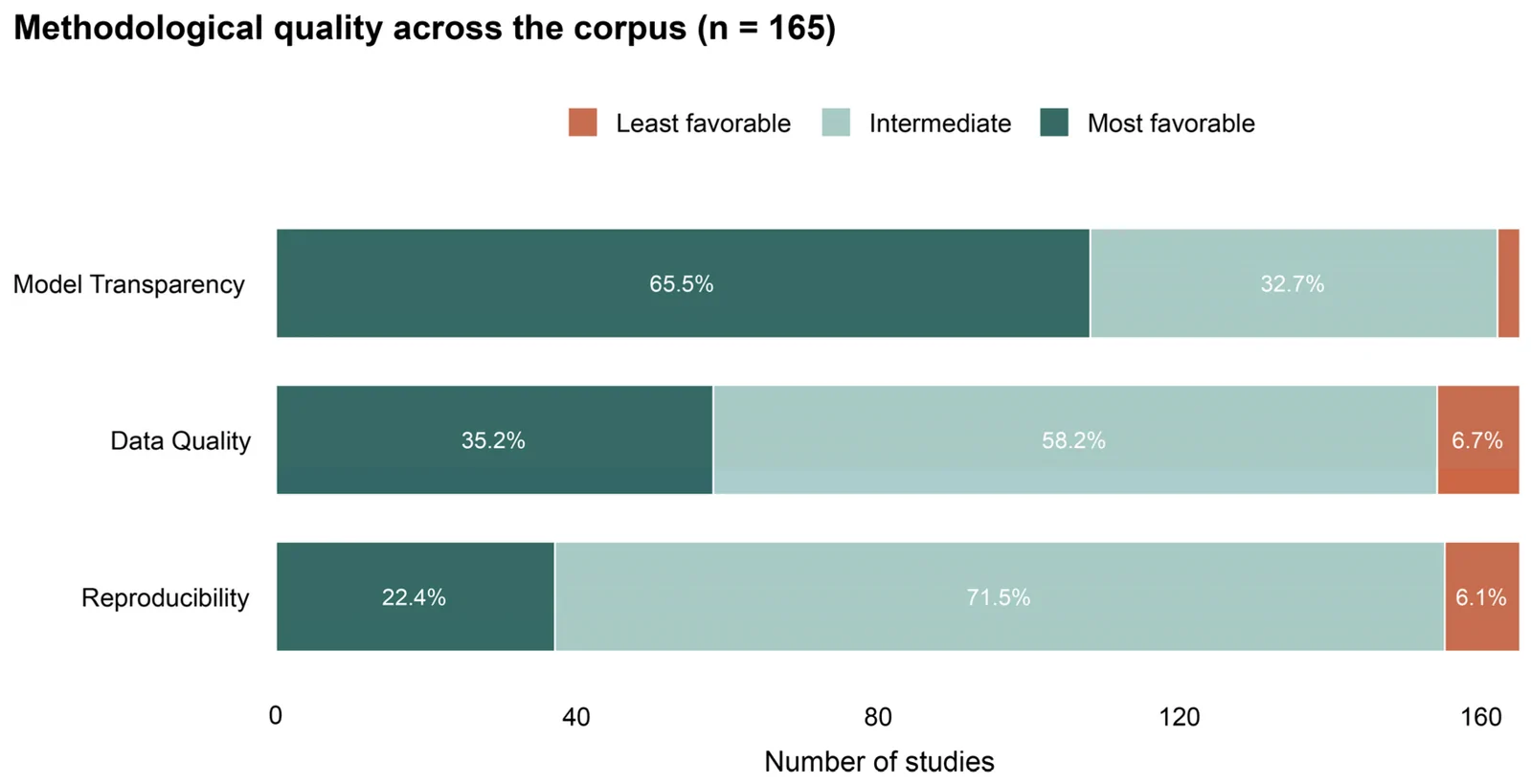
Methodological quality across the corpus for the three rated dimensions, each shown as the share of studies rated at the least favorable, intermediate and most favorable levels (*n* = 165).

On the three rated dimensions, most studies fall in the middle. The revised composite rating places 20 studies (12.1%) at Low concern, 141 (85.5%) at Some concerns, and 4 (2.4%) at High concern (Figure 7; Supplementary Table S7).

A second rating of 40 randomly chosen studies showed that the three dimensions are not equally reproducible. Agreement with the first rating was good for reproducibility (90.0%, kappa 0.70). It was poor for model transparency (67.5%, kappa 0.12). For data quality it was no better than chance (37.5%, kappa 0.04). On data quality, the second reviewer applied the written rule and rated 20 studies more favorably and 5 less favorably than the first rating. This suggests that the first rating also weighed how adequate the sample was, which the written rule does not score. Agreement on the composite rating was moderate (87.5%, kappa 0.55). The composite is therefore kept for the sensitivity analysis, where it changes little. The data quality and model transparency distributions should be read as descriptive only. The analyses use the first ratings. Both ratings and every disagreement are held by the authors and available on request.

Only 30 studies (18.2%) tested their model on data from outside the training context (Section 3.3). Of these, 9 tested on an independent farm, herd, region or trial, 15 on an independent dataset, and 6 on a later time period only. Half of the corpus, 81 studies (49.1%), states that the model applies beyond the setting it was built in. Of these, 16 name a wider population, such as a country, breed or production system, and 65 make a general claim. Only 18 of the 81 (22.2%; 95% CI 14.5 to 32.4) report any test outside the training context, and 6 (7.4%) report a test on an independent farm, herd, region or trial. The remaining 63 claim more than they tested. Another 72 studies (43.6%) limit their claims to the studied farm, system or mapped area, and 12 (7.3%) say nothing about where the model applies (Supplementary Table S23). Of the 26 national studies, 8 claim applicability beyond their data and 1 tested it. Of the 56 single farm studies, 29 claim it and 4 tested it (Supplementary Table S24).

Sharing of code or data was limited. Overall, 37 studies (22.4%) share code or data. Among dated studies, the sharing rate rose from 1 of 12 (8.3%) in 2014 to 2018 to 21 of 84 (25.0%) in 2022 to 2024. Reported sharing varied more than fourfold across application domains (Supplementary Table S8). This is a descriptive difference; the review did not assess the reasons for it.

A highest reported R^2^ could be extracted from 92 studies. In the smallest sample size bin, 57% of studies report R^2^ of at least 0.95, against 29% to 37% in the three larger bins (Supplementary Figure S8). This describes what is reported; it is not evidence of an inverse relationship, for three reasons. The highest value per study is taken across different targets and models. The sample size field counts animals, farms, plots, images, experimental runs or years, so the bins do not measure comparable amounts of independent information. And across the 92 studies, the rank correlation between the reported value and sample size is weak. Some reported values are at the theoretical maximum. Okwu et al. [72] report R^2^ of 1.0 with zero error for a fuzzy biogas model fitted on thirteen experimental runs. Şenol et al. [73] report a correlation of exactly 1 on both training and test partitions for a national biomethane forecast. In two studies a simpler method outperformed a more flexible one. Rodriguez et al. [74] found that conventional regression (r = 0.85) outperformed a neural network (r = 0.63) for CO_2_ emission from piglet housing. Basak et al. [75] found ridge regression superior to a neural network and a random forest for methane from pig manure. Validation practice differs by method family in two directions. Cross validation is reported least often for deep learning studies (3 of 28, against 13.4% to 15.2% of the other families). Some form of external validation, however, is reported most often for them (8 of 28, 28.6%, against 10.4% to 26.5%; Supplementary Table S16). For structured animal, spatial and temporal data, the independence of the validation split matters more than either label. These counts therefore do not show that any family is evaluated with weaker safeguards.

### 3.7. The Decision Support Gap

#### 3.7.1. Claimed and Delivered Decision Support

**Figure 8:**
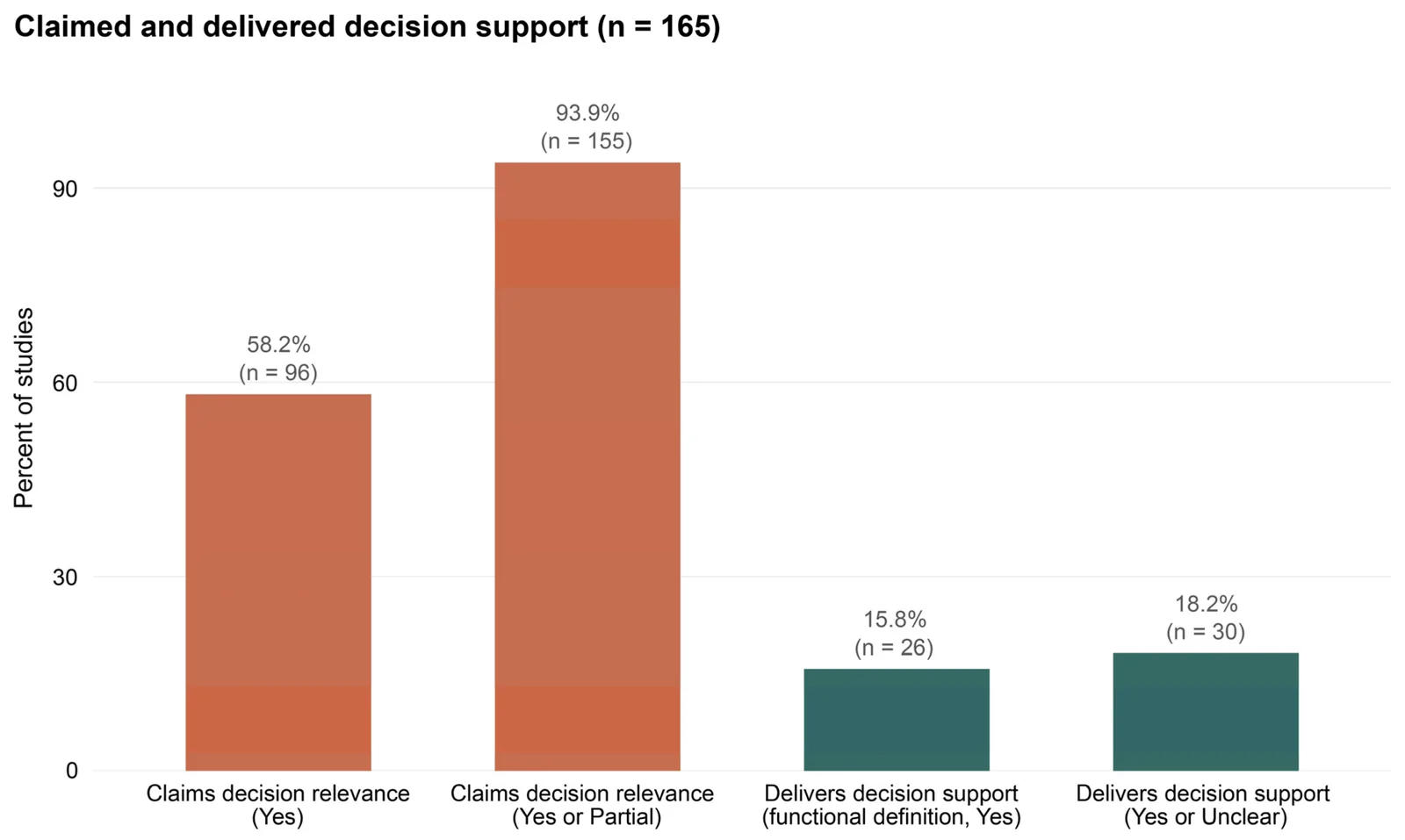
Claimed and delivered decision support. The primary contrast is between studies that explicitly claim decision relevance (Yes, 58.2%, *n* = 96) and studies that deliver it under the functional definition (Yes, 15.8%, *n* = 26), a difference of 42.4 percentage points (95% CI 33.7 to 51.2). The inclusive claim rate (Yes or Partial, 93.9%, *n* = 155) and the upper bound of delivery (Yes or Unclear, 18.2%, *n* = 30) are secondary figures; the inclusive rate lies 78.2 points (95% CI 71.9 to 84.5) above delivery. Denominator *n* = 165. Table 4 gives the full cross-tabulation.

Across the corpus, 96 of 165 studies (58.2%; 95% CI 50.6 to 65.4) explicitly claim that their work is relevant to producer, process, or policy decisions. Applying the functional definition of Section 2.7 to the full texts, 26 (15.8%; 95% CI 11.0 to 22.1) deliver decision support, 4 (2.4%) could not be settled either way, and 135 do not; counting the unclear studies as delivering gives an upper bound of 30 (18.2%). The primary contrast is therefore 58.2% claimed against 15.8% delivered, a difference of 42.4 percentage points (95% CI 33.7 to 51.2) (Table 4, Figure 8). A further 59 studies (35.8%) claim relevance only partially, so 155 (93.9%) make some claim; this inclusive rate is secondary because it mixes clear and partial claims. Of the 96 explicit claimants, 77 (80.2%) do not demonstrate the decision function they invoke, and 7 of the 26 delivering studies claimed relevance only partially, so claiming and delivering overlap only in part (Table 4).

#### 3.7.2. Systematicity Across Application Domains

**Figure 9:**
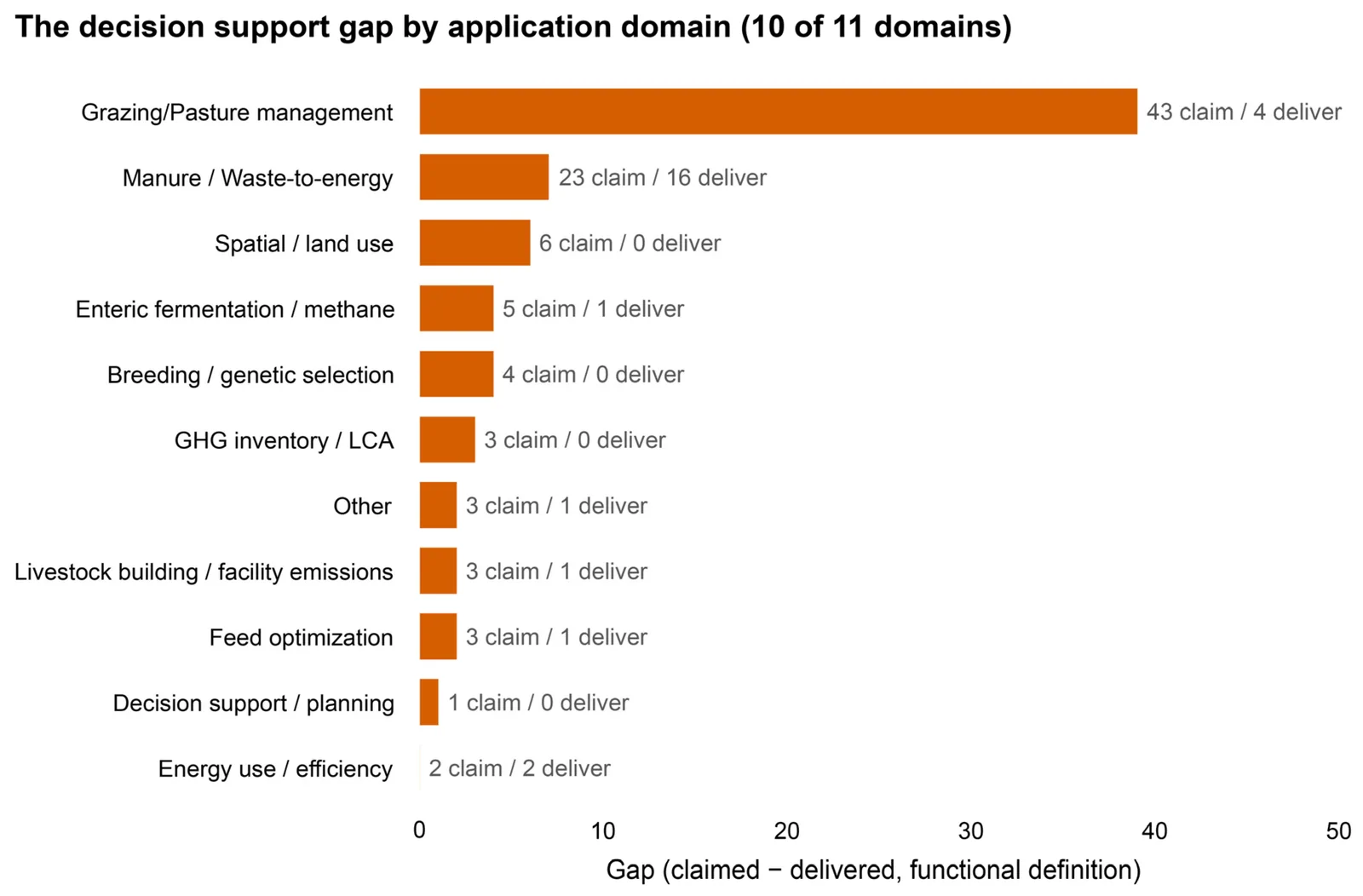
Difference between claimed and delivered decision support by canonical application domain under the functional definition, ordered by size of difference. The gap is present in 10 of 11 domains; energy use and efficiency shows no difference.

The gap between claimed and delivered decision support appeared in nearly every application domain (Table 5; Figure 9). In 10 of the 11 domains, more studies claimed decision support than met our functional definition of it. Energy use and efficiency was the only exception, where both studies that claimed decision relevance (2 studies) also met the definition. Relative to the number of claims, the gap was smallest in the manure and waste-to-energy domain (23 claimed, 16 delivered), which included most of the process-level optimization studies. Even when studies classified as ‘Unclear’ were counted as delivering decision support, the gap remained in those 10 domains.

Studies working at larger scales were more likely to claim decision relevance. Explicit claim rates were 100% for studies reporting outcomes at national level (5 studies), 70.0% for regional studies (50 studies), 61.4% for farm studies (57 studies), and 39.5% for animal-level studies (38 studies). This review counts policy and program decisions as decision support, so regional and national studies could qualify. However, these studies made the claim most often but delivered it least. Of the 50 regional studies, only 3 met the functional definition, and only 2 described a deployed system. In contrast, animal-level studies, which focus on the unit a producer directly manages, made the claim least often.

Claim rates also differed by domain. Domains that produce maps had high claim rates, including spatial land use (85.7%) and grazing (76.8%). Domains that produce coefficients or inventory values had low claim rates, including enteric fermentation (21.7%) and GHG inventory (37.5%). Several of these domains include only a small number of studies, so these rates should be interpreted with caution.

#### 3.7.3. Studies Meeting the Functional Definition

Table 6 profiles the 26 studies whose functional status is ‘Yes’, ordered by decision level, and records the stage each has reached. A blind second coding of all 165 studies agreed with the first on 95.2% to 100.0% of studies for each flag (kappa 0.66 to 1.00). Percent agreement was lowest for F1, names a decision (95.2%), and kappa was lowest for F5, evaluated in use, which only 4 studies meet. For the final status, agreement was 97.6% (kappa 0.92). The two codings differ on the status of 4 studies. Under the second coding 27 studies deliver and 5 are unclear, against 26 and 4 under the first. The headline count therefore does not depend on the coder (Supplementary Tables S35 and S36).

The decision support claim was also coded a second time for all 165 studies. A second coder worked from the full text under a written rule and, for 147 of the studies, was blind to the first coding (Supplementary Section S14, Tables S39 and S40). Agreement was much lower than for the functional flags. The two codings agreed on 101 of 165 studies (61.2%; kappa 0.24 over the three categories, 0.28 for explicit claim against the rest). Most of the 64 differences lie on the line between an explicit and a partial claim. In total, 37 studies moved from ‘Partial’ to ‘Yes’ and 11 from ‘Yes’ to ‘Partial’. On whether any claim is made at all, the codings agree on 149 studies (90.3%). The second coding was the more inclusive at the explicit level. It coded 119 studies (72.1%; 95% CI 64.8 to 78.4) as explicit claimants and 147 (89.1%) as making some claim, against 96 and 155 under the first coding. Under the second coding, the explicit claim rate exceeds delivery by 56.4 percentage points (95% CI 48.8 to 63.9) and the inclusive rate by 73.3 points (95% CI 66.6 to 80.1). The explicit rate exceeds delivery in 10 of the 11 application domains, as under the first coding. The gap therefore does not depend on the coder, and under the second coding it is wider. What depends on the coder is where a hedged statement of intended use stops being an explicit claim; this moves the explicit claim rate by about 13.9 percentage points. All 26 delivering studies are explicit claimants under the second coding (19 explicit and 7 partial under the first). The first coding remains the analysis coding. The limitation is stated in Section 4.5.

Of the 26, 11 inform a farm decision, 11 a process decision, and 4 a policy or program decision. Most of the process level studies find the operating conditions of a digester, composter, or treatment step by optimization over a fitted model. Seven of them work at bench or laboratory scale and one at pilot scale, so whether their optima transfer to operating plants is untested. The farm level studies cover feeding, grazing, cooling, ventilation, and energy inputs. Five studies illustrate the range of what is delivered. Long et al. [42] and Zou et al. [44] turn a fitted model into a search over operating conditions and return a recommended setting or a Pareto front. Ndlovu et al. [71] couple a learned surrogate to a life cycle model, search ten thousand breeding and forage scenarios, and return a specification rather than a prediction. Lima et al. [77] used drone maps of forage cactus to direct herd movement in the field and measured the gain in goat mass. Chen et al. [69] drove precision feeding from real time weight estimation on a commercial farm and measured the reduction in feed, manure, and carbon footprint. It is one of only two studies in the corpus in which a decision taken with the model was evaluated in operation.

Four studies could not be settled either way. They are reported as ‘Unclear’ rather than forced into a category. Xing et al. [90] feeds a digital twin without naming a decision. Rouegui et al. [91] states a recommendation but does not trace it to the model. Qi et al. [92] compares scenarios that bundle assumptions rather than a single lever. Silveira et al. [93] classifies bulls for selection without recommending any. Counting all four as delivering raises the count to 30 (18.2%) and changes no domain level conclusion (Table 5).

#### 3.7.4. Optimization and the Functional Definition

Across the corpus, the primary model purpose was prediction in 94 studies (57.0%), multiple purposes in 42 (25.5%), classification in 19 (11.5%), optimization in 7 (4.2%), and decision support in 3 (1.8%). Counted as a non-exclusive capability, an explicit search over alternatives appears in 17 studies (10.3%), and 16 of these are among the 26 delivering studies. This overlap is largely definitional rather than empirical, because an optimization procedure by construction evaluates alternative settings of a decision variable and returns a preferred one, which corresponds to criteria F2 and F3 of Section 2.7. The association therefore cannot be read as evidence that optimization causes delivered decision support. Nor is optimization necessary for delivery, since 10 of the 26 delivering studies meet the definition through scenario comparison or a stated recommendation without a formal search. The distribution supports a descriptive observation only. Prediction tasks are common (127 studies when counted as a non-exclusive capability), explicit searches over alternatives are uncommon, and most of those are process-level optimizations at bench scale.

#### 3.7.5. Practical Readiness

**Figure 10:**
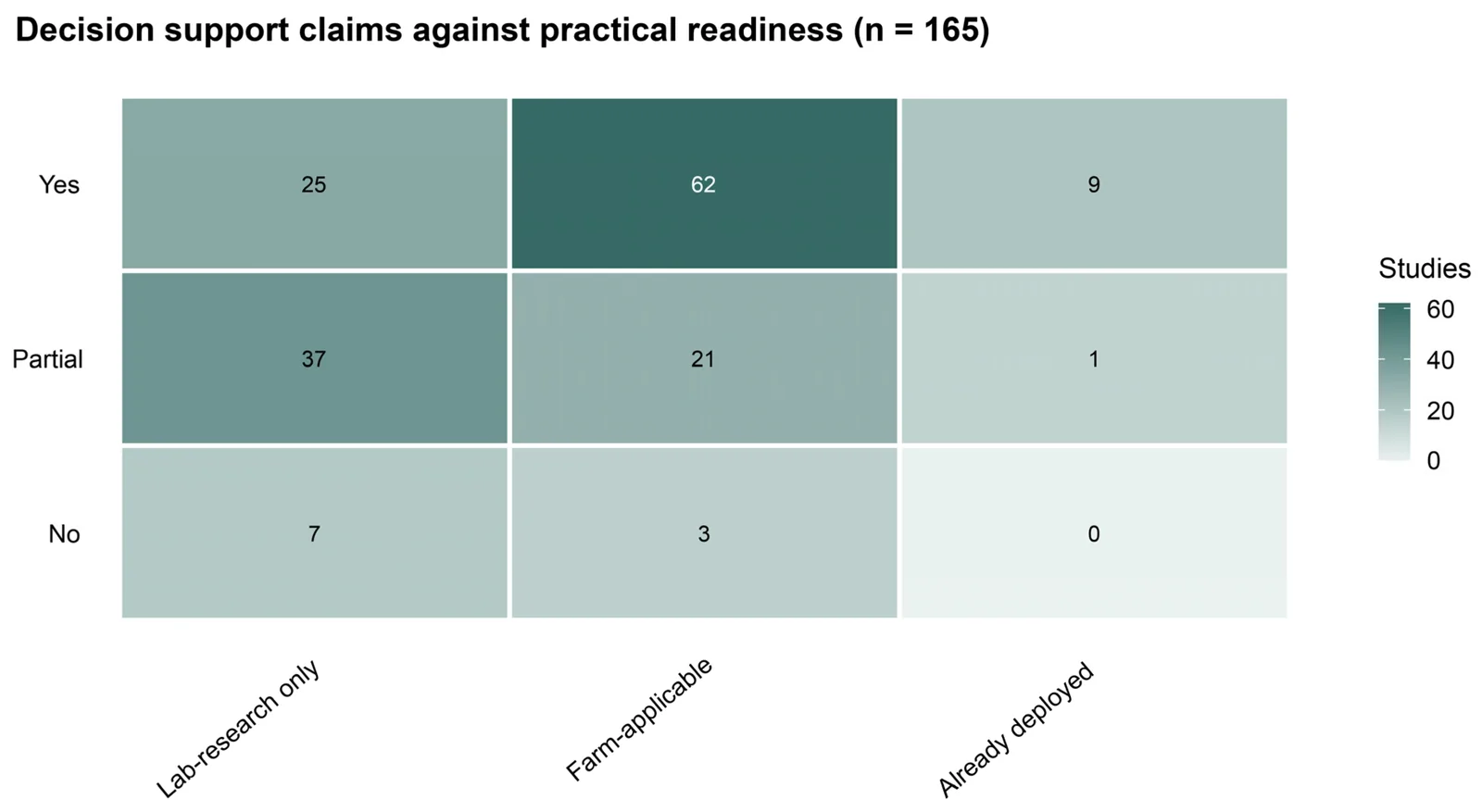
Decision support claim (No, Partial, Yes) against practical readiness (laboratory research only, farm applicable, already deployed), *n* = 165.

Cross tabulating the decision support claim against real world readiness gives a consistent picture (Figure 10).

Across the corpus, 85 studies (51.5%) describe outputs the authors judge could be applied on a farm. One further study was tested on a farm without being deployed and is counted with these in Figure 10. Another 69 (41.8%) describe work that has not left the laboratory or the dataset. Only 10 (6.1%) are coded as deployed, and this category groups different achievements: three public datasets; two evaluations of commercial products that serve monitoring rather than a decision; two full scale digesters whose data were modeled; one public web tool that has not been evaluated in use [79]; and two operational systems whose decisions were taken and whose effects were measured [69,77]. Among the 26 delivering studies, therefore, 3 provide an accessible tool or operational system, 2 were evaluated in operational use, and 2 measured the effect of the decision taken. Under our coding, those 2 of 165 included studies (1.2%) met F6, the criterion for measuring the real-world consequences of decisions based on the model or its outputs.

#### 3.7.6. Sensitivity Analysis

Excluding the 4 studies rated High concern on the composite leaves 161 studies. None of the 4 delivers decision support, so deliveries stay at 26 (16.1%), and the share of explicit claimants not meeting the definition moves from 80.2% to 79.3%, a change of less than one percentage point (Supplementary Table S6). Excluding instead the 10 studies rated Limited on reproducibility, the dimension with the best inter-rater agreement, leaves 155 studies with 23 deliveries (14.8%) and 81.6% of explicit claimants not meeting the definition; the gap remains in 10 of 11 application domains under both exclusions. Only one of the 26 delivering studies, Chasen et al. [79], was rated Low concern under the composite rule.

Because the functional definition was specified after the data were collected, two alternative definitions were written down and dated before being applied to any study (Supplementary Section S2). The broader, information-based definition counted a study when it named a decision (F1) and documented an output that the decision maker could act on for that decision, without requiring the model to compare or recommend actions. Seven studies had F1 coded ‘Yes’ without meeting the primary definition; three met the broader definition [36,91,94], one could not be settled because the level of the named decision is ambiguous [29], and three did not [67,95,96]. The stricter, implementation-oriented definition required the primary criteria plus an accessible tool (F4) or evaluation in use (F5); three studies met it [69,77,79]. Delivered decision support therefore ranges from 3 studies (1.8%) under the strict definition, through 26 (15.8%) under the primary definition, to 29 (17.6%; upper bound 30, 18.2%) under the broad definition, against an explicit claim rate of 58.2% and an inclusive rate of 93.9%. Under the broad definition the gap persists in 10 of 11 application domains and 74 of 96 explicit claimants (77.1%) still do not meet it (Supplementary Table S22).

## 4. Discussion

### 4.1. What the Field Currently Delivers

The principal result of this review is not the volume of publication but the distance between claim and delivery. In the corpus, 96 of 165 studies (58.2%) explicitly claim decision relevance while 26 (15.8%) meet a functional definition of delivering it, a difference of 42.4 percentage points (95% CI 33.7 to 51.2), and 155 (93.9%) make at least a partial claim. The gap is present in 10 of 11 application domains (Table 5), and it is essentially unchanged after exclusion of high concern studies, which is consistent with a structural property of the field rather than the idiosyncrasy of a small number of papers. The size of that distance depends on the definition, but not its existence. Under a broader definition that accepts decision-relevant information without a comparison or recommendation, 29 studies (17.6%) deliver; under a stricter definition that also requires a tool or evaluation in use, 3 (1.8%) do (Section 3.7.6).

The interpretation offered here is that decision support has functioned in this literature as motivating framing rather than as a design specification. A statement that a model will support decision making can make a study appear practically relevant in the introduction and conclusion, but this goal often does not shape how the model itself is designed. Research papers are typically judged on predictive accuracy, methodological novelty, and performance relative to existing approaches. Much less attention is given to the specific decision the model is meant to support, the alternatives a user must choose among, or the costs and consequences of the recommended action. As a result, decision support may remain part of the study’s framing while the actual research is optimized for a different objective.

This interpretation is consistent with previous reviews showing that precision livestock farming has focused largely on monitoring and prediction, while environmental sustainability and operational decision support remain less developed [10,13]. It also aligns with several decades of agricultural decision support research showing that predictive tools are often developed but rarely adopted when they are not designed around the decisions producers actually need to make [16,17,23,24]. The contribution of this review is to turn that qualitative observation into a measurable quantity with clearly defined denominators, allowing progress to be assessed rather than simply claimed.

Placed on the readiness scale that Ruchay et al. [18] propose for AI systems in livestock housing, the 26 delivering studies sit at the advisory level, where a recommendation is produced for a person to approve. The 3 with an accessible tool and the 2 evaluated in use reach the supervised execution level. None approaches closed-loop control. This distribution matches that review’s conclusion that AI in livestock production is presently more mature as a decision support layer than as an autonomous control technology.

### 4.2. Why Prediction Does Not Become Decision Support

Two regularities in Section 3 point in the same direction. Method family varies with the structure of the available input data more than with the application domain. Claim rates rise with reporting scale and with the legibility of the output, not with demonstrated delivery of decision support. One reading is that the field is organized around the data that happen to be available rather than around the decisions that require support. The cross-tabulations are consistent with that reading but cannot establish it, because they do not observe how investigators chose their methods. A more common explanation for low adoption of agricultural technologies is that producers are resistant to change. The adoption literature suggests that this explanation may be too simple.

Precision agriculture technologies that perform a clearly defined task automatically, such as global navigation satellite system (GNSS) guidance, have been adopted about as rapidly as any major agricultural technology on record. The technologies that struggle to gain adoption typically provide a recommendation that the producer must evaluate and choose whether to follow [97,98]. The technologies examined in this review fall largely into the second category, yet most do not actually produce a clear recommendation.

The adoption literature examines producers. This review examines the tools, and six features of the corpus bear directly on whether the tools could reasonably be trusted by a producer willing to try them.

First, the models have seldom been evaluated outside the context in which they were built. Thirty studies of 165 (18.2%) report some form of external validation, 9 of them on an independent farm, herd, region, or trial, and of the 81 studies that claim applicability beyond their own setting only 18 (22.2%) tested it. A producer’s expectation that a model calibrated elsewhere may not transfer is a reasonable reading of the available evidence rather than an expression of conservatism. Section 3.6 adds that geographic extent does not substitute for this. Of the 26 national scope studies, 8 claim applicability beyond their data and 1 tested it.

Reported accuracy calls for the same caution. A performance close to the theoretical maximum on a small dataset is evidence about model flexibility rather than about the state of the world. Two examples are the fuzzy biogas model fitted on thirteen experimental runs [72] and the national biomethane forecast with a correlation of exactly 1 on both partitions [73] (Section 3.6). Sample size should therefore be inspected before accuracy is interpreted. Studies in which a simpler method outperformed a more flexible one [74,75] are correspondingly informative.

Second, the output is generally a quantity rather than an option. An estimate of 4200 kg dry matter (DM) per ha standing biomass is not a decision; the decision concerns whether to move the herd, purchase feed, or adjust stocking. The translation is left to the user, unaided.

Third, objectives are rarely traded off. Farms are multi-objective systems in which environmental improvement carries cost and labor implications. Integrated economic environmental assessment was the primary sustainability focus in only 19 studies (11.5%). Most of the remaining studies optimized a single sustainability dimension without reporting the trade-offs or costs imposed on others.

Fourth, economic outcomes were rarely quantified. The few studies that reported monetary or proportional benefits therefore stand out. They include annual energy savings of USD 958.84 per farm [64], a predicted 12% increase in biogas output [42], a measured 7.8% reduction in feed consumption under farm conditions [69], and an estimated 75% reduction in cooling water use [80]. This lack of economic evidence is important because Barnes et al. [99] identified confidence in payback as a central factor in technology adoption.

Fifth, the data burden imposed on the user is rarely quantified. Nearly every model in this corpus requires inputs that a commercial farm may not routinely collect. Correa-Luna et al. [36] is particularly informative because it makes the implementation burden explicit by quantifying the additional rising plate meter measurements required each month. Few other studies report this practical cost so clearly.

Sixth, independent verification is often difficult. Only 37 studies (22.4%) shared code or data.

Together with the two regularities noted above, these patterns help explain what the current generation of tools actually provides. They do not demonstrate that low adoption is caused by tool design rather than producer behavior, because this review evaluated the tools themselves and did not study producers directly. It therefore cannot separate supply side limitations from demand side barriers. What it does show is that even a producer willing to adopt such technologies would encounter relatively few tools that clearly identify a decision, compare actionable alternatives, quantify costs, or demonstrate performance outside the original development setting. These are properties of tool design, and they can be addressed directly.

Several other patterns in Section 3 suggest explanations that this review did not test. First, national research output concentrates in particular domains (Section 3.2). These concentrations may follow national regulatory and infrastructural context, such as biogas incentive structures, extensive grazing systems with an established commercial decision tool market, and permitting regimes for concentrated animal feeding operations. Herd composition is an alternative explanation that the extracted data cannot separate from it. Second, research volume may track the cost of measurement rather than the size of the emission source. Enteric fermentation is the single largest emission source in ruminant production and attracts 23 studies, while grazing and pasture management attracts 56 (Section 3.4). Satellite imagery is free, global, and revisits every few days, so pasture state can be estimated at low marginal cost. Digesters are instrumented for operational reasons, so manure process data exist as a byproduct of production. Enteric methane requires respiration chambers, tracer techniques, or GreenFeed units and cannot be measured at scale on commercial farms. We did not extract a measurement cost variable, and other explanations, such as funding priorities or disciplinary tradition, would fit the same counts. Third, the coupling of species and domains (Section 3.5) is compatible with differences in production and housing systems. Manure and air outcomes may be more readily measured when manure is collected and air is enclosed, whereas landscape-related outcomes may be more observable in extensive systems. The coupling may also reflect data availability and cost, measurement feasibility, disciplinary practices, or biological differences among species; this review cannot distinguish among these explanations. Fourth, claim rates appear to track the legibility of the output rather than its actionability. Domains whose outputs are maps claim decision relevance far more often than those whose outputs are coefficients and inventory quantities (Section 3.7.2), although neither output constitutes a decision. Fifth, the corpus is spread over 99 journals with no single disciplinary home (Section 3.2). This may be one reason why a pattern visible only in aggregate, such as the gap reported here, is hard to see from within any single venue. Each of these is a reading of descriptive counts, offered as a hypothesis.

### 4.3. From Prediction to Integrated Decision Architectures

The distribution described in Section 3.7.4 suggests a design hypothesis, to be tested rather than a finding of this review. Adding an explicit comparison or search step to an existing predictive model is a direct route by which predictive work could become decision support.

A promising structural approach is to divide the task between two complementary model classes rather than expecting a single model to perform every function.

Machine learning and mechanistic models have different strengths and weaknesses. Machine learning models can identify complex relationships that are difficult to represent with explicit equations and, once trained, can generate predictions quickly. However, they do not inherently enforce physical constraints such as mass balance, may produce physically unrealistic predictions, and often perform poorly when applied outside the conditions represented in their training data. This limitation is particularly relevant given that only 30 of the 165 studies in this review tested a model outside the training context. An umbrella review of 112 reviews of computer vision in precision livestock farming reached the same diagnosis from a neighboring branch of the field [14]. Reported model performance consistently exceeded the documented evidence for farm deployment, and validation-critical items such as data splits and site description were among the least complete.

Mechanistic whole farm models offer complementary strengths. They represent biological, economic, and environmental processes using established process relationships, can enforce mass and energy balances, and are generally more interpretable. Because their core relationships are not fitted exclusively to a single dataset, they can also be more transferable across production contexts [19,20]. Their main limitations are the substantial effort required for parameterization and calibration and the computational cost of evaluating large numbers of management scenarios.

The two approaches can therefore compensate for one another’s weaknesses. Mechanistic models encode established process knowledge and constrain outputs through physical relationships such as conservation laws and feedbacks. Machine learning components can then represent processes that are difficult to describe mechanistically or serve as fast surrogate models that make it practical to explore much larger decision spaces.

This hybrid approach is already established in crop science. There, combining mechanistic models with data driven methods has often improved prediction and generalization compared with purely data driven approaches, although the size of the improvement varies across applications and contexts [21,22].

Three observations from this corpus support further evaluation of coupling mechanistic and ML models, although none is conclusive on its own. First, hybrid methods account for 33 of 165 studies (20.0%) and are concentrated in domains where process knowledge is relatively well developed but substantial unexplained variation remains. They appear in 8 of 23 studies on enteric fermentation (34.8%) and 12 of 47 studies on manure and waste to energy systems (25.5%). These studies also tend to produce physically interpretable outputs rather than dimensionless performance measures. Ten of 33 hybrids (30.3%) report GHG mass or inventory units, and 9 (27.3%) report biogas or energy yields. As shown in Supplementary Table S10, physical and spatial outputs are more closely associated with deployment than dimensionless ratios. When mechanistic, life cycle, or farm system coupling is counted as a specific capability rather than as a broad method category, it appears in 7 studies (4.2%), 4 of which are among the 26 studies classified as delivering decision support. Notably, however, no study in the decision support and planning domain uses a hybrid approach. The pattern is therefore visible in adjacent domains but has not yet been transferred to the area in which the decision support gap is greatest.

Second, hybrid methods concentrate at animal and farm scale rather than at regional scale. This is consistent with coupling needing a process model to couple to. A coupled model also carries its own calibration and validation requirements for the mechanistic component, which the corpus does not yet document.

Third, the seven coupled studies deliver decision support more often than the rest of the corpus, 4 of 7 against 22 of 158 (Table 7). That is a delivery rate about four times higher. This result needs strong caution, for three reasons. The first is that only seven studies are coupled, so the estimate is imprecise. The second is that most of the difference is explained by another feature, an optimization step. Nearly every study with an optimization step delivers decision support, coupled or not (2 of 2 coupled and 14 of 15 uncoupled). Among studies without one, the coupled studies still deliver more often (2 of 5 against 8 of 143), but that comparison rests on five studies. The third is that the coupling flag and the delivery status were coded by the same team from the same papers, and the comparison is between published studies, not between designs tested under the same conditions. Coupling is therefore a hypothesis worth testing, not a demonstrated advantage.

Two qualifications are important. First, the hybrid category is heterogeneous. It includes genuine coupling of ML with mechanistic models [71,100], but also looser algorithmic combinations such as metaheuristic hyperparameter tuning. The argument here concerns the former. Second, hybrid methods do not receive higher quality ratings in this corpus. High confidence ratings were assigned to 41.2% of ensemble studies (14 of 34), 31.3% of traditional ML studies (21 of 67), and 17.9% of deep learning studies (5 of 28). Hybrid studies received the lowest share, 15.2% (5 of 33). The case for hybrid coupling therefore does not rest on demonstrated quality superiority within this review. Rather, it rests on its structural fit to the decision problem, its association with physically meaningful outputs, and supporting evidence from crop system applications. Within this corpus it is a hypothesis to be tested, not an established finding.

Ndlovu et al. [71] provide a useful example of this architecture at working scale. A mechanistic life cycle assessment model supplied the accounting framework and physical relationships, while a learned surrogate provided the computational speed needed to search a large scenario space. The result was not simply a prediction, but a specification of an optimal forage ideotype with explicit targets for digestibility, protein, fertilizer use, and methane yield. Zou et al. [44] and Tryhuba et al. [43] similarly illustrate how multi-objective optimization can be incorporated at component and farm scales, respectively.

**Figure 11:**
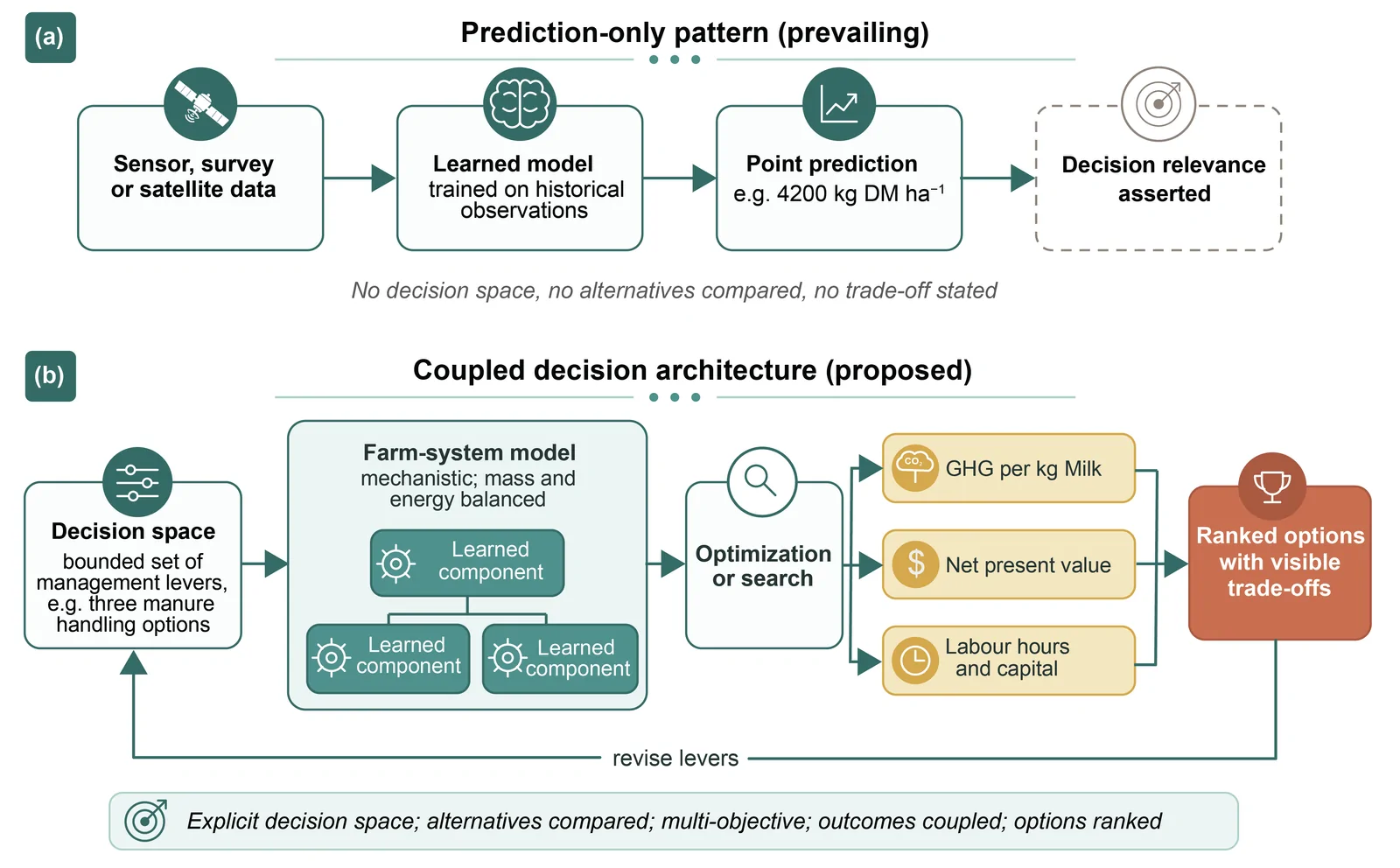
Contrast between the prevailing prediction only pattern and a coupled decision architecture. (**a**) Data are used to fit a learned model producing a point prediction, with decision relevance asserted rather than constructed. (**b**) An explicit decision space is evaluated through a farm system representation containing learned components, producing multi-objective outcomes and ranked options with visible trade offs.

The recurring pattern documented in Section 3.7 is that accurate prediction is presented as decision support without the components that would let a user choose among options. Table 8 sets out the components that an integrated sustainability decision tool would desirably contain, contrasted with the prevailing prediction only pattern. Figure 11 contrasts that pattern with the coupled architecture described above. The components are not the eligibility criteria used in Section 3.7, which required only a named decision and a model-based comparison or recommendation; they describe what a fuller tool would offer. The components also follow the progression that Ruchay et al. [18] describe for agentic systems, from passive sensing through advisory decision support to supervised and eventually safety-constrained action. The integrated tool described here corresponds to the advisory and supervised stages; closed-loop control is deliberately out of scope.

Assessed against these components, each of the 26 studies meeting the functional definition provides some and none provides all. The process level optimizations typically supply the decision space, alternatives, and a recommendation for one objective. The two farm-level studies that met F6 under our coding supply a recommendation and a measured decision outcome, without a multi-objective structure. That is a description of where the frontier presently sits rather than a criticism of those studies, which are among the more operationally useful in the corpus. The framework is offered as a design target and reporting aid, not as a scoring or exclusion instrument. No study in this review was excluded or classified on the basis of it.

The components can be illustrated briefly. Consider a dairy operation choosing between continuing to land-apply manure, installing a digester, or installing a digester with solids separation. It would need a farm system model in which a change in manure handling propagates to fertilizer purchase, field emissions, energy purchase, and labor. Learned components would be placed where mechanism is weakest, such as biogas yield from the actual feedstock [42]. The tool would return, for each option, emissions per kilogram of milk, net present value, capital requirement, and labor hours, each with an uncertainty range, and would show where the criteria conflict. Every technical component of that description exists within the studies reviewed here. They have not yet been assembled into a single instrument.

Such a tool would also need to be trusted, and that requirement is technical rather than presentational. It would require reporting uncertainty rather than point estimates, so that the model’s own confidence is visible. It would require attribution, so that a recommendation can be traced to the variables driving it, as provided by Nsabiyeze et al. [66] and Long et al. [42]. It would require an inspectable representation of the farm. This is an argument for mechanistic coupling on grounds of trust, separate from the accuracy argument above. An assumed dry-matter intake can be disputed by a producer in a way that a weight matrix cannot. And it would require honest performance reporting on the user’s context rather than on the training region. Hempel et al. [52,53] and Correa-Luna et al. [36] already demonstrate the principle by specifying how many days of measurement and how much calibration their tools require. That makes the limits of a tool legible, which is the precondition for trusting it within them.

### 4.4. Research Priorities

Several changes follow from the corpus evidence and are set out with the supporting observation in Table 9.

These priorities fall into three groups. The first is evidence of transferability: external validation on a different farm, region, or year, reported even where performance declines, since 30 studies of 165 is a norm rather than an oversight and norms change when reviewers and editors change what they request. The second is integration of technical outputs with the decisions they are meant to serve: explicit alternatives, economic consequences reported alongside environmental ones, the data burden a tool imposes stated in time, equipment, and labor, and an optimization step that turns a prediction into ranked options. The third is transparency: code and data sharing, which varies more than fourfold across application domains (Section 3.6) and which journals and funders can require, publication of null comparisons, and a reporting standard for what a claim of decision relevance entails, toward which Table 8 is offered on the model of guidance developed for clinical prediction [31].

### 4.5. Strengths and Limitations

This review has several strengths. The protocol was registered. Reporting follows PRISMA 2020. Data were extracted with a 47-field template and an auditable cleaning log. Study quality was assessed with a structured instrument adapted to computational prediction studies. And the central gap was measured with an explicit, reproducible definition against stated denominators.

The review also has limitations. The claim rate reflects how authors framed their own work, not an independent judgment, so it should be read as a measure of stated intent. A second coding of the claim field from the full text agreed with the first on 61.2% of studies (kappa 0.24). The two codings differed mainly on where an explicit claim ends and a partial one begins. Under the second coding 72.1% of studies claimed explicitly, against 58.2% under the first. The gap holds under both codings, but the explicit claim rate itself is uncertain by about 13.9 percentage points (Section 3.7.3).

The review departed from its registered protocol in ways listed, with reasons, in Supplementary Section S2, Table S13. The scope was narrowed to AI and ML, two registered databases were replaced, Google Scholar was not used, and the registered meta-analysis was replaced by a narrative synthesis. The registered species-specific exception to the outcome criterion was not applied, and the four goat studies it would have admitted [102,103,104,105] were excluded under E3 (Supplementary Section S2, amendment 6). Most importantly, the central endpoint, the gap between claimed and delivered decision support, was not registered. Its definition was fixed after data extraction, so the headline result is exploratory.

Each study was extracted by one reviewer. Several fields were coded a second time: the functional flags, the claim field, the input structure variable, three quality dimensions, and the stated transportability target. The last two were re-coded on a sample of 40 studies; for the stated target, agreement was 62.5% (kappa 0.44). The other extraction fields, including application domain, practical readiness, and external validation, were coded once and were not independently checked. Full texts were also screened by one reviewer each, so the 143 full text exclusions were not independently checked.

The search has known gaps. The search strings were narrower than the vocabulary of the included studies. An expanded re-run and citation tracking of the included studies both indicate that a substantial number of potentially eligible studies were not retrieved (Supplementary Sections S12 and S15). Three sources named at registration (Scopus, AGRICOLA and Google Scholar) were not searched, and the overlap assumed with the searched databases was not tested directly. The librarian who reviewed the search noted that the livestock terms omit several species (for example horses, rabbits, turkeys, ducks and camelids) and that Compendex and the ProQuest Agricultural and Environmental Science Collection would extend coverage. The search was not updated after 9 January 2026, and coverage of 2026 is partial. The corpus of all 165 included studies [8,28,29,30,36,37,38,39,40,41,42,43,44,45,46,47,48,49,50,51,52,53,54,55,56,57,58,59,60,61,62,63,64,65,66,67,68,69,70,71,72,73,74,75,76,77,78,79,80,81,82,83,84,85,86,87,88,89,90,91,92,93,94,95,96,100,101,106,107,108,109,110,111,112,113,114,115,116,117,118,119,120,121,122,123,124,125,126,127,128,129,130,131,132,133,134,135,136,137,138,139,140,141,142,143,144,145,146,147,148,149,150,151,152,153,154,155,156,157,158,159,160,161,162,163,164,165,166,167,168,169,170,171,172,173,174,175,176,177,178,179,180,181,182,183,184,185,186,187,188,189,190,191,192,193,194,195,196,197,198,199,200,201,202,203] is a reproducible but not exhaustive sample of the eligible literature. The records missed are of the same kinds as the included studies, so the comparison between claimed and delivered decision support is unlikely to change in direction.

Several coding and analysis choices also limit the findings. The hybrid category is heterogeneous. The quality instrument was designed for this review rather than taken from a validated tool; its ratings involve judgment, and the second rating reproduced the data quality rating no better than chance (Section 3.6). The canonical variables rest on written rules together with 22 recorded judgment overrides. Countries were counted by the first country listed, so the 10 studies that name several countries are each attributed to one. No pooled synthesis was attempted. Finally, the explanations offered in Section 4.2, which attribute patterns to policy context, measurement cost, housing system, or output legibility, are interpretations of descriptive cross tabulations and admit alternative explanations.

## 5. Conclusions

Research applying artificial intelligence and machine learning to livestock sustainability has grown quickly. Four in five dated studies in our 2010–2026 search window were published between 2022 and 2025. The field has produced work of lasting value, such as multi-decade gridded grazing datasets, methane prediction from milk samples already collected for other purposes, and optimization of a commercial-scale digester. It is also methodologically narrow. Random forests and shallow neural networks account for more than half of the studies, and the choice of method follows the structure of the available data more than the application domain. The central finding is a large gap between claimed and delivered decision support. Of the 165 included studies, 96 (58.2%) explicitly claim that their work is relevant to producer, process, or policy decisions. Only 26 (15.8%) name a decision and use their model to compare alternatives or recommend an action. Of these 26, three provide an accessible tool, and two measure the real-world effect of a decision taken with the model. Because this definition was fixed after data extraction, the finding is exploratory. The gap is nevertheless consistent. It appears in 10 of 11 application domains, holds under a broader and a stricter definition and under a blind second coding, and is essentially unchanged when lower-quality studies are excluded. Finally, half of the studies claim that their model applies beyond the setting in which it was built, yet fewer than one in five test it outside that setting.

These results imply that predictive accuracy, the measure on which most studies are judged, is not by itself evidence of usefulness for decisions. A producer or planner seeking support in this literature would find few tools that specify the decision, compare actionable alternatives, state the costs and data burden of use, or demonstrate performance outside the setting in which they were developed. Whatever role producer attitudes play in adoption, these shortcomings are properties of how tools are designed and reported. Researchers, reviewers, and funders can therefore address them directly. Until a study demonstrates the decision function it describes, a claim of decision relevance is best read as a statement of intent.

Four research priorities follow. First, external validation on farms, regions, or years not used for training should become standard practice and should be reported even where performance declines. Second, studies that claim decision relevance should name the decision and the controllable lever, compare alternatives, and report economic as well as environmental consequences, together with the data and labor needed to use the tool. Third, coupling learned models with mechanistic farm system models should be evaluated as a route to transferable, multi-objective decision tools. Coupling occurs more often among the studies that deliver decision support than in the rest of the corpus. But only seven studies are coupled, and the difference largely reflects their optimization step, so this is a hypothesis to be tested rather than a demonstrated advantage. Fourth, such tools should be developed and tested in underrepresented species and production systems; sheep, goats, and other species together account for 22 of the 165 studies. Wider sharing of code and data, encouraged through journal and funder policy, would allow each of these advances to be independently verified.

## Figures and Tables

**Table 1:** Eligibility criteria, exclusion codes, and full-text exclusions. Codes were applied in the order shown, recording only the first applicable reason. Unretrieved reports (E6) are counted separately from assessed and excluded reports, following PRISMA 2020.

Code	Criterion Not Met	Applied When	Full Text Exclusions (*n*)
E1	AI or ML as a core method	No method that learns from data and predicts beyond the cases it was fitted on (three-test rule, Section 2.2), or such a method applied only to a peripheral analytical step within a feeding trial, wet laboratory experiment or metagenomic analysis	14
E2	Livestock as the subject	Focus on crops, aquaculture, wildlife or insects; model and decision confined to a stage after the farm gate (slaughter, meat or dairy processing, packing, retail or consumption); or livestock present only as background context such as building energy management or soil science on former pasture	29
E3	Sustainability outcome measured	No environmental, resource efficiency or integrated sustainability outcome measured, predicted or assessed, including studies using sustainability terminology while measuring only production metrics	81
E4	Original research	Review articles, meta-analyses, editorials, commentaries, perspectives, conference abstracts, book chapters and bibliometric analyses	18
E5	English language	Full text not available in English	0
E6	Full text unavailable	Full text not obtainable through any available means	2
E7	Peer reviewed journal article	Preprints and other reports that have not passed peer review	1
		Total full text exclusions	143

Note. Another 1684 records were excluded during title and abstract screening without exclusion codes.

**Table 2:** Functional flags used to code delivered decision support (*n* = 165). Each flag was coded as Yes, No, or Unclear based on the full text of each study. The headline functional status was determined from F1–F3.

Flag	Definition	Studies Coded Yes, *n* (%)
F1. Names a decision	Identifies a specific decision and a controllable management, design, or policy lever for an identifiable decision-maker at the farm, process, or policy/program level. General claims of supporting management or decision-making were coded No unless both the decision and controllable lever were specified.	33 (20.0%)
F2. Compares alternatives	Uses the model, or an optimization or simulation directly based on model outputs, to prospectively evaluate two or more alternative settings of the decision lever identified in F1. Studies were coded No if the models only measured or predicted outcomes within an experimental comparison, evaluated decisions retrospectively, or varied only external conditions without changing a controllable decision lever.	27 (16.4%)
F3. Recommends or controls	Uses model output to identify a preferred option, ranked or targeted set of options, target value, operating point, or control rule for the specified decision.	24 (14.5%)
F4. Accessible tool	Provides an implemented tool, software package, web application, commercial product, or operational system giving intended users access to the model or its decision support output without retraining or rebuilding the model. Research prototypes unavailable for practical use were coded No.	6 (3.6%)
F5. Evaluated in use	Reports evaluation of the model or its output used by, or on behalf of decision makers in a real operational setting. Laboratory validation, purely simulated deployment, and technical model validation without operational use were coded No.	4 (2.4%)
F6. Outcome measured	Measures the real-world consequences of model-based decisions or its output. Effects that were only estimated, simulated, or projected were coded No.	2 (1.2%)
Functional status Yes (headline)	F1 = Yes and at least one of F2 or F3 = Yes.	26 (15.8%)
Functional status Unclear	The available information was insufficient to determine whether the study satisfied either the Yes or No classification rule.	4 (2.4%)
Upper bound (Yes or Unclear)	Sensitivity analysis in which studies coded Unclear were treated as Yes.	30 (18.2%)
Sensitivity definition B (broader)	F1 = Yes and a documented output the decision maker could act on for that decision, without requiring F2 or F3; specified before coding.	29 (17.6%)
Sensitivity definition C (stricter)	Functional status Yes and F4 = Yes or F5 = Yes.	3 (1.8%)

**Table 3:** Canonical application domains with representative included studies (*n* = 165).

Application Domain	*n* (%)	Characteristic Data and Method	Representative Studies
Grazing and pasture management	56 (33.9)	Satellite and drone imagery; random forest, CNN	[36,37,38,39,40]
Manure and waste to energy	47 (28.5)	Digester operating data; ANN, gradient boosting, hybrid	[41,42,43,44,45]
Enteric fermentation and methane	23 (13.9)	Diet composition, milk spectra, rumen microbiome	[46,47,48,49]
GHG inventory and LCA	8 (4.8)	Census and satellite data; regional accounting	[50,51]
Housing and facility emissions	7 (4.2)	In barn sensor time series; LSTM, gradient boosting	[52,53,54,55]
Spatial and land use	7 (4.2)	High resolution imagery; CNN classification	[56,57,58]
Breeding and genetic selection	6 (3.6)	Genomic, spectral and microbiome data	[59,60,61]
Feed optimization	4 (2.4)	Intake and efficiency measurements	[62]
Other	3 (1.8)	Varied	[63]
Energy use and efficiency	3 (1.8)	Farm energy input inventories	[64,65,66]
Decision support and planning	1 (0.6)	Farm panel records; matrix completion	[29]

Note. Abbreviations: ANN, artificial neural network; CNN, convolutional neural network; LCA, life cycle assessment; LSTM, long short-term memory.

**Table 4:** Decision support claim by functional status (*n* = 165). Claim is the study’s own framing of decision relevance, coded Yes (explicit), Partial, or No. Functional status applies the definition of Section 2.7 (Yes, Unclear, No). Share delivering counts Unclear as No; 95% confidence intervals (CI) use the Wilson method.

Claim of Decision Relevance	Studies	Delivered: Yes	Delivered: Unclear	Delivered: No	Share Delivering, % (95% CI)
Yes (explicit)	96	19	4	73	19.8 (13.1 to 28.9)
Partial	59	7	0	52	11.9 (5.9 to 22.5)
No	10	0	0	10	0.0 (0.0 to 27.8)
Total	165	26	4	135	15.8 (11.0 to 22.1)

Note. Primary contrast: explicit claim 58.2% (95% CI 50.6 to 65.4) against functional delivery 15.8% (95% CI 11.0 to 22.1), a paired difference of 42.4 percentage points (95% CI 33.7 to 51.2), based on 77 studies that claim without delivering and 7 that deliver without an explicit claim. Secondary contrast: inclusive claim (Yes or Partial) 93.9% (95% CI 89.2 to 96.7) against the same delivery rate, a difference of 78.2 points (95% CI 71.9 to 84.5). The No row is small, so its interval is wide.

**Table 5:** Claimed and delivered decision support by application domain (*n* = 165). Claimed decision support refers to studies that identified decision support as a focus. Delivered decision support refers to studies that met the functional definition described in Section 2.7. Studies classified as Unclear are presented separately. The difference represents the number of studies claiming decision support minus the number delivering it. It is reported under two assumptions: treating Unclear studies as No (the primary estimate) and as Yes (the upper-bound estimate).

Application Domain	Total	Claim (Yes)	% Claiming	Delivered (Yes)	Unclear	Difference (Headline)	Difference (Upper Bound)
Grazing and pasture management	56	43	76.8	4	0	39	39
Manure and waste to energy	47	23	48.9	16	1	7	6
Spatial and land use	7	6	85.7	0	0	6	6
Enteric fermentation and methane	23	5	21.7	1	1	4	3
Breeding and genetic selection	6	4	66.7	0	1	4	3
GHG inventory and LCA	8	3	37.5	0	0	3	3
Housing and facility emissions	7	3	42.9	1	0	2	2
Other	3	3	100	1	1	2	1
Feed optimization	4	3	75	1	0	2	2
Decision support and planning	1	1	100	0	0	1	1
Energy use and efficiency	3	2	66.7	2	0	0	0
Total	165	96	58.2	26	4	70	66

Note. The Partial and No claim categories are cross-tabulated in Table 4. Of the 26 delivering studies, 19 are explicit claimants and 7 are Partial claimants.

**Table 6:** The 26 studies meeting the functional definition of delivered decision support, by decision level. Flags F1 to F6 are shown in order as Y (Yes) or N (No or Unclear). Stage records whether an accessible tool exists (F4), whether use was evaluated in an operational setting (F5), and whether the effect of decisions taken with the output was measured (F6). Composite quality is the composite rating from the methodological quality and reporting assessment.

Study	Year	Country	Decision Level	Decision Informed and Output	F1 to F6	Stage	Confidence	Composite Quality
Elahi et al. [65]	2019	Pakistan	Farm	Target input levels for feed, minerals, and electricity on buffalo farms from a neural network; 30.5% energy saving	YYYNNN	none	Medium	Some concerns
Elahi et al. [64]	2022	Pakistan	Farm	Target energy inputs per poultry farm from a neural network; 318 MJ per flock and 959 USD per farm per year saved	YYYNNN	none	Medium	Some concerns
Xie et al. [55]	2022	United States	Farm	Ventilation control action from a proposed fuzzy controller driven by CNN LSTM predicted gas concentrations; simulated, no comparison of strategies	YNYNNN	none	Medium	Some concerns
Gao et al. [76]	2023	China	Farm	Recommended grazing intensity (3 sheep per day per ha) and duration (85 to 104 days) from modeled scenarios	YYYNNN	none	Medium	Some concerns
Guarnido-Lopez et al. [30]	2023	Spain	Farm	When to switch diets in multiphase feeding from changepoint detection; multiphase against single diet simulated; 16% less N excretion, 44% more CH_4_	YYYNNN	none	Medium	Some concerns
Lima et al. [77]	2023	Brazil	Farm	Where to move the goat herd from CNN drone maps of forage cactus; system operated in the field and goat mass measured (71 to 86 kg)	YYYYYY	tool, in use, measured	Medium	Some concerns
Altshuler et al. [78]	2025	Israel	Farm	Which farms to target with a methane reducing feed additive from a microbiome model; targeted against uniform deployment as modeled scenarios on 14 farms	YYYNNN	none	Medium	Some concerns
Chasen et al. [79]	2025	United States	Farm	Continuous against rotational grazing compared through a random forest yield model released as a public web tool; no recommendation issued	YYNYNN	tool	High	Low concern
Chen et al. [69]	2025	China	Farm	Feed quantity set by real time CNN weight estimation; precision fed group compared with conventional feeding on a commercial farm; 7.8% less feed, 5.1% lower carbon footprint measured	YYYYYY	tool, in use, measured	Medium	Some concerns
Tryhuba et al. [43]	2025	Ukraine	Farm	Farm configuration and feeding ration chosen by multi criteria optimization over a neural network model; 16% lower CO_2_-eq and 7 to 16% more biogas on a 900 head farm	YYYNNN	none	Medium	Some concerns
Valle et al. [80]	2025	United States	Farm	When to activate cooling sprinklers from YOLO cow presence detection; presence based against continuous operation; water saving of up to 75% estimated, not measured	YYYNNN	none	Medium	Some concerns
Yusof et al. [81]	2014	Malaysia	Process	Ratio, initial pH, and temperature for poultry manure and food waste co digestion from RSM and a neural network; optimum stated; laboratory	YYYNNN	none	Medium	Some concerns
Rico-Contreras et al. [82]	2017	Mexico	Process	Whether to invest in reducing poultry litter moisture before combustion; fuzzy moisture model feeding Monte Carlo economics for two scenarios	YYYNNN	none	Medium	Some concerns
Reza et al. [83]	2022	United States	Process	pH, irradiation time, and power for microwave ammonia stripping from digested dairy manure from RSM and a neural network; optimum stated; bench	YYYNNN	none	Medium	Some concerns
Ahmad et al. [84]	2023	India	Process	Mixing ratio, retention time, and temperature for cattle dung and food waste co digestion from a neural network with RSM and a genetic algorithm; 2 cubic meter pilot digester	YYYNNN	none	Medium	Some concerns
Okwu et al. [72]	2023	South Africa	Process	Blend ratio and retention time for cow dung and poultry waste digestion from fuzzy and neural network models fitted on 13 runs; laboratory	YYYNNN	none	Low	Some concerns
Olatunji et al. [85]	2023	South Africa	Process	Mixing ratio, temperature, and retention time for duck waste co digestion from ANFIS and RSM; optimum 75:25; laboratory	YYYNNN	none	Medium	Some concerns
Şenol et al. [86]	2024	Türkiye	Process	Biochar dose and pretreatment for cattle manure and algae co digestion from a neural network with RSM; optimum doses recommended for pilot testing; laboratory	YYYNNN	none	Medium	Some concerns
Zaki et al. [87]	2024	United States	Process	Which manure conversion route to adopt; pyrolysis against hydrothermal carbonization with random forest, life cycle, cost, and social scores	YYYNNN	none	Medium	Some concerns
Zou et al. [44]	2024	China	Process	Operating conditions of a microbial electrolysis cell digester (voltage, spacing, substrate, pH) from NSGA II over a neural network; Pareto front; 2 L bench reactor	YYYNNN	none	Medium	Some concerns
Long et al. [42]	2025	United States	Process	Operating temperature and pH of a commercial digester from optimization over a random forest model; 12% more biogas, 65% less H_2_S predicted; not implemented	YYYNNN	none	High	Some concerns
Wu et al. [88]	2025	China	Process	Composting setpoints for moisture, aeration, and temperature from an optimized ensemble; validated in a composting experiment; 35% less NH_3_	YYYNNN	none	Medium	Some concerns
Jeong et al. [45]	2022	United States	Policy or program	Which dairies to target for anaerobic digesters; 162 large facilities identified from U-Net herd size estimates and per facility reduction potential	YYYNNN	none	Medium	Some concerns
Vaysi et al. [63]	2024	Iran	Policy or program	Which manure energy pathway to pursue at provincial scale; biomethane substitution against biogas electricity and baseline on neural network forecasts; biomethane favored	YYYNNN	none	Medium	Some concerns
Ndlovu et al. [71]	2025	Ireland	Policy or program	Forage and cattle trait targets for a breeding program selected by search over 10,000 life cycle scenarios with an XGBoost surrogate; 36.7% lower global warming potential	YYYNNN	none	Medium	Some concerns
Wu et al. [89]	2025	China	Policy or program	Pasture protection level in forestation programs; no, moderate, and strict protection compared for carbon and livestock on random forest tree cover projections	YYNNNN	none	Medium	Some concerns

Note. Of the 26 studies, 16 contain an explicit optimization step, 4 couple a learned model to a life cycle, process, or farm system model, 4 are coded as deployed systems, and 3 report external validation. 8 use hybrid methods, 6 deep learning, 5 traditional ML, 5 ensembles, and 2 fuzzy logic. 2 achieve High overall confidence (Long, Chasen), none is rated High concern on the composite quality rating and 1 (Chasen) is rated Low concern. Abbreviations: ANFIS, adaptive neuro-fuzzy inference system; CNN, convolutional neural network; LSTM, long short-term memory; NSGA-II, non-dominated sorting genetic algorithm II; RSM, response surface methodology; YOLO, You Only Look Once (an object detection model).

**Table 7:** Mechanistic coupling by delivered decision support (*n* = 165). Coupling is the capability flag for a learned model linked to a life cycle, process, or farm system model (Supplementary Table S16); delivered is the functional definition of Section 2.7, with Unclear counted as No. Share delivering with Wilson 95% confidence interval (CI).

Mechanistic Coupling	Studies	Delivered: Yes	Delivered: No	Share Delivering, % (95% CI)
Yes	7	4	3	57.1 (25.0 to 84.2)
No	158	22	136	13.9 (9.4 to 20.2)
Total	165	26	139	15.8 (11.0 to 22.1)

Note. Risk ratio 4.10 (95% CI 1.94 to 8.68); risk difference 43.2 percentage points; Fisher exact p = 0.012. Split by the presence of an optimization step: with one, 2 of 2 coupled and 14 of 15 uncoupled studies deliver; without one, 2 of 5 coupled and 8 of 143 uncoupled studies deliver. The association is descriptive and does not show that coupling causes delivery. The coupled studies are [67,69,71,87,89,100,101].

**Table 8:** Desirable components of an integrated sustainability decision tool, contrasted with the prevailing prediction only pattern.

Component	Prediction Only Pattern	Integrated Decision Tool (Desirable)
Decision space	Implicit or unstated; the output is an estimate	An explicit, bounded set of management levers the user can alter
Alternatives	A single scenario or the observed condition	Several management configurations simulated and compared
Objective structure	One accuracy metric on one outcome	Multiple stated objectives, including at least one environmental and one economic
Outcome coupling	A standalone statistical estimate	Outcomes propagated through a farm system representation so that trade offs are visible
Recommendation	Prediction reported; usefulness asserted	Options ranked against stated criteria with the reasoning inspectable
Transferability	Context specific, single site	Architecture designed for relocation to other systems, regions and scales
Cost of use	Not reported	Data burden, labor requirement and payback period stated

**Table 9:** Research priorities arising from this review, with the supporting corpus observation.

Horizon	Priority	Supporting Observation
Immediate	Report external validation as standard, including where performance declines	30 of 165 studies (18.2%) report some external validation and 9 report it on an independent site; of the 81 studies that claim applicability beyond their own setting, 18 (22.2%) tested it, and of the 8 national scope studies that claim it, 1 tested it
Immediate	State the data burden a tool imposes in time, equipment and labor	Quantified explicitly by Correa-Luna et al. [36] and almost nowhere else
Immediate	Report economic consequences alongside environmental outcomes	Integrated economic environmental assessment is the primary category of 19 (11.5%)
Immediate	Publish negative and null comparisons	Rodriguez et al. [74] and Basak et al. [75], in which simpler methods outperformed flexible ones, are more informative than a marginal gain
Near term	Evaluate coupled learned and mechanistic farm system models, with their own calibration and validation, and report the result as a decision tool	Hybrids cluster where process knowledge is deepest and produce physical outputs; mechanistic coupling appears in 7 studies, 4 of them among the 26 delivering studies, and in none of the decision support domain (0 of 1)
Near term	Build and evaluate multi-objective outputs including ranked options and Pareto fronts	An optimization step appears in 17 of 165 studies (10.3%) and in 16 of the 26 delivering studies, most of them at bench scale; Zou et al. [44] and Tryhuba et al. [43] demonstrate feasibility at component and farm scale
Near term	Move code and data sharing toward a majority through journal and funder policy	Sharing rose from 8.3% (2014 to 2018) to 25.0% (2022 to 2024) and varies fourfold across domains; the reasons were not assessed
Medium term	Develop and test decision architectures in underserved species and production systems	Sheep, goats and other species together account for 22 of 165 studies (13.3%); no study in the decision support and planning domain uses a hybrid method
Medium term	Establish reporting norms for decision relevance	58.2% of the corpus explicitly claims decision relevance (93.9% counting partial claims) and 15.8% meets a functional definition of delivering it, with no accepted standard for what the claim entails

## Data Availability

The Open Science Framework project https://osf.io/hsvyu (accessed on 30 October 2026) holds the registration record and, under a Creative Commons Attribution 4.0 International (CC BY 4.0) license, the analysis materials (version 2026-10-30): the frozen extraction workbook, including the cleaning log, the canonical mapping tables, the functional decision support coding and the capability flags; the executed search strings and the record sets exported on 9 January 2026; the Python code (Python 3.13.3, Python Software Foundation, Wilmington, DE, USA; openpyxl 3.1.5) that regenerates the canonical variables (canonical_rules.py); and the R code and data that generate all figures, with SHA-256 checksums of every file. The second codings, the quality and transportability ratings and the screening logs of the search checks are available from the corresponding author on request.

## Supplementary Materials

The following supporting information can be downloaded at: https://www.mdpi.com/article/10.3390/ani16213502/s1, Table S1: Publication year distribution of the 165 included studies; Table S2: Machine learning category by publication period; Table S3: Machine learning category by outcome unit family; Table S4: Hybrid method distribution by canonical application domain; Table S5: Primary species by canonical application domain; Table S6: Sensitivity analysis excluding the 4 studies rated High concern on the composite quality rating and the 10 studies rated Limited on reproducibility; Table S7: Quality dimensions, composite quality rating and inter-rater agreement across the corpus; Table S8: Code and data sharing by canonical application domain; Table S9: Decision support claim and practical readiness by measurement level; Table S10: Practical readiness by outcome unit family; Table S11: Data extraction fields; Table S12: Canonical value sets applied to extracted free text fields; Figure S1: Sustainability outcome unit families by primary livestock species; Figure S2: Decision support capability flags F1 to F5 and their intersections; Figure S3: Validation strategies as non exclusive flags; Figure S4: Frequency of reported performance metrics; Figure S5: Geographic distribution of included studies; Figure S6: Machine learning category by application domain; Figure S7: Algorithm families by application domain; Figure S8: Highest reported R^2^ by sample size bin; Table S13: Deviations from the registered protocol; Table S14: Outcomes for the registered hypotheses; Table S15: Characteristics of all 165 included studies [8,28,29,30,36,37,38,39,40,41,42,43,44,45,46,47,48,49,50,51,52,53,54,55,56,57,58,59,60,61,62,63,64,65,66,67,68,69,70,71,72,73,74,75,76,77,78,79,80,81,82,83,84,85,86,87,88,89,90,91,92,93,94,95,96,100,101,106,107,108,109,110,111,112,113,114,115,116,117,118,119,120,121,122,123,124,125,126,127,128,129,130,131,132,133,134,135,136,137,138,139,140,141,142,143,144,145,146,147,148,149,150,151,152,153,154,155,156,157,158,159,160,161,162,163,164,165,166,167,168,169,170,171,172,173,174,175,176,177,178,179,180,181,182,183,184,185,186,187,188,189,190,191,192,193,194,195,196,197,198,199,200,201,202,203]; Table S16: Capability flag definitions, derivation rules, and counts; Table S17: Definitions of terms used in the review; Table S18: Method families represented in the corpus with a corpus example; Table S19: Performance metrics reported in the corpus; Table S20: Profile of the included corpus; Table S21: Method family by structure of input data; Table S22: Sensitivity of delivered decision support to alternative definitions; Table S23: Stated transportability target by test outside the training context; Table S24: Transportability by geographic scope; Table S25: SWiM reporting items, where each is addressed, and how they were adapted; Table S26: Reviews of AI and ML in livestock farming identified by a targeted search, with the level at which each examines what became of the models; Table S27: Retrieval of the included studies by database; Table S28: Search terms matched in the included studies; Table S29: Expanded re-run of the search with a screened sample; Table S30: PRESS self-assessment of the search strategy; Table S31: Potentially eligible records found by the expanded re-run and absent from the original search; Table S32: Cleaning log entries by class; Table S33: Judgement overrides recorded in the cleaning log; Table S34: Country counts under three counting conventions; Table S35: Agreement between the first and the blind second coding of the functional flags and the final status; Table S36: Studies on which the two codings of the functional flags differ; Table S37: Citation tracking of the included studies, from seeds to screened sample; Table S38: Potentially eligible records found by citation tracking and absent from the original search; Table S39: Agreement between the first and the second coding of the decision support claim; Table S40: Studies on which the two codings of the decision support claim differ. Sections S1 to S15: S1, electronic search strings; S2, registration and protocol amendments; S3, PRISMA 2020 checklist; S4, supporting distributions and cross tabulations; S5, data extraction fields and canonical value sets; S6, supplementary figures; S7, evidence table for all included studies; S8, included studies; S9, capability flag definitions and sensitivity definitions; S10, additional tables; S11, targeted search for earlier reviews; S12, sensitivity of the search strategy; S13, canonicalization rules, recorded judgement overrides and a check of the derived variables; S14, independent second coding of the functional flags and of the decision support claim; S15, citation tracking of the included studies.
