# Supplementary material for: From Prediction to Practice: A Systematic Review of the Decision Support Gap in Artificial Intelligence and Machine Learning for Environmentally Sustainable Livestock Production
Source: Animals (Basel). 2026 Nov 5;16(21):3502. doi: 10.3390/ani16213502 (PMC13761973; doi:10.3390/ani16213502)
Supplement: Supplementary file 1 [file animals-16-03502-s001.zip › PRISMA_2020_checklist.pdf]

PRISMA 2020 Checklist

Section and Topic	Item #	Checklist item	Location where item is reported
**TITLE**			
Title	1	Identify the report as a systematic review.	Title page (manuscript title); the phrase "A Systematic Review" appears in the title.
**ABSTRACT**			
Abstract	2	See the PRISMA 2020 for Abstracts checklist.	Abstract (a single unstructured paragraph, as the journal requires, covering the PRISMA 2020 for Abstracts items); Simple Summary.
**INTRODUCTION**			
Rationale	3	Describe the rationale for the review in the context of existing knowledge.	Sections 1.1 and 1.2.
Objectives	4	Provide an explicit statement of the objective(s) or question(s) the review addresses.	Section 1.3.
**METHODS**			
Eligibility criteria	5	Specify the inclusion and exclusion criteria for the review and how studies were grouped for the syntheses.	Section 2.2; Table 1 (eligibility criteria and exclusion codes E1 to E7, applied in a fixed hierarchy; E6 records full texts that could not be retrieved). Written rules adopted at revision for E1 (AI or ML as a core method; Supplementary Section S2, amendment 10) and E2 (farm gate; amendment 9). Grouping for synthesis is described in Sections 2.5 and 2.8.
Information sources	6	Specify all databases, registers, websites, organisations, reference lists and other sources searched or consulted to identify studies. Specify the date when each source was last searched or consulted.	Section 2.3 (four databases: Web of Science Core Collection, CAB Abstracts via EBSCO, IEEE Xplore and PubMed; search date 9 January 2026; English language journal articles from January 2010 onward; Scopus, AGRICOLA and Google Scholar, named at registration, were not searched, with the reasons stated); Supplementary Section S1 (platform, field, limits and record count for each database); Supplementary Section S11 (OpenAlex search for earlier reviews, 26 October 2026); Supplementary Section S15 (backward and forward citation tracking of the included studies in OpenAlex, 26 October 2026, as a coverage check).
Search strategy	7	Present the full search strategies for all databases, registers and websites, including any filters and limits used.	Section 2.3 (base search string with its three concept blocks and the limits applied); the string as executed in each database in Supplementary Section S1; the strategy tested at revision by a known-

PRISMA 2020 Checklist

Section and Topic	Item #	Checklist item	Location where item is reported
			item check, term matching, an expanded re-run and a PRESS self-assessment in Supplementary Section S12.
Selection process	8	Specify the methods used to decide whether a study met the inclusion criteria of the review, including how many reviewers screened each record and each report retrieved, whether they worked independently, and if applicable, details of automation tools used in the process.	Section 2.4 (two stage screening in Rayyan; titles and abstracts of all unique records screened independently by two reviewers, with conflicts resolved by discussion; full texts divided among five reviewers and each screened once against a written guideline, with decisions and exclusion codes recorded in a shared template; included studies re-examined at revision under written rules).
Data collection process	9	Specify the methods used to collect data from reports, including how many reviewers collected data from each report, whether they worked independently, any processes for obtaining or confirming data from study investigators, and if applicable, details of automation tools used in the process.	Section 2.5 (47 fields; five reviewers divided the studies between them and worked from a shared written guide; each study was extracted by one reviewer and no study was extracted twice; one author then checked every extracted value against written cleaning rules and logged each change, Supplementary Section S13; unlocated values recorded as not reported rather than inferred). At revision, all 165 studies were re-coded by a second coder on the functional flags F1 to F6 (Supplementary Section S14) and on input structure (Section S13), the decision support claim field was re-coded by a second coder from the full text (Section S14, Tables S39 and S40), and 40 studies were re-rated on the three quality dimensions (Table S7); the other fields were coded once (Section 4.5).
Data items	10a	List and define all outcomes for which data were sought. Specify whether all results that were compatible with each outcome domain in each study were sought (e.g. for all measures, time points, analyses), and if not, the methods used to decide which results to collect.	Sections 2.5 and 2.7; full field list in Supplementary Table S11; canonical value sets in Table S12; definitions of terms in Supplementary Table S17.
	10b	List and define all other variables for which data were sought (e.g. participant and intervention characteristics, funding sources). Describe any assumptions made about any missing or unclear information.	Section 2.5; Supplementary Table S12 (canonical value sets applied to free text fields) and Section S13 (mapping rules, recorded judgement overrides and checks of the derived variables); unlocated values recorded as not reported.
Study risk of bias assessment	11	Specify the methods used to assess risk of bias in the included studies, including details of the tool(s) used, how many reviewers assessed each study and whether they worked independently, and if applicable, details of automation tools	Section 2.6 (three dimensions adapted to computational prediction studies, each

Section and Topic	Item #	Checklist item	Location where item is reported
		used in the process.	with written signalling questions; composite rating derived by rule; second rating of 40 studies by a second reviewer, blind to the first); Supplementary Table S7 (distributions and inter-rater agreement); Supplementary Section S2, amendment 8.
Effect measures	12	Specify for each outcome the effect measure(s) (e.g. risk ratio, mean difference) used in the synthesis or presentation of results.	Not applicable. No meta-analysis was performed and no effect measures were computed. Results are counts and percentages; denominators are stated explicitly in Sections 2.7 and 2.8.
Synthesis methods	13a	Describe the processes used to decide which studies were eligible for each synthesis (e.g. tabulating the study intervention characteristics and comparing against the planned groups for each synthesis (item #5)).	Sections 2.5 and 2.8 (canonical variables used for all counting; synthesis proceeds from descriptive distributions to cross tabulations).
	13b	Describe any methods required to prepare the data for presentation or synthesis, such as handling of missing summary statistics, or data conversions.	Section 2.5 (mapping of free text entries onto canonical value sets, recorded in an auditable cleaning log; rules, overrides and checks in Supplementary Section S13); Section 2.8 (denominator conventions).
	13c	Describe any methods used to tabulate or visually display results of individual studies and syntheses.	Section 2.8; figures produced in R 4.5.0 with ggplot2. Tables and figures throughout Section 3; supporting cross tabulations in Supplementary Section S4; additional tables in Supplementary Section S10 and figures in Section S6.
	13d	Describe any methods used to synthesize results and provide a rationale for the choice(s). If meta-analysis was performed, describe the model(s), method(s) to identify the presence and extent of statistical heterogeneity, and software package(s) used.	Sections 2.1 and 2.8. Narrative synthesis reported following the Synthesis Without Meta-analysis (SWiM) guideline (Supplementary Table S25 maps the nine items); meta-analysis was not appropriate given heterogeneity in species, algorithm and outcome. Rationale also given in Supplementary Section S2, amendment 4.
	13e	Describe any methods used to explore possible causes of heterogeneity among study results (e.g. subgroup analysis, meta-regression).	Not applicable. No meta-analysis was performed, so no statistical exploration of heterogeneity was undertaken. Variation across species, method family, application domain and measurement level is described descriptively in Sections 3.3 to 3.6.
	13f	Describe any sensitivity analyses conducted to assess robustness of the synthesized results.	Sections 2.6, 2.7 and 3.7.6. The central

Section and Topic	Item #	Checklist item	Location where item is reported
			findings were re-estimated with the 4 studies rated High concern on the revised composite excluded and, separately, with the 10 studies rated Limited on reproducibility excluded (Supplementary Table S6); under two alternative definitions of delivered decision support written down before coding (Supplementary Table S22); and with unclear studies counted as delivering, giving an upper bound (Section 3.7.1). The goat eligibility exception and its sensitivity analysis were withdrawn at revision (Supplementary Section S2, amendment 6).
Reporting bias assessment	14	Describe any methods used to assess risk of bias due to missing results in a synthesis (arising from reporting biases).	Not applicable. Formal assessment of risk of bias due to missing results (for example funnel plot asymmetry or small study effects) requires a meta-analysis and was not undertaken. Study level reporting completeness is captured instead within the quality assessment described in Section 2.6.
Certainty assessment	15	Describe any methods used to assess certainty (or confidence) in the body of evidence for an outcome.	Not applicable. GRADE and comparable certainty frameworks were not applied, as the review synthesizes methodological and descriptive characteristics rather than intervention effect estimates. Study level quality and risk of bias were assessed instead (Section 2.6).
**RESULTS**			
Study selection	16a	Describe the results of the search and selection process, from the number of records identified in the search to the number of studies included in the review, ideally using a flow diagram.	Section 3.1 and Fig. 1 (PRISMA 2020 flow diagram): 2,664 records identified; 670 duplicates removed; 1,994 screened; 1,684 excluded at title and abstract; 310 full texts sought, 2 not retrieved, 308 assessed; 143 excluded with reasons; 165 studies included.
	16b	Cite studies that might appear to meet the inclusion criteria, but which were excluded, and explain why they were excluded.	Section 3.1 and Table 1. The 143 full text exclusions are reported by reason under the E1 to E7 hierarchy (six exclusion reasons; E6 is the retrieval status): no measured sustainability outcome (81), not livestock (29), not original research (18), not AI or ML (14), not a peer reviewed journal article (1); 2 reports could not be

PRISMA 2020 Checklist

Section and Topic	Item #	Checklist item	Location where item is reported
			retrieved.
Study characteristics	17	Cite each included study and present its characteristics.	Sections 3.2 to 3.6; Supplementary Table S15 (evidence table, one row per included study); all 165 included studies are cited in the reference list and listed in Supplementary Section S8.
Risk of bias in studies	18	Present assessments of risk of bias for each included study.	Section 3.6 and Fig. 7; Supplementary Table S7 (the three quality dimensions, the composite rating and inter-rater agreement across the corpus); composite rating of each delivering study in Table 6.
Results of individual studies	19	For all outcomes, present, for each study: (a) summary statistics for each group (where appropriate) and (b) an effect estimate and its precision (e.g. confidence/credible interval), ideally using structured tables or plots.	Supplementary Table S15 (characteristics and extracted values for each of the 165 included studies, drawn directly from the analysis workbook); highest reported R2 per study in Supplementary Figure S8.
Results of syntheses	20a	For each synthesis, briefly summarise the characteristics and risk of bias among contributing studies.	Sections 3.2 to 3.7; risk of bias among contributing studies summarized in Section 3.6.
	20b	Present results of all statistical syntheses conducted. If meta-analysis was done, present for each the summary estimate and its precision (e.g. confidence/credible interval) and measures of statistical heterogeneity. If comparing groups, describe the direction of the effect.	Sections 3.2 to 3.7. No meta-analysis was performed. Syntheses are reported as counts and percentages with Wilson 95% confidence intervals against stated denominators, a paired difference between the claim and delivery rates with its confidence interval (Table 4), and one risk ratio with a Fisher exact test for mechanistic coupling by delivery (Table 7) (Tables 3 to 7, Figs. 2 to 10, Supplementary Tables S1 to S10 and S20 to S24).
	20c	Present results of all investigations of possible causes of heterogeneity among study results.	Not applicable. No meta-analysis was performed and no statistical investigation of heterogeneity was conducted.
	20d	Present results of all sensitivity analyses conducted to assess the robustness of the synthesized results.	Section 3.7.6; Supplementary Tables S6 and S22.
Reporting biases	21	Present assessments of risk of bias due to missing results (arising from reporting biases) for each synthesis assessed.	Not applicable. See item 14.
Certainty of evidence	22	Present assessments of certainty (or confidence) in the body of evidence for each outcome assessed.	Not applicable. See item 15.
**DISCUSSION**			
Discussion	23a	Provide a general interpretation of the results in the context of other evidence.	Sections 4.1, 4.2 and 4.3.
	23b	Discuss any limitations of the evidence included in the review.	Section 4.5.

PRISMA 2020 Checklist

Section and Topic	Item #	Checklist item	Location where item is reported
	23c	Discuss any limitations of the review processes used.	Section 4.5; Supplementary Sections S12 (sensitivity of the search strategy), S13 (canonical variables) and S14 (reliability of the functional coding).
	23d	Discuss implications of the results for practice, policy, and future research.	Sections 4.3, 4.4 and 5.
**OTHER INFORMATION**			
Registration and protocol	24a	Provide registration information for the review, including register name and registration number, or state that the review was not registered.	Section 2.1. Registered with the Open Science Framework in September 2025, registration ysw5q, https://doi.org/10.17605/OSF.IO/YSW5Q. Also stated in Supplementary Section S2.
	24b	Indicate where the review protocol can be accessed, or state that a protocol was not prepared.	Section 2.1; Supplementary Section S2. The registered protocol is publicly accessible at the OSF record above.
	24c	Describe and explain any amendments to information provided at registration or in the protocol.	Section 2.1; Supplementary Section S2 (ten amendments in the order in which they arose); Supplementary Table S13 (deviations from the registered protocol); Supplementary Table S14 (outcomes for the registered hypotheses).
Support	25	Describe sources of financial or non-financial support for the review, and the role of the funders or sponsors in the review.	Funding statement (back matter): U.S. Department of Agriculture, National Institute of Food and Agriculture, Agriculture and Food Research Initiative Sustainable Agricultural Systems program, grant number 2024 68012 41749. The Conflicts of Interest statement records that the sponsors had no role in the design, execution, interpretation, or writing of the study.
Competing interests	26	Declare any competing interests of review authors.	Conflicts of Interest statement (back matter). The authors declare no conflict of interest.
Availability of data, code and other materials	27	Report which of the following are publicly available and where they can be found: template data collection forms; data extracted from included studies; data used for all analyses; analytic code; any other materials used in the review.	Data Availability Statement (back matter). OSF project https://osf.io/hsvyu (CC BY 4.0) holds the registration record and the analysis materials (version 2026-10-30), with SHA-256 checksums of every file. Search strings in Supplementary Section S1; data collection form and canonical value sets in Supplementary Section S5 (Tables S11 and S12); data extracted from included studies in Supplementary Section S7 (Table S15); the frozen extraction

PRISMA 2020 Checklist

Section and Topic	Item #	Checklist item	Location where item is reported
			workbook with its cleaning log, mapping tables, functional coding and capability flags, the executed search strings and the exported record sets, the mapping rules as code (canonical_rules.py), and the R code and data for every figure are deposited there. The second codings, the quality and transportability ratings and the screening logs of the search checks are available from the corresponding author on request.

From: Page MJ, McKenzie JE, Bossuyt PM, Boutron I, Hoffmann TC, Mulrow CD, et al. The PRISMA 2020 statement: an updated guideline for reporting systematic reviews. BMJ 2021;372:n71. doi: 10.1136/bmj.n71. This work is licensed under CC BY 4.0. To view a copy of this license, visit https://creativecommons.org/licenses/by/4.0/
