# Supplementary material for: From Prediction to Practice: A Systematic Review of the Decision Support Gap in Artificial Intelligence and Machine Learning for Environmentally Sustainable Livestock Production
Source: Animals (Basel). 2026 Nov 5;16(21):3502. doi: 10.3390/ani16213502 (PMC13761973; doi:10.3390/ani16213502)
Supplement: Supplementary file 1 [file animals-16-03502-s001.zip › Supplementary_Materials_revised_highlighted.pdf]

Supplementary Materials

Supplementary material for: *From Prediction to Practice: A Systematic Review of the Decision Support Gap in Artificial Intelligence and Machine Learning for Environmentally Sustainable Livestock Production*

Amir Mohammad Shadjou[a], Saghar Mohsenian[b], Parham Vatankhah[c], Farzam Hosseinpour Vardin[a], Uttama Deb[b], Fatemeh Taghvaei[a], Dongyi Wang[a], Marty Matlock[a,*]

[a] *Department of Biological and Agricultural Engineering, University of Arkansas, Fayetteville, AR 72701, USA*
[b] *Environmental Dynamics Ph.D. Program, Graduate School and International Education, University of Arkansas, Fayetteville, AR 72701, USA*
[c] *School of Biomedical Engineering, College of Science and Engineering, Flinders University, Adelaide, SA, Australia*

[*] *Corresponding author: Marty Matlock, Department of Biological and Agricultural Engineering, University of Arkansas, Fayetteville, AR 72701, USA. Email: mmatlock@uark.edu*

Registration: Open Science Framework, ysw5q (*https://doi.org/10.17605/OSF.IO/YSW5Q*).

Archived copy: *https://archive.org/details/osf-registrations-ysw5q-v1*

Contents of this supplement

Table S1. Publication year distribution of the 165 included studies.

Table S2.. Machine learning category by publication period.

Table S3.. Machine learning category by outcome unit family.

Table S4. Hybrid method distribution by canonical application domain.
Table S5.. Primary species by canonical application domain.
Table S6. Sensitivity analysis excluding the 4 studies rated High concern on the composite quality rating and the 10 studies rated Limited on reproducibility.
Table S7.. Quality dimensions, composite quality rating and inter-rater agreement across the corpus.
Table S8.. Code and data sharing by canonical application domain.

Table S9.. Decision support claim and practical readiness by measurement level.
Table S10.. Practical readiness by outcome unit family.
Table S11. Data extraction fields.
Table S12. Canonical value sets applied to extracted free text fields.
Figure S1.. Sustainability outcome unit families by primary livestock species.
Figure S2. Decision support capability flags F1 to F5 and their intersections across the 165 studies.
Figure S3. Validation strategies counted as non exclusive flags.
Figure S4. Frequency of reported performance metrics.
Figure S5. Geographic distribution of included studies.
Figure S6. Machine learning category by application domain.
Figure S7. Algorithm families by application domain.
Figure S8. Highest reported R2 by sample size bin.
Table S13. Deviations from the registered protocol (OSF ysw5q).
Table S14. Outcomes for the registered hypotheses (OSF ysw5q).
Table S15. Characteristics of all 165 included studies.
Table S16. Functional decision support flags and capability flags: definitions, derivation rules, and counts.
Table S17. Definitions of terms used in the review.
Table S18. Method families represented in the corpus with a corpus example.
Table S19. Performance metrics reported in the corpus.
Table S20. Profile of the included corpus.
Table S21. Method family by structure of input data.
Table S22. Sensitivity of delivered decision support to alternative definitions, overall and by application domain.
Table S23. Stated transportability target by test outside the training context.
Table S24. Transportability by geographic scope of the data.
Table S25. SWiM reporting items, where each is addressed, and how they were adapted.
Table S26. Reviews of AI and ML in livestock farming identified by a targeted search, with the level at which each examines what became of the models.
Table S27. Retrieval of the included studies by database.
Table S28. Search terms matched in the title, abstract and keywords of the included studies.
Table S29. Expanded re-run of the search in PubMed and OpenAlex with a screened random sample of the additional records.
Table S30. PRESS self-assessment of the search strategy.
Table S31. Potentially eligible records found by the expanded re-run and absent from the original search.
Table S32. Entries of the cleaning log by class.
Table S33. Judgement overrides recorded in the cleaning log.
Table S34. Country counts under three counting conventions.
Table S35. Agreement between the first and the blind second coding of the functional flags and the final status.

Table S36. Studies on which the two codings of the functional flags differ.
Table S37. Citation tracking of the included studies, from seeds to screened sample.
Table S38. Potentially eligible records found by citation tracking and absent from the original search.
Table S39. Agreement between the first and the second coding of the decision support claim.
Table S40. Studies on which the two codings of the decision support claim differ.

This document contains the electronic search strategy (S1), the registration record and protocol amendments (S2), the PRISMA 2020 checklist location (S3), supporting distributions and cross tabulations summarized in the main text (S4, Tables S1 to S10), the data extraction field definitions and canonical value sets (S5, Tables S11 and S12), eight supplementary figures (S6, Figures S1 to S8), the complete evidence table for all 165 included studies (S7, Table S15), the full list of those 165 included studies (S8), the flag definitions (S9, Table S16), the definitions of terms, the method and metric summaries, the corpus profile, the input structure cross tabulation, and the sensitivity definitions table cited in the main text (S10, Tables S17 to S25), the targeted search for earlier reviews (S11, Table S26), the sensitivity analysis of the search strategy (S12, Tables S27 to S31), the canonicalization rules, the recorded judgement overrides and a check of the derived variables (S13, Tables S32, S33 and S34), the second codings of the functional flags and of the decision support claim (S14, Tables S35, S36, S39 and S40), and the citation tracking of the included studies (S15, Tables S37 and S38). Section, table, figure and study numbering run as four separate series, so Section S5, Table S5, Figure S1 and study S5 are unrelated items; study identifiers S1 to S170 refer only to entries in the S8 list, five of which (S45, S77, S89, S135 and S166) are vacant. All counts derive from the frozen analysis workbook and match the figures reported in the main text. Percentages use the full corpus denominator of 165 studies unless stated otherwise.

S1. Electronic search strings

The search was executed on 9 January 2026 in four databases. The Boolean logic combined an AI and ML concept block, a livestock species block and a sustainability outcome block. The canonical string, adapted to the syntax of each database, was as follows; the exact executed string for each database, with its interface, filters and record count, is given below.

Block 1 (AI and ML): ("artificial intelligence" OR "machine learning" OR "deep learning" OR "neural network*" OR "random forest" OR "support vector machine" OR "gradient boosting" OR "XGBoost" OR "reinforcement learning")

AND Block 2 (livestock): (livestock OR dairy OR cattle OR cow* OR bovine OR goat* OR caprine OR sheep OR ovine OR pig* OR swine OR porcine OR poultry OR chicken* OR "farm animal*")

AND Block 3 (sustainability): (sustainab* OR "life cycle assessment" OR "life cycle analysis" OR LCA OR "carbon footprint" OR "greenhouse gas" OR GHG OR methane OR "environmental impact" OR "water footprint" OR "water use" OR "energy use" OR "economic viability" OR "profitability" OR "production efficiency")

Filters applied: English language; publication years 2010 to 2026; document type journal articles. Conference papers were retrievable from IEEE Xplore at the search stage and were excluded during screening under the journal articles only scope. Records identified per source: Web of Science 909; CAB Abstracts 1,264; IEEE Xplore 65; PubMed 426; total 2,664.

Web of Science. Web of Science Core Collection (Clarivate), Advanced Search, Topic field, which searches title, abstract, author keywords and Keywords Plus. Limits as above (publication years 2010 to 2026, English, document type article). Records: 909. String as run:

("artificial intelligence" OR "machine learning" OR "deep learning" OR "neural network*" OR "random forest" OR "support vector machine" OR "gradient boosting" OR "XGBoost" OR "reinforcement learning") AND (livestock OR dairy OR cattle OR cow* OR bovine OR goat* OR caprine OR sheep OR ovine OR pig* OR swine OR porcine OR poultry OR chicken* OR "farm animal*") AND (sustainab* OR "life cycle assessment" OR "life cycle analysis" OR LCA OR "carbon footprint" OR "greenhouse gas" OR GHG OR methane OR "environmental impact" OR "water footprint" OR "water use" OR "energy use" OR "economic viability" OR "profitability" OR "production efficiency")

CAB Abstracts. CAB Abstracts with Full Text (CABI) on the EBSCOhost platform, Advanced Search with three search boxes combined with AND and no field selected (the platform default, which searches the citation, abstract and subject fields). Limits as above. Records: 1,264. Boxes as run:

Box 1: "artificial intelligence" OR "machine learning" OR "deep learning" OR "neural network*" OR "random forest" OR "support vector machine" OR "gradient boosting" OR "XGBoost" OR "reinforcement learning"

Box 2: livestock OR dairy OR cattle OR cow* OR bovine OR goat* OR caprine OR sheep OR ovine OR pig* OR swine OR porcine OR poultry OR chicken* OR "farm animal*"

Box 3: sustainab* OR "life cycle assessment" OR "life cycle analysis" OR LCA OR "carbon footprint" OR "greenhouse gas" OR GHG OR methane OR "environmental impact" OR "water

footprint" OR "water use" OR "energy use" OR "economic viability" OR "profitability" OR "production efficiency"

IEEE Xplore. IEEE Xplore (IEEE), Advanced Search in the All Metadata field (title, abstract, index terms and other bibliographic metadata, not full text). The platform does not accept truncation inside quoted phrases, so the wildcards of the base string were removed and single word forms were used. Limits as above (year range 2010 to 2026; journal articles; all 65 exported records are journal articles). Records: 65. String as run:

("artificial intelligence" OR "machine learning" OR "deep learning" OR "neural network" OR "random forest" OR "support vector machine" OR "gradient boosting" OR "XGBoost" OR "reinforcement learning") AND (livestock OR dairy OR cattle OR cow OR bovine OR goat OR caprine OR sheep OR ovine OR pig OR swine OR porcine OR poultry OR chicken OR "farm animal") AND (sustainability OR "life cycle assessment" OR "life cycle analysis" OR LCA OR "carbon footprint" OR "greenhouse gas" OR GHG OR methane OR "environmental impact" OR "water footprint" OR "water use" OR "energy use" OR "economic viability" OR profitability OR "production efficiency")

PubMed. PubMed (National Library of Medicine), main search box; unquoted terms are expanded by automatic term mapping and quoted terms are searched as phrases. Filters: English, journal article, publication date 2010 to 2026. Records: 426 (460 before the filters, from the results by year export). Compared with the base string, the PubMed string omitted XGBoost in the AI and ML block; cow*, caprine, pig*, porcine and farm animal* in the livestock block; and life cycle analysis, LCA, GHG, water use, energy use, economic viability and profitability in the sustainability block. PubMed does not support truncation inside quoted phrases, which explains

the truncated terms; the other terms were dropped at execution. These omissions reduce the sensitivity of the PubMed search and are recorded here as a limitation. String as run:

("artificial intelligence" OR "machine learning" OR "deep learning" OR "neural network" OR "random forest" OR "support vector machine" OR "gradient boosting" OR "reinforcement learning") AND (livestock OR dairy OR cattle OR bovine OR goat OR sheep OR ovine OR swine OR poultry OR chicken) AND (sustainability OR "life cycle assessment" OR "carbon footprint" OR "greenhouse gas" OR methane OR "environmental impact" OR "water footprint" OR "production efficiency")

The exported record sets of 9 January 2026 (RIS, CSV and NBIB files) are deposited with the analysis materials; their record counts are the counts given above. Search strings are reproduced with straight quotation marks as typed into each platform.

S2. Registration and protocol amendments

The review was registered with the Open Science Framework in September 2025 *https://doi.org/10.17605/OSF.IO/YSW5Q*. Ten amendments to the registered protocol are described below in the order in which they arose, each with its date. A concise summary of amendments 1–5, including the timing of each change relative to screening, data extraction, and analysis, is provided in Section 2.1 of the main text. The full details are retained below.

1. Scope. The registered protocol covered three method families: farm system modeling, life cycle assessment, and artificial intelligence or optimization. The present review reports the artificial intelligence and machine learning component only. Studies applying mechanistic modeling or life cycle assessment without a machine learning component were excluded under

code E1. The narrowing was made because the three families proved to have distinct literatures, eligibility boundaries and quality criteria, and combining them would have produced a corpus that could not be characterized coherently.

2. Information sources. The registered sources were Scopus, Web of Science, AGRICOLA and CAB Abstracts, with supplementary searching in Google Scholar. Scopus was not accessible to the team through an institutional subscription. AGRICOLA, which is freely accessible, was not searched because its agricultural coverage was judged to be largely duplicated by CAB Abstracts; that judgement was not tested. Google Scholar was dropped after trial searches returned several hundred thousand records that could not be exported or screened reproducibly. IEEE Xplore and PubMed were added to extend coverage of the engineering and biomedical literature respectively. The consequence for coverage was tested by the expanded re-run of Section S12 and by the citation tracking of Section S15, and is stated as a limitation in Sections 2.3 and 4.5 of the main text.

3. Publication types. Eligibility was restricted to peer reviewed journal articles. The registration admitted conference papers reporting primary data.

4. Analysis plan. The registration specified twelve directional and non directional hypotheses with an accompanying inferential analysis plan, including chi square tests, count regression, logistic regression with false discovery rate control, and random effects meta analysis of reported impact values. The narrowing of scope described under amendment 1 removed the fields required to test several of these hypotheses, in particular those concerning life cycle assessment system boundaries, allocation methods and functional units, none of which were extracted from the AI and ML corpus. The synthesis reported here is descriptive and narrative, following the Synthesis Without Meta analysis guideline, rather than inferential.

5. Principal finding. The decision support gap that organizes this review was not among the registered hypotheses. It emerged during cross tabulation of the extracted decision support focus field against canonical application domain, and it is reported as an exploratory finding. The registered protocol provided that additional analyses arising from observed patterns would be labeled as exploratory and used to generate hypotheses for future work, and that provision is applied here. The operational definition of delivered decision support (action-oriented, model-based decision support; main text Section 2.7) is a functional coding applied to the full text of every study under a single scope covering farm, process, and policy decisions; it gives 26 studies with 4 unclear (Table S16). The definition was fixed after data extraction and is post hoc; this is recorded in Table S13.

6. Eligibility threshold (24 October 2026). During screening, goat studies had been admitted under a lower sustainability threshold than other species because of the research program's focus on goats. On 24 October 2026 that exception was withdrawn and the registered eligibility criteria were applied uniformly to every species. Re-examination under criterion E3 (no environmental, resource efficiency or integrated sustainability outcome measured, predicted or assessed) excluded the three studies that had been admitted under the exception (Temenos et al., 2024; Guo et al., 2023; Zhang et al., 2022b) and one mixed species study whose admission route had been undetermined (Navon et al., 2013; re-read in full on 24 October 2026, it reports only identification and false positive rates for jaw movements). After this amendment the corpus was 166 studies (165 after amendment 9); Table 1 of the main text records 81 E3 exclusions and 143 full text exclusions in total. The four records are archived with reasons in the analysis workbook.

7. Sensitivity definitions (24 October 2026). Two alternative operational definitions of delivered decision support were specified in writing on 24 October 2026, before being applied to any study. Definition B (broader, information-based) requires F1 = Yes and a documented decision-relevant output (B2a: the model's own output or a quantity directly derived from it; B2b: in a form the named decision maker could act on for the F1 decision at its spatial and temporal unit; B2c: an explicit statement in Methods, Results or Discussion of how the output feeds that decision). Definition C (stricter, implemented) requires the primary criteria plus F4 = Yes or F5 = Yes. The candidate set for Definition B was fixed in advance as the seven studies with F1 = Yes and primary status other than Yes; each was coded from its full text with B2a to B2c recorded separately before the status, and the evidence quotes are held in the analysis workbook (DS Functional Coding, columns AM to AQ). Results: Definition C, 3 of 165 (1.8%); primary definition, 26 (15.8%); Definition B, 29 (17.6%), or 30 (18.2%) with the one Unclear candidate counted as Yes (Table S22).

8. Quality assessment and transportability (25 October 2026). The composite quality rating was calculated from the three dimensions that share a three-level scale (data quality, model transparency and reproducibility). The external validity label, which has only two levels, and the overall confidence rating, which is itself a summary of the other ratings, are not part of the composite and are reported descriptively (Table S7). Written signalling questions for the three dimensions and coding rules for a new item, the transportability target stated by the authors, were written on 25 October 2026 before any second rating or coding; they are reproduced in the notes to Tables S7 and S23. A second reviewer rated a simple random sample of 40 studies (seed 2026) on the three dimensions from the full texts, blind to the first rating; inter-rater agreement is reported in Table S7. The stated transportability target was coded for all 165 studies from the

abstract, discussion or conclusion and the stated limitations, and is cross-tabulated with the scope of the data and with tests outside the training context in Tables S23 and S24; a second reviewer coded the same 40-study sample blind, and that agreement is given in the note to Table S23.

9. Scope boundary for criterion E2 (25 October 2026). A written rule for the livestock as subject criterion was adopted on 25 October 2026, before any study was checked against it. A study is in scope when the quantity modelled with the AI or ML method, or the decision that model supports, concerns live animals or herds, the farm or grazing system that keeps them, manure, slurry or litter at any stage of treatment or use, or inventories and maps of livestock populations and their emissions. A study is out of scope when both the model and the decision concern only a stage after the animal has become a food product (slaughter, meat or dairy processing, packing, retail or consumption); a study that covers farms together with processing sites stays in scope when the farm stage is part of what is modelled or decided. Every included study was audited against the rule from its title, abstract, conclusion and extraction fields (production stage, demonstration scale, application domain and decision level), with a keyword screen for slaughter, abattoir, processing plant, processor, packing and retail; the audit is available from the authors on request. One study failed the rule: López-Andrés et al. (2018; S77), in which a neural network process model and a genetic algorithm choose the product mix, fuel and refrigerant of a poultry processing plant, with the farm stage entering only as life cycle inventory input. It was excluded under code E2, so Table 1 of the main text records 29 E2 exclusions and 143 full text exclusions, and the corpus is 165 studies. Two studies that model farms together with processing sites (Bi et al., 2024b; Jobarteh et al., 2025) stay in scope because the farm stage is modelled. Identifier S77 is vacant in Table S15 and in the S8 list.

10. Rule for AI or ML as a core method (26 October 2026). A written rule for criterion E1 was adopted on 26 October 2026 and applied to every included study. A study counts as applying AI or ML when its core method passes three tests: it learns from data (an algorithm fits the model's parameters or structure from observations; values set by hand, by expert rules or by physical equations alone do not count); it produces outputs for cases beyond those it was fitted on (predicting, classifying, clustering, detecting or choosing actions); and it is central to the study. Edge cases: optimization algorithms on their own (particle swarm, genetic algorithms, ant colony, linear programming) do not count, and count only when used to fit or tune a model that passes the first two tests, the study then being classified by that model; statistical models (linear or logistic regression, partial least squares, mixed models) count only when built and judged as predictive models with held-out validation (train and test split, cross validation or external data); fuzzy systems count when their membership functions or rules are learned or tuned from data (ANFIS, neuro-fuzzy), and a purely expert-built fuzzy system does not; mechanistic or process models do not count even when calibrated, and give a Hybrid study only when coupled with a model that passes the first two tests; there is no Other category, so every included study falls into one of the five method categories or is excluded under E1. Outcome: 137 of the 165 studies pass on their primary method alone (random forests, neural networks, boosting, support vector machines, convolutional and recurrent networks, ANFIS); the 28 whose primary method is a regression, a clustering, a fuzzy system, an optimizer or a method coded Other were examined one by one. None was excluded. Two were reclassified from Other to Traditional ML: Peng et al. (2019, S108), a projection model fitted by particle swarm optimization and tested on held-out years, and Martinsson et al. (2024, S82), matrix completion predicting counterfactual outcomes. These two and Guarnido-Lopez et al. (2023, S44), changepoint detection with mixed-effects

growth curves judged on a train and test split, pass only through the clause on statistical models and are the weakest inclusions. Rico-Contreras et al. (2017, S114) keeps the Fuzzy Logic category because its expert-built fuzzy system is run alongside a neural network that passes the tests. The ML Category column of the analysis workbook carries the two reclassifications with cleaning log entries.

Correction at reconciliation. One record admitted at screening (Aryee et al., 2024) was identified at reconciliation as a bioRxiv preprint without a journal version and was excluded under the journal only scope of the protocol (exclusion code E7). The corpus was 170 studies at that stage; after amendments 6 and 9 it is 165 studies. This is an application of the registered eligibility criteria rather than an amendment to them.

S3. PRISMA 2020 checklist

A completed PRISMA 2020 27 item checklist accompanies this submission as a separate file, mapping each item to the section of the main text where it is addressed. The PRISMA flow diagram is reported as Fig. 1 of the main text.

S4. Supporting distributions and cross tabulations

The tables in this section give the fine grained distributions and cross tabulations that are summarized in the main text. They are placed here so that the main text can remain focused on the decision support argument.

Table S1. Publication year distribution (n = 165; 2026 is a partial year).

Year	n	Cumulative	% of total
2014	3	3	1.8
2015	1	4	0.6
2016	4	8	2.4
2017	1	9	0.6
2018	3	12	1.8
2019	7	19	4.2
2020	7	26	4.2
2021	6	32	3.6
2022	19	51	11.5
2023	26	77	15.8
2024	39	116	23.6
2025	48	164	29.1
2026	1	165	0.6
**Total**	**165**		**100**

Note. Temporal figures in the main text exclude the single 2026 indexed study, giving a temporal denominator of 164.

Table S2. *Machine learning category by publication period (n = 165).*

Period	Traditional ML	Ensemble	Hybrid	Deep Learning	Fuzzy Logic	Other	Total
2014 to 2018	7 (58%)	0 (0%)	3 (25%)	1 (8%)	1 (8%)	0 (0%)	12
2019 to 2021	10 (50%)	5 (25%)	3 (15%)	2 (10%)	0 (0%)	0 (0%)	20
2022 to 2023	17 (38%)	9 (20%)	10 (22%)	8 (18%)	1 (2%)	0 (0%)	45
2024 to 2026	33 (38%)	20 (23%)	17 (19%)	17 (19%)	1 (1%)	0 (0%)	88

Note. The share of traditional ML declined from 58% in the first period to 38% in each of the two most recent periods, and deep learning rose from 8% to 18% and 19%. Ensemble methods are absent before 2019. The Other column is empty: under the AI or ML rule (Section S2, amendment 10) every study falls into one of the five method categories.

Table S3. *Machine learning category by outcome unit family (n = 165). The Other column is empty after the two reclassifications under the AI or ML rule (Section S2, amendment 10).*

Outcome unit family	Traditional ML	Ensemble	Hybrid	Deep Learning	Fuzzy Logic	Other	Total
Spatial intensity / per area	22	11	6	9	0	0	48
GHG mass / inventory	16	10	10	4	1	0	41
Biogas / energy yield	16	4	9	4	2	0	35
Concentration / pollutant	2	4	5	5	0	0	16
Efficiency / dimensionless ratio	5	2	3	4	0	0	14
Performance metric only	6	3	0	2	0	0	11

Note. Of 33 hybrid studies, 10 (30.3%) report GHG mass or inventory units and 9 (27.3%) report biogas or energy yields.

Table S4. *Hybrid method distribution by canonical application domain (n = 165).*

**Application domain**	**Total studies**	**Hybrid**	**% hybrid**
Manure / Waste to energy	47	12	25.5
Enteric fermentation / methane	23	8	34.8
Grazing/Pasture management	56	6	10.7
Breeding / genetic selection	6	3	50.0
GHG inventory / LCA	8	2	25.0
Livestock building / facility emissions	7	1	14.3
Feed optimization	4	1	25.0
Other	3	0	0.0
Spatial / land use	7	0	0.0
Energy use / efficiency	3	0	0.0
Decision support / planning	1	0	0.0
**Total**	**165**	**33**	**20.0**

Note. Hybrid methods concentrate in enteric fermentation and manure, which together account for 20 of 33 hybrid studies, and are absent from the decision support and planning domain.

Table S5. *Primary species by canonical application domain (n = 165).*

Species	Grazing	Manure	Enteric	GHG inv.	Building	Spatial	Breeding	Feed	Energy	DSS	Other	Total
Cattle	23	15	18	3	4	1	5	3	0	1	0	73
Mixed/Multiple	22	12	0	3	0	5	0	0	0	0	3	45
Pigs	0	9	1	0	3	0	0	1	0	0	0	14
Poultry	0	8	0	0	0	1	0	0	2	0	0	11
Sheep	6	1	2	2	0	0	1	0	0	0	0	12
Goat	2	0	1	0	0	0	0	0	0	0	0	3
Other	3	2	1	0	0	0	0	0	1	0	0	7

Note. Species domain coupling is consistent with housing system. Pigs (9 of 14) and poultry (8 of 11) concentrate in manure and waste to energy; cattle, sheep and goats concentrate in grazing and enteric fermentation.

Table S6. *Sensitivity analysis of the central findings under two exclusions: the 4 studies rated High concern on the composite quality rating (three dimensions, Section 2.6 of the main text) and the 10 studies rated Limited on reproducibility, the dimension with the best inter-rater agreement. Delivered decision support is the functional definition of Section 2.7 of the main text, with Unclear studies shown separately.*

**Quantity**	**Full corpus (n = 165)**	**High concern on the composite excluded (n = 161)**	**Limited reproducibility excluded (n = 155)**
Studies claiming decision support (Yes)	96 (58.2%)	92 (57.1%)	87 (56.1%)
Studies claiming decision support (Yes or Partial)	155 (93.9%)	151 (93.8%)	145 (93.5%)
Functional status Yes (headline)	26 (15.8%)	26 (16.1%)	23 (14.8%)
Functional status Unclear	4 (2.4%)	4 (2.5%)	3 (1.9%)
Yes or Unclear (upper bound)	30 (18.2%)	30 (18.6%)	26 (16.8%)
Explicit claimants not meeting the definition	77 of 96 (80.2%)	73 of 92 (79.3%)	71 of 87 (81.6%)
Inclusive claimants not meeting the definition	129 of 155 (83.2%)	125 of 151 (82.8%)	122 of 145 (84.1%)
Corpus level difference, Yes only claim rate minus headline delivery	42.4 pp	41.0 pp	41.3 pp
Corpus level difference, inclusive claim rate minus headline delivery	78.2 pp	77.6 pp	78.7 pp
Corpus level difference, Yes only claim rate minus upper bound	40.0 pp	38.5 pp	39.4 pp
Corpus level difference, inclusive claim rate minus upper bound	75.8 pp	75.2 pp	76.8 pp
Hybrid methods	33 (20.0%)	33 (20.5%)	31 (20.0%)
Studies sharing code or data	37 (22.4%)	37 (23.0%)	37 (23.9%)
Studies reporting some external validation (flag)	30 (18.2%)	28 (17.4%)	27 (17.4%)

Note. None of the 4 studies rated High concern on the composite (Alejo et al., 2018; Correa-Luna et al., 2024; Dach et al., 2016; Lemphane et al., 2024) delivers decision support, so the headline count is unchanged under that exclusion. 3 of the 26 delivering studies (Altshuler et al., 2025; Elahi et al., 2019; Xie et al., 2022) are rated Limited on reproducibility, so the count falls to 23 under the second exclusion, and 1 Unclear study is also excluded. The share of explicit claimants not meeting the definition changes by about one percentage point or less (80.2% against 79.3% and 81.6%), and the gap remains in 10 of 11 application domains under both exclusions. Differences are computed from proportions before rounding.

Table S7. *Quality dimensions, composite quality rating and inter-rater agreement across the corpus (n = 165; agreement on a random sample of 40 studies).*

Dimension	Most favorable	n (%)	Intermediate	n (%)	Least favorable	n (%)
Data quality	High	58 (35.2)	Medium	96 (58.2)	Low	11 (6.7)
Model transparency	Full details	108 (65.5)	Partial	54 (32.7)	Black box	3 (1.8)
Reproducibility	Code and data shared	37 (22.4)	Methods clear	118 (71.5)	Limited	10 (6.1)
Composite quality rating, revised (three dimensions)	Low concern	20 (12.1)	Some concerns	141 (85.5)	High concern	4 (2.4)
Composite rating, first version (five dimensions; superseded)	Low concern	6 (3.6)	Some concerns	148 (89.7)	High concern	11 (6.7)
External validity label (first version; descriptive only)	Broad applicability	9 (5.5)	Context-specific	156 (94.5)	No third level	–
Overall confidence (descriptive only)	High	45 (27.3)	Medium	109 (66.1)	Low	11 (6.7)

Table S7 (continued). *Inter-rater agreement between the first rating and a second, blind rating of 40 randomly sampled studies.*

Item	Percent agreement	Cohen's kappa (unweighted)	Cohen's kappa (linear weights)	Second rater more favorable, n	Second rater less favorable, n
Data quality	37.5	0.04	0.00	20	5
Model transparency	67.5	0.12	0.10	6	7
Reproducibility	90.0	0.70	0.70	3	1
Composite quality rating, revised	87.5	0.55	0.55	3	2

Note. Signalling questions, written on 25 October 2026 before the second rating. Data quality: Q1 sample size reported; Q2 data source and collection described; Q3 data handling (preprocessing, quality control, missing data or outliers) described; High if all three are met, Medium if Q1 and one other, Low otherwise. Model transparency: Q1 model type and structure specified; Q2 hyperparameter values or the tuning procedure reported; Q3 training procedure (split or resampling, software, stopping rule) reported; Full details if all three, Partial if one or two, Black box if none. Reproducibility: Q1 code or data shared publicly; Q2 methods described well enough to re-implement (transparency Full or Partial and data source described); Code and data shared if Q1, Methods clear if only Q2, Limited otherwise. The composite is Low concern if all three dimensions are at the most favorable level, High concern if two or more are at the least favorable level, Some concerns otherwise. The second rating covered a simple random sample of 40 studies (seed 2026), rated from the full text without sight of the first rating; the analyses use the first rating, and both ratings with all disagreements are held by the authors and available on

request. The external validity label used only two levels, so its least favorable count was zero by construction; it and the overall confidence rating are no longer part of the composite and are shown for the record.

Table S8. *Code and data sharing by canonical application domain (n = 165).*

**Application domain**	**Total**	**Code or data shared**	**% shared**
Breeding / genetic selection	6	3	50.0
Enteric fermentation / methane	23	8	34.8
Other	3	1	33.3
Livestock building / facility emissions	7	2	28.6
Spatial / land use	7	2	28.6
Grazing/Pasture management	56	15	26.8
Feed optimization	4	1	25.0
GHG inventory / LCA	8	1	12.5
Manure / Waste to energy	47	4	8.5
Energy use / efficiency	3	0	0.0
Decision support / planning	1	0	0.0
**Total**	**165**	**37**	**22.4**

Note. Sharing rates vary more than fourfold across domains. This review did not assess the reasons for the difference.

Table S9. *Decision support claim and practical readiness by measurement level (n = 165).*

**Measurement level**	**Total**	**Claim Yes**	**% Yes**	**Laboratory only**	**% lab**	**Farm-applicable**	**Deployed**
National	5	5	100.0	2	40.0	3	0
Regional	50	35	70.0	15	30.0	33	2
Farm	57	35	61.4	17	29.8	35	5
Multiple	11	5	45.5	4	36.4	5	2
Herd	2	1	50.0	1	50.0	1	0
Animal	38	15	39.5	28	73.7	9	1
Not specified	2	0	0.0	2	100.0	0	0

Note. Explicit claim rates rise with the scale at which outcomes are reported, from 39.5% at animal level to 70.0% at regional level and 100% at national level, while deployment moves in the opposite direction. Of 50 regional studies, 2 are coded as deployed. The one study tested on a farm without being deployed (Lei et al., 2020; regional) is counted as farm applicable here and in Figure 10 of the main text.

Table S10. *Practical readiness by outcome unit family (n = 165).*

**Outcome unit family**	**Total**	**Laboratory only**	**% lab**	**Farm-applicable**	**Deployed**
Spatial intensity / per area	48	9	18.8	32	7

GHG mass / inventory	41	19	46.3	21	1
Biogas / energy yield	35	19	54.3	14	2
Concentration / pollutant	16	8	50.0	8	0
Efficiency / dimensionless ratio	14	9	64.3	5	0
Performance metric only	11	5	45.5	6	0

Note. Dimensionless efficiency ratios are the least likely to be deployed, whereas spatial and per area intensities are the most likely. Physical mass quantities occupy an intermediate position. The one study tested on a farm without being deployed (Lei et al., 2020; spatial intensity) is counted as farm applicable.

S5. Data extraction fields and canonical value sets

Each included study was read in full and the following fields were extracted into a standardized template. Extraction was performed by five reviewers who divided the studies between them and worked from a shared written guide; each study was extracted by one reviewer and no study was extracted twice. One author then checked every extracted value against the written cleaning rules and recorded each change in the cleaning log (Section S13). The fields coded a second time are listed in Section 2.5 of the main text (functional flags and decision support claim, Section S14; input structure, Section S13; three quality dimensions and the stated transportability target on 40 studies, Tables S7 and S23); the other fields were coded once. Where a value could not be located in a study it was recorded as not reported rather than inferred.

Table S11*. Data extraction fields.*

Column	Field	Definition
A	Study ID	Unique identifier (e.g., Smith_2023_001)
B	First Author	Last name of first author
C	Year	Publication year (2010-2026)
D	Title	Full paper title
E	Journal	Journal name
F	DOI	Digital object identifier
G	Country/Region	Where study was conducted
H	Study Type	Experimental/Observational/Modeling/Hybrid
I	Data Source	Farm/Experimental station/Database/Sensor/Remote sensing

Column	Field	Definition
J	Sample Size	Number of animals, farms, or observations
K	Time Period	Data collection period
L	Geographic Scope	Single farm/Multi-farm/Regional/National/International
M	Primary Species	Main livestock species studied
N	Secondary Species	Additional species if multi-species study
O	Breed	Specific breed if mentioned
P	Production System	Intensive/Extensive/Pasture-based/Mixed/Not specified
Q	Production Stage	Growing/Lactating/Breeding/All stages/Not specified
R	ML Category	Traditional ML/Deep Learning/Ensemble/Hybrid/Fuzzy Logic
S	Primary Algorithm	Main ML algorithm used
T	Secondary Algorithms	Additional algorithms if compared
U	Model Purpose	Prediction/Classification/Optimization/Decision support
V	Input Variables	List main predictor variables
W	Validation Method	Train-test split/Cross-validation/External/None
X	Performance Metrics	R^2, RMSE, MAE, Accuracy, F1, etc.
Y	Best Model Performance	Reported values for primary metrics
Z	Primary Sustainability Category	GHG/Resource efficiency/Land-ecosystem/Water/Energy/Integrated
AA	Specific Outcome	e.g., Enteric methane, Feed efficiency, Ammonia
AB	Secondary Outcomes	Additional sustainability outcomes
AC	Measurement Level	Animal/Herd/Farm/Regional/National
AD	Outcome Unit	Units of measurement (g CH_4/day, kg CO_2eq, %, etc.)
AE	Application Domain	Specific area of application
AF	Decision Support Focus	Yes/No/Partial
AG	Practical Implementation	Lab-research only/Farm-applicable/Deployed
AH	Main Finding	2-3 sentence summary of key result
AI	Novelty/Contribution	What's new or significant
AJ	Limitations Noted	Self-reported study limitations
AK	Data Quality	High/Medium/Low/Not assessable
AL	Model Transparency	Full details/Partial/Black box
AM	Reproducibility	Code-data available/Methods clear/Limited
AN	External Validity	Broad applicability/Context-specific (kept for the record; not used in the composite, see Table S7)
AO	Overall Confidence	High/Medium/Low
AP	Goat Study?	Yes/No
AQ	Methods Transferable to Goats?	High/Medium/Low/N/A. Rated by the extractor of each study (one rater per study) in answer to the question "Could the ML methods or findings be applied to goat systems?", with no further written rule; not used in any result of this review
AR	Notes for Your Model	Insights for Insight Maker/RL work

Column	Field	Definition
AS	Extraction Date	Date of data extraction
AT	Extractor Notes	Any additional notes
AU	Extractor	Free text
AV	Goat Present?	Yes if goats appear anywhere in the study (primary or secondary species or system inclusion); No otherwise. Distinct from AP “Goat Study?” which captures only primary-focus goat papers.

Note. Field AQ (Methods Transferable to Goats?) is the judgement of the extractor of each study on a three-level scale, with no written rule beyond the question in the extraction guide; it is not used in any analysis reported in this review. Fields AR to AU record notes internal to the wider research program and were not used in any analysis reported in this review. Column I (Data Source) remains uncanonicalized free text and was not used to produce any mutually exclusive distribution; it is used only within the input structure classification described below, which is coded by hand (Section S13).

Free text entries were mapped onto canonical value sets so that consistent counting was possible. Every mapping decision was recorded in an auditable cleaning log held in the analysis workbook. The canonical sets are as follows.

Table S12. *Canonical value sets applied to extracted free text fields.*

Variable	Source column	Canonical values	n categories
Species canonical	M	Cattle; Mixed/Multiple; Pigs; Poultry; Sheep; Goat; Other	7
Country canonical	G	First country listed, parenthetical detail removed, English exonyms standardized	50 observed: 43 countries and 7 regional or multi-country labels (Section S13)
Geographic scope	L	Single farm; Multi-farm; Regional; National; International; Not specified	6
ML category	R	Traditional ML; Deep Learning; Ensemble; Hybrid; Fuzzy Logic	5
Algorithm family	S, T	Random Forest; Shallow Neural Networks; Gradient Boosting / Tree Ensembles; Deep Learning - CNN; Deep Learning - LSTM/RNN; Linear/Logistic/Kernel Regression; SVM/SVR; Fuzzy Logic; Other	9
Model purpose canonical	U	Prediction; Classification; Optimization; Decision support; Multiple	5
Validation method	W	Train-test split; Cross-validation; External validation; Multiple; None reported	5
Application domain canonical	AE	Grazing/Pasture management; Manure / Waste-to-energy; Enteric fermentation / methane; GHG inventory / LCA; Livestock building / facility emissions; Spatial / land use; Breeding / genetic selection; Feed optimization; Energy use / efficiency; Decision support / planning; Other	11
Outcome unit family	AD	Spatial intensity / per-area; GHG mass / inventory; Biogas / energy yield; Concentration / pollutant; Efficiency / dimensionless ratio; Performance metric only; Other	7
Measurement level	AC	Animal; Herd; Farm; Regional; National; Multiple; Not specified	7
Sample size bin	J	Small; Medium; Large; Very large	4
Composite quality rating	AK to AO	Low; Some concerns; High, derived by rule from the three quality dimensions	3
Goat relevance category	M, AV	Primary goat study; Mixed-species goat-included study; Non-goat study	3
Input structure	I, V, S	Tabular measurements; Imagery or spectral; Sequential or temporal; Imagery and temporal, coded by hand under the definitions of Section S13	4

Note. The input structure variable used in Fig. 4 of the main text and Supplementary Table S21 was coded by hand for every study from the Input Variables, Data Source and Primary Algorithm fields, under the written definitions of Section S13. The hand coding (one row per study) and the highest reported R squared per study used in Supplementary Figure S8 are in the analysis workbook (sheet Input Structure and R2 Map).

Two operational definitions carry the central analysis of the main text and are restated here. A claim of decision support is taken from column AF, which records whether a study frames its outputs as supporting producer, process, or policy decisions, coded Yes, Partial or No; this records author framing and involves no independent adjudication, and a second coding of the field from the full text is reported in Section S14 (Tables S39 and S40). Delivered decision support, defined as action-oriented, model-based decision support (main text Section 2.7), is coded independently of the claim from six functional flags applied to the full text of every study, F1 names a decision, F2 compares alternatives, F3 recommends or controls, F4 accessible tool, F5 evaluated in use, and F6 outcome measured, recorded for every study in the DS Functional Coding sheet of the workbook with an evidence note (definitions and derivation rules in Table S16). The scope covers farm, process, and policy or program decisions, and the scale of demonstration is recorded but is not a criterion. A study's functional status is Yes when F1 is Yes and F2 or F3 is Yes, No when F1 is No or both F2 and F3 are No, and Unclear otherwise: 26 studies (15.8%) are Yes, 4 are Unclear, and counting the Unclear studies as Yes gives the upper bound of 30 (18.2%). In Table S15 an asterisk marks the 26 studies with status Yes and a double dagger marks the 4 Unclear studies.

One goat study (Wang et al., 2023, Wang_2023_002) is coded with an enteric fermentation application domain and an integrated sustainability category although its primary outcome is growth and lactation performance predicted from the rumen microbiome. The classification was reviewed and retained because the study measured methane production in vitro with rumen fluid from high and low gain goats, with high gain goats producing less methane, which is an enteric fermentation outcome; the review decision and rationale are recorded in the workbook cleaning

log. Because that measured methane outcome satisfies the eligibility criteria, the study remains in the corpus under amendment 6 (Section S2).

S6. Supplementary figures

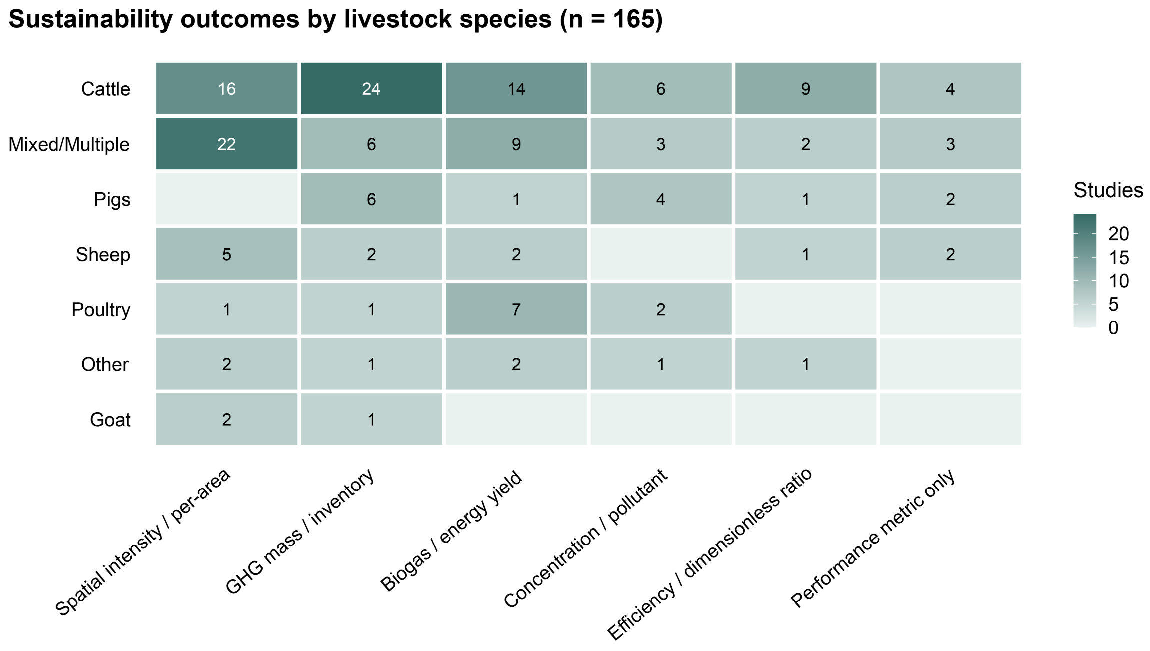

Figure S1. *Sustainability outcome unit families by primary livestock species (n = 165).*

Note. All other figures generated in the analysis are reported in the main text. The five multi panel figures in the main text (Figs. 2, 3, 5, 6 and 11) are composites; their component panels are generated individually at full resolution by the R code deposited with the analysis materials, which includes a mapping of every output file to its figure number.

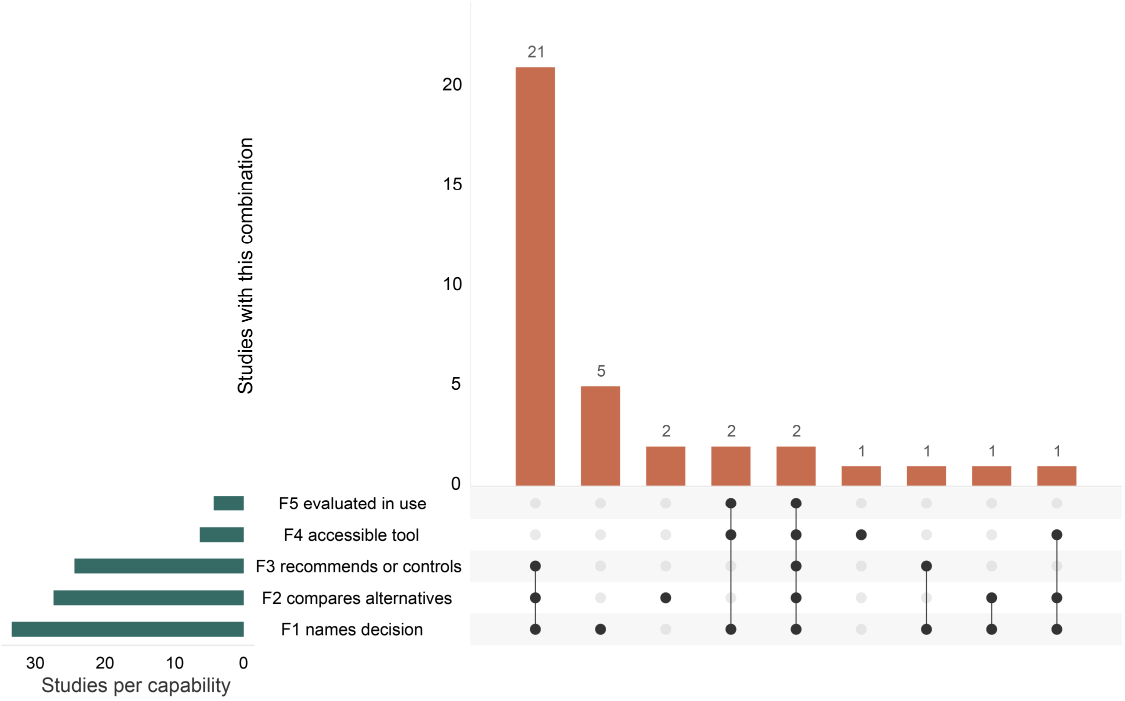

Figure S2. *Decision support capability flags F1 to F5 and their intersections across the 165 included studies. Horizontal bars give the number of studies holding each flag (F1 names a decision 33, F2 compares alternatives 27, F3 recommends or controls 24, F4 accessible tool 6, F5 evaluated in use 4); vertical bars give the number of studies holding each exact combination. The 129 studies holding no flag are omitted.*

Note. Studies holding exactly one, two, three, four and five flags number 8, 2, 24, 0 and 2 respectively. Flags coded Unclear are counted as No in this figure. The figure was drawn with the UpSetR package; a ggplot2 companion (capability counts and co occurrence heatmap) is deposited with the analysis materials.

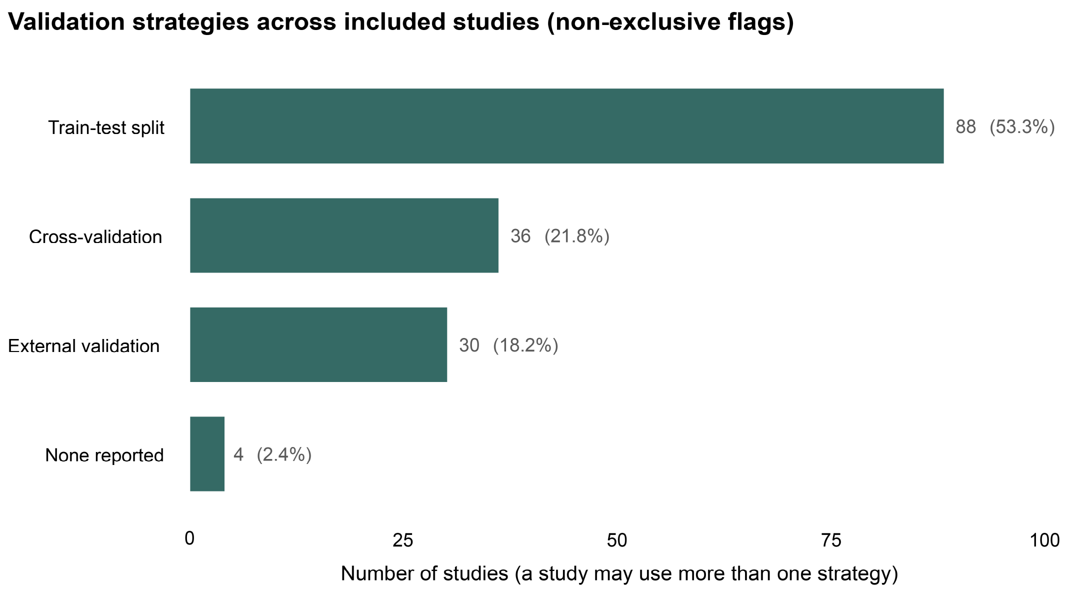

Figure S3. Validation strategies across the 165 included studies counted as non exclusive flags. A study may use more than one strategy, so the bars do not sum to 165. Train test split 88 (53.3%), cross validation 36 (21.8%), some form of external validation 30 (18.2%), none reported 4 (2.4%).

Note. Of the 30 studies flagged, 9 report validation on an independent farm, herd, region or trial, 15 against an independent dataset, and 6 on a later time period; grouped cross validation that leaves out a farm, herd or treatment is counted under cross validation. Derivation rules are given in Table S16.

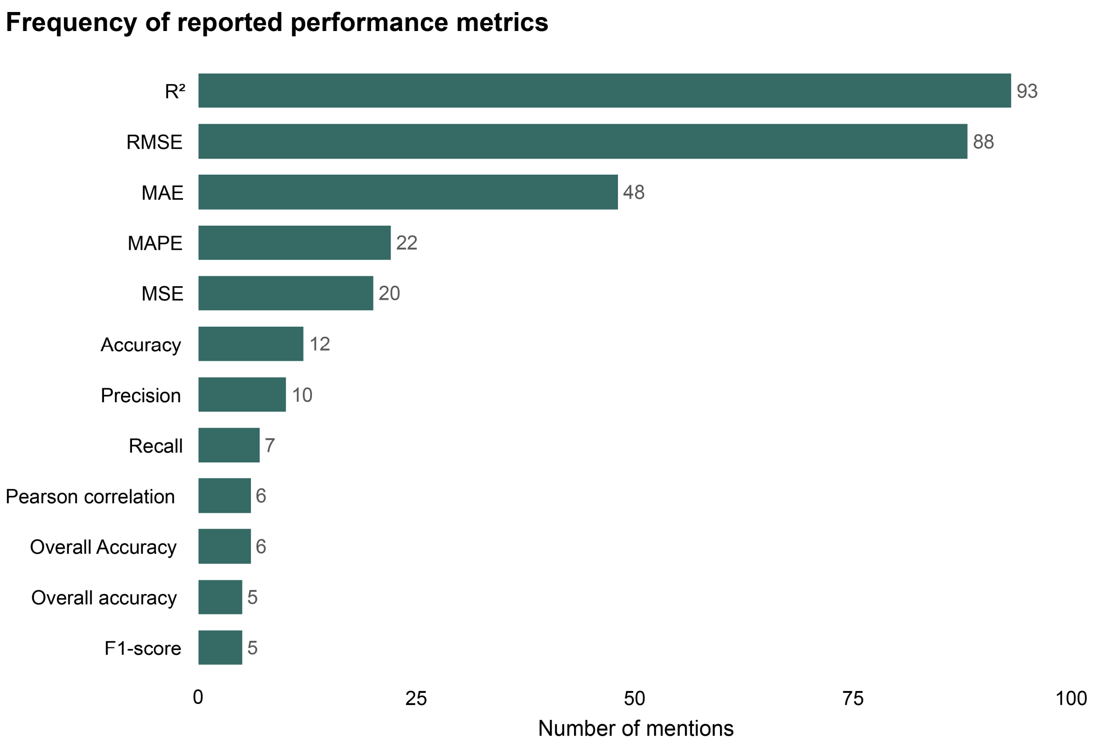

Figure S4. *Frequency with which each performance metric is reported across the 165 included studies. Counts exceed the corpus total because most studies report more than one metric.*

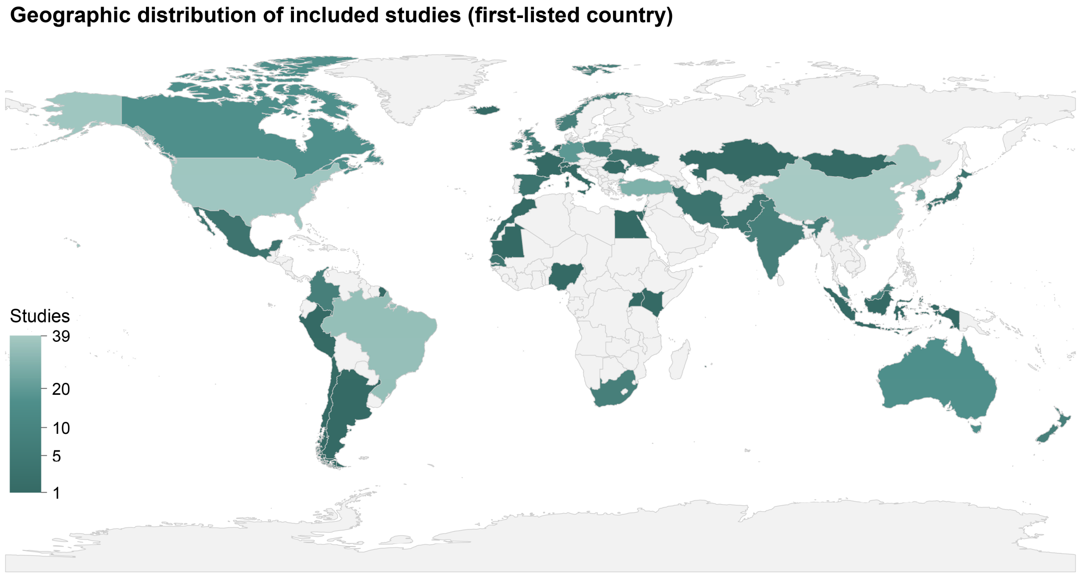

Figure S5. *Geographic distribution of included studies by first-listed country of study; studies naming more than one country are counted under the first (Table S34).*

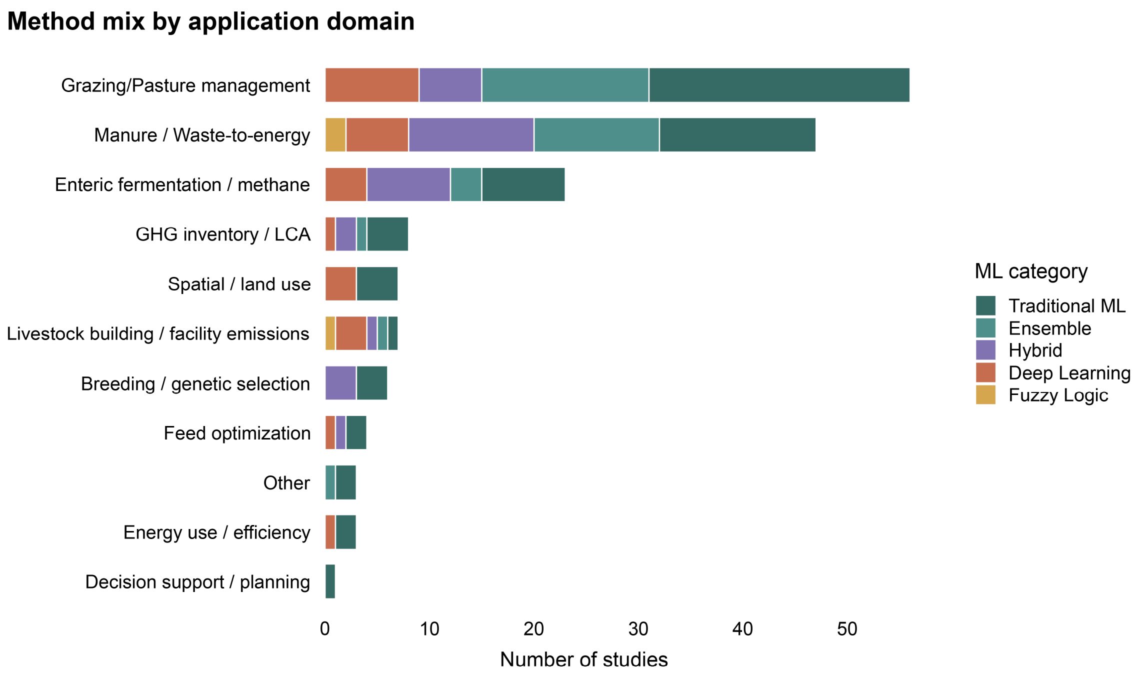

Figure S6. *Machine learning category by canonical application domain.*

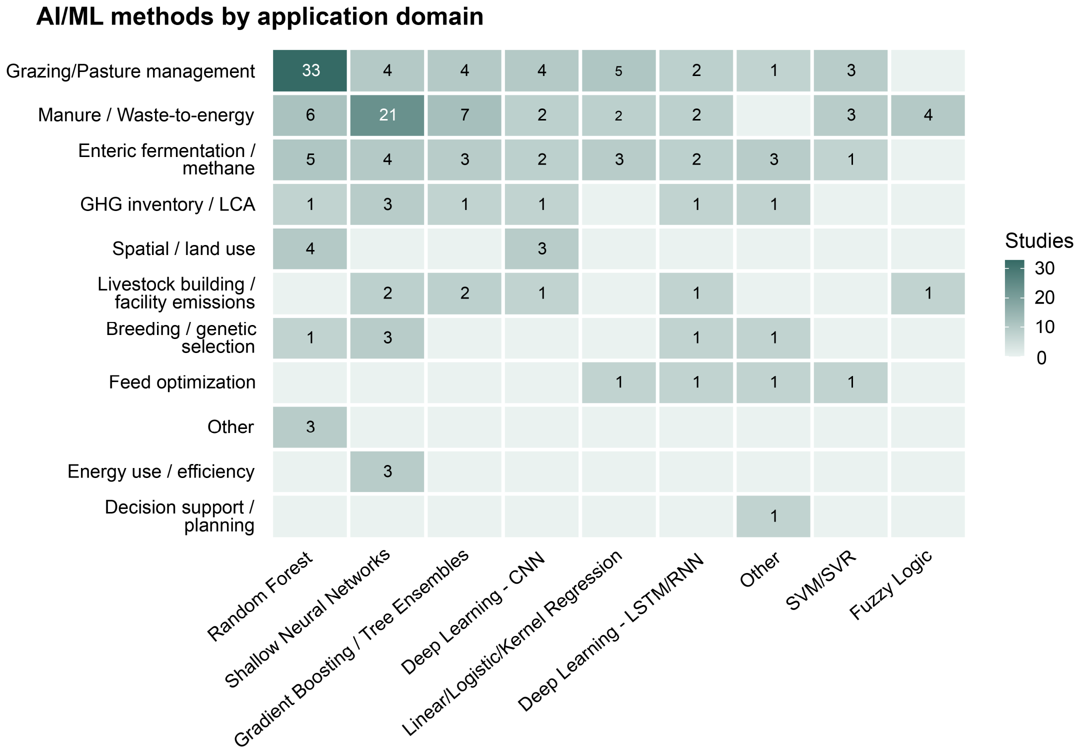

Figure S7. *Algorithm families by canonical application domain.*

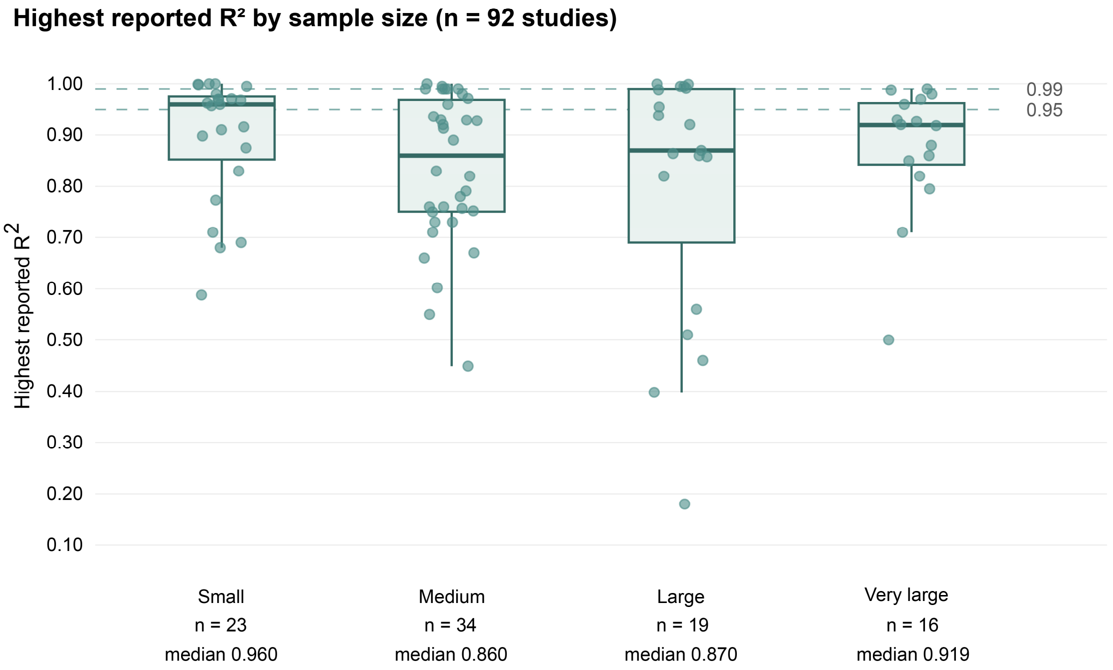

Figure S8. *Highest reported R2 by sample size bin (n = 92 studies from which an R2 could be extracted). Median reported R2 is 0.960 in the smallest sample size bin against 0.860 to 0.919 in the larger bins, and 57% of small sample studies report R2 of at least 0.95. The figure is a descriptive audit of reporting, not evidence of an inverse relationship: the highest value per study is taken across different targets and models, the sample size field counts animals, farms, plots, images, experimental runs or years, and across the 92 studies the rank correlation between the highest reported value and the extracted sample size is weak (Spearman rho = −0.12, p = 0.25).*

Table S13. Deviations from the registered protocol (OSF ysw5q).

Registered	Executed	Reason
Meta analysis with pooled effect estimates	Narrative synthesis following SWiM, with no pooling (item by item mapping in Table S25)	The corpus proved too heterogeneous in species, algorithm, functional unit and outcome for pooling to be defensible
Twelve hypotheses (H1 to H12) with an inferential analysis plan	Outcomes reported for the six hypotheses testable without pooling (H1, H2, H3, H7, H8, H11), with partial results for H4 and H9; H5, H6, H10 and H12 were not tested. See Table S14	H5, H6, H10 and H12 depend on life cycle assessment fields (system boundary, allocation method, impact values) and on funding source, none of which were carried into the extraction schema when the scope narrowed to AI and ML
Three literatures joined by OR: farm system modeling, life cycle assessment, and artificial intelligence or optimization	AI or ML required; farm system models and life cycle assessments retained only where combined with AI or ML	The farm system modeling and life cycle assessment streams are reported separately, keeping the present synthesis within the scope of a single article
Scopus, Web of Science, AGRICOLA and CAB Abstracts, with Google Scholar as a supplementary source	Web of Science, CAB Abstracts, IEEE Xplore and PubMed	Scopus was not accessible to the team; AGRICOLA was not searched because its coverage was judged to be largely duplicated by CAB Abstracts (untested); Google Scholar returned several hundred thousand records that could not be exported or screened reproducibly; IEEE Xplore and PubMed were added to reach the engineering and biomedical literature. Coverage tested in Sections S12 and S15.
Peer reviewed journal articles in English, with conference papers reporting primary data admitted	Peer reviewed journal articles only. Conference papers retrievable from IEEE Xplore at the search stage were excluded during screening	
300 to 400 records anticipated, 80 to 120 studies included	2,664 records imported, 1,994 unique records screened, 165 studies included (see the eligibility threshold and scope rows below)	The search yield exceeded the scoping estimate; the registration stated that the final number was not predetermined
No preregistered analysis of decision support delivery	The gap between claimed and delivered decision support is the organizing analysis of the review. Delivered decision support is a functional coding applied to every full text under one scope (farm, process, and policy decisions), with the requirement that the model itself evaluate or recommend the alternatives, giving 26 studies with status Yes and 4 Unclear (Table S16)	Not preregistered. This focus emerged during extraction and is reported as exploratory; the functional definition is post hoc and was fixed after data extraction

Registered	Executed	Reason
Goat studies admitted under a lower sustainability threshold with no preregistered sensitivity analysis	The exception was withdrawn on 24 October 2026 and the four affected studies (three admitted under the rule and one of undetermined route) were excluded under E3; the Admitted_via_goat_rule flag is retained in the workbook for audit only (Section S2, amendment 6)	A systematic review should apply the same outcome threshold to every species
No alternative definitions of delivered decision support	Two sensitivity definitions (B, broader; C, stricter) specified in writing on 24 October 2026 before coding and then applied (Table S22)	See Section S2, amendment 7
Exclusive canonical categories for validation method, model purpose and method family	Non exclusive capability flags (VAL_*, PURP_*, HYBRID_mechanistic_coupling) added alongside the unchanged canonical columns (Table S16)	Collapsing compound entries to Multiple undercounted capabilities such as external validation (2 canonical against 30 flagged) and optimization (7 against 17)
Peer reviewed journal articles only	One record admitted at screening (Aryee et al., 2024) was identified at reconciliation as a preprint and excluded under code E7; after amendments 6 and 9 the corpus is 165 studies	Application of the registered eligibility criteria at reconciliation, not an amendment; PRISMA counts and every table were recomputed
Reports not retrieved initially counted among full text exclusions	The 2 reports that could not be retrieved are reported separately from the 308 reports assessed (Fig. 1 of the main text)	Alignment with the PRISMA 2020 flow diagram
Five quality dimensions, including a two-level external validity label and an overall confidence rating, collapsed into one composite	Composite recalculated from the three three-level dimensions; external validity replaced by three items (scope of the data, test outside the training context, stated transportability target) with a duplicate rating on a 40-study sample (Tables S7, S23 and S24)	See Section S2, amendment 8
Livestock as the study subject (criterion E2), with no written rule for studies set after the farm gate	A farm gate scope rule for E2 was written on 25 October 2026 and every included study was audited against it; one poultry processing plant study (López-Andrés et al., 2018) was excluded under E2 and every count was recomputed for 165 studies	See Section S2, amendment 9

Table S14. Outcomes for the registered hypotheses (OSF ysw5q).

Hypothesis	Registered prediction	Outcome	Result
H1	Cattle studies more numerous than goats, sheep, pigs or poultry	Cattle 73/165 (44.2 percent, CI 36.9 to 51.9); goats 3 (1.8), sheep 12 (7.3), pigs 14 (8.5), poultry 11 (6.7). Chi square 143.6, df 4, $p < 0.001$	Supported
H2	More studies from the Global North than the Global South	Global South 98/163 (60.1 percent, CI 52.5 to 67.3); Global North 65/163 (39.9 percent). China contributes 39 studies	Not supported, direction reversed
H3	Positive trend in study numbers over time	3.45 studies per year, $r = 0.854$, $p < 0.001$, 2014 to 2025; Spearman rho 0.930	Supported
H4	Environmental metrics reported more often than economic, and economic more often than social	Environmental primary categories 130/165 (78.8 percent). The extraction schema has no separate economic or social category, so the full ordering could not be tested	Partially testable
H5	Cradle to gate boundaries more common than other boundary types	System boundary was not an extracted field	Not testable
H6	GHG intensities per functional unit differ across species	Quantitative impact values per functional unit were not extracted	Not testable
H7	Studies reporting validation or uncertainty analysis less frequent than those that do not	Any validation reported 161/165 (97.6 percent, CI 93.9 to 99.1). External validation in the canonical column 2/165 (1.2 percent, CI 0.3 to 4.3); some external validation as a non exclusive flag 30/165 (18.2 percent). Composite quality rating Low concern in 20 of 165 on the composite (6 of 165 on the first version, Table S7)	Not supported, direction reversed
H8	Open data or code available in fewer than 25 percent of studies	Code or data available 37/165 (22.4 percent, CI 16.7 to 29.4). One sided binomial against 0.25, $p = 0.25$	Directionally consistent, not statistically distinguishable from 25 percent
H9	Studies integrating farm system modeling, LCA and AI in fewer than 10 percent	LCA was not an extracted field. The nearest available measure, mechanistic plus ML hybrid study type, is 56/165 (33.9 percent, CI 27.2 to 41.5); coupling of a learned model to a mechanistic, life cycle, or farm system model as a capability flag is 7/165 (4.2 percent)	Not testable as specified
H10	Publicly funded studies report validation and open artifacts more often than industry funded studies	Funding source was not an extracted field	Not testable
H11	Growth in AI use over time stronger in cattle than in underrepresented species	Cattle share 50.0 percent in 2014 to 2020 and 43.2 percent in 2021 to 2026, chi square 0.41, $p = 0.52$. Trend slopes 2014 to 2025: cattle 1.47 studies per year per year, underrepresented	Not supported

		species 0.82	
H12	LCA results differ by allocation method and system boundary	Allocation method and system boundary were not extracted fields	Not testable

Note. N = 165 included studies, publication years 2014 to 2026. Proportions are given with Wilson 95 percent confidence intervals. The 2026 partial year is excluded from the trend estimate in H3. Global North was defined as North America, Europe, Australia, New Zealand, Japan, South Korea and Israel; Global South was all other countries; two records reporting global or international scope were excluded from the H2 denominator. These analyses are reported descriptively rather than as confirmatory tests, so the Benjamini Hochberg procedure specified in the registration was not applied.

S7. Evidence table for all included studies

Table S15. Characteristics of all 165 included studies, one row each**, drawn directly from the analysis workbook. The Study column gives each study's identifier in S8 (Included studies) and links to its full bibliographic entry there. An asterisk marks the 26 studies whose functional decision support status is Yes and a double dagger marks the 4 Unclear studies. DSS records the decision support focus as claimed by the study (Yes, Partial or No). Performance values are the best reported metric for the primary model as extracted; NR indicates not reported. Full bibliographic details are given in S8. **Identifiers S45, S77, S89, S135 and S166 are vacant: four of those records were excluded under criterion E3 (Section S2, amendment 6) and one (S77) under criterion E2 (amendment 9).

Study	Species	Primary algorithm	Application domain	Sustainability outcome	DSS	Best reported performance
S1 Alejo (2018)	Other	SVM (Support Vector Machine with Pearson VII …	Manure/W2E	Prediction of Total Ammonia Nitrogen (TAN) co…	Yes	SVM PUK: $R^2 = 0.898$, MAE = 0.152, MSE = 0.095, $\chi^2 = 1.772$ (vs. ANN: $R^2 = 0.875$, MAE = 0.269; An…
S2 Alemu (2025)	Sheep	Neural Network GBLUP (NN GBLUP), Bayesian mul…	Breeding	Genomic prediction of enteric methane emissio…	Yes	CH4: accuracy 0.30 (train test), 0.27 (5 fold CV) with NN GBLUP (25% PCA) vs 0.09/0.15 for GM b…
S3 Ali (2021)	Mixed/Multiple	Feedforward Backpropagation ANN (FFBP, 6 8 3 …	Manure/W2E	Cumulative CH_4 production from anaerobic dige…	Partial	ANN cow manure: R^2=0.995, RMSE=0.111, MSE=0.0123; ANN sheep manure: R^2=0.991, RMSE=0.164, MSE=0…

Study	Species	Primary algorithm	Application domain	Sustainability outcome	DSS	Best reported performance
S4 Altshuler (2025)*	Cattle	Network based unsupervised clustering (k-mer …	Enteric	Enteric methane (CH_4) reduction prediction	Yes	Spearman Rho = −0.86; Kendall Tau = −0.69; optimized deployment achieved 29% methane reduction …
S5 Ashworth (2024)	Cattle	Neural Network (ANN), 3 layer feedforward (28…	Grazing/pasture	Grazing management optimization for grassland…	Partial	Neural network validation: low validation loss converging with training loss (no overfitting); …
S6 Bao (2025)	Mixed/Multiple	Gradient Boosting Regression (GBR)	GHG inventory	Atmospheric methane concentration (XCH_4) driv…	Partial	GBR: R^2=0.918, RMSE=11.78 ppb, MAPE=0.47% on independent test set
S7 Barczyk (2024)	Cattle	Random Forest	Enteric	Nitrous oxide (N_2O) emissions from grazed pas…	Partial	RF gap filling: R^2=0.96 (training), R^2=0.88 (test); RMSE=0.19 g N_2O N ha^{-1} h^{-1} (training), 0.27…
S8 Bartels (2022)	Cattle	Recurrent Neural Network (RNN, simple Recurre…	Grazing/pasture	Precision livestock farming for environmental…	Partial	Best 32 bit model: accuracy=95.7%, MAE=0.0571 (GRU, 2 layers, 17,720 weights); best quantized m…
S9 Basak (2022a)	Pigs	Ridge Regression (RR; best testing R^2=0.922 f…	Manure/W2E	Daily CH_4 production rate (kg CH_4 pig^{-1} day^{-1}…	Partial	NR
S10 Basak (2022b)	Pigs	Exponential regression	Enteric	CO_2 emission rate from pigs	No	R^2=0.757, RMSE=16.45 ppm pig^{-1} (exponential model, testing stage, feed intake dataset)

Study	Species	Primary algorithm	Application domain	Sustainability outcome	DSS	Best reported performance
S11 Basak (2024)	Pigs	Support Vector Regression (SVR)	Manure/W2E	Ammonia (NH_3) concentration from pigs	Partial	R^2=0.913, RMSE=0.136 ppm pig^{-1} day^{-1}, MAE=0.124 ppm pig^{-1} day^{-1} (SVR, D3 dataset, testing stage…
S12 Bhattad (2025)	Poultry	LSTM (Long Short Term Memory)	Manure/W2E	Diurnal CO_2, CH_4, and NH_3 emission concentrat…	Yes	MSE=35.2; R^2=0.87; average MAE=3.47 ppm; average RMSE=4.12 ppm
S13 Bi (2024a)	Cattle	Random Forest	Breeding	Satellite derived methane (CH_4) column concen…	Partial	R^2 = 0.97; MSE = 51,000 (Random Forest with VIF processed data)
S14 Bi (2024b)	Cattle	LSTM	Enteric	Atmospheric methane (CH_4) concentration bench…	Partial	RMSE = 14.8 (with season and year features)
S15 Boulanger-Lapointe (2022)	Sheep	Ensemble (average of Maxent, Random Forest, T…	Grazing/pasture	Grazing impact; species distribution overlap …	Yes	AUC = 0.9708 (sheep); AUC = 0.8817 (reindeer); AUC = 0.9143 (pink footed goose); AUC = 0.8844 (…
S16 Cao (2016)	Cattle	LS SVM with RBF kernel (prediction set: R^2=0.…	Manure/W2E	Biochar yield from cattle manure pyrolysis; p…	Partial	LS SVM prediction set: R^2=0.9625, AAPRE=4.95%, RMSE=0.3652 g
S17 Cao (2025)	Pigs	Extra Trees Regression (ETR)	Manure/W2E	Odor concentration prediction from pig manure…	Partial	ETR (P all) test set: R^2=0.81, RMSE=1.05; training set: R^2=0.99, RMSE=0.06; PLS coupled model: …

Study	Species	Primary algorithm	Application domain	Sustainability outcome	DSS	Best reported performance
S18 Cardinale (2022)	Cattle	Combinatorial network ML (Synomics Insights)	Enteric	Enteric methane emission	Partial	Synomics Insights J = 0.261 for methane (vs. random J = 0.165); 21/26 taxa differentially enric…
S19 Chasen (2025)*	Other	Random Forest	Grazing/pasture	Pasture yield prediction for sustainable graz…	Yes	R^2=0.99, RMSE=0.11 tons/acre (4% of average yield)
S20 Chen (2025)*	Pigs	EcoSegLite (custom lightweight YOLO11 seg var…	Manure/W2E	Carbon footprint per kg live pig (kg CO_2 eq/k…	Yes	Segmentation: mAP50=96.7%, mAP50 95=82.3%, F1=92.0%, 1.60 M params, 4.0 GFLOPs, 98.2 FPS; Weigh…
S21 Cheng (2025)	Mixed/Multiple	Random Forest	Manure/W2E	Veterinary antibiotic pollution loads from li…	Yes	Livestock distribution: internal cross validation R^2>0.945, external validation R^2>0.652, cross…
S22 Chernysh (2025)	Poultry	Multilayer Perceptron (MLP)	Manure/W2E	Biogas and methane yield from poultry manure …	No	MLP electrofermentation: training MSE=0.02%, test MSE=0%; MLP ultrasound: training MSE=0.02%, t…
S23 Choo (2025)	Cattle	Hybrid Stacked Ensemble (NN meta learner comb…	Enteric	Methane (CH_4) emissions from enteric fermenta…	Partial	Hybrid Stacked Ensemble, Time split: RMSE=877.45, nRMSE=36.6%, MAE=584.79, R^2=0.82; LOOCV: RMSE…
S24 Correa-Luna (2024)	Cattle	Neural Network (ANN)	Grazing/pasture	Carrying capacity	Yes	Control (no training): MAE=498 kg DM/ha (19.1%), RMSE=602 kg DM/ha; 2W training: MAE=342 kg DM/…

Study	Species	Primary algorithm	Application domain	Sustainability outcome	DSS	Best reported performance
S25 Dach (2016)	Mixed/Multiple	MLP (Multilayer Perceptron)	Manure/W2E	Cumulative methane production from bovine and…	Yes	MN1 (bovine): R = 0.999, S.D. Ratio = 0.049 (training); MN2 (porcine): R = 0.986, S.D. Ratio = …
S26 Dai (2023)	Mixed/Multiple	Random Forest	Grazing/pasture	Human appropriation of NPP (HANPP) on grazed …	Yes	RF derived HANPP vs grazing intensity: R=0.704, R^2=0.50, RMSE=41.29 (2010); R=0.667, 0.703 in 2…
S27 Fernández (2023)	Cattle	Random Forest	Grazing/pasture	GHG total emissions (CO_2eq/ha), GHG emission …	Yes	R^2=0.82 (Jaccard coefficient for silage bag classification); silage area significant predictor …
S28 De Rosa (2021)	Cattle	Random Forest	Grazing/pasture	Pasture biomass prediction for sustainable gr…	Yes	RF: NRMSE=16.51%, d=0.90, r^2=0.71 (70:30 split, all variables); GAM spatial: NRMSE=22.9%, d=0.6…
S29 Defalque (2024)	Cattle	XGBoost Regressor (biomass summer), Support V…	Grazing/pasture	Pasture biomass and dry matter estimation for…	Yes	Biomass summer (XGB): Adj R^2=0.761, RMSE=1914.80 kg/ha (18.84%); Dry matter summer (SVR): Adj R…
S30 Dhussa (2014)	Cattle	NARX (Nonlinear Autoregressive Exogenous) neu…	Manure/W2E	Biogas production rate from anaerobic digesti…	Yes	Optimized NARX (5 input, two hidden layers of 10 neurons each, 18 day delay, traincgb algorithm…

Study	Species	Primary algorithm	Application domain	Sustainability outcome	DSS	Best reported performance
S31 Dong (2024)	Mixed/Multiple	Random Forest (RF)	Grazing/pasture	Grassland aboveground biomass estimation for …	Yes	R^2=0.55, RMSE=64.33 g·m⁻²; estimated AGB mean (85.33 g·m⁻²) closely matched measured mean (85.3…
S32 Dong (2014)	Cattle	Backpropagation Neural Network (BPNN; CH_4 bes…	Enteric	In vitro CH_4 production from rumen fermentati…	No	BPNN CH_4 (3 8 1): r^2=0.95, RMSPE%=3.89%; BPNN CO_2 (3 11 1): r^2=0.97, RMSPE%=2.95%; BPNN total g…
S33 Du (2025)	Mixed/Multiple	Random Forest (RF) regression	Spatial	Global spatiotemporal livestock distribution …	Yes	Model level: r = 0.65 0.86; Province/state level: r = 0.97; County level: r = 0.78; Pixel level…
S34 Duan (2022)	Mixed/Multiple	Random Forest	Grazing/pasture	Rangeland degradation sensitivity assessment …	Yes	RF model AUC=1.00; top variable importance: vegetation cover type (>90% MDA), grazing intensity…
S35 Duku (2025)	Mixed/Multiple	Random Forest	Grazing/pasture	Carrying capacity (TLU/ha)	Yes	OOB accuracy >90%; Pearson r >0.9; mean prediction bias ~7%
S36 Elahi (2019)*	Other	ANN (Backpropagation Neural Network)	Energy	Energy use efficiency optimization; energy sa…	Yes	R^2=0.67 (validation); R^2=0.63 (overall)
S37 Elahi (2022)*	Poultry	ANN (Backpropagation Neural Network, Levenber…	Energy	Farm energy use efficiency optimization; ener…	Yes	R^2=0.83, MAPE=14.42, EF=0.79 (at 10 neurons in hidden layer)

Study	Species	Primary algorithm	Application domain	Sustainability outcome	DSS	Best reported performance
S38 Ellis (2020)	Other	Support Vector Regression (SVR)	Enteric	In vitro methane (CH_4) production from rumen …	No	SVR: CCC=0.917, RMSPE=8.3% (CH_4i); ANN: CCC=0.907, RMSPE=9.5% (CH_4d), on internal evaluation da…
S39 Embaby (2025)	Cattle	Gasformer	Enteric	Enteric methane	Partial	Gasformer mIoU=85.1%, mFscore=91.72%, FPS=64.56, GFLOPs=9.921, Params=3.65M; class wise IoU: Ba…
S40 Fang (2025)	Mixed/Multiple	Random Forest	Grazing/pasture	Seasonal pasture distribution and spatiotempo…	Yes	OA exceeded 93.10% across all six time periods (range 93.10 96.89%); Kappa 0.80 0.89; MICE 0.80…
S41 Fernandes (2024)	Cattle	SVR	Grazing/pasture	Pasture biomass and forage quality monitoring…	Yes	Dry leaf FM: SVR R^2=0.62, RMSPE=35.69 g/m², CCC=0.78; Dry green FM: SVR R^2=0.64, RMSPE=63.48 g/…
S42 Foschi (2025)	Cattle	Gaussian Kernel Regression (best performer fo…	Manure/W2E	CO_2 eq emissions (carbon footprint) per 1 kg …	Yes	Gaussian Kernel Regression: RMSE=14.65% (on ML imputed complete dataset); baseline before imput…
S43 Gao (2023)*	Mixed/Multiple	Support Vector Regression	Grazing/pasture	Grassland desertification index; soil compact…	Yes	SVM regression R=0.88 (soil evaporation at 10 cm); NAR temperature model R=0.99; overall desert…

Study	Species	Primary algorithm	Application domain	Sustainability outcome	DSS	Best reported performance
S44 Guarnido-Lopez (2023)*	Cattle	Changepoint detection (Binseg method)	Feed	Nitrogen excretion	Yes	MP concentration: exponential decay model AIC=485, BIC=487, RMSE=2.33; NE concentration: quadra…
S46 Hamidi (2023)	Cattle	Random Forest	Grazing/pasture	Pasture utilization and herbage on offer chan…	Yes	RF model mean R^2=0.43, RMSE=5.59 min (active time prediction); RGBVI vs herbage on offer R^2=0.5…
S47 Handan-Nader (2019)	Mixed/Multiple	CNN (Inception V3 with transfer learning)	Spatial	CAFO facility detection and mapping for envir…	Yes	Poultry: ROC AUC=0.972 (all images), 0.998 (non occluded); PR AUC=0.917 (all), 0.980 (non-occlu…
S48 Hansen (2020)	Cattle	Gradient component wise boosting (best for CO…	Grazing/pasture	Direct CO_2 emissions (kg) per MJ forage harve…	Yes	CO_2 test error: 1.112×10^{-3} (gradient CW boosting); cost test error: 0.0285 (RF); capacity test …
S49 Hansen (2025)	Cattle	Artificial Neural Network (ANN)	GHG inventory	GHG emissions prediction (CO2 eq per function…	Yes	GHG: ANN best (RMSE=0.50, R^2=0.86), MLR second (RMSE=0.68, R^2=0.74); E intensity: SVM best (RMS…
S50 Hempel (2020a)	Cattle	Gradient Boosting	Building	Ammonia (NH_3) emissions from naturally ventil…	Yes	Gradient Boosting: predicted mean emission within <20% of measured value (1.406 g h^{-1} LU^{-1}); be…

Study	Species	Primary algorithm	Application domain	Sustainability outcome	DSS	Best reported performance
S51 Hempel (2020b)	Cattle	Gradient Boosting	Building	Methane (CH4) emission factor prediction from…	No	Best $R^2 \approx 0.66$; MAE ≈ 1.20 g h^{-1} LU^{-1}; relative error generally <10% for ensemble methods
S52 Hosseinzadeh-Bandbafha (2015)	Cattle	Multi layer perceptron (MLP) with back-propag…	GHG inventory	GHG emissions (CO_2eq) from dairy farm operati…	Partial	Training R=0.999, Test R=0.968, All R=0.991; output energy: R^2=0.7191, RMSE=1.5184, MAPE=3.56%;…
S53 Hou (2023)	Cattle	Random Forest	Grazing/pasture	Grazing intensity / stocking rate effects on …	Yes	Random forest 10 fold cross validation accuracy = 91%, kappa = 85%; K means vs random forest cl…
S54 Irisarri (2025)	Cattle	Random Forest	Grazing/pasture	Forage quality prediction (CP, NDF, ADF, WSC)…	Yes	CP: R^2=0.86, RMSE=0.68, MAE=0.44. NDF: R^2=0.84, RMSE=1.36, MAE=0.87. WSC: R^2=0.81, RMSE=1.11, M…
S55 Jakimow (2018)	Cattle	Random Forest	Grazing/pasture	Pasture management intensity / cattle ranchin…	Yes	RF out of bag score 93.8%; class F1-scores 85 98%; area adjusted OAA ≥85% for all annual maps (…
S56 Jaman (2023)	Mixed/Multiple	Artificial Neural Network (feedforward backpr…	Manure/W2E	Biogas/biomethane production from anaerobic c…	Partial	ANN2: R^2=0.97164, RMSE=0.0164, MSE=0.00027; best kinetic (First Order): mean R^2=0.94035, mean R…

Study	Species	Primary algorithm	Application domain	Sustainability outcome	DSS	Best reported performance
S57 Jeong (2022)*	Cattle	U-Net (CNN)	Manure/W2E	Dairy enteric and manure CH_4 emissions (spati…	Partial	Image segmentation accuracy ~90%; r=0.95 (herd population vs. Vista CA at 0.1°); r=0.96 (CH_4 em…
S58 Ji (2025)	Mixed/Multiple	Random Forest	Grazing/pasture	Grazing induced vegetation breakpoint detecti…	Partial	Random Forest detected 44% of summer camp movements (vs. 28% for BFAST); coherence prediction R…
S59 Jia (2021)	Pigs	Long Short Term Memory (LSTM) neural network	Feed	Production efficiency of pig breeding under e…	Yes	LSTM prediction accuracy >80.1% across all three data sets (national pricing, market price, web…
S60 Jobarteh (2025)	Mixed/Multiple	LSTM (Long Short Term Memory), best performer…	Manure/W2E	CH_4 and CO_2 atmospheric concentrations attrib…	Yes	LSTM: RMSE=10 for CH_4; RMSE=362 for CO_2. ARIMA: RMSE=17 (CH_4), 615 (CO_2). XGBoost: RMSE=19 (CH_4…
S61 Kolluru (2023)	Mixed/Multiple	Random Forest regression (90:10 split, 10 km …	Grazing/pasture	High resolution (1 km²) gridded livestock den…	Partial	2015 small ruminants (10 km buffer, 90:10): CV R^2=0.76, RMSE=17.53, MAE=4.87; test R^2=0.82, RMS…
S62 Kuchler (2020)	Cattle	Random Forest (RF)	Grazing/pasture	Mapping integrated crop livestock (iCL) syste…	Partial	RF with raw NDVI+EVI: OA=0.96, Kappa=0.94, avg F score=0.96; SVM with raw NDVI+EVI: OA=0.95, Ka…

Study	Species	Primary algorithm	Application domain	Sustainability outcome	DSS	Best reported performance
S63 Kuchler (2022)	Cattle	Random Forest (RF, 100 trees)	Spatial	Monitoring adoption of integrated crop-livest…	Yes	Level 1 (SC/DC): mean OA=0.89 (sd=0.03); Level 2 (SCo/SCe/iCL): mean OA=0.84 (sd=0.07); Level 3…
S64 Küçüktopcu (2021)	Poultry	ANFIS SC (Adaptive Neuro Fuzzy Inference Syst…	Manure/W2E	Ammonia (NH_3) concentration in poultry buildi…	Yes	Best validation: ANFIS SC (3,0.1) with T, RH, V → R^2=0.858; RMSE=1.130 ppm; MRPE=4.032%
S65 Lei (2020)	Other	Random Forest	Grazing/pasture	Grazing intensity (GI) spatial temporal distr…	Yes	RGI PA (full monitoring period) training sample fit MAE=0.5298, r^2=0.9684; independent validati…
S66 Lemphane (2024)	Sheep	Neural Network (ANN)	Grazing/pasture	Pasture height prediction for sustainable gra…	Yes	ANN temperature RMSE = 1.225 (vs. regression 1.985); ANN rainfall RMSE = 1.746 (vs. regression …
S67 Li (2016)	Mixed/Multiple	ANN (feedforward neural network with back pro…	Manure/W2E	Biogas methane yield from anaerobic codigesti…	Partial	CH_4 yields: PC ANN R^2 = 0.782 (validation), MAE = 16.36; COD: PC ANN R^2 = 0.875, MAE = 186.79; …
S68 Li (2024)	Mixed/Multiple	Random Forest (RF)	Grazing/pasture	Grassland carrying capacity; grass livestock …	Yes	Land cover OA=93.88%, Kappa=0.902; Grassland precision=93.2%, recall=88.5%; AGB model R=0.78, R…

Study	Species	Primary algorithm	Application domain	Sustainability outcome	DSS	Best reported performance
S69 Li (2025)	Mixed/Multiple	Random Forest	Grazing/pasture	Interannual variability of forage production …	Yes	RF1 (remote sensing): 70% overall accuracy; RF2 (climate only): 71% overall accuracy; RF3 (clim…
S70 Lima (2023)*	Goat	CNN (modified LeNet 5 architecture: 6 and 16 …	Grazing/pasture	Forage cactus density as indicator of grazing…	Yes	Normalized confusion matrix: 0.99 accuracy for both palm and non palm classes; false positive r…
S71 Liu (2025)	Cattle	ANFIS	Building	Ammonia emissions; Methane emissions	Partial	NH_3: R^2 = 0.9270 (gaussmf), NRMSE = 0.1751, MAPE = 7.18%; CH_4: R^2 = 0.8977 (trapmf), NRMSE = 0….
S72 Liu (2016)	Sheep	Multilayer Perceptron (MLP), best model: MLP …	Enteric	Prediction and quantification of methane prod…	Partial	LDA PT LFER: Sn=0.963, Sp=0.935, Ac=0.947, MCC=0.894 (training and validation); Best MLP (PT NL…
S73 Liu (2024)	Mixed/Multiple	Random Forest (RandomForestRegressor, scikit-…	Other	1 km gridded livestock density maps enabling …	Yes	Model accuracy: total 92.88%, sheep&goats 91.35%, cattle 89.96%, horses 89.86%. R^2: total 0.958…
S74 Lo (2022)	Mixed/Multiple	Multiple Linear Regression (MML)	Grazing/pasture	Dry season forage availability monitoring for…	Yes	Best BH model (Sentinel-2 MML): R^2 = 0.74, RMSE = 378 kg DM/ha, RRMSE = 26%, NSE = 0.76; Best B…

Study	Species	Primary algorithm	Application domain	Sustainability outcome	DSS	Best reported performance
S75 Lo (2024)	Mixed/Multiple	Multilinear Regression (MLR)	Grazing/pasture	Dry season rangeland forage quality (crude pr…	Yes	MLR best models (CP): HQ R^2=0.81 RRMSE=11%, LQ R^2=0.72 RRMSE=11%, HLQ R^2=0.70 RRMSE=12%. ADF: H…
S76 Long (2025)*	Mixed/Multiple	Random Forest	Manure/W2E	Biogas production optimization (+12%); H2S em…	Yes	RF: Accuracy=0.91, R^2=0.60 (gas production); Accuracy=0.93, R^2=0.71 (methane %); Accuracy=0.91,…
S78 Lush (2018)	Sheep	Random Forest	GHG inventory	N_2O emissions from livestock urination (spati…	Partial	Urination: best precision 90.0% (10s window), best recall 72.2% (5s window). Overall model Kapp…
S79 Lyu (2024)	Mixed/Multiple	Random Forest	Spatial	Human Appropriation of Net Primary Production…	Yes	Specific values reported in **Figure S1.** (supplementary); study used mean of top 10 of 50 RF models …
S80 Malek (2024)	Cattle	Maximum Entropy (MaxEnt)	Grazing/pasture	Grazing intensity and spatial distribution	Partial	AUC = 0.71 (Atlantic) to 0.87 (Boreal/Nemoral/Alpine North)
S81 Marin (2022)	Cattle	Random Forest	Grazing/pasture	Grassland spatiotemporal dynamics	Partial	Global Accuracy 0.94 0.97; Kappa 0.87 0.93

Study	Species	Primary algorithm	Application domain	Sustainability outcome	DSS	Best reported performance
S82 Martinsson (2024)	Cattle	Matrix completion for causal panel data (nucl…	DSS/planning	Farm level GHG emissions efficiency (eco-effi…	Yes	Matrix completion RMSE: 0.17 for GHG emissions efficiency; per factor RMSE: off farm income 0.3…
S83 McParland (2024)	Cattle	Bayesian Regularized Neural Network (brnn R p…	Breeding	Predicting individual cow daily enteric metha…	Yes	Best strategy: NN with weighted average MIR + milk yield + DIM, flanking 6 d methane phenotype;…
S84 Meng (2023)	Mixed/Multiple	Random Forest	Grazing/pasture	Gridded grazing intensity (sheep units per pi…	Yes	Proposed M4 method (RF with partition + CSFs) R^2=0.80, 35.59% higher accuracy than traditional …
S85 Monteiro (2024)	Cattle	Ridge Regression	Feed	Feed efficiency (residual feed intake) linked…	Yes	RFI: R^2=0.36 (microbiome alone), R^2=0.89 (full model with microbiome), F1=0.75; MFE: R^2=0.92 (f…
S86 Mota (2024)	Cattle	Support Vector Regression (SVR) with Radial B…	Breeding	Feed efficiency (ratio of ADG to DMI); residu…	Partial	SVR: accuracy 0.62 0.69 across traits (ADG=0.67, DMI=0.62, FE=0.64, RFI=0.69); MTGBLUP: 0.62 0.…
S87 Nakashima (2025)	Cattle	YOLOv5x	Grazing/pasture	Cattle dung distribution mapping for sustaina…	Yes	YOLOv5x dung detection: mAP=0.75, precision=0.77, undetected rate=0.23; reliable identification…
S88 Naturinda (2025)	Cattle	YOLOv4	GHG inventory	Enteric methane (CH_4) and nitrous oxide (N_2O)…	Partial	Average F1=88.9%, Precision=97%, Recall=82.9%, mAP=80%

Study	Species	Primary algorithm	Application domain	Sustainability outcome	DSS	Best reported performance
S90 Ndlovu (2025)*	Cattle	XGBoost Regressor	Grazing/pasture	Global warming potential (kg CO_2 eq kg^{-1} FPCM…	Yes	XGBoost: R^2=0.9878 (GWP), R^2=0.9857 (EP), R^2=0.9857 (AP), R^2=0.9832 (FRDP); average R^2≈99%; GWP…
S91 Negussie (2022)	Cattle	Random Forest	Enteric	Enteric methane (CH_4) production (g/d)	Yes	Within herd: r=0.84, RMSE=18.5% (21k dataset, RF with imputed DMI); Between herd: r=0.63, RMSE=…
S92 Nsabiyeze (2025)	Sheep	ANN (best for CO_2 prediction, R^2=0.995); KNN …	GHG inventory	Prediction of carbon emissions (CO_2 eq) from …	Yes	ANN CO_2: R^2=0.995, RMSE=0.671, MAE=0.602; KNN energy efficiency: R^2=0.989, RMSE=0.001, MAE=0.02…
S93 Obileke (2024)	Cattle	ANN (feed forward backpropagation, Levenberg-…	Manure/W2E	Daily volume of biogas yield (m^3/day) from co…	Partial	NR
S94 Ocholla (2025)	Mixed/Multiple	YOLOv5	Grazing/pasture	Livestock population monitoring from satellit…	Partial	U-Net with Tversky Loss F1=87.4% on LUMO Pleiades test data; YOLOv5 F1=85.5% on LUMO WV3; YOLOv…
S95 Ogungbuyi (2023)	Sheep	ANN	Grazing/pasture	Grazing impact	Yes	TSDM: MAE=745 kg DM/ha, RMSE=904 kg DM/ha, R^2=0.14; Standing green: MAE=703 kg DM/ha, RMSE=880 …

Study	Species	Primary algorithm	Application domain	Sustainability outcome	DSS	Best reported performance
S96 Ogungbuyi (2024)	Sheep	Random Forest	Grazing/pasture	Pasture biomass estimation for sustainable gr…	Yes	UAS 3D photogrammetry: R^2=0.75, RMSE=1240 kg DM/ha, MAE=980 kg DM/ha; S2-RF: R^2=0.56, RMSE=2140…
S97 Okwu (2023)*	Mixed/Multiple	Fuzzy Mamdani Model (FMM)	Manure/W2E	Biogas and methane yield from co digested cow…	Partial	FMM: R^2=1.0, RMSE=0, MAE=0, AAD=0; ANN: R^2=1.0, RMSE=0.00022, MAE=0.000062, AAD=0.00012; RSM: R…
S98 Olatunji (2023)*	Poultry	ANFIS	Manure/W2E	Biogas/methane yield from livestock waste co-…	Partial	ANFIS FCM5clusters test: RMSE=2.7629, MAE=2.4046, MAD=2.2517, MAPE=6.4563%; RSM: R^2=0.5879
S99 Parente (2019)	Cattle	Random Forest	Grazing/pasture	Spatio-temporal dynamics of Brazilian pasture…	Partial	Overall accuracy ~90% across all 33 years; user accuracy ~95% (from 2000 onward); producer accu…
S100 Park (2023)	Pigs	LSTM (best short term W MAE); Transformer (be…	Building	Ammonia (NH_3) concentration prediction in mec…	Partial	LSTM W MAE=1.78 ppm (2 week prediction); Transformer L MAE=1.83 ppm (4 week training window, lo…

Study	Species	Primary algorithm	Application domain	Sustainability outcome	DSS	Best reported performance
S101 Park (2024)	Cattle	k Nearest Neighbor (KNN)	Enteric	Enteric methane production (g/d) predicted fr…	Partial	KNN: R^2=1.00, MAE=0.00, RMSE=0.00 for VFA prediction; KNN methane KM1 & KM2 clusters R^2=1.00; A…
S102 Paz-Kagan (2023)	Goat	Random Forest	Grazing/pasture	Vegetation cover change trends driven by goat…	Yes	R^2=0.93, MAE=0.184 Z score units (Model 1, all herds combined)
S103 Pei (2022)	Cattle	ELM (Extreme Learning Machine)	Manure/W2E	Energy production (biogas) from anaerobic co-…	Partial	ELM: R^2=0.9574, MAE/10=0.678
S104 Pence (2023)	Mixed/Multiple	Support Vector Machine (ν SVM, 4th degree pol…	Manure/W2E	Biogas production potential from animal manur…	Yes	R^2=0.999 (SVM for biogas, CO_2, electricity, thermal energy); R^2=0.997 (LR for coal); R^2=0.977 (…
S105 Pence (2024a)	Mixed/Multiple	CatBoost (best overall performer across most …	Manure/W2E	Direct N_2O emissions, indirect N_2O emissions,…	Partial	CatBoost Scenario 1 (2021): R^2=0.996 (direct N_2O), 0.996 (indirect N_2O), 0.992 (CH_4 manure), 0.…
S106 Pence (2024b)	Mixed/Multiple	XGBoost Regressor (XGBR)	Manure/W2E	CH_4 emissions from livestock manure managemen…	Partial	XGBR test: R^2=0.9883 (biogas), R^2=0.9835 (CH_4 tier1), R^2=0.9773 (CH_4 tier2); MAPE=0.46%, 1.07%,…
S107 Peng (2023)	Cattle	Random Forest	Enteric	Enteric methane (CH_4) emission prediction and…	Partial	RF regression: R^2=0.56 (Wallace, microbiome+metadata), R^2=0.42 (Merged, microbiome+metadata), R…

Study	Species	Primary algorithm	Application domain	Sustainability outcome	DSS	Best reported performance
S108 Peng (2019)	Mixed/Multiple	Particle Swarm Optimization (PSO)	GHG inventory	Enteric methane (CH_4) from livestock; CH_4 fro…	Partial	RMSE and MAE near zero for all species (e.g., cattle enteric: RMSE=3.58E 06, MAE=3.17E 06; goat…
S109 Pereira (2025)	Mixed/Multiple	Random Forest	Grazing/pasture	Native Cerrado vegetation loss (forest, savan…	Yes	2015 classification: Overall Accuracy 71.53%, Kappa 0.663; 2020 classification: Overall Accurac…
S110 Perumal (2024)	Mixed/Multiple	LSTM	GHG inventory	Enteric methane emissions from livestock (nat…	Yes	LSTM: RMSE=61.07 (or 62.98), MAPE=0.35%, SMAPE=0.34%, MDA=0.95/0.98; ARIMA: RMSE=44.29 (or 44.5…
S111 Qi (2026)‡	Mixed/Multiple	Random Forest (FA ACO RF combined model)	Other	Carbon emissions from combined agriculture an…	Yes	Prediction accuracy 99.19% (FA ACO RF); MSE=0.00113, MAE=0.02861, RMSE=0.03355
S112 Ramirez-Agudelo (2025)	Cattle	YOLOv8n	Enteric	Enteric methane (CH_4) emissions monitoring at…	Yes	YOLOv8n: Precision=1.00, Recall=1.00, F1-Score=1.00, Accuracy=1.00, Latency=24 ms
S113 Reza (2022)*	Cattle	RSM ANN (feed forward 3:5:1, Levenberg Marqua…	Manure/W2E	NH_3 N removal efficiency (%) from anaerobical…	Partial	NR
S114 Rico-Contreras (2017)*	Poultry	Fuzzy Logic (Mamdani inference)	Manure/W2E	GHG emissions mitigation from poultry litter …	Yes	FL: R^2=0.9288; ANN: R^2=0.9291 (moisture prediction on 20 validation cases)

Study	Species	Primary algorithm	Application domain	Sustainability outcome	DSS	Best reported performance
S115 Rodriguez (2022)	Pigs	Multilayer Perceptron ANN (3 4 4 1 architectu…	Building	CO_2 emission rate from weaned piglet housing,…	No	ANN 60 min: r=0.63, RMSE=1.21 mg s^{-1}, MARE=0.23; ANN 10 min: r=0.56, RMSE=1.27, MARE=0.25; Regr…
S116 Rouegui (2025)‡	Sheep	Gradient Boosting Machine (GBM)	Manure/W2E	Biochemical methane potential (BMP) predictio…	Yes	GBM RMSE = 5.05 (vs. RFR RMSE = 14.75; LM RMSE = 17.69)
S117 Saha (2025)	Mixed/Multiple	Random Forest	Spatial	Identification of AFO locations for nutrient …	Partial	Overall accuracy=87%; Recall (AFO)=0.95; Precision (AFO)=0.89; F1=0.86 (weighted avg); AUC=0.73
S118 Sahraei (2025)	Cattle	LSTM	Enteric	Enteric methane (CH_4) emissions from dairy co…	Partial	LSTM (S1, all features): R^2=0.88, MBE=13.55 ppm, RMSE=44.87 ppm, CRE=18.92%; LSTM (S4, moderate…
S119 Şenol (2025)	Poultry	ANN LM (Levenberg Marquardt)	Manure/W2E	Poultry manure biogas/biomethane potential; C…	Partial	ANN LM: R^2=0.9914, RMSE=4.00, MAPE=0.71%; ANN BR: R^2=0.9909, RMSE=4.11, MAPE=1.22%; ARIMA: R^2=0…
S120 Şenol (2021)	Cattle	Neural Network (ANN), feedforward with Bayesi…	Manure/W2E	Bovine manure based biomethane potential (bil…	Yes	Training R = 1; Test R = 1; MSE for TIPP = 0.0092; RMSE for TIPP = 0.0961; MSE for BPPC = 7.63 …

Study	Species	Primary algorithm	Application domain	Sustainability outcome	DSS	Best reported performance
*S121 Şenol (2024a)**	Cattle	Artificial Neural Network (ANN), Levenberg Ma…	Manure/W2E	Biogas yield optimization from anaerobic co d…	Partial	ANN OPBC: R^2=0.9999, RMSE=0.55, MAPE=2.1, MAE=0.347; ANN Co UPBC: R^2=1.0000, RMSE=5.12E 03; RSM…
S122 Şenol (2024b)	Other	Artificial Neural Network (ANN) with Levenber…	Manure/W2E	Bovine manure based biogas potential (BMBP) /…	Yes	LM ANN overall best: R^2 up to 0.9820 (Mediterranean), MAPE 1.122 3.436 across regions; BR ANN b…
S123 Serpa-Imbett (2025)	Cattle	Ridge Regression	Grazing/pasture	Forage biomass (dry matter yield) and nutriti…	Yes	Ridge: Biomass R^2=0.815 0.934 (shade/sun); Quality traits R^2=0.938 0.998 (CF, NDF, ADF, LIG, CP…
S124 Shadpour (2022)	Cattle	BRANN (Bayesian Regularisation ANN) with MIRS…	Breeding	Individual enteric CH_4 emission (g/d) in lact…	Yes	Best model (linear BRANN, set 8, validation B): r=0.704, RMSE=70.83 g/d, RPD=1.43
S125 Shea (2022)	Pigs	Random Forest	Manure/W2E	Detection of liquid manure application events…	Yes	Dormant season model: overall accuracy 97%, Kappa 96.54%; early growing season model: accuracy …
S126 Shetty (2023)	Cattle	GLM Stacked Ensemble (E C MAN) with 26 base l…	Manure/W2E	E. coli levels in dairy manure effluent (micr…	Partial	RMSE=0.51 (transformed), R^2=0.71 (training); RMSE=0.72, R^2=0.65 (testing); reverse transformed …

Study	Species	Primary algorithm	Application domain	Sustainability outcome	DSS	Best reported performance
S127 Shi (2023)	Cattle	Support Vector Machine (SVM)	Feed	Nitrogen use efficiency (NUE) and nitrogen lo…	Partial	Within herd NUE: R²=0.66, SpearR=0.82, RE=0.15 (SVM, Model 5, SNV spectra); External NUE: R²=0….
S128 Shorten (2021)	Cattle	Majority Vote (MV) ensemble of 7 classifiers	Grazing/pasture	Nitrogen excretion from cattle urination; uri…	Yes	Urination event detection (validation): F1=0.88, Precision=0.94, Sensitivity=0.83; Duration pre…
S129 Silveira (2023)‡	Cattle	K means clustering	Breeding	"Sustainable animal" composite indicator inte…	Yes	EFA cumulative variance explained 83.59 89.73% per indicator; CDA correctly classified 78.7% (r…
S130 Simion (2025)	Other	CNN	Grazing/pasture	Pasture/grassland condition monitoring; veget…	Yes	CNN classification accuracy = 95% on independent test set; Pearson r between ExGR and precipita…
S131 Ahmad (2023)*	Cattle	ANN (MLP feed forward back propagation, 3 6 1…	Manure/W2E	Biogas (energy) production from anaerobic co-…	Yes	ANN: R=0.9888, MAPE=3.89%, RMSE=0.0563, MSE=0.0032; RSM: R²=99.78%, adjusted R²=99.64%; optimiz…
S132 Soto (2024)	Mixed/Multiple	Random Forest	Grazing/pasture	Rangeland health indicators (land cover class…	Yes	LCC overall accuracy=85.57%, kappa=0.832; VFC bare ground R²=0.795, nRMSE=0.123, p<2.2×10⁻¹⁶, s…

Study	Species	Primary algorithm	Application domain	Sustainability outcome	DSS	Best reported performance
S133 Su (2025a)	Mixed/Multiple	BMogLSTM-KAN	Grazing/pasture	Grazing intensity (livestock stocking rate pe…	Yes	BMogLSTM-KAN: R^2=0.93, RMSE=0.68, MAE=0.53. MogLSTM: R^2=0.90, RMSE=0.80. RF: R^2=0.87, RMSE=0.96…
S134 Su (2025b)	Sheep	Random Forest (RF)	Grazing/pasture	Grazing intensity quantification for sustaina…	Yes	NDVIp reconstruction (RF, type level stratification): R^2 = 0.864; AGBs model: R^2 = 0.76; AGBp m…
S136 Tonn (2019)	Cattle	Random Forest	Grazing/pasture	Grazing induced sward patch stability (short …	Partial	F1 scores: 0.851 (2005), 0.943 (2010), 0.863 (2013), 0.977 (2015); Cohen's kappa: 0.663, 0.903,…
S137 Tryhuba (2025)*	Cattle	Multilayer Perceptron (MLP)	Manure/W2E	CO_2 equivalent emissions from livestock waste…	Yes	MLPopt: MSE=0.0005, MAE=0.0175, R^2=0.9896, MAPE=6.51%
S138 Tulbure (2024)	Poultry	Deep learning CNN (Robinson et al. 2022 U-Net…	Spatial	Spatial distribution and density of poultry C…	Yes	54% reduction in overestimation of poultry CAFO density per census tract after heuristics; MD f…
S139 Valle (2025)*	Cattle	YOLOv5s	Building	Water use efficiency for sprinkler based cow …	Yes	Overall training mAP=0.940; accuracy=0.924; mAP=0.80 on 100 random dewarped images; mAP=0.73 0.…

Study	Species	Primary algorithm	Application domain	Sustainability outcome	DSS	Best reported performance
S140 Vaysi (2024)*	Mixed/Multiple	Radial Basis Function (RBF) neural network	Manure/W2E	CH_4, CO_2, N_2O emissions from livestock manure…	Partial	RBF: R^2=0.99 (GHG), R^2=0.99 (poultry population), R^2=0.99 (sheep/goat), R^2=0.97 (cattle); MAPE=…
S141 Vendruscolo (2020)	Cattle	Random Forest	Manure/W2E	Biogas/methane production from livestock manu…	No	NR
S142 Wallace (2019)	Cattle	Ridge Regression	Enteric	Enteric methane (CH_4) emissions; CO_2 emission…	Partial	Rumen propionate prediction r^2 approaching 0.9; methane (CH_4 g/day) prediction r^2 up to 0.4; mi…
S143 Wang (2023a)	Goat	Random Forest	Enteric	Growth performance (ADG) and lactation perfor…	Partial	ADG classification: Prevotellaceae UCG 003 AUC=0.813, Ruminococcus gauvreauii AUC=0.804; A:P ra…
S144 Wang (2025)	Cattle	Random Forest	Enteric	Enteric methane (methanogenesis pathway activ…	No	Variance explained=30.19%; Treponema succinifaciens r=−0.570 (p<0.001); T. bryantii A r=−0.486 …
S145 Wang (2024)	Cattle	GA BP (Genetic Algorithm Backpropagation Neur…	Enteric	Enteric methane (in vitro rumen methane produ…	Partial	GA BP methane: R^2=0.96, RMSE=0.89 mL/g; GA BP acetic acid: R^2=0.94, RMSE=1.785 mmol/L

Study	Species	Primary algorithm	Application domain	Sustainability outcome	DSS	Best reported performance
S146 Wang (2023b)	Cattle	Stacking ensemble (GBRT + GPR + Bagging Regre…	Enteric	Enteric methane prediction from diet composit…	Yes	Methane: R^2=0.928, RMSE=0.968 mL/g; AA: R^2=0.888, RMSE=1.975 mmol/L; PA: R^2=0.924, RMSE=0.74 mm…
S147 Wawrzyniak (2023a)	Cattle	ANN (MLP, multilayer perceptron, single hidde…	Manure/W2E	Biogas production potential from cattle manur…	Partial	Correlation ≈ 0.999 (training) across most models; ~99% fitting quality; best model (southern m…
S148 Wawrzyniak (2023b)	Cattle	Multi Layer Perceptron (MLP)	Manure/W2E	Biogas/biomethane production potential from c…	Yes	Mazowieckie manure (MLP 7 5 1): SD ratio 0.0106, correlation 0.9999; Mazowieckie slurry (MLP 7-…
S149 Werner (2024)	Mixed/Multiple	Temporal Convolutional Neural Network (TempCN…	Spatial	Mapping and monitoring of integrated crop liv…	Yes	TempCNN with DF data cube: SA1 OA=90.0%, F1-Score (ICLS)=98.6% to 100.0%; SA2 OA=95.6%, F1-Scor…
*S150 Wu (2025a)**	Mixed/Multiple	Random Forest	Other	Carbon sequestration (AGB + BGB)	Yes	RF model R^2=0.92; LPJ GUESS validation R^2=0.83
S151 Wu (2025b)	Mixed/Multiple	Geographically Weighted Ridge Regression (GWR…	Grazing/pasture	Gridded livestock spatial distribution mappin…	Yes	GWRR: training R^2=0.752, validation R^2=0.725, township RMSE=73.627, MAE=59.123; RF+residual ass…

Study	Species	Primary algorithm	Application domain	Sustainability outcome	DSS	Best reported performance
S152 Wu (2025c)*	Pigs	CatBoost	Manure/W2E	NH_3 N emissions; N_2O N emissions; nitrogen lo…	Yes	CatBoost (C/N): training R^2=0.9996, RMSE=0.043, MAE=0.034; test R^2=0.933, RMSE=1.861, MAE=1.066…
S153 Xi (2024)	Mixed/Multiple	Support Vector Machine Regression (SVMR)	Grazing/pasture	Grazing intensity / livestock carrying capaci…	Yes	R^2=0.98, MAE=0.068 SU/km², RMSE=0.07 SU/km² (SVMR, training); R^2=0.95, MAE=0.07, RMSE=0.11 (tes…
S154 Xie (2022)*	Pigs	CNN LSTM AM (hybrid Convolutional Neural Netw…	Manure/W2E	Ammonia (NH_3), carbon dioxide (CO_2), and hydr…	Yes	Concentration prediction: PLS NH_3 summer R^2=0.967, RMSE=0.094 ppm; Emission estimation: NH_3 win…
S155 Xing (2025)‡	Cattle	Stacking (meta learner: Ridge Regression)	Enteric	Ammonia (NH_3) emissions; urinary urea nitroge…	Yes	Stacking: milk yield R^2=0.85, MAE=2.537, RMSE=3.236; milk urea R^2=0.794, MAE=3.817, RMSE=5.076….
S156 Yang (2024)	Mixed/Multiple	Random Forest	Grazing/pasture	Grassland aboveground biomass (AGB) as proxy …	Yes	RF Region I: R^2=0.80, RMSE=823.75 kg/hm², Precision=83.22%; RF Region II: R^2=0.76, RMSE=595.37 …
S157 Ye (2025)	Cattle	KNN	Grazing/pasture	Grazing intensity spatial clustering and gras…	Partial	KNN + SMOTE ENN: F1=0.960 (continuous), F1=0.956 (rotational); CA=0.965 (continuous), CA=0.953 …

Study	Species	Primary algorithm	Application domain	Sustainability outcome	DSS	Best reported performance
S158 Yelmen (2019)	Poultry	Feed Forward Back Propagation ANN (FFBPANN)	Energy	Total energy equivalent of chicken meat outpu…	No	R^2=0.936; RMSE=0.232; MAE=0.019 (best model: ANN 7 12 2)
S159 Yu (2025)	Cattle	Backpropagation Neural Network (BPNN, 4 2 1 a…	Enteric	In vitro CH_4 production from rumen fermentati…	Partial	BPNN CH_4: r^2=0.93, RMSPE%=2.29%, CCC=0.96; MLR CH_4: r^2=0.91, RMSPE%=3.42%, CCC=0.92; BPNN CO_2: …
S160 Yusof (2014)*	Poultry	Artificial Neural Network (ANN)	Manure/W2E	Methane yield (mL CH_4/g VS) from anaerobic co…	Partial	R^2=0.998 (ANN); R^2=0.991 (RSM)
S161 Zaki (2024)*	Poultry	Random Forest Regression	Manure/W2E	Global warming potential (MT CO_2 eq/year) fro…	Yes	Pyrolysis RFR: training R^2=0.91, testing R^2=0.61, testing RMSE=6.70, testing MAE=4.75; HTC RFR:…
S162 Zhang (2023)	Sheep	glmmLasso (generalized linear mixed model wit…	Enteric	Enteric methane (CH_4) production (g CH_4/anima…	No	ANIM B glmmLasso: RMSPE=2.85 g/d, MAE=2.18 g/d, CCC=0.70; DMI B SCAD: RMSPE=1.92 g/kg DMI, MAE=…
S163 Zhang (2025)	Sheep	Random Forest	Grazing/pasture	Soil carbon and nitrogen mineralization rates…	Yes	Random forest for soil C mineralization: R^2=0.49, p<0.01; random forest for soil N mineralizati…
S164 Zhang (2024)	Pigs	XGBoost	Manure/W2E	Carbon emissions (CO_2, CH_4, N_2O as CO_2eq) fro…	Partial	Train R^2=0.99, Test R^2=0.97; Train RMSE=28.86, Test RMSE=58.78; Average RE=5.1%

Study	Species	Primary algorithm	Application domain	Sustainability outcome	DSS	Best reported performance
S165 Zhang (2022a)	Mixed/Multiple	Random Forest	Grazing/pasture	Grassland aboveground biomass (AGBp) and theo…	Partial	R^2=0.76 (P<0.001), nRMSE=10.87, Bias=0.05
S167 Zhou (2025)	Pigs	Closed Form Continuous Time neural network (C…	Building	Environmental anomaly detection in pig farm h…	Yes	Proposed CfC model: Accuracy=92.39%, Precision=92.08%, Recall=85.84%, F1=88.19%; outperforms Ti…
S168 Zhou (2024)	Mixed/Multiple	Extra Trees (ET)	Grazing/pasture	Grazing intensity mapping for grassland degra…	Partial	ET model: R^2=0.955, MAE=0.081 SU ha^{-2}, RMSE=0.164 SU ha^{-2} (county level test set); GDGI pixel l…
S169 Zou (2024)*	Pigs	Back propagation neural network (BP NN) with …	Manure/W2E	Methane production optimization from swine ma…	Yes	BP NN methane production: R^2 = 0.8967, RMSE = 7.0106, MAPE = 0.0135, TIC = 0.0119; BP NN total …
S170 Zwick (2024)	Cattle	Regularized Random Forest (RRF)	Grazing/pasture	Pasture biomass (dry matter) and forage quali…	Yes	CP: R^2=0.52 0.75, RMSE=1.66 2.22%; DM: R^2=0.47 0.65, RMSE=112 260 g/m^2; IVD: R^2=0.59 0.60 (Patí…

Note. Each study is identified by its number in S8 (Included studies); the Study column links to the corresponding entry. Suffixed years (e.g. 2022a, 2022b) distinguish included studies sharing a first author surname and year within this supplement; the main text assigns its own suffixes within its reference list. An asterisk marks studies whose functional status is Yes and a double dagger marks Unclear studies.

S8. Included studies

All 165 studies included in the systematic review, numbered S1 to S170 with five numbers vacant (S45, S77, S89, S135 and S166; Section S2, amendments 6 and 9) and ordered alphabetically by first author surname, with two exceptions: the entries for Fernández et al. (2023) and Ahmad et al. (2023) keep the positions assigned before their author lists were corrected at reconciliation (they had been listed under David and Singh), so that the numbering used throughout Table S15 is preserved. Suffixed years distinguish studies sharing a first author surname and year within this list.

S1. Alejo, L., Atkinson, J., Guzmán-Fierro, V., Roeckel, M., 2018. Effluent composition prediction of a two-stage anaerobic digestion process: machine learning and stoichiometry techniques. Environmental Science and Pollution Research 25, 21149–21163. *https://doi.org/10.1007/s11356-018-2224-7*

S2. Alemu, S.W., Bilton, T.P., Johnson, P.L., Perry, B.J., Henry, H., Dodds, K.G., McEwan, J.C., Rowe, S.J., 2025. Improving genomic prediction accuracy for methane emission and feed efficiency in sheep: integrating rumen microbial PCA with host genomic variation using neural network GBLUP (NN-GBLUP). Genetics Selection Evolution 57, 41. *https://doi.org/10.1186/s12711-025-00987-x*

S3. Ali, M.M., Ndongo, M., Yetilmezsoy, K., Bahramian, M., Bilal, B., Youm, I., Goncaloğlu, B.İ., 2021. Appraisal of methane production and anaerobic fermentation kinetics of livestock manures using artificial neural networks and sinusoidal growth functions. Journal of Material Cycles and Waste Management 23, 301–314. *https://doi.org/10.1007/s10163-020-01130-2*

S4. Altshuler, Y., Chebach, T.C., Cohen, S., Gatica, J., 2025. Assessing the methane-mitigating effects of feed additives on dairy cows: Validation of an AI-Based predictive model using a monensin feed additive. Animal Feed Science and Technology 329, 116483. *https://doi.org/10.1016/j.anifeedsci.2025.116483*

S5. Ashworth, A.J., Avila, A., Smith, H., Winzeler, T.E., Owens, P., Flynn, C., O'Brien, P., Philipp, D., Su, J., 2024. Predicting spatiotemporal patterns of productivity and grazing from multispectral data using neural network analysis based on system complexity. Agrosystems, Geosciences & Environment 7, e20571. https://doi.org/10.1002/agg2.20571

S6. Bao, C., Bagan, H., Te, T., Wang, Q., Boris, Z., Kinoshita, T., 2025. Spatiotemporal variability of methane concentrations driven by ruminant livestock in Mongolia: insights from satellite observations and machine learning. GIScience & Remote Sensing 62, 2582118. https://doi.org/10.1080/15481603.2025.2582118

S7. Barczyk, L., Six, J., Ammann, C., 2024. Partitioning and driver analysis of eddy covariance derived N2O emissions from a grazed and fertilized pasture. Agricultural and Forest Meteorology 359, 110278. https://doi.org/10.1016/j.agrformet.2024.110278

S8. Bartels, J., Tokgoz, K.K., A, S., Fukawa, M., Otsubo, S., Li, C., Rachi, I., Takeda, K.-I., Minati, L., Ito, H., 2022. TinyCowNet: Memory- and Power-Minimized RNNs Implementable on Tiny Edge Devices for Lifelong Cow Behavior Distribution Estimation. IEEE Access 10, 32706–32727. https://doi.org/10.1109/ACCESS.2022.3156278

S9. Basak, J.K., Arulmozhi, E., Moon, B.E., Bhujel, A., Kim, H.T., 2022a. Modelling methane emissions from pig manure using statistical and machine learning methods. Air Quality, Atmosphere & Health 15, 575–589. https://doi.org/10.1007/s11869-022-01169-0

S10. Basak, J.K., Kim, N.E., Shahriar, S.A., Paudel, B., Moon, B.E., Kim, H.T., 2022b. Applicability of statistical and machine learning–based regression algorithms in modeling of carbon dioxide emission in experimental pig barns. Air Quality, Atmosphere & Health 15, 1899–1912. https://doi.org/10.1007/s11869-022-01225-9

S11. Basak, J.K., Paudel, B., Deb, N.C., Kang, D.Y., Kang, M.Y., Roy, S.K., Shahriar, S.A., Kim, H.T., 2024. Modeling ammonia concentration in swine building using biophysical data and machine learning algorithms. Computers and Electronics in Agriculture 225, 109269. https://doi.org/10.1016/j.compag.2024.109269

S12. Bhattad, S., Ahmed, A.A., Abdel-Wareth, A.A.A., Lohakare, J., 2025. An IoT-Based System for Measuring Diurnal Gas Emissions of Laying Hens in Smart Poultry Farms. AgriEngineering 7, 267. https://doi.org/10.3390/agriengineering7080267

S13. Bi, H., Neethirajan, S., 2024a. Mapping Methane—The Impact of Dairy Farm Practices on Emissions Through Satellite Data and Machine Learning. Climate 12, 223. https://doi.org/10.3390/cli12120223

S14. Bi, H., Neethirajan, S., 2024b. Satellite Data and Machine Learning for Benchmarking Methane Concentrations in the Canadian Dairy Industry. Sustainability 16, 10400. https://doi.org/10.3390/su162310400

S15. Boulanger-Lapointe, N., Ágústsdóttir, K., Barrio, I.C., Defourneaux, M., Finnsdóttir, R., Jónsdóttir, I.S., Marteinsdóttir, B., Mitchell, C., Möller, M., Nielsen, Ó.K., Sigfússon,

A.Þ., Þórisson, S.G., Huettmann, F., 2022. Herbivore species coexistence in changing rangeland ecosystems: First high resolution national open-source and open-access ensemble models for Iceland. Science of the Total Environment 845, 157140. https://doi.org/10.1016/j.scitotenv.2022.157140

S16. Cao, H., Xin, Y., Yuan, Q., 2016. Prediction of biochar yield from cattle manure pyrolysis via least squares support vector machine intelligent approach. Bioresource Technology 202, 158–164. https://doi.org/10.1016/j.biortech.2015.12.024

S17. Cao, T., Zheng, Y., Shang, B., Cong, Q., Cao, Q., Dong, H., 2025. Fast prediction of odor concentration along pig manure chain based on machine learning: Monitoring 20 instead of over 100 odorous substances. Computers and Electronics in Agriculture 233, 110146. https://doi.org/10.1016/j.compag.2025.110146

S18. Cardinale, S., Kadarmideen, H.N., 2022. Host Genome–Metagenome Analyses Using Combinatorial Network Methods Reveal Key Metagenomic and Host Genetic Features for Methane Emission and Feed Efficiency in Cattle. Frontiers in Genetics 13, 795717. https://doi.org/10.3389/fgene.2022.795717

S19. Chasen, E., Booth, E., Gratton, C., 2025. Simulating Pasture Yield Under Alternative Environments and Grazing Management in Wisconsin, USA. Agronomy 15, 437. https://doi.org/10.3390/agronomy15020437

S20. Chen, M., Li, H., Zhang, Z., Ren, R., Wang, Z., Feng, J., Cao, R., Hu, G., Liu, Z., 2025. Real-Time Pig Weight Assessment and Carbon Footprint Monitoring Based on Computer Vision. Animals 15, 2611. https://doi.org/10.3390/ani15172611

S21. Cheng, N., Wang, J., Cheng, H., Wang, X., 2025. Spatial estimation of veterinary antibiotics from husbandry to soil to river with resistance risk assessment in urban agglomerations. Environmental Research 273, 121212. https://doi.org/10.1016/j.envres.2025.121212

S22. Chernysh, Y., Chubur, V., Shtepa, V., Alekseevsky, D., Bataltsev, Y., Roubík, H., 2025. Anaerobic Digestion of Poultry Manure with Phosphogypsum Using Microbial Electrolysis and Ultrasound: A Neural Network Approach. Environmental Processes 12, 52. https://doi.org/10.1007/s40710-025-00794-y

S23. Choo, W., Raghavan, S., 2025. Predicting methane emissions in smallholder dairy systems: a clustering and ensemble learning approach. Frontiers in Sustainable Food Systems 9, 1668517. https://doi.org/10.3389/fsufs.2025.1668517

S24. Correa-Luna, M., Gargiulo, J., Beale, P., Deane, D., Leonard, J., Hack, J., Geldof, Z., Wilson, C., Garcia, S., 2024. Accounting for minimum data required to train a machine learning model to accurately monitor Australian dairy pastures using remote sensing. Scientific Reports 14, 16927. https://doi.org/10.1038/s41598-024-68094-3

S25. Dach, J., Koszela, K., Boniecki, P., Zaborowicz, M., Lewicki, A., Czekała, W., Skwarcz, J., Qiao, W., Piekarska-Boniecka, H., Białobrzewski, I., 2016. The use of neural modelling to estimate the methane production from slurry fermentation processes. Renewable and Sustainable Energy Reviews 56, 603–610. https://doi.org/10.1016/j.rser.2015.11.093

S26. Dai, X., Yang, Y., Zheng, H., Meng, N., Zhu, J., Li, R., Ma, J., Lu, Z., Li, Z., 2023. An integrated method to quantify human appropriation of net primary production in

grasslands of the Qinghai-Tibet Plateau. Applied Geography 158, 103055. https://doi.org/10.1016/j.apgeog.2023.103055

S27. Fernández, P.D., Gärtner, P., Nasca, J.A., Rojas, T., Gasparri, N.I., 2023. Beyond pastures, look at plastic: Using Sentinel-2 imagery to map silage bags to improve understanding of cattle intensity. Science of the Total Environment 855, 158390. https://doi.org/10.1016/j.scitotenv.2022.158390

S28. De Rosa, D., Basso, B., Fasiolo, M., Friedl, J., Fulkerson, B., Grace, P.R., Rowlings, D.W., 2021. Predicting pasture biomass using a statistical model and machine learning algorithm implemented with remotely sensed imagery. Computers and Electronics in Agriculture 180, 105880. https://doi.org/10.1016/j.compag.2020.105880

S29. Defalque, G., Santos, R., Bungenstab, D., Echeverria, D., Dias, A., Defalque, C., 2024. Machine learning models for dry matter and biomass estimates on cattle grazing systems. Computers and Electronics in Agriculture 216, 108520. https://doi.org/10.1016/j.compag.2023.108520

S30. Dhussa, A.K., Sambi, S.S., Kumar, Shashi, Kumar, Sandeep, Kumar, Surendra, 2014. Nonlinear Autoregressive Exogenous modeling of a large anaerobic digester producing biogas from cattle waste. Bioresource Technology 170, 342–349. https://doi.org/10.1016/j.biortech.2014.07.078

S31. Dong, P., Jing, C., Wang, G., Shao, Y., Gao, Y., 2024. The Estimation of Grassland Aboveground Biomass and Analysis of Its Response to Climatic Factors Using a Random Forest Algorithm in Xinjiang, China. Plants 13, 548. https://doi.org/10.3390/plants13040548

S32. Dong, R., Zhao, G., 2014. The use of artificial neural network for modeling in vitro rumen methane production using the CNCPS carbohydrate fractions as dietary variables. Livestock Science 162, 159–167. https://doi.org/10.1016/j.livsci.2013.12.033

S33. Du, Z., Yu, L., Zhao, Y., Li, Xinyue, Liu, X., Li, Xiyu, Hao, P., Chen, Z., Guo, Z., You, L., Ma, X., Wang, H., 2025. Annual global grided livestock mapping from 1961 to 2021. Earth System Science Data 17, 5543–5556. https://doi.org/10.5194/essd-17-5543-2025

S34. Duan, C., Shi, P., Zong, N., Zhang, X., Yu, C., 2022. Assessing Rangeland Sensitivity to Degradation in North Tibet. Rangeland Ecology & Management 84, 86–97. https://doi.org/10.1016/j.rama.2022.07.001

S35. Duku, C., Diro, G.T., Demissie, T., Dawit, S., 2025. Climate change impacts livestock carrying capacity in East Africa. Regional Environmental Change 25, 110. https://doi.org/10.1007/s10113-025-02440-7

S36. Elahi, E., Weijun, C., Jha, S.K., Zhang, H., 2019. Estimation of realistic renewable and non-renewable energy use targets for livestock production systems utilising an artificial neural network method: A step towards livestock sustainability. Energy 183, 191–204. https://doi.org/10.1016/j.energy.2019.06.084

S37. Elahi, E., Zhang, Z., Khalid, Z., Xu, H., 2022. Application of an artificial neural network to optimise energy inputs: An energy- and cost-saving strategy for commercial poultry farms. Energy 244, 123169. https://doi.org/10.1016/j.energy.2022.123169

S38. Ellis, J.L., Alaiz-Moretón, H., Navarro-Villa, A., McGeough, E.J., Purcell, P., Powell, C.D., O'Kiely, P., France, J., López, S., 2020. Application of Meta-Analysis and Machine

Learning Methods to the Prediction of Methane Production from In Vitro Mixed Ruminal Micro-Organism Fermentation. Animals 10, 720. https://doi.org/10.3390/ani10040720

S39. Embaby, M.G., Sarker, T.T., AbuGhazaleh, A., Ahmed, K.R., 2025. Optical gas imaging and deep learning for quantifying enteric methane emissions from rumen fermentation in vitro. IET Image Processing 19, e13327. https://doi.org/10.1049/ipr2.13327

S40. Fang, P., Wang, J., Xu, H., Li, R., Yu, L., Huang, S., Liang, Y., Zheng, P., 2025. Enhanced supervised classification of seasonal pastures on the Qinghai-Tibet Plateau (1990–2020) using Landsat optimal time window. GIScience & Remote Sensing 62, 2530310. https://doi.org/10.1080/15481603.2025.2530310

S41. Fernandes, M.H.M.D.R., FernandesJunior, J.D.S., Adams, J.M., Lee, M., Reis, R.A., Tedeschi, L.O., 2024. Using sentinel-2 satellite images and machine learning algorithms to predict tropical pasture forage mass, crude protein, and fiber content. Scientific Reports 14, 8704. https://doi.org/10.1038/s41598-024-59160-x

S42. Foschi, L., Pennino, D., Gislon, G., Grossi, G., Mantino, A., Barbanera, M., Marconi, M., Cesarini, F., Rossi, C., Vitali, A., Sandrucci, A., Zucali, M., Bava, L., Finocchi, M., Mele, M., Lacetera, N., 2025. Development of a machine learning tool for the enhancement of carbon footprint prediction for cattle milk production. The International Journal of Life Cycle Assessment 30, 3161–3174. https://doi.org/10.1007/s11367-025-02521-z

S43. Gao, Z., Zhu, Q., Tao, H., Jiao, Y., 2023. Grassland Health in Xilin Gol League from the Perspective of Machine Learning—Analysis of Grazing Intensity on Grassland Sustainability. Sustainability 15, 3398. https://doi.org/10.3390/su15043398

S44. Guarnido-Lopez, P., Devant, M., Llonch, L., Marti, S., Benaouda, M., 2023. Multiphase diets may improve feed efficiency in fattening crossbreed Holstein bulls: a retrospective simulation of the economic and environmental impact. animal 17, 101030. https://doi.org/10.1016/j.animal.2023.101030

S46. Hamidi, D., Hütt, C., Komainda, M., Grinnell, N.A., Horn, J., Riesch, F., Hamidi, M., Traulsen, I., Isselstein, J., 2023. Grid grazing: A case study on the potential of combining virtual fencing and remote sensing for innovative grazing management on a grid base. Livestock Science 278, 105373. https://doi.org/10.1016/j.livsci.2023.105373

S47. Handan-Nader, C., Ho, D.E., 2019. Deep learning to map concentrated animal feeding operations. Nature Sustainability 2, 298–306. https://doi.org/10.1038/s41893-019-0246-x

S48. Hansen, B.G., 2020. Exploring the relationship between CO2 emissions from on-farm use of diesel fuel and costs associated with forage harvesting – A win-to-win situation. Acta Agriculturae Scandinavica, Section A — Animal Science 69, 210–219. https://doi.org/10.1080/09064702.2020.1804993

S49. Hansen, K.N.J., Steinshamn, H., Hansen, S., Koesling, M., Dalgaard, T., Hansen, B.G., 2025. Comparing different statistical models for predicting greenhouse gas emissions, energy-, and nitrogen intensity. Computers and Electronics in Agriculture 234, 110209. https://doi.org/10.1016/j.compag.2025.110209

S50. Hempel, S., Adolphs, J., Landwehr, N., Janke, D., Amon, T., 2020a. How the Selection of Training Data and Modeling Approach Affects the Estimation of Ammonia Emissions from a Naturally Ventilated Dairy Barn—Classical Statistics versus Machine Learning. Sustainability 12, 1030. https://doi.org/10.3390/su12031030

S51. Hempel, S., Adolphs, J., Landwehr, N., Willink, D., Janke, D., Amon, T., 2020b. Supervised Machine Learning to Assess Methane Emissions of a Dairy Building with Natural Ventilation. Applied Sciences 10, 6938. https://doi.org/10.3390/app10196938

S52. Hosseinzadeh-Bandbafha, H., Safarzadeh, D., Ahmadi, E., 2015. Modeling Output Energy and Green House Gas Emissions of Dairy Farms using Neural Networks. Biological Forum – An International Journal 7, 743–752.

S53. Hou, L., Xin, X., Sun, H., Tao, Y., Chen, J., Yan, R., Zhang, X., Shen, B., Altome, A.I.A., Hamed, Y.M.Z., Wang, X., Nurlan, S., Adilbek, N., Balzhan, A., Kussainova, M., Amarjargal, A., Fang, W., Pulatov, A., 2023. Grazing-induced cattle behaviour modulates the secondary production in a Eurasian steppe ecosystem. Science of the Total Environment 889, 164191. https://doi.org/10.1016/j.scitotenv.2023.164191

S54. Irisarri, J.G.N., Texeira, M.A., Harris, P., Pembleton, K.G., 2025. Satellite-based monitoring of forage quality in grasslands of the United Kingdom using sentinel-2 data and random forest regression. Frontiers in Veterinary Science 12, 1678123. https://doi.org/10.3389/fvets.2025.1678123

S55. Jakimow, B., Griffiths, P., Van Der Linden, S., Hostert, P., 2018. Mapping pasture management in the Brazilian Amazon from dense Landsat time series. Remote Sensing of Environment 205, 453–468. https://doi.org/10.1016/j.rse.2017.10.009

S56. Jaman, K., Idrus, S., Wahab, A.M.A., Harun, R., Daud, N.N.N., Ahsan, A., Shams, S., Uddin, Md.A., 2023. Influence of Molasses Residue on Treatment of Cow Manure in an Anaerobic Filter with Perforated Weed Membrane and a Conventional Reactor:

Variations of Organic Loading and a Machine Learning Application. Membranes 13, 159. https://doi.org/10.3390/membranes13020159

S57. Jeong, S., Fischer, M.L., Breunig, H., Marklein, A.R., Hopkins, F.M., Biraud, S.C., 2022. Artificial Intelligence Approach for Estimating Dairy Methane Emissions. Environmental Science & Technology 56, 4849–4858. https://doi.org/10.1021/acs.est.1c08802

S58. Ji, S., Gonchigsumlaa, G., Damdindorj, S., Tseren, T., Sharavjamts, D., Otgondemberel, A., Gurjav, E.-A., Puntsagsuren, M., Tsabatshir, B., Gungaa, T., Batbold, N., Drees, L., Ganbayar, B., Orosoo, D., Lkhamsuren, B., Ganbat, B., Damdinsuren, M., Gombosuren, G., Dashpurev, B., Phan, T.N., Dejid, N., Müller, T., Lehnert, L., 2025. Can vegetation breakpoints in Eastern Mongolia rangeland be detected using Sentinel-1 coherence time series data? GIScience & Remote Sensing 62, 2540222. https://doi.org/10.1080/15481603.2025.2540222

S59. Jia, Y., Zhang, Z., He, Z., Zhu, P., Zhang, Y., Sun, T., 2021. Production Efficiency Prediction of Pig Breeding Industry by Optimized LSTM Computer Algorithm under Environmental Regulation. Scientific Programming 2021, 1–12. https://doi.org/10.1155/2021/3074167

S60. Jobarteh, B., Neethirajan, S., 2025. Leveraging satellite data for greenhouse gas mitigation in Canadian poultry farming. Smart Agricultural Technology 10, 100704. https://doi.org/10.1016/j.atech.2024.100704

S61. Kolluru, V., John, R., Saraf, S., Chen, J., Hankerson, B., Robinson, S., Kussainova, M., Jain, K., 2023. Gridded livestock density database and spatial trends for Kazakhstan. Scientific Data 10, 839. https://doi.org/10.1038/s41597-023-02736-5

S62. Kuchler, P.C., Bégué, A., Simões, M., Gaetano, R., Arvor, D., Ferraz, R.P.D., 2020. Assessing the optimal preprocessing steps of MODIS time series to map cropping systems in Mato Grosso, Brazil. International Journal of Applied Earth Observation and Geoinformation 92, 102150. https://doi.org/10.1016/j.jag.2020.102150

S63. Kuchler, P.C., Simões, M., Ferraz, R., Arvor, D., De Almeida Machado, P.L.O., Rosa, M., Gaetano, R., Bégué, A., 2022. Monitoring Complex Integrated Crop–Livestock Systems at Regional Scale in Brazil: A Big Earth Observation Data Approach. Remote Sensing 14, 1648. https://doi.org/10.3390/rs14071648

S64. Küçüktopcu, E., Cemek, B., 2021. Comparison of neuro-fuzzy and neural networks techniques for estimating ammonia concentration in poultry farms. Journal of Environmental Chemical Engineering 9, 105699. https://doi.org/10.1016/j.jece.2021.105699

S65. Lei, G., Li, A., Zhang, Z., Bian, J., Hu, G., Wang, C., Nan, X., Wang, J., Tan, J., Liao, X., 2020. The Quantitative Estimation of Grazing Intensity on the Zoige Plateau Based on the Space-Air-Ground Integrated Monitoring Technology. Remote Sensing 12, 1399. https://doi.org/10.3390/rs12091399

S66. Lemphane, N.J., Kotze, B., Kuriakose, R.B., 2024. Developing a Digital Twin Model for Improved Pasture Management at Sheep Farm to Mitigate the Impact of Climate Change. International Journal of Advanced Computer Science and Applications 15, 251. https://doi.org/10.14569/IJACSA.2024.0150627

S67. Li, H., Ke, L., Chen, Z., Feng, G., Xia, D., Wang, Y., Zheng, Y., Li, Q., 2016. Estimating the Fates of C and N in Various Anaerobic Codigestions of Manure and Lignocellulosic

Biomass Based on Artificial Neural Networks. Energy & Fuels 30, 9490–9501. https://doi.org/10.1021/acs.energyfuels.6b01883

S68. Li, M., Wang, J., Li, K., Liu, Y., Ochir, A., Davaasuren, D., 2024. Assessment of grazing livestock balance on the Eastern Mongolian Plateau based on remote sensing monitoring and grassland carrying capacity evaluation. Scientific Reports 14, 32151. https://doi.org/10.1038/s41598-024-84215-4

S69. Li, Z., Roche, L.M., Ostoja, S., Jin, Y., 2025. Long-term mean climate and seasonal variability drive spatial patterns of forage production fluctuation trends across California annual grasslands. Environmental Research: Ecology 4, 035008. https://doi.org/10.1088/2752-664X/adf6c1

S70. Lima, M.L.D.F., Souza, S.M.F.D., Sá, I.V.D., Santana, O.A., 2023. Deep learning with aerial surveys for extensive livestock hotspot recognition in the Brazilian Semi-arid Region. Ciência e Agrotecnologia 47, e010922. https://doi.org/10.1590/1413-7054202347010922

S71. Liu, H., Wang, X., Tana, Xie, T., Hurichabilige, Zhen, Q., Li, W., 2025. Predicting Winter Ammonia and Methane Emissions from a Naturally Ventilated Dairy Barn in a Cold Region Using an Adaptive Neural Fuzzy Inference System. Agriculture 15, 1560. https://doi.org/10.3390/agriculture15141560

S72. Liu, Y., Peñuelas-Rivas, C.G., Tenorio-Borroto, E., Rivas-Guevara, M., Buendía-Rodríguez, G., Tan, Z., González-Díaz, H., 2016. Chemometric approach to fatty acid metabolism-distribution networks and methane production in ruminal microbiome.

Chemometrics and Intelligent Laboratory Systems 151, 1–8. https://doi.org/10.1016/j.chemolab.2015.11.008

S73. Liu, Y., Wang, J., Yang, K., Ochir, A., 2024. Mapping livestock density distribution in the Selenge River Basin of Mongolia using random forest. Scientific Reports 14, 11090. https://doi.org/10.1038/s41598-024-61959-7

S74. Lo, A., Diouf, A.A., Diedhiou, I., Bassène, C.D.E., Leroux, L., Tagesson, T., Fensholt, R., Hiernaux, P., Mottet, A., Taugourdeau, S., Ngom, D., Touré, I., Ndao, B., Sarr, M.A., 2022. Dry season forage assessment across senegalese rangelands using earth observation data. Frontiers in Environmental Science 10, 931299. https://doi.org/10.3389/fenvs.2022.931299

S75. Lo, A., Diouf, A.A., Leroux, L., Tagesson, T., Fensholt, R., Mottet, A., Bonnal, L., Diedhiou, I., 2024. Remote Sensing-Based Assessment of Dry-Season Forage Quality for Improved Rangeland Management in Sahelian Ecosystems. Rangeland Ecology & Management 96, 94–104. https://doi.org/10.1016/j.rama.2024.05.009

S76. Long, F., Xu, M., Liao, W., Liu, H., 2025. Machine learning for predicting and optimizing the performance of a commercial-scale anaerobic digester with diverse feedstocks and operating conditions. Bioresource Technology 435, 132940. https://doi.org/10.1016/j.biortech.2025.132940

S78. Lush, L., Wilson, R.P., Holton, M.D., Hopkins, P., Marsden, K.A., Chadwick, D.R., King, A.J., 2018. Classification of sheep urination events using accelerometers to aid improved measurements of livestock contributions to nitrous oxide emissions. Computers and Electronics in Agriculture 150, 170–177. https://doi.org/10.1016/j.compag.2018.04.018

S79. Lyu, F., Wu, J., Yu, Z., Di, H.J., Pan, Y., 2024. Mapping ecological resource use and supply-consumption difference in China from 2000 to 2020. Resources, Conservation and Recycling 211, 107879. https://doi.org/10.1016/j.resconrec.2024.107879

S80. Malek, Ž., Romanchuk, Z., Yaschun, O., Jones, G., Petersen, J.-E., Fritz, S., See, L., 2024. Improving the representation of cattle grazing patterns in the European Union. Environmental Research Letters 19, 114077. https://doi.org/10.1088/1748-9326/ad858a

S81. Marin, N.A., Barboza, E., López, R.S., Vásquez, H.V., Gómez Fernández, D., Terrones Murga, R.E., Rojas Briceño, N.B., Oliva-Cruz, M., Gamarra Torres, O.A., Silva López, J.O., Cayo, E.T., 2022. Spatiotemporal Dynamics of Grasslands Using Landsat Data in Livestock Micro-Watersheds in Amazonas (NW Peru). Land 11, 674. https://doi.org/10.3390/land11050674

S82. Martinsson, E., Hansson, H., Mittenzwei, K., Storm, H., 2024. Evaluating environmental effects of adopting automatic milking systems on Norwegian dairy farms. European Review of Agricultural Economics 51, 128–156. https://doi.org/10.1093/erae/jbad041

S83. McParland, S., Frizzarin, M., Lahart, B., Kennedy, M., Shalloo, L., Egan, M., Starsmore, K., Berry, D.P., 2024. Predicting methane emissions of individual grazing dairy cows from spectral analyses of their milk samples. Journal of Dairy Science 107, 978–991. https://doi.org/10.3168/jds.2023-23577

S84. Meng, N., Wang, L., Qi, W., Dai, X., Li, Z., Yang, Y., Li, R., Ma, J., Zheng, H., 2023. A high-resolution gridded grazing dataset of grassland ecosystem on the Qinghai-Tibet Plateau in 1982-2015. Scientific Data 10, 68. https://doi.org/10.1038/s41597-023-01970-1

S85. Monteiro, H.F., Figueiredo, C.C., Mion, B., Santos, J.E.P., Bisinotto, R.S., Peñagaricano, F., Ribeiro, E.S., Marinho, M.N., Zimpel, R., Da Silva, A.C., Oyebade, A., Lobo, R.R., Coelho Jr, W.M., Peixoto, P.M.G., Ugarte Marin, M.B., Umaña-Sedó, S.G., Rojas, T.D.G., Elvir-Hernandez, M., Schenkel, F.S., Weimer, B.C., Brown, C.T., Kebreab, E., Lima, F.S., 2024. An artificial intelligence approach of feature engineering and ensemble methods depicts the rumen microbiome contribution to feed efficiency in dairy cows. Animal Microbiome 6, 5. https://doi.org/10.1186/s42523-024-00289-5

S86. Mota, L.F.M., Arikawa, L.M., Santos, S.W.B., Fernandes Júnior, G.A., Alves, A.A.C., Rosa, G.J.M., Mercadante, M.E.Z., Cyrillo, J.N.S.G., Carvalheiro, R., Albuquerque, L.G., 2024. Benchmarking machine learning and parametric methods for genomic prediction of feed efficiency-related traits in Nellore cattle. Scientific Reports 14, 6404. https://doi.org/10.1038/s41598-024-57234-4

S87. Nakashima, N., Kawamura, K., Hirata, M., Muneoka, T., Kimura, M., Shinkai, M., 2025. Effects of Nocturnal Grazing Behavior and Stock Density on Cattle Dung in Sloped Rotational Grazing. Rangeland Ecology & Management 99, 18–25. https://doi.org/10.1016/j.rama.2024.11.006

S88. Naturinda, E., Kemigyisha, F., Gidudu, A., Kabenge, I., Omia, E., Aboth, J., 2025. Quantification of greenhouse gas emissions from livestock using remote sensing & artificial intelligence. Artificial Intelligence in Geosciences 6, 100147. https://doi.org/10.1016/j.aiig.2025.100147

S90. Ndlovu, N., Styles, D., Kafunah, J., Narayanan, M., Quiroz, L.F., Gondalia, N., Brychkova, G., McKeown, P.C., Spillane, C., 2025. Optimizing selective breeding of livestock and

forage crops to reduce the environmental impacts of grass-based dairy production by combining life cycle assessment and machine learning. Science of the Total Environment 1003, 180615. https://doi.org/10.1016/j.scitotenv.2025.180615

S91. Negussie, E., González-Recio, O., Battagin, M., Bayat, A.-R., Boland, T., De Haas, Y., Garcia-Rodriguez, A., Garnsworthy, P.C., Gengler, N., Kreuzer, M., Kuhla, B., Lassen, J., Peiren, N., Pszczola, M., Schwarm, A., Soyeurt, H., Vanlierde, A., Yan, T., Biscarini, F., 2022. Integrating heterogeneous across-country data for proxy-based random forest prediction of enteric methane in dairy cattle. Journal of Dairy Science 105, 5124–5140. https://doi.org/10.3168/jds.2021-20158

S92. Nsabiyeze, A., Zhang, M., Li, J., Jiang, D., Zhao, Q., Yan, W., Zhang, X., 2025. Harnessing predictive modeling for optimized energy efficiency and carbon footprint reduction in sheep farming. Journal of Cleaner Production 534, 147104. https://doi.org/10.1016/j.jclepro.2025.147104

S93. Obileke, K., Tangwe, S., Makaka, G., Mukumba, P., 2024. Comparison of prediction of biogas yield in a batch mode underground fixed dome digester with cow dung. Biomass Conversion and Biorefinery 14, 26427–26442. https://doi.org/10.1007/s13399-023-04593-z

S94. Ocholla, I.A., Heiskanen, J., Karanja, F., Boitt, M., Pellikka, P., 2025. Towards monitoring livestock using satellite imagery: Transferability of object detection and segmentation models in Kenyan rangelands. ISPRS Open Journal of Photogrammetry and Remote Sensing 18, 100106. https://doi.org/10.1016/j.ophoto.2025.100106

S95. Ogungbuyi, M.G., Guerschman, J.P., Fischer, A.M., Crabbe, R.A., Mohammed, C., Scarth, P., Tickle, P., Whitehead, J., Harrison, M.T., 2023. Enabling Regenerative Agriculture Using Remote Sensing and Machine Learning. Land 12, 1142. https://doi.org/10.3390/land12061142

S96. Ogungbuyi, M.G., Mohammed, C., Fischer, A.M., Turner, D., Whitehead, J., Harrison, M.T., 2024. Integration of Drone and Satellite Imagery Improves Agricultural Management Agility. Remote Sensing 16, 4688. https://doi.org/10.3390/rs16244688

S97. Okwu, M.O., Samuel, O.D., Otanocha, O.B., Tartibu, L.K., Omoregbee, H.O., Mbachu, V.M., 2023. Development of ternary models for prediction of biogas yield in a novel modular biodigester: a case of fuzzy Mamdani model (FMM), artificial neural network (ANN), and response surface methodology (RSM). Biomass Conversion and Biorefinery 13, 917–926. https://doi.org/10.1007/s13399-020-01113-1

S98. Olatunji, K.O., Madyira, D.M., Adeleke, O., 2023. Optimizing anaerobic co-digestion of Xyris capensis and duck waste using neuro-fuzzy model and response surface methodology. Fuel 354, 129334. https://doi.org/10.1016/j.fuel.2023.129334

S99. Parente, L., Mesquita, V., Miziara, F., Baumann, L., Ferreira, L., 2019. Assessing the pasturelands and livestock dynamics in Brazil, from 1985 to 2017: A novel approach based on high spatial resolution imagery and Google Earth Engine cloud computing. Remote Sensing of Environment 232, 111301. https://doi.org/10.1016/j.rse.2019.111301

S100. Park, J., Jo, G., Jung, M., Oh, Y., 2023. Comparative Analysis of Neural Network Models for Predicting Ammonia Concentrations in a Mechanically Ventilated Sow Gestation Facility in Korea. Atmosphere 14, 1248. https://doi.org/10.3390/atmos14081248

S101. Park, M., Cho, S., Jeon, E., Choi, N.-J., 2024. Development of Volatile Fatty Acid and Methane Production Prediction Model Using Ruminant Nutrition Comparison of Algorithms. Fermentation 10, 410. https://doi.org/10.3390/fermentation10080410

S102. Paz-Kagan, T., Alexandroff, V., Ungar, E.D., 2023. Detection of goat herding impact on vegetation cover change using multi-season, multi-herd tracking and satellite imagery. Science of the Total Environment 895, 164830. https://doi.org/10.1016/j.scitotenv.2023.164830

S103. Pei, Z., Liu, S., Jing, Z., Zhang, Y., Wang, J., Liu, J., Wang, Y., Guo, W., Li, Y., Feng, L., Zhou, H., Li, G., Han, Y., Liu, D., Pan, J., 2022. Understanding of the interrelationship between methane production and microorganisms in high-solid anaerobic co-digestion using microbial analysis and machine learning. Journal of Cleaner Production 373, 133848. https://doi.org/10.1016/j.jclepro.2022.133848

S104. Pence, I., Kumaş, K., Siseci Cesmeli, M., Akyüz, A., 2023. Modeling of energy and emissions from animal manure using machine learning methods: the case of the Western Mediterranean Region, Turkey. Environmental Science and Pollution Research 30, 22631–22652. https://doi.org/10.1007/s11356-022-23780-5

S105. Pence, I., Kumaş, K., Siseci Cesmeli, M., Akyüz, A., 2024a. Animal-based CO2, CH4, and N2O emissions analysis: Machine learning predictions by agricultural regions and climate dynamics in varied scenarios. Computers and Electronics in Agriculture 226, 109423. https://doi.org/10.1016/j.compag.2024.109423

S106. Pence, I., Kumaş, K., Siseci Cesmeli, M., Akyüz, A., 2024b. Future prediction of biogas potential and CH4 emission with boosting algorithms: the case of cattle, small ruminant,

and poultry manure from Turkey. Environmental Science and Pollution Research 31, 24461–24479. https://doi.org/10.1007/s11356-024-32666-7

S107. Peng, C., May, A., Abeel, T., 2023. Unveiling microbial biomarkers of ruminant methane emission through machine learning. Frontiers in Microbiology 14, 1308363. https://doi.org/10.3389/fmicb.2023.1308363

S108. Peng, E.K., Abdul Malek, M., Shamsuddin, S.M., 2019. Artificial Intelligence Projection Model for Methane Emission from Livestock in Sarawak. Sains Malaysiana 48, 1325–1332. https://doi.org/10.17576/jsm-2019-4807-02

S109. Pereira, P.R.M., Bolfe, É.L., Costa, F.W.D., Rodrigues, T.C.S., Farias Filho, M.S., Braga, E.V., 2025. Geospatial Scenario Modeling with Cellular Automata: Land Use and Cover Change in Southern Maranhão, Brazilian Savanna (2020–2030). Geomatics 5, 65. https://doi.org/10.3390/geomatics5040065

S110. Perumal, A., Mazumdar, C., Srinatha, T.N., Rath, S., Likhitha, S., 2024. Mitigating Climate Impact: A Machine Learning Approach to Forecast Methane Emissions from Indian Livestock. IJAE 79, 610–620. https://doi.org/10.63040/25827510.2024.03.020

S111. Qi, Y., Song, B., Chai, M., Zhu, J., Deng, Y., Luo, X., 2026. Research on Carbon Emission Forecastingin the Agricultural and Livestock Industry- A Case Study of Sichuan Province, China. Polish Journal of Environmental Studies 35, 2233–2246. https://doi.org/10.15244/pjoes/201938

S112. Ramirez-Agudelo, J.-F., Bedoya-Mazo, S., Moreno-Pulgarín, L.-F., Guarin, J.-F., 2025. Development of an electronic device for automatic and individualized monitoring of

enteric methane emissions in dairy cows. Revista Colombiana de Ciencias Pecuarias 38, e358533. https://doi.org/10.17533/udea.rccp.e358533

S113. Reza, A., Chen, L., Kruger, K., 2022. Microwave irradiated ammonia nitrogen removal from anaerobically digested liquid dairy manure: A response surface methodology and artificial neural network-based optimization and modeling. Journal of Environmental Chemical Engineering 10, 108279. https://doi.org/10.1016/j.jece.2022.108279

S114. Rico-Contreras, J.O., Aguilar-Lasserre, A.A., Méndez-Contreras, J.M., López-Andrés, J.J., Cid-Chama, G., 2017. Moisture content prediction in poultry litter using artificial intelligence techniques and Monte Carlo simulation to determine the economic yield from energy use. Journal of Environmental Management 202, 254–267. https://doi.org/10.1016/j.jenvman.2017.07.034

S115. Rodriguez, M.R., Besteiro, R., Ortega, J.A., Fernandez, M.D., Arango, T., 2022. Evolution and Neural Network Prediction of CO2 Emissions in Weaned Piglet Farms. Sensors 22, 2910. https://doi.org/10.3390/s22082910

S116. Rouegui, M., Bellabair, H., El Asli, A., Amar, A., Zoerner, W., Rachidi, F., Lghoul, R., 2025. Bio-Methanization of Sheep Manure and Beet Waste in the Meknes–Fès Region, Morocco: Effects of Pretreatment and Machine Learning Applications for Biochemical Methane Potential Prediction. Recycling 10, 213. https://doi.org/10.3390/recycling10060213

S117. Saha, A., Rashid, B., Liu, T., Miralha, L., Muenich, R.L., 2025. Machine learning-based identification of animal feeding operations in the United States on a parcel-scale. Science of the Total Environment 960, 178312. https://doi.org/10.1016/j.scitotenv.2024.178312

S118. Sahraei, A., Knob, D., Lambertz, C., Gattinger, A., Breuer, L., 2025. Modeling enteric methane emission from dairy cows using deep learning approach. Science of the Total Environment 984, 179713. https://doi.org/10.1016/j.scitotenv.2025.179713

S119. Şenol, H., 2025. Estimation of biogas potential from poultry manure to 2040 in Türkiye using time series and the artificial neural network (ANN). Renewable and Sustainable Energy Reviews 207, 114976. https://doi.org/10.1016/j.rser.2024.114976

S120. Şenol, H., Ali Dereli, M., Özbilgin, F., 2021. Investigation of the distribution of bovine manure-based biomethane potential using an artificial neural network in Turkey to 2030. Renewable and Sustainable Energy Reviews 149, 111338. https://doi.org/10.1016/j.rser.2021.111338

S121. Şenol, H., Çolak, E., Elibol, E.A., Hassaan, M.A., El Nemr, A., 2024a. Optimisation of biochar dose in anaerobic co-digestion of green algae and cattle manure using artificial neural networks and response surface methodology. Chemical Engineering Journal 493, 152750. https://doi.org/10.1016/j.cej.2024.152750

S122. Şenol, H., Çolak, E., Oda, V., 2024b. Forecasting of biogas potential using artificial neural networks and time series models for Türkiye to 2035. Energy 302, 131949. https://doi.org/10.1016/j.energy.2024.131949

S123. Serpa-Imbett, C.M., Gómez-Palencia, E.L., Medina-Herrera, D.A., Mejía-Luquez, J.A., Martínez, R.R., Burgos-Paz, W.O., Aguayo-Ulloa, L.A., 2025. In-Field Forage Biomass and Quality Prediction Using Image and VIS-NIR Proximal Sensing with Machine Learning and Covariance-Based Strategies for Livestock Management in Silvopastoral Systems. AgriEngineering 7, 111. https://doi.org/10.3390/agriengineering7040111

S124. Shadpour, S., Chud, T.C.S., Hailemariam, D., Plastow, G., Oliveira, H.R., Stothard, P., Lassen, J., Miglior, F., Baes, C.F., Tulpan, D., Schenkel, F.S., 2022. Predicting methane emission in Canadian Holstein dairy cattle using milk mid-infrared reflectance spectroscopy and other commonly available predictors via artificial neural networks. Journal of Dairy Science 105, 8272–8285. https://doi.org/10.3168/jds.2021-21176

S125. Shea, K., Schaffer-Smith, D., Muenich, R.L., 2022. Using remote sensing to identify liquid manure applications in eastern North Carolina. Journal of Environmental Management 317, 115334. https://doi.org/10.1016/j.jenvman.2022.115334

S126. Shetty, B.D., Amaly, N., Weimer, B.C., Pandey, P., 2023. Predicting Escherichia coli levels in manure using machine learning in weeping wall and mechanical liquid solid separation systems. Frontiers in Artificial Intelligence 5, 921924. https://doi.org/10.3389/frai.2022.921924

S127. Shi, R., Lou, W., Ducro, B., Van Der Linden, A., Mulder, H.A., Oosting, S.J., Li, S., Wang, Y., 2023. Predicting nitrogen use efficiency, nitrogen loss and dry matter intake of individual dairy cows in late lactation by including mid-infrared spectra of milk samples. Journal of Animal Science and Biotechnology 14, 8. https://doi.org/10.1186/s40104-022-00802-3

S128. Shorten, P.R., Welten, B.G., 2021. Assessment of a non-invasive acoustic sensor for detecting cattle urination events. Biosystems Engineering 207, 177–187. https://doi.org/10.1016/j.biosystemseng.2021.05.003

S129. Silveira, R.M.F., Façanha, D.A.E., McManus, C.M., Ferreira, J., Silva, I.J.O.D., 2023. Machine intelligence applied to sustainability: A systematic methodological proposal to

identify sustainable animals. Journal of Cleaner Production 420, 138292. https://doi.org/10.1016/j.jclepro.2023.138292

S130. Simion, B., Copăcean, L., Cojocariu, L., 2025. Integrating open-source satellite data and artificial intelligence for grassland monitoring. A case study. Research Journal of Agricultural Science 57, 434–443. https://doi.org/10.59463/RJAS.2025.3.48

S131. Ahmad, A., Yadav, A.K., Singh, A., Singh, D.K., 2023. Optimisation of biogas yield from anaerobic co-digestion of dual waste for environmental sustainability: ANN, RSM and GA approach. International Journal of Oil, Gas and Coal Technology 33, 75–101. https://doi.org/10.1504/IJOGCT.2023.130377

S132. Soto, G.E., Wilcox, S.W., Clark, P.E., Fava, F.P., Jensen, N.D., Kahiu, N., Liao, C., Porter, B., Sun, Y., Barrett, C.B., 2024. Mapping rangeland health indicators in eastern Africa from 2000 to 2022. Earth System Science Data 16, 5375–5404. https://doi.org/10.5194/essd-16-5375-2024

S133. Su, J., Wang, H., Luo, D., Yang, Y., Ma, S., Wu, P., Wang, X., 2025a. Grazing intensity estimation in temperate typical grasslands of Inner Mongolia using machine learning models. Ecological Indicators 172, 113318. https://doi.org/10.1016/j.ecolind.2025.113318

S134. Su, R., Yang, Y., Chang, S., A, G., Yun, X., Song, X., Liu, A., 2025b. Quantifying Grazing Intensity from Aboveground Biomass Differences Using Satellite Data and Machine Learning. Agronomy 15, 2537. https://doi.org/10.3390/agronomy15112537

S136. Tonn, B., Raab, C., Isselstein, J., 2019. Sward patterns created by patch grazing are stable over more than a decade. Grass and Forage Science 74, 104–114. https://doi.org/10.1111/gfs.12389

S137. Tryhuba, A., Mudryk, K., Tryhuba, I., Kotsylovskyi, M., Sorokin, D., Bezaltychna, O., Pysz, P., Hutsol, T., 2025. Models for sustainable management of livestock waste based on neural network architectures. Scientific Reports 15, 28082. https://doi.org/10.1038/s41598-025-13150-9

S138. Tulbure, M.G., Caineta, J., Cox, B., Stehman, S.V., Ercumen, A., Witter, R., Emanuel, R., Powell, D.E., Burdette, K., White-Williamson, S., Tuberty, S., 2024. Earth Observation Data to Support Environmental Justice: Linking Non-Permitted Poultry Operations to Social Vulnerability Indices. GeoHealth 8, e2024GH001179. https://doi.org/10.1029/2024GH001179

S139. Valle, F., Anklam, K., Döpfer, D., 2025. Artificial Intelligence—Assisted Monitoring of Water Usage for Cooling Cows on a Dairy Farm. Animals 15, 3470. https://doi.org/10.3390/ani15233470

S140. Vaysi, A., Pashaki, S.G.A., Rohani, A., Khojastehpour, M., 2024. Variation and Spatial Distribution of Emissions from Livestock Manure Management in Iran: An Evaluation and Location Analysis. International Journal of Environmental Research 18, 104. https://doi.org/10.1007/s41742-024-00654-x

S141. Vendruscolo, E.C.G., Mesa, D., Rissi, D.V., Meyer, B.H., De Oliveira Pedrosa, F., De Souza, E.M., Cruz, L.M., 2020. Microbial communities network analysis of anaerobic

reactors fed with bovine and swine slurry. Science of the Total Environment 742, 140314. https://doi.org/10.1016/j.scitotenv.2020.140314

S142. Wallace, R.J., Sasson, G., Garnsworthy, P.C., Tapio, I., Gregson, E., Bani, P., Huhtanen, P., Bayat, A.R., Strozzi, F., Biscarini, F., Snelling, T.J., Saunders, N., Potterton, S.L., Craigon, J., Minuti, A., Trevisi, E., Callegari, M.L., Cappelli, F.P., Cabezas-Garcia, E.H., Vilkki, J., Pinares-Patino, C., Fliegerová, K.O., Mrázek, J., Sechovcová, H., Kopečný, J., Bonin, A., Boyer, F., Taberlet, P., Kokou, F., Halperin, E., Williams, J.L., Shingfield, K.J., Mizrahi, I., 2019. A heritable subset of the core rumen microbiome dictates dairy cow productivity and emissions. Science Advances 5, eaav8391. https://doi.org/10.1126/sciadv.aav8391

S143. Wang, D., Chen, L., Tang, G., Yu, J., Chen, J., Li, Z., Cao, Y., Lei, X., Deng, L., Wu, S., Guan, L.L., Yao, J., 2023a. Multi-omics revealed the long-term effect of ruminal keystone bacteria and the microbial metabolome on lactation performance in adult dairy goats. Microbiome 11, 215. https://doi.org/10.1186/s40168-023-01652-5

S144. Wang, W., Chen, X., Fang, D., Li, L., Yueda, A.G., Gan, J., Deng, X., Ma, X., Chen, Y., Shi, Y., He, F., Wu, C., Yi, Z., Fu, M., Yi, J., 2025. Partial Treponema spp. as candidate probiotics for rumen methane mitigation revealed by a module-based activity index. Frontiers in Veterinary Science 12, 1654829. https://doi.org/10.3389/fvets.2025.1654829

S145. Wang, X., Zhou, J., Jiang, R., Wang, Y., Zhang, Y., Wu, R., A, X., Du, H., Tian, J., Wei, X., Shen, W., 2024. Development of an Alternative In Vitro Rumen Fermentation Prediction Model. Animals 14, 289. https://doi.org/10.3390/ani14020289

S146. Wang, Y., Zhou, J., Wang, X., Yu, Q., Sun, Y., Li, Y., Zhang, Y., Shen, W., Wei, X., 2023b. Rumen Fermentation Parameters Prediction Model for Dairy Cows Using a Stacking Ensemble Learning Method. Animals 13, 678. https://doi.org/10.3390/ani13040678

S147. Wawrzyniak, A., Przybylak, A., Boniecki, P., Sujak, A., Zaborowicz, M., 2023a. Neural Modelling in the Study of the Relationship between Herd Structure, Amount of Manure and Slurry Produced, and Location of Herds in Poland. Agriculture 13, 1451. https://doi.org/10.3390/agriculture13071451

S148. Wawrzyniak, A., Przybylak, A., Sujak, A., Boniecki, P., 2023b. Neural Modelling in the Exploration of the Biomethane Potential from Cattle Manure: A Case Study on Herds Structure from Wielkopolskie, Podlaskie, and Mazowieckie Voivodeships in Poland. Sensors 23, 164. https://doi.org/10.3390/s23010164

S149. Werner, J.P.S., Belgiu, M., Bueno, I.T., Dos Reis, A.A., Toro, A.P.S.G.D., Antunes, J.F.G., Stein, A., Lamparelli, R.A.C., Magalhães, P.S.G., Coutinho, A.C., Esquerdo, J.C.D.M., Figueiredo, G.K.D.A., 2024. Mapping Integrated Crop–Livestock Systems Using Fused Sentinel-2 and PlanetScope Time Series and Deep Learning. Remote Sensing 16, 1421. https://doi.org/10.3390/rs16081421

S150. Wu, C., Liu, X., Pei, F., Liu, K., Cai, Yaotong, Xu, X., Zhuang, H., Cai, Yiling, Li, B., 2025a. Non-negligible large impact of potential forestation on livestock production in China. Geography and Sustainability 6, 100270. https://doi.org/10.1016/j.geosus.2025.100270

S151. Wu, T., Gao, P., Zhang, S., Cheng, T., Wang, Y., 2025b. Improving Accuracy of Gridded Livestock Mapping by Combining Trend Modeling and Residual Assignment. Land Degradation & Development 36, 1092–1105. https://doi.org/10.1002/ldr.5413

S152. Wu, X., Ren, Y., Wu, W., Yang, X., Yi, G., Zhou, S., Tang, K.H.D., Huang, L., Li, R., 2025c. Optimizing swine manure composting parameters with integrated CatBoost and XGBoost models: nitrogen loss mitigation and mechanism. Journal of Environmental Management 388, 125995. https://doi.org/10.1016/j.jenvman.2025.125995

S153. Xi, G., Ma, C., Ji, F., Huang, H., Zhang, H., Guo, Z., Zhang, X., Zhao, S., Xie, Y., 2024. Spatial and temporal dynamics of livestock grazing intensity in the Selinco region: Towards sustainable grassland management. Journal of Cleaner Production 473, 143541. https://doi.org/10.1016/j.jclepro.2024.143541

S154. Xie, Q., Ni, J.-Q., Li, E., Bao, J., Zheng, P., 2022. Sequential air pollution emission estimation using a hybrid deep learning model and health-related ventilation control in a pig building. Journal of Cleaner Production 371, 133714. https://doi.org/10.1016/j.jclepro.2022.133714

S155. Xing, R., Li, B., Dora, S., Whittaker, M., Mathie, J., 2025. Engineering-Oriented Explainable Machine Learning and Digital Twin Framework for Sustainable Dairy Production and Environmental Impact Optimisation. Algorithms 18, 670. https://doi.org/10.3390/a18100670

S156. Yang, D., Yang, Z., Wen, Q., Ma, L., Guo, J., Chen, A., Zhang, M., Xing, X., Yuan, Y., Lan, X., Yang, X., 2024. Dynamic monitoring of aboveground biomass in inner Mongolia grasslands over the past 23 Years using GEE and analysis of its driving forces.

Journal of Environmental Management 354, 120415. https://doi.org/10.1016/j.jenvman.2024.120415

S157. Ye, G., Yu, R., 2025. Spatiotemporal Mapping of Grazing Livestock Behaviours Using Machine Learning Algorithms. Sensors 25, 4561. https://doi.org/10.3390/s25154561

S158. Yelmen, B., 2019. The use of artificial neural networks in energy use modeling in broiler farms: A case study of Mersin Province in the Mediterranean region. Applied Ecology and Environmental Research 17, 13169–13183. https://doi.org/10.15666/aeer/1706_1316913183

S159. Yu, G., Li, Z., Dong, R., 2025. Comparative Prediction of Methane Production In Vitro Using Multiple Regression Model and Backpropagation Neural Network Based on Cornell Net Carbohydrate and Protein System. Veterinary Sciences 12, 1099. https://doi.org/10.3390/vetsci12111099

S160. Yusof, T.R.T., Man, H.C., Rahman, N.A.A., Hafid, H.S., 2014. Optimization of Methane Gas Production From Co-digestion of Food waste and Poultry Manure Using Artificial Neural Network and Response Surface Methodology. JAS 6, p27. https://doi.org/10.5539/jas.v6n7p27

S161. Zaki, M.T., Rowles, L.S., Hallowell, J., Orner, K.D., 2024. A data-driven framework to inform sustainable management of animal manure in rural agricultural regions using emerging resource recovery technologies. Cleaner Environmental Systems 13. https://doi.org/10.1016/j.cesys.2024.100188

S162. Zhang, B., Lin, S., Moraes, L., Firkins, J., Hristov, A.N., Kebreab, E., Janssen, P.H., Bannink, A., Bayat, A.R., Crompton, L.A., Dijkstra, J., Eugène, M.A., Kreuzer, M.,

McGee, M., Reynolds, C.K., Schwarm, A., Yáñez-Ruiz, D.R., Yu, Z., 2023. Methane prediction equations including genera of rumen bacteria as predictor variables improve prediction accuracy. Scientific Reports 13, 21305. https://doi.org/10.1038/s41598-023-48449-y

S163. Zhang, J., Wu, Z., Liu, Y., Hou, F., 2025. Tibetan sheep grazing and selenium addition promote soil carbon and nitrogen mineralization in alpine meadow. Applied Soil Ecology 206, 105886. https://doi.org/10.1016/j.apsoil.2025.105886

S164. Zhang, Q., Chen, L., Khan, H.S., Zhang, Z., Li, H., 2024. Spatiotemporal differentiation and trend prediction of carbon emissions in China's swine industry. Ecological Indicators 166, 112391. https://doi.org/10.1016/j.ecolind.2024.112391

S165. Zhang, X., Li, M., Wu, J., He, Y., Niu, B., 2022a. Alpine Grassland Aboveground Biomass and Theoretical Livestock Carrying Capacity on the Tibetan Plateau. Journal of Resources and Ecology 13, 129–141. https://doi.org/10.5814/j.issn.1674-764x.2022.01.015

S167. Zhou, H., Chung, S., Waqar, M.M., Zain Ul Abideen, M.I., Ahmad, A., Ilyas, M.A., Kim, H., Kim, S., 2025. Unsupervised Anomaly Detection with Continuous-Time Model for Pig Farm Environmental Data. Agriculture 15, 1419. https://doi.org/10.3390/agriculture15131419

S168. Zhou, J., Niu, J., Wu, N., Lu, T., 2024. Annual high-resolution grazing-intensity maps on the Qinghai–Tibet Plateau from 1990 to 2020. Earth System Science Data 16, 5171–5189. https://doi.org/10.5194/essd-16-5171-2024

S169. Zou, L., Zhao, X., Wu, K., Liang, C., Liu, J., Yang, H., Wang, C., Yang, B., Yin, F., Zhang, W., 2024. Multi-objective optimization for microbial electrolysis cell-assisted anaerobic digestion of swine manure. International Journal of Hydrogen Energy 77, 545–556. https://doi.org/10.1016/j.ijhydene.2024.06.166

S170. Zwick, M., Cardoso, J.A., Gutiérrez-Zapata, D.M., Cerón-Muñoz, M., Gutiérrez, J.F., Raab, C., Jonsson, N., Escobar, M., Roberts, K., Barrett, B., 2024. Pixels to pasture: Using machine learning and multispectral remote sensing to predict biomass and nutrient quality in tropical grasslands. Remote Sensing Applications: Society and Environment 36, 101282. https://doi.org/10.1016/j.rsase.2024.101282

S9. Capability flag definitions and sensitivity definitions

This section gives the definitions and derivation rules of the functional and capability flags (Table S16) and the two sensitivity definitions specified before coding (Table S22).

Table S16. *Functional decision support flags and capability flags: definitions, derivation rules, and counts (n = 165). Flags are non exclusive and were added alongside the unchanged canonical columns; rules are recorded in the workbook Codebook (sections 11 to 13).*

Flag or column	Definition and derivation rule	Source	Studies coded Yes, n (%)
F1 Names a decision	The study names a specific decision on a specific management or design lever taken by an identifiable decision maker at farm level (feeding, grazing, stocking, housing control, technology adoption), process level (operating conditions, feedstock ratios, pretreatment, or	Full text of each study; extracted fields U, AA, AE, AF, AG, AH, AI, AJ, AT	33 (20.0)

	configuration of a digester, composter, or manure treatment process), or policy or program level (which farms or areas to target, which pathway or program to adopt). The scale at which the decision was demonstrated (bench, pilot, commercial) is recorded separately and is not a criterion. A decision mentioned only as a general motivation (could support decision making) is No.		
F2 Compares alternatives	The AI or ML model, or an optimization or simulation driven directly by its outputs, evaluates two or more alternative settings of the lever prospectively (scenario analysis, optimization over a decision space, counterfactual for a decision not yet taken). Comparisons in which the model served only as a measurement instrument within an experimental or observational contrast, ex post evaluations of a decision already taken, and scenarios that vary exogenous drivers (climate, population, economic growth) rather than the lever are No.	Full text of each study; extracted fields U, AA, AE, AF, AG, AH, AI, AJ, AT	27 (16.4)
F3 Recommends or controls	The study states a preferred setting, a ranked or targeted list, a target value, or a control rule derived from the model output.	Full text of each study; extracted fields U, AA, AE, AF, AG, AH, AI, AJ, AT	24 (14.5)
F4 Accessible tool	A tool, software, web application, commercial product, or operational system embodying the model is stated to exist and to be usable by decision makers without retraining. Prototype or proof of concept systems and statements that a tool could be developed are No.	Full text of each study; extracted fields U, AA, AE, AF, AG, AH, AI, AJ, AT	6 (3.6)
F5 Evaluated in use	Use of the model output by or for decision makers in a real operational setting (farm, plant, agency) was evaluated and the outcomes of that use	Full text of each study; extracted fields U, AA, AE, AF, AG, AH, AI, AJ, AT	4 (2.4)

	reported. Validation experiments and simulated deployment scenarios are No.		
F6 Outcome measured	The effect of decisions taken with the model output was measured in operation (feed, water, emissions, or productivity recorded, not estimated or simulated).	Full text of each study; extracted fields U, AA, AE, AF, AG, AH, AI, AJ, AT	2 (1.2)
DS_functional_status	DS_functional_status: Yes if F1 = Yes and (F2 = Yes or F3 = Yes); No if F1 = No or (F2 = No and F3 = No); Unclear otherwise. DS_functional_core counts Unclear as No (headline); DS_functional_core_upper counts Unclear as Yes (sensitivity bound).	derived	Yes 26 (15.8); Unclear 4 (2.4); No 135
DS_functional_core	Status Yes (headline)	derived	26 (15.8)
DS_functional_core_upper	Status Yes or Unclear (upper bound)	derived	30 (18.2)
DS_decision_level	Farm; Process (plant, digester, composter, or treatment); Policy or program. Recorded for every study with F1 Yes or Unclear; scale of demonstration is recorded separately and is not a criterion	full text	Among status Yes: Farm 11; Process 11; Policy or program 4
Stage_tool_accessible; Stage_evaluated_in_use; Stage_outcome_measured	Status Yes and F4; status Yes and F5; status Yes and F6 (reported separately in place of the draft 4 full definition)	derived	3; 2; 2
DS_functional_full	Status Yes and (F4 Yes or F5 Yes); equals Definition C (Table S22)	derived	3 (1.8)
SensB_status (Definition B, broader)	Information based decision support: F1 = Yes and (B2a) the output is the model's own output or a quantity directly derived from it, (B2b) the output is in a form the named decision maker could act on for the F1 decision at its spatial and temporal unit (a value or map of the lever or of the quantity it controls, a threshold or alert tied to the decision, a ranking, or a time resolved estimate), and (B2c) the paper states in Methods,	Full text of the 7 candidates; DS Functional Coding AM to AQ	29 (17.6); Unclear 1; upper bound 30 (18.2)

	Results or Discussion how the output feeds that decision. Specified in writing on 24 October 2026 before any candidate study was opened; candidate set fixed in advance as the 7 studies with F1 = Yes and status other than Yes; B2a to B2c recorded separately before the status; Unclear counted as No.		
SensC_status (Definition C, stricter)	Implemented decision support: status Yes and (F4 = Yes or F5 = Yes). No new coding.	derived; DS Functional Coding AR	3 (1.8)
DS_any_capability	Any of F1 to F5 Yes	derived	36 (21.8)
VAL_external	Any independent external validation reported: a different farm, herd, region, trial, or year, or a dataset not used in training, including cases the canonical column collapsed to Multiple; grouped cross validation that leaves out a farm, herd, or treatment is counted under VAL_crossval	W, original W (Cleaning Log), Y, AH, AT	30 (18.2)
VAL_crossval	Canonical W = Cross-validation, or the original W text or notes describe cross validation, k fold, leave one out, nested, or grouped resampling	W, original W, AT	36 (21.8)
VAL_traintest	Canonical W = Train test split, or the original W text describes a train test, holdout, or percentage split	W, original W	88 (53.3)
VAL_none_reported	Canonical W = None reported	W	4 (2.4)
PURP_prediction	Raw purpose contains prediction, regression, or forecasting, or raw purpose is Multiple and the finding, novelty, domain, or outcome text describes prediction, forecasting, regression, or estimation	U, AH, AI, AE, AA	127 (77.0)
PURP_classification	Raw purpose contains classification, detection, anomaly, or clustering, or raw purpose is Multiple and the text describes	U, AH, AI, AE, AA	40 (24.2)

	classification, detection, segmentation, or clustering		
PURP_optimization	A search over a decision or design space is part of the methods (metaheuristic, multi objective, response surface, or model based optimum), whatever the canonical purpose; hyperparameter or weight tuning by an optimizer does not count	U, AE, AA, AH, AI, AT	17 (10.3)
PURP_decision_support	Decision support named in raw model purpose, raw application domain, or the extractor's purpose note (a label based flag, distinct from F1 to F6)	U, AE, AT	7 (4.2)
HYBRID_mechanistic_coupling	A learned model coupled to a mechanistic, process based, life cycle, or farm system model; ML combined only with statistics, metaheuristics, or another learner is No	R, S, AH, AI, AT	7 (4.2)

S10. Additional tables

This section collects the definitions of terms, the method and metric summaries, the corpus profile, the input structure cross tabulation, the sensitivity definitions and transportability tables that the main text cites, and the mapping of the SWiM reporting items (Table S25). All counts use the corpus of 165 studies.

Table S17. *Definitions of terms used throughout the review.*

Term	Definition
Artificial intelligence (AI)	Computational systems performing tasks that would otherwise require human judgment. Used in this literature largely interchangeably with machine learning.
Machine learning (ML)	Algorithms that infer a rule from examples rather than applying a rule specified in advance.
Feature (input, predictor)	A variable available to the model, such as a spectral band, ambient temperature or dry matter intake.
Target (output, label)	The quantity or category the model is trained to produce.

Term	Definition
Training	Adjustment of model parameters so that outputs match targets on data where both are known.
Regression	Prediction of a continuous quantity.
Classification	Assignment of a discrete category.
Clustering	Grouping of similar cases without supervision.
Random forest (RF)	An average over many decision trees, each fitted to a random subset of observations and features.
Decision tree	A sequence of binary splits on feature values terminating in a prediction.
Gradient boosting (XGBoost, LightGBM, CatBoost)	Models fitted sequentially, each trained on the residual error of its predecessors.
Support vector machine (SVM, SVR)	A method identifying the separating boundary with the widest margin between classes.
Artificial neural network (ANN, MLP)	Layers of simple units combining weighted inputs through nonlinear functions.
Deep learning	Neural networks with many layers, applied where inputs are not readily tabulated.
Convolutional neural network (CNN)	Deep architecture for image data, applying learnable filters across the image.
LSTM, RNN	Deep architectures for sequential data, maintaining internal state across time steps.
YOLO (You Only Look Once)	CNN family optimized for single pass detection of multiple objects, suited to video rate operation.
Ensemble	Several models combined so that individual errors partially cancel.
Hybrid model	Machine learning combined with a non ML component: a mechanistic model, an optimization algorithm or a conventional statistical method.
Mechanistic (process based) model	A model constructed from physical, chemical or biological relationships rather than fitted to data.
Surrogate model	A fast learned approximation of a slow mechanistic model, permitting many scenarios to be evaluated.
Fuzzy logic	Reasoning with graded rather than binary category membership.
Overfitting	Reproduction of training data without learning a generalizable relationship.
Train test split	Fitting on one portion of the data and evaluating on the held out remainder.
Cross validation	Repetition of the split across several partitions, with results averaged.
External validation	Evaluation of data from a different farm, region or time period.
SHAP values	A method attributing an individual prediction to its contributing input variables.
Pareto front	The set of solutions on which no objective can be improved without worsening another.

Term	Definition
Decision support system (DSS)	A tool assisting a user in choosing among management options, as distinct from one predicting an outcome.
Life cycle assessment (LCA)	Accounting of environmental impacts across a production chain.

Table S18. *Method families represented in the corpus (n = 165), with a worked example from an included study.*

Family	n (%)	Characteristic operation	Suited to	Corpus example
Traditional ML	67 (40.6)	Learns from tabular observations; dominated by random forests	Tabular data at moderate sample size	*Paz-Kagan et al. (2023)*: random forest on 4.2 M GPS coordinates and Landsat imagery explains 93% of variance in vegetation cover change under goat herding
Ensemble	34 (20.6)	Combines several models so errors partially cancel; gradient boosting fits sequentially	Tabular problems where maximum accuracy is required	*Hempel et al. (2020a)*: gradient boosting predicts dairy barn methane within approximately 10% across 27 sampling scenarios, outperforming regression, neural networks and SVM
Hybrid	33 (20.0)	Couples ML with a mechanistic model, an optimizer or a conventional method	Problems combining established process knowledge with large residual variation	*Zou et al. (2024)*: a neural network of a swine manure digester with NSGA-II optimization produces a Pareto front trading methane yield against energy efficiency, within 3% of experiment
Deep learning	28 (17.0)	Stacks many layers to construct representations from raw input	Images, video and sequences, where features cannot be hand specified	*Jeong et al. (2022)*: U-Net segmentation of 316,882 aerial tiles locates California dairies and estimates herd size (r = 0.96 against a human inspection inventory)
Fuzzy logic	3 (1.8)	Reasons with graded category membership	Problems specified in expert linguistic terms	*Rico-Contreras et al. (2017)*: Mamdani inference predicts poultry litter moisture from 13 management variables (R^2 = 0.93)

Table S19. *Performance metrics reported in the corpus: definition, interpretive limits and a corpus example.*

Metric	Range	Reports	Does not report	Corpus example

Metric	Range	Reports	Does not report	Corpus example
R^2	1 or less; negative out of sample when worse than the mean	Proportion of variance explained	Difficulty of the prediction problem	*Peng et al. (2023)*: $R^2 = 0.56$ for individual animal methane from rumen microbiome, a substantive result on a hard target; *Okwu et al. (2023)*: $R^2 = 1.0$ on 13 experimental runs
RMSE	0 upward, target units	Typical error, penalizing large deviations	Requires the magnitude of the target for interpretation	*Chasen et al. (2025)*: RMSE = 0.11 t acre^{-1}, approximately 4% of mean yield
MAE	0 upward, target units	Mean absolute error	Distribution of errors around the mean	*Correa-Luna et al. (2024)*: MAE reduced from 498 to 342 kg DM ha^{-1} with field calibration
MAPE	%	Mean error as a percentage of the true value	Unstable where true values approach zero	Şenol (*2025*): MAPE = 0.71% for national poultry manure biogas potential
Accuracy	0 to 1 or %	Proportion of cases classified correctly	Class balance; misleading where classes are unequal	Shea et al. (2022): 97% overall accuracy for detecting liquid manure applications from satellite imagery
Precision	0 to 1	Proportion of positive predictions that are correct	Cases missed	*Naturinda et al. (2025)*: precision = 0.97 for cattle detection from UAV imagery
Recall (sensitivity)	0 to 1	Proportion of true positives identified	False alarms	*Naturinda et al. (2025)*: recall = 0.829 for the same detections
F1	0 to 1	Harmonic mean of precision and recall	Relative cost of the two error types	*Ocholla et al. (2025)*: F1 declining from 0.85 in open grassland to 0.55 in dense bushland
mAP	0 to 1 or %	Joint detection and localization quality	Scene difficulty, which varies independently of the model	Naturinda et al. (2025): mAP = 80% for detecting livestock in remote sensing imagery
CCC	−1 to 1	Agreement with observation, including bias	Direction of any systematic bias	*Ellis et al. (2020)*: CCC = 0.917 for SVR against 0.42 to 0.70 for conventional equations
ROC AUC	0 to 1; 0.5 equals chance ranking	Ranking ability across all thresholds	Performance at any usable operating threshold	*Handan-Nader and Ho (2019)*: AUC 0.97 to 1.00 for detection of concentrated animal feeding operations

Table S20. Profile of the included corpus (n = 165). Publication period percentages use the full corpus denominator of 165; temporal figures in the main text use 164, excluding the 2026 indexed study.

Characteristic	Category	n	%
**Publication period**	2014 to 2018	12	7.3
	2019 to 2021	20	12.1
	2022 to 2023	45	27.3
	2024 to 2025	87	52.7
	2026 (partial year)	1	0.6
**Country of study**	China	39	23.6
	United States	17	10.3
	Brazil	12	7.3
	Türkiye	8	4.8
	South Korea	6	3.6
	Germany	5	3.0
	Canada	4	2.4
	Australia	4	2.4
	Remaining 35 countries and 7 other labels	70	42.4
**Primary species**	Cattle	73	44.2
	Mixed or multiple	45	27.3
	Pigs	14	8.5
	Sheep	12	7.3
	Poultry	11	6.7
	Other	7	4.2
	Goats	3	1.8
**Geographic scope**	Single farm	56	33.9
	Regional	50	30.3
	National	26	15.8
	International	11	6.7
	Multi farm	11	6.7
	Not specified	11	6.7
**Measurement level**	Farm	57	34.5
	Regional	50	30.3
	Animal	38	23.0

	Multiple	11	6.7
	National	5	3.0
	Herd	2	1.2
	Not specified	2	1.2
**Study design**	Modeling	78	47.3
	Hybrid (data collection and modeling)	56	33.9
	Experimental	24	14.5
	Observational	7	4.2
**Practical readiness**	Farm applicable	85	51.5
	Laboratory research only	69	41.8
	Already deployed	10	6.1
	Tested on farm	1	0.6

Table S21. *Method family by structure of input data (n = 165). Input structure was coded by hand for every study from the extracted input variable, data source and algorithm fields (Section S13).*

Input data structure	Traditional ML	Ensemble	Hybrid	Deep learning	Fuzzy logic	Total	Deep learning share (%)
Tabular measurements	38	18	22	5	3	86	5.8
Imagery or spectral	18	11	5	14	0	48	29.2
Sequential or temporal	3	2	4	7	0	16	43.8
Imagery and temporal	8	3	2	2	0	15	13.3
**Total**	**67**	**34**	**33**	**28**	**3**	**165**	**17.0**

Table S22. Sensitivity of delivered decision support to alternative definitions (n = 165). Definitions B and C were specified in writing on 24 October 2026, before being applied to any study (Section S2, amendment 7; derivation rules in Table S16). The candidate set for Definition B was fixed in advance as the seven studies with F1 = Yes and primary status other than Yes. Coding: Yes for Correa-Luna et al. (2024), Ogungbuyi et al. (2023) and Rouegui et al. (2025); Unclear for Martinsson et al. (2024); No for Fernandez et al.

(2023), Hamidi et al. (2023) and Hou et al. (2023). Definition C positives: Lima et al. (2023), Chen et al. (2025) and Chasen et al. (2025). Gaps are computed from proportions before rounding.

Quantity	Definition C (stricter: status Yes and F4 or F5)	Primary (F1 and F2 or F3)	Primary upper bound (Unclear as Yes)	Definition B (broader: F1 and documented decision relevant output)	Definition B upper bound (Unclear as Yes)
Studies positive, n (%)	3 (1.8%)	26 (15.8%)	30 (18.2%)	29 (17.6%)	30 (18.2%)
Gap below the explicit claim rate, 96 (58.2%)	56.4 pp	42.4 pp	40.0 pp	40.6 pp	40.0 pp
Gap below the inclusive claim rate, 155 (93.9%)	92.1 pp	78.2 pp	75.8 pp	76.4 pp	75.8 pp
Explicit claimants not meeting the definition	93 of 96 (96.9%)	77 of 96 (80.2%)	73 of 96 (76.0%)	74 of 96 (77.1%)	73 of 96 (76.0%)

Table S22 (continued). Definition B by application domain (n = 165).

Application domain	Total	Claim Yes	Primary Yes	Definition B Yes	Definition B Unclear	Claim Yes minus Definition B Yes
Grazing / pasture management	56	43	4	6	0	37
Manure / waste to energy	47	23	16	17	0	6
Enteric fermentation / methane	23	5	1	1	0	4
GHG inventory / LCA	8	3	0	0	0	3
Livestock building / facility emissions	7	3	1	1	0	2
Spatial / land use	7	6	0	0	0	6
Breeding / genetic selection	6	4	0	0	0	4
Feed optimization	4	3	1	1	0	2
Other	3	3	1	1	0	2
Energy use / efficiency	3	2	2	2	0	0
Decision support / planning	1	1	0	0	1	1
Total	165	96	26	29	1	67

Note. Claim exceeds the Definition B count in 10 of 11 domains; energy use and efficiency shows no difference under any definition. The one Unclear candidate (Martinsson et al., 2024) is in the decision support and planning domain.

Table S23. Stated transportability target by test outside the training context (n = 165). The stated target is the setting the authors say the model is meant for, coded from the abstract, discussion or conclusion and the stated limitations (Section S2, amendment 8). Test outside the training context follows the VAL_external flag and its basis (Table S16).

Stated target	Studies	Tested outside the training context, n (%)	Independent farm, herd, region or trial	Independent dataset	Later time period only	No test outside
Own setting	72	12 (16.7)	3	7	2	60
Named wider population	16	6 (37.5)	2	1	3	10
General claim	65	12 (18.5)	4	7	1	53
Not stated	12	0 (0.0)	0	0	0	12
Claim beyond own setting (Named wider population or General claim)	81	18 (22.2)	6	8	4	63
**Total**	**165**	**30 (18.2)**	**9**	**15**	**6**	**135**

Note. Coding rules: Own setting, the authors restrict the model to the studied farm, herd, digester, dataset or mapped area, or say it should not be extrapolated; Named wider population, the authors name a specific wider population or setting the model is meant to serve beyond the data used (a region, country, breed, species group or production system); General claim, the authors assert broad applicability without naming a population; Not stated, no statement about where the model applies. Of the 81 studies that claim applicability beyond their own setting, 18 (22.2%; Wilson 95% CI 14.5 to 32.4) report any test outside the training context and 6 (7.4%) a test on an independent farm, herd, region or trial. Under the first-version label, 3 of the 9 studies called broadly applicable and 27 of the 156 called context specific had such a test. A second coder re-coded the stated target for a random sample of 40 studies (24%) from the full texts, blind to the first coding, after writing decision rules that are kept with the workbook. The two codings agreed on 25 of 40 studies (62.5%; 95% CI 47.0 to 75.8; kappa 0.44) over the four categories and on 30 of 40 (75.0%; kappa 0.51) as to whether a study claims applicability beyond its own setting. In 9 of the 15 differences the first coder gave Own setting and the second a wider or general claim, so the first coding is the more conservative and the share of studies claiming applicability beyond their own setting is a lower bound. A first pass by the second coder, before the rules were written, agreed on 21 of 40 (kappa 0.32); both passes are held by the authors and available on request.

Table S24. Transportability by geographic scope of the data (n = 165).

Geographic scope	Studies	Own setting	Named wider	General claim	Not stated	Claim beyond own	of which tested	Any test outside

			population			setting	outside	
Single farm	56	20	3	26	7	29	4	7
Multi-farm	11	3	4	3	1	7	3	3
Regional	50	26	7	17	0	24	8	13
National	26	18	2	6	0	8	1	4
International	11	2	0	8	1	8	1	2
Not specified	11	3	0	5	3	5	1	1
**Total**	**165**	**72**	**16**	**65**	**12**	**81**	**18**	**30**

Note. Geographic scope is the extraction field of Table S11 (column L). Claim beyond own setting is Named wider population plus General claim; tested outside means any test on data from outside the training context (Table S23). National studies claim applicability beyond their data less often (8 of 26) because the data already cover the target, whereas most international studies make a general claim (8 of 11); single farm and regional studies make most of the claims that were not tested.

Table S25. Synthesis Without Meta-analysis (SWiM) reporting items: what each item asks for, where it is addressed in this review, and how it was applied or adapted.

SWiM item	What the guideline asks for	Where it is addressed	How it was applied or adapted
Item 1. Grouping studies for synthesis	Describe how the studies were grouped for synthesis and justify the groups.	Section 2.5 (canonical mapping) and Table S12 (value sets); Sections 3.4, 3.5 and 3.7; Tables 3, 4 and 5.	Each study was placed in exactly one canonical category for application domain (11 groups), species, measurement level, geographic scope and method family, using written mapping rules recorded in the cleaning log. For the central synthesis, studies were grouped by their claim of decision relevance (Yes, Partial, No) and by functional status (Yes, Unclear, No). The groups follow the extracted fields, not the results.
Item 2. Standardised metric and transformation methods	Describe the standardised metric and any transformation used for each outcome.	Section 2.8; Tables 2, 4 and 5; Table S19; Figure S8.	Adapted. Outcomes, units and algorithms were too heterogeneous for a common effect size, so no effect estimates were extracted or transformed. The standardised unit is the study: results are counts and percentages of the corpus (n = 165) with Wilson 95% confidence intervals. Reported performance metrics are described, not combined (Table S19, Figure S8).
Item 3. Synthesis methods	Describe and justify the synthesis method.	Sections 2.7, 2.8, 3.7 and 4.3; Tables 4, 5 and 7.	Adapted. Studies were coded against written definitions (the claim field; functional flags F1 to F6) and the codes were cross tabulated. The headline statistic is the paired difference between the share of studies that claim decision relevance and the share that deliver it, with a Wald interval for paired proportions; a risk ratio with Fisher exact test is used for mechanistic coupling by delivery. No vote

			counting by direction of effect and no combination of p values were used, because no effects were synthesised.
Item 4. Criteria used to prioritise results	State the criteria used to decide which results to summarise and synthesise.	Sections 2.7, 2.8, 3.7.1 and 3.7.6; Supplementary Section S2, amendment 7.	The explicit claim (Yes only) against the functional definition is the primary contrast; the inclusive claim (Yes or Partial) is secondary. Unclear studies count as No in the headline and as Yes in the upper bound. Two alternative definitions of delivery were written down and dated before they were applied (Table S22).
Item 5. Investigation of heterogeneity	Describe how variation in results across studies was explored.	Sections 3.7.2, 3.7.5 and 3.7.6; Tables 5, S6, S9 and S23; Figures 9 and 10.	Adapted. Instead of heterogeneity in effect sizes, the gap between claimed and delivered decision support was examined across pre specified groups (application domain, measurement level, practical readiness) and under alternative definitions and quality exclusions.
Item 6. Certainty of evidence	Describe how the certainty of the evidence was assessed.	Sections 2.6, 3.6 and 3.7.6; Tables S6 and S7.	A composite quality rating from three dimensions (data quality, model transparency, reproducibility), with a second blind rating of 40 studies and reported agreement; sensitivity analyses exclude High concern and Limited reproducibility studies. No GRADE type rating was made because the synthesis is descriptive and does not estimate an effect.
Item 7. Data presentation methods	Describe the graphical and tabular methods used to present the synthesis.	Tables 4 to 7; Figures 8 to 10; Tables S15 and S22 to S24.	Cross tabulations with counts, percentages and confidence intervals; bar charts of the difference by domain and of claim against readiness; the evidence table (Table S15) lists every study with its codes and marks the studies that contribute to each synthesis.
Item 8. Reporting results	For each synthesis, describe the findings and their certainty, state which studies contribute, and use language consistent with the question.	Sections 3.7.1 to 3.7.6; Table 6; Table S15 (asterisks).	Every result is reported as a count, denominator, percentage and interval. The 26 delivering studies are listed in Table 6 and marked in Table S15. Results are worded as what studies claim and what they deliver, not as effects of an intervention.
Item 9. Limitations of the synthesis	Report the limitations of the synthesis methods and of the groupings.	Section 4.5; Section 3.7.6; Supplementary Section S2, amendment 5.	The claim rate records author framing, not independent judgement; the functional definition was specified after data collection and is reported as exploratory; the hybrid category is heterogeneous; quality ratings involve judgement, and the second rating reproduced the data quality rating no better than chance; cross tabulations admit alternative explanations; no pooled estimate is possible.

Note. SWiM (Synthesis Without Meta-analysis; Campbell et al., 2020, BMJ 368, l6890) is a nine item reporting guideline for systematic reviews that synthesise results without pooling effect estimates. It was written for syntheses

of intervention effects. In this review the unit of synthesis is the study and its classification (claim of decision relevance; functional delivery), so items 2, 3 and 5 were adapted as described in the last column; the other items were applied as written. The completed PRISMA 2020 checklist accompanies the submission as a separate file (Section S3).

Outcome units differ in their association with deployment. Of 48 studies reporting spatial or per area intensities, 9 (18.8%) are laboratory research only and 7 describe deployed systems; of 14 reporting dimensionless efficiency ratios, 9 (64.3%) are laboratory research only and none is deployed. Studies reporting physical, mass based quantities occupy an intermediate position, with 19 of 41 (46.3%) reporting GHG mass or inventory units laboratory confined. The pattern is consistent with the interpretation that dimensionless ratios do not correspond to quantities that a manager allocates or controls, whereas physical quantities and mapped areas do. This observation is drawn on in Section 4.3 of the main text.

S11. Targeted search for earlier reviews

The statement about earlier reviews in Section 1.2 of the main text rests on a documented search. The search rule and the screening rule were written on 26 October 2026, before the search was run, and are reproduced in the screening workbook, which is available from the authors on request. OpenAlex was searched through its application programming interface on 26 October 2026 for works published from 2015 to 2026, in English, whose title contains an AI or ML term (machine learning, deep learning, artificial intelligence, computer vision, neural network), a livestock term (livestock, dairy, cattle, cow, pig, swine, poultry, broiler, sheep, goat, ruminant) and a review term (review, survey, overview, meta-analysis, state of the art). The search returned 164 records.

A record was included when it is a review (narrative, systematic, scoping, umbrella or tertiary) of the application of AI or ML methods, including computer vision and sensor based ML, to farmed livestock. Primary studies, peer review records, datasets, repository preprints without a peer reviewed version, editorials, duplicates, and reviews limited to crops, food processing, supply

chains or a single veterinary diagnostic task were excluded (53 records: peer review records (13), reviews of a single veterinary diagnostic task or disease (8), primary studies (7), food processing or supply chain (7), repository records without a peer reviewed version (7), other reasons (11)). Because a title search misses reviews whose titles carry no review term, five reviews already known to the authors, the review articles that reached full text in our own search and were excluded under criterion E4, and two further reviews identified after the search were screened by the same rule and added where they qualified (6 additions; the E4 articles that did not qualify are listed in the workbook with the reason).

Each of the 117 included reviews was coded from its abstract, and where available its stated aims and conclusions, at one of three levels: 0, it describes methods, tasks, data or accuracy, possibly stating that AI can support decisions without checking it; 1, it examines deployment, readiness or adoption for the studies it covers; 2, it examines whether model outputs were used to inform or take a specific management decision. The result is 106 reviews at level 0, 10 at level 1 and 1 at level 2 (Table S26). The one level 2 review (Ufitikirezi et al., 2026) coded on-farm deployment and quantitative evidence of productivity or management impact across 191 computer vision studies and found such evidence in fewer than 5 percent; it asks whether a model reached the farm and changed an outcome, not whether it was used to compare alternatives or recommend an action, which is the question of this review. Of the 11 reviews at level 1 or 2, 6 were published after the search date of this review (9 January 2026). The coding was made by one reviewer and is reported as descriptive.

Table S26. Reviews of AI and ML in livestock farming identified by the targeted search of 26 October 2026 (n = 117), with the level at which each examines what became of the models (0, methods, tasks, data and accuracy only; 1, deployment, readiness or adoption; 2, use of model outputs in a management decision). Source: S, title search of OpenAlex;

C, already known to the authors; E, review article excluded under criterion E4 at full text in our own search; R, identified after the search.

Source	First author (year)	Title	Journal or venue	Level
S	Ufitikirezi (2026)	Computer vision in livestock and poultry farming: A quantitative and translational review of research trends and real-world impact	Smart Agricultural Technology	2
R	Ruchay (2027)	Agentic AI for Livestock Housing Management: Applications, Benchmarking, and Readiness Assessment	Computers and Electronics in Agriculture	1
S	Choudhary (2026)	Artificial Intelligence in Sustainable and Climate-Smart Dairy Farming: A Critical Narrative Review of Evidence, Limitations and Research Priorities	Book Publisher International (a part of SCIENCEDOMAIN International)	1
S	Ghazy (2026)	A systematic review of artificial intelligence in small ruminant production systems: applications, performance outcomes, and reported implementation challenges	BMC Veterinary Research	1
S	Ruchay (2026)	Two Decades of Computer Vision in Precision Livestock Farming: an Umbrella Review of Applications, Benchmarking Evidence, and the Performance-to-Deployment Gap Across Cattle, Pig, and Poultry Systems	Computers and Electronics in Agriculture	1
S	Wang (2026)	Closed-loop artificial intelligence agents for animal epidemic prediction and decision support in livestock farming: a review	Frontiers in Veterinary Science	1
S	Zhang (2026)	An Operation-Centered Review of Deep-Learning Computer Vision for Dairy Cow Management	Agriculture	1
S	Trapanese (2024)	Short review of current limits and challenges of application of machine learning algorithms in the dairy sector	ACTA IMEKO	1
S	Georgopoulos (2023)	Factors Influencing the Adoption of Artificial Intelligence Technologies in Agriculture, Livestock Farming and Aquaculture: A Systematic Literature Review Using PRISMA 2020	Sustainability	1
S	Xi (2021)	A Review: Development of Computer Vision-Based Lameness Detection for Dairy Cows and Discussion of the Practical Applications	Sensors	1
S	Cockburn (2020)	Review: Application and Prospective Discussion of Machine Learning for the Management of Dairy Farms	Animals	1
S	Amorim (2026)	Applications and trends of accelerometer and artificial intelligence techniques in livestock production: a scoping review	Smart Agricultural Technology	0
S	Astuti (2026)	Dairy farming sustainability: A systematic review to understand mastitis and lameness detection using machine learning (ML)	Annals of Agricultural Science Cairo	0
S	Aydın (2026)	Integrating biomarkers and artificial intelligence for precision diagnostics in cattle health and herd management: a review	Veterinary Research Communications	0

S	Cahanap (2026)	Poultry Systems: A Systematic Review on IoT, Artificial Intelligence, and Multimodal Technologies for Precision Poultry Farming	International Journal of Transformative Multidisciplinary Studies	0
S	Dhungana (2026)	A Narrative Review on Internet of Things and Artificial Intelligence for Poultry Production	Animals	0
S	Engelbrecht (2026)	Precision farming: A review of artificial intelligence applications in broiler poultry farming	Emirates Journal of Food and Agriculture	0
S	Fang (2026)	Application of artificial intelligence in cattle disease diagnosis: A comprehensive review	Artificial Intelligence in Agriculture	0
S	Guo (2026)	A survey on deep learning-based livestock and poultry behavior recognition: Methods, dataset and prospects	Engineering Applications of Artificial Intelligence	0
S	Hassan (2026)	Multimodal fusion for precision livestock behavior monitoring: A systematic review of deep learning, pose estimation, and sensor integration strategies	Information Fusion	0
S	Islam (2026)	Computer Vision for Cattle Health and Welfare Monitoring: A Comprehensive Review of Methods, Applications, and Interdisciplinary Integration in Smart Agriculture	Sensors	0
S	Janani (2026)	Artificial Intelligence Applications in Poultry Health and Welfare Monitoring: A Systematic Review and Meta-analysis of Model Performance	Journal of Poultry Sciences and Avian Diseases	0
S	Kaur (2026)	Exploring Machine Learning and IoT Technologies for Disease Detection in Cows: A Systematic Review	Springer proceedings in mathematics & statistics	0
S	Lo Presti (2026)	Innovative Techniques for the Evaluation and Optimization of Sustainable Feeds in Poultry Nutrition: A Critical Integrative Review of Advanced Analytical Approaches, Omics, and Artificial Intelligence	Applied Sciences	0
S	Masyhur (2026)	Artificial Intelligence Applications in Smart Feedlot and Livestock Management: A Comprehensive Review with Perspectives for Malaysia	Advances in Computational and Intelligent Systems	0
S	Nasser (2026)	Deep learning based 2D computer vision in precision livestock farming for pigs: A systematic literature review and future research directions	Smart Agricultural Technology	0
S	Paneru (2026)	Computer vision models for precision poultry farming: A narrative review of behavioral and welfare monitoring studies	Poultry Science	0
S	Rebh (2026)	The Impact of Artificial Intelligence Techniques on The Growth and Carcass Performance of Broiler Chickens, A Review	Al-Kunooze Scientific Journal	0
S	Ruchay (2026)	Open Datasets and AI/GenAI-Driven Computer Vision for Precision Livestock Farming: A Bidirectional Review	Computers and Electronics in Agriculture	0
S	Sreedhara (2026)	Application of Machine Learning and Artificial Intelligence for Disease Detection in Dairy Animals: A Critical Review	UTTAR PRADESH JOURNAL OF ZOOLOGY	0
S	Yin (2026)	Computer vision-based body measurement of	Computers and	0

		cattle and pigs: A systematic review	Electronics in Agriculture	
S	Yuan (2026)	A review of cattle behavior recognition methods based on computer vision and deep learning	Smart Agricultural Technology	0
S	Zin (2026)	— Invited Review — Computer vision in precision livestock farming: artificial intelligence-driven technologies and applications for sustainable animal production	Animal Bioscience	0
S	Adam (2025)	Deep Learning Approaches for Automatic Livestock Detection in UAV Imagery: State-of-the-Art and Future Directions	Future Internet	0
S	Adenuga (2025)	Advancing Livestock Reproductive Performance through Artificial Intelligence: A Systematic Literature Review	International Journal of Advancing Research, Innovation, and Entrepreneurship	0
S	Antognoli (2025)	Computer Vision in Dairy Farm Management: A Literature Review of Current Applications and Future Perspectives	Animals	0
S	Deng (2025)	Methods and challenges in computer vision-based livestock anomaly detection, a systematic review	Biosystems Engineering	0
C	Distante (2025)	Artificial Intelligence Applied to Precision Livestock Farming: A Tertiary Study	Smart Agricultural Technology	0
S	Grzesiak (2025)	The Use of Selected Machine Learning Methods in Dairy Cattle Farming: A Review	Animals	0
S	Irlon (2025)	Systematic Literature Review of Deep Learning Based Approaches for Broiler Chicken Growth Prediction	Systematic Literature Review Journal	0
S	Juhos (2025)	Integrating Deep Learning and Transcriptomics to Assess Livestock Aggression: A Scoping Review	Biology	0
S	Kumar (2025)	A Review on the Role of Artificial Intelligence in Livestock Health Management	Archives of Current Research International	0
S	Li (2025)	A survey of open-access datasets for computer vision in precision poultry farming	Poultry Science	0
S	Maulana (2025)	The Integration of Artificial Intelligence and Internet of Things in Ventilation Systems of Closed Houses in Broiler Chicken Farms of Indonesia: A Literature Review	Journal of World s Poultry Research	0
S	Meng (2025)	Livestock Biometrics Identification Using Computer Vision Approaches: A Review	Agriculture	0
S	Mia (2025)	Machine learning overview and its application in the livestock industry	Meat Research | ISSN (Online Version) 2790-1971	0
S	Naeem (2025)	Advancements in machine learning applications in poultry farming: a literature review	The Journal of Applied Poultry Research	0
S	Parivendan (2025)	Socializing AI: Integrating Social Network Analysis and Deep Learning for Precision Dairy Cow Monitoring—A Critical Review	Animals	0

S	Peter (2025)	ASAS-NANP symposium: mathematical modeling in animal nutrition: review of time series techniques and machine learning models for advancing livestock production	Journal of Animal Science	0
S	Previti (2025)	A systematic literature review on integrated livestock systems using machine learning methods: strengths and future directions from an animal welfare perspective within the "one health" approach	Journal of Consumer Protection and Food Safety	0
S	Santoro (2025)	Artificial Neural Networks for Predicting Emissions from the Livestock Sector: A Review	Animals	0
S	Singh (2025)	The advent of artificial intelligence in dairy farming: A comprehensive overview	International Journal of Veterinary Sciences and Animal Husbandry	0
S	Tedeschi (2025)	– Invited Review – Advancing precision livestock farming: integrating artificial intelligence and emerging technologies for sustainable livestock management	Animal Bioscience	0
S	Umurungi (2025)	Leveraging the potential of convolutional neural networks in poultry farming: a 5-year overview	World s Poultry Science Journal	0
S	Xu (2025)	Recent advances in computer vision for non-contact phenotyping and weight estimation in livestock: A systematic review	Information Processing in Agriculture	0
S	Yang (2025)	Review of computer vision for livestock body conformation assessment	Agriculture Communications	0
S	Anusree (2024)	A Comprehensive Review of Recent Artificial Intelligence Techniques and IOT Applications in Dairy Farms	International Conference on Advanced Computing and Communication Systems (ICACCS)	0
S	Bhujel (2024)	A systematic survey of public computer vision datasets for precision livestock farming	Computers and Electronics in Agriculture	0
E	Chowdhury (2024)	Artificial intelligence for enteric methane: a perspective	Animal Frontiers	0
S	Deepak (2024)	Advancements in Automated Livestock Monitoring: A Concise Review of Deep Learning-Based Cattle Activity Recognition	International Conference on Advanced Computing and Communication Systems (ICACCS)	0
S	Fatoki (2024)	Role of computer vision and deep learning algorithms in livestock behavioural recognition: A state-of-the-art- review	Edelweiss Applied Science and Technology	0
S	Ghavipanje (2024)	Trends and Future Directions of Artificial Intelligence Applications in Iranian Livestock Production Systems – A Review	Annals of Animal Science	0
S	Goyal (2024)	A Literature Review on the Role of Internet of Things, Computer Vision, and Sound Analysis in a	ACS Agricultural Science &	0

		Smart Poultry Farm	Technology	
S	Kalita (2024)	Application of artificial intelligence and machine learning in poultry disease detection and diagnosis: A review	Letters In Animal Biology	0
S	Ma (2024)	Computer-Vision-Based Sensing Technologies for Livestock Body Dimension Measurement: A Survey	Sensors	0
S	Ma (2024)	Computer Vision-Based Measurement Techniques for Livestock Body Dimension and Weight: A Review	Agriculture	0
S	Mahaboob (2024)	Survey of livestock monitoring and management system using a machine learning algorithm	AIP conference proceedings	0
S	Menezes (2024)	Artificial intelligence for livestock: a narrative review of the applications of computer vision systems and large language models for animal farming	Animal Frontiers	0
S	Michelena (2024)	A review and future trends of precision livestock over dairy and beef cow cattle with artificial intelligence	Logic Journal of IGPL	0
S	Olanrewaju (2024)	Review of Poultry Monitoring using Computer Vision	Nigerian Journal of Physics	0
S	Pradeepa (2024)	A Review of Smart Livestock Farming Using Artificial Intelligence	Journal of Information Technology Review	0
S	Reddy (2024)	A Comprehensive Review of Machine Learning Approaches in Livestock Health Monitoring	Journal of Big Data Technology and Business Analytics	0
S	Reza (2024)	Thermal imaging and computer vision technologies for the enhancement of pig husbandry: a review	Journal of Animal Science and Technology	0
S	Rohan (2024)	Application of deep learning for livestock behaviour recognition: A systematic literature review	Computers and Electronics in Agriculture	0
S	Shwetha (2024)	Latest Trend and Challenges in Machine Learning– and Deep Learning–Based Computational Techniques in Poultry Health and Disease Management: A Review	Journal of Computer Networks and Communications	0
S	Subramani (2024)	Machine learning and deep learning techniques for poultry tasks management: a review	Multimedia Tools and Applications	0
S	Taleb (2024)	Using Artificial Intelligence to Improve Poultry Productivity – A Review	Annals of Animal Science	0
S	Ufitikirezi (2024)	Enhancing cattle production and management through convolutional neural networks. A review	Czech Journal of Animal Science	0
S	Yang (2024)	Computer Vision-Based cybernetics systems for promoting modern poultry Farming: A critical review	Computers and Electronics in Agriculture	0
S	Zhao (2024)	Intelligent Flocks: A Systematic Review of Artificial Intelligence Driven Precision Reproductive Management in Small Ruminants	Iranian journal of applied animal science	0
S	Chen (2023)	A Review of Posture Detection Methods for Pigs	Applied Sciences	0

		Using Deep Learning		
S	Curti (2023)	Applications of livestock monitoring devices and machine learning algorithms in animal production and reproduction: an overview	Animal Reproduction	0
S	De Vries (2023)	Invited Review: Examples and opportunities for artificial intelligence (AI) in dairy farms*	Applied Animal Science	0
S	Digambar (2023)	Review on poultry automation using IoT and machine learning	World Journal of Advanced Research and Reviews	0
S	McVey (2023)	Invited Review: Applications of unsupervised machine learning in livestock behavior: Case studies in recovering unanticipated behavioral patterns from precision livestock farming data streams	Applied Animal Science	0
S	Mijwil (2023)	Innovative Livestock: A Survey of Artificial Intelligence Techniques in Livestock Farming Management	Wasit Journal of Computer and Mathematics Science	0
S	Noor (2023)	Sheep health behavior analysis in machine learning: A short comprehensive survey	Smart Agricultural Technology	0
S	Petrović (2023)	Application of computer vision in livestock and crop production—A review	Computing and artificial intelligence.	0
S	Verma (2023)	A Review of Machine Learning Models for Disease Prediction in Poultry Chickens	Algorithms for intelligent systems	0
S	Wang (2023)	Application of infrared thermography and machine learning techniques in cattle health assessments: A review	Biosystems Engineering	0
S	Wang (2023)	A review of three-dimensional computer vision used in precision livestock farming for cattle growth management	Computers and Electronics in Agriculture	0
S	Hakem (2022)	Classification of Body Weight in Beef Cattle via Machine Learning Methods: A Review	Procedia Computer Science	0
S	Hossain (2022)	A systematic review of machine learning techniques for cattle identification: Datasets, methods and future directions	Artificial Intelligence in Agriculture	0
S	Kaur (2022)	Cattle identification with muzzle pattern using computer vision technology: a critical review and prospective	Soft Computing	0
S	Nascimento (2022)	Application of Artificial Intelligence in Cattle Farming: A Scope Review	Revista Electronica de Veterinaria	0
S	Ojo (2022)	Internet of Things and Machine Learning techniques in poultry health and welfare management: A systematic literature review	Computers and Electronics in Agriculture	0
S	Rahman (2022)	Prospect and scope of artificial neural network in livestock farming: a review	Biological Rhythm Research	0
S	Roy (2022)	Discussing the present, past, and future of Machine learning techniques in livestock farming: A systematic literature review	International Conference on Machine Learning, Big Data, Cloud and Parallel Computing (COM-IT-CON)	0

S	Yousefi (2022)	A Systematic Literature Review on the Use of Deep Learning in Precision Livestock Detection and Localization Using Unmanned Aerial Vehicles	IEEE Access	0
S	Aharwal (2021)	Worth of Artificial Intelligence in the Epoch of Modern Livestock Farming: A Review	Agricultural Science Digest - A Research Journal	0
E	Balhara (2021)	Data mining and decision support systems for efficient dairy production	Veterinary World	0
C	Chen (2021)	Behaviour Recognition of Pigs and Cattle: Journey from Computer Vision to Deep Learning	Computers and Electronics in Agriculture	0
S	Dohmen (2021)	Computer vision-based weight estimation of livestock: a systematic literature review	New Zealand Journal of Agricultural Research	0
S	Faysal (2021)	A Review of Groundbreaking Changes in the Poultry Industry in Bangladesh Using the Internet of Things (IoT) and Computer Vision Technology	International Conference on Automation, Control and Mechatronics for Industry 4.0 (ACMI)	0
C	Li (2021)	Practices and Applications of Convolutional Neural Network-Based Computer Vision Systems in Animal Farming: A Review	Sensors	0
S	Lima (2021)	Artificial Intelligence in Support of Welfare Monitoring of Dairy Cattle: A Systematic Literature Review	International Conference on Computational Science and Computational Intelligence (CSCI)	0
S	Mahmud (2021)	A systematic literature review on deep learning applications for precision cattle farming	Computers and Electronics in Agriculture	0
S	Nayak (2021)	Predicting the Cattle Production Parameters Through Deep Learning Approach: A Review	IEEE International Conference on Electronics, Computing and Communication Technologies (CONECCT)	0
S	Nyalala (2021)	Weight and volume estimation of poultry and products based on computer vision systems: a review	Poultry Science	0
S	Oliveira (2021)	A review of deep learning algorithms for computer vision systems in livestock	Livestock Science	0
S	Abd Aziz (2020)	A Review on Computer Vision Technology for Monitoring Poultry Farm—Application, Hardware, and Software	IEEE Access	0
S	Bindushree (2020)	Review on poultry automation using IoT and machine learning	World Journal of Advanced Research and Reviews	0
S	García (2020)	A systematic literature review on the use of machine learning in precision livestock farming	Computers and Electronics in	0

			Agriculture	
S	Okinda (2020)	A review on computer vision systems in monitoring of poultry: A welfare perspective	Artificial Intelligence in Agriculture	0
S	Singh (2020)	Artificial Intelligence and IoT based Monitoring of Poultry Health: A Review	IEEE COMNETSAT	0
S	Slob (2020)	Application of machine learning to improve dairy farm management: A systematic literature review	Preventive Veterinary Medicine	0
S	Dongre (2016)	Applications of artificial neural networks for enhanced livestock productivity: A review	The Indian Journal of Animal Sciences	0

Note. Level 1 reviews: Ruchay (2027), Choudhary (2026), Ghazy (2026), Ruchay (2026), Wang (2026), Zhang (2026), Trapanese (2024), Georgopoulos (2023), Xi (2021), Cockburn (2020); level 2: Ufitikirezi (2026). Reviews are ordered by level, then year. Titles are given as indexed in OpenAlex. The full screening log with every record, its decision and the reason is held in the screening workbook, available from the authors on request.

S12. Sensitivity of the search strategy

The search strategy of Section S1 was tested in four ways. The rules were written on 26 October 2026 before any analysis was run and are reproduced, together with every record, decision and reason, in a workbook available from the authors on request. First, a known-item check: each of the 165 included studies was matched by DOI or title against the four record sets exported on 9 January 2026. Second, term matching: each term of the executed blocks, and each candidate term considered for addition, was tested against the title, abstract and keywords of each included study as exported. Third, an expanded re-run: the three blocks were widened with the candidate terms (AI block: artificial, convolutional and recurrent neural networks, LSTM, decision tree, k-nearest neighbours, LightGBM, CatBoost, fuzzy logic, ANFIS, computer vision, object detection, genetic algorithm, metaheuristic, particle swarm; livestock block: the terms dropped in PubMed plus calf, buffalo, broiler, hen, small ruminant; sustainability block: the terms dropped in PubMed plus ammonia, nitrous oxide, nitrogen, phosphorus, manure, eutrophication, land use, biodiversity, feed efficiency, feed conversion, emission) and run in PubMed, the platform of the

original search, with the original limits and an entry date on or before 9 January 2026, and in OpenAlex as a proxy for the breadth of Web of Science and CAB Abstracts. The records returned by the expanded string but not by the original one were sampled at random (100 per platform, seed 2026) and screened from title and abstract against the eligibility codes of Table 1 of the main text; a record passing the codes that can be judged from the abstract is called potentially eligible, and one with a single uncertain criterion is called borderline. Each potentially eligible or borderline record was then looked up in the pooled 2,664 records of the original search, and only records absent from all four exports count as missed. Fourth, the strategy was assessed against the six elements of the PRESS checklist.

Known-item check (Table S27). All 165 included studies are present in the exports: Web of Science retrieved 141, CAB Abstracts 95, PubMed 42 and IEEE Xplore 1; 79 were retrieved by one database only (59 by Web of Science alone, 16 by CAB Abstracts alone, 4 by PubMed alone), 58 by two and 28 by three, none by all four. Because the corpus was screened from these very exports, this check confirms the record of the search but cannot measure what the search missed.

Term matching (Table S28). The AI block matched 163 of the 165 included studies, but 90 of them only through the four generic terms (artificial intelligence, machine learning, deep learning, neural network*), and 51 contain one of the suggested algorithm names without any of the five specific executed AI terms. The sustainability block matched 139 studies, 48 of them only through sustainab*, and 35 contain one of the suggested outcome terms (most often manure, nitrogen, ammonia, land use or feed efficiency) without any specific executed outcome term. The 2 studies with no AI term and the 26 with no sustainability term in the exported text were retrieved through fields the exports do not carry (Keywords Plus in Web of Science, subject

headings). The search therefore depended on generic words, and studies that name their method and their outcome specifically were retrieved only when a generic word also appeared.

Expanded re-run (Table S29). In PubMed the original string with its limits returns 426 records, exactly the count of 9 January 2026, and the expanded string returns 1433, that is 1007 additional records. Of the 100 sampled, 5 were potentially eligible and 5 borderline from title and abstract; 4 potentially eligible and 5 borderline records were absent from all four exports. Applied to the 1007 additional records, this gives an estimated 40 (16 to 99) potentially eligible studies missed in PubMed alone, or 91 (48 to 163) counting borderline records. In OpenAlex the expanded string returns 1221 records that the original string does not; of the 100 sampled, 13 were potentially eligible and 13 borderline, and 12 potentially eligible and 12 borderline records were absent from all four exports, an estimated 147 (85 to 242) potentially eligible studies, or 293 (204 to 406) counting borderline records. The two platforms overlap, so the estimates cannot be added. Potentially eligible from an abstract is not included: in the original review, 165 of the 310 reports read in full were included, so roughly half of the potentially eligible records would be expected to survive full text screening. The missed records found in the samples (Table S31) are of the same kinds as the included studies: prediction of ammonia, odour or nitrogen outputs, manure treatment and composting, pasture mapping and herbage prediction, and energy or feed efficiency models.

PRESS self-assessment (Table S30). The strategy translates the research question correctly, is syntactically sound and reproduces its record counts, but it used no controlled vocabulary in any database and its text words were narrower than the vocabulary of the field in both the AI and the sustainability blocks. The assessment was made by the authors. An independent PRESS review by a university librarian, received in October 2026, agreed that the strategy is narrower than the

topic and added three points. First, the livestock terms omit several species, such as horses and other equids, rabbits, turkeys, ducks, geese, camels, llamas and alpacas. Second, Compendex, AGRICOLA and the ProQuest Agricultural and Environmental Science Collection would extend coverage; the Web of Science string returned more than 2,600 records in Compendex before deduplication and screening. Third, a check run by the librarian in October 2026 returned more records than the January search (Web of Science 960 against 909, PubMed 625 against 426), mainly because studies published since January 2026 are now indexed. The searches were not re-run, and these gaps are reported as limitations (main text Section 4.5). Conclusion: the corpus is a reproducible but not exhaustive sample of the eligible literature. The descriptive counts in the main text describe the retrieved corpus; the central comparison between claimed and delivered decision support concerns proportions within that corpus and the missed records are of the same kinds as the included ones, but the limitation is stated in Section 4.5 of the main text and the expanded terms are recorded here for any re-run.

Table S27. Retrieval of the 165 included studies by database, from the record sets exported on 9 January 2026 (matched by DOI, or by title where the DOI was missing).

Item	Included studies, n	% of 165
Retrieved by Web of Science	141	85.5
Retrieved by CAB Abstracts	95	57.6
Retrieved by PubMed	42	25.5
Retrieved by IEEE Xplore	1	0.6
Retrieved by Web of Science only	59	35.8
Retrieved by CAB Abstracts only	16	9.7
Retrieved by PubMed only	4	2.4
Retrieved by one database	79	47.9
Retrieved by two databases	58	35.2
Retrieved by three databases	28	17.0
Retrieved by all four	0	0.0
Not found in any export	0	0.0

Table S28. Search terms matched in the title, abstract and keywords of the 165 included studies as exported (case insensitive; * is truncation; hyphenated and spaced forms both

counted). Executed terms are given in the Web of Science form; suggested terms are those proposed at review.

Group	Term	Included studies containing the term, n
AI block, generic	artificial intelligence	17
AI block, generic	machine learning	76
AI block, generic	deep learning	14
AI block, generic	neural network*	59
AI block, specific	random forest	58
AI block, specific	support vector machine	7
AI block, specific	gradient boosting	11
AI block, specific	XGBoost	7
AI block, specific	reinforcement learning	0
Livestock block	livestock	81
Livestock block	dairy	37
Livestock block	cattle	58
Livestock block	cow*	27
Livestock block	bovine	6
Livestock block	goat*	7
Livestock block	caprine	0
Livestock block	sheep	17
Livestock block	ovine	0
Livestock block	pig*	15
Livestock block	swine	10
Livestock block	porcine	1
Livestock block	poultry	19
Livestock block	chicken*	5
Livestock block	farm animal*	0
Sustainability block, generic	sustainab*	75
Sustainability block, specific	life cycle assessment	3
Sustainability block, specific	life cycle analysis	0
Sustainability block, specific	LCA	4
Sustainability block, specific	carbon footprint	6
Sustainability block, specific	greenhouse gas	24
Sustainability block, specific	GHG	8
Sustainability block, specific	methane	57
Sustainability block, specific	environmental impact	21
Sustainability block, specific	water footprint	0
Sustainability block, specific	water use	1
Sustainability block, specific	energy use	4
Sustainability block, specific	economic viability	2
Sustainability block, specific	profitability	4

Sustainability block, specific	production efficiency	5
Suggested AI terms (not executed)	convolutional neural network or CNN	7
Suggested AI terms (not executed)	long short-term memory or LSTM	8
Suggested AI terms (not executed)	recurrent neural network or RNN	1
Suggested AI terms (not executed)	artificial neural network or ANN	36
Suggested AI terms (not executed)	decision tree	2
Suggested AI terms (not executed)	k-nearest neighbour or kNN	5
Suggested AI terms (not executed)	LightGBM	0
Suggested AI terms (not executed)	CatBoost	2
Suggested AI terms (not executed)	fuzzy or ANFIS	6
Suggested AI terms (not executed)	computer vision	1
Suggested AI terms (not executed)	object detection	3
Suggested AI terms (not executed)	genetic algorithm, particle swarm or metaheuristic	6
Suggested outcome terms (not executed)	ammonia	15
Suggested outcome terms (not executed)	nitrous oxide or N2O	6
Suggested outcome terms (not executed)	nitrogen	16
Suggested outcome terms (not executed)	phosphorus	2
Suggested outcome terms (not executed)	manure	43
Suggested outcome terms (not executed)	eutrophication	1
Suggested outcome terms (not executed)	land use	12
Suggested outcome terms (not executed)	biodiversity	3
Suggested outcome terms (not executed)	feed efficiency or feed conversion	11
Summary	AI block matched only by generic terms	90
Summary	No AI term in the exported text	2
Summary	Suggested AI term present, no specific executed AI term	51
Summary	Sustainability block matched only by sustainab*	48
Summary	No sustainability term in the exported text	26
Summary	Suggested outcome term present, no specific executed outcome term	35
Summary	No livestock term in the exported text	5

Table S29. Expanded re-run of the search in PubMed and OpenAlex (26 October 2026) with a screened random sample of the additional records. PubMed: original string and limits of Section S1 plus an entry date on or before 9 January 2026; OpenAlex: title and abstract search, 2010 to 2026, English, articles and reviews. Wilson 95% confidence intervals; estimates are the sampled share applied to the additional records.

Quantity	PubMed	OpenAlex
Original string, records	426	1910
Expanded string, records	1433	3182
Additional records (expanded minus original)	1007	1221
Random sample screened (seed 2026)	100	100
Potentially eligible in the sample	5 of 100 (5.0%; 95% CI 2.2 to 11.2)	13 of 100 (13.0%; 95% CI 7.8 to 21.0)
Potentially eligible or borderline in the sample	10 of 100 (10.0%; 95% CI 5.5 to 17.4)	26 of 100 (26.0%; 95% CI 18.4 to 35.4)
Potentially eligible and absent from all four exports	4 of 100 (4.0%; 95% CI 1.6 to 9.8)	12 of 100 (12.0%; 95% CI 7.0 to 19.8)
Potentially eligible or borderline and absent from all four exports	9 of 100 (9.0%; 95% CI 4.8 to 16.2)	24 of 100 (24.0%; 95% CI 16.7 to 33.2)
Estimated potentially eligible records missed (share times additional records)	40 (16 to 99)	147 (85 to 242)
Estimated potentially eligible or borderline records missed	91 (48 to 163)	293 (204 to 406)

Note. The two platforms overlap, so their estimates are not additive. Screening was made from title and abstract by one reviewer and classifies records as potentially eligible, not as included; in the original review about half of the reports read in full were included. The strings as run are in the workbook.

Table S30. Self-assessment of the executed search strategy against the six elements of the PRESS checklist (Peer Review of Electronic Search Strategies). An independent review by a university librarian is summarized in the text of Section S12.

PRESS element	What the strategy did	Weakness found	Consequence or action
1. Translation of the research question	Three concept blocks (AI or ML method; livestock species; sustainability outcome) joined with AND, matching the three parts of the eligibility criteria. Decision support was not searched because it emerged as the organising outcome during analysis.	The sustainability block mixes a framing term (sustainab*) with outcome terms, so it finds studies that use the word sustainability as much as studies that measure an outcome; specific environmental outcomes are under-represented.	Moderate. Addressed in the expanded re-run by adding outcome terms (Table S29).
2. Boolean and proximity operators	OR within blocks, AND between blocks, correct nesting in all four platforms; no proximity operators.	None found. Truncation was removed in IEEE Xplore and PubMed because the platforms do not accept it inside quoted phrases, which narrows those two searches to single word forms.	Minor for IEEE Xplore (65 records); larger for PubMed, where several terms were also dropped (Section S1).
3. Subject headings	No controlled vocabulary was used in any database: no MeSH terms in PubMed beyond automatic term mapping, no CAB	Records indexed under headings such as Machine Learning, Neural Networks (Computer), Livestock, Manure, Greenhouse Gases,	Lower sensitivity. A re-run should add the headings available in each

	Thesaurus descriptors, no Keywords Plus strategy in Web of Science.	Ammonia or Animal Feed were retrieved only when the free text matched.	database.
4. Text word searching	AI block: nine terms; livestock block: fifteen; sustainability block: fifteen. Term matching on the 165 included studies shows that the AI block matched 90 of them only through the four generic terms and the sustainability block matched 48 only through sustainab*.	The AI block omits common algorithm names (artificial neural network, convolutional and recurrent networks, LSTM, decision tree, k-nearest neighbours, LightGBM, CatBoost, fuzzy or ANFIS, computer vision, object detection, metaheuristics); 51 included studies contain one of these without any specific executed AI term. The sustainability block omits ammonia, nitrous oxide, nitrogen, phosphorus, manure, eutrophication, land use, biodiversity and feed efficiency; 35 included studies contain one of these without any specific executed outcome term.	Major. The expanded re-run estimates the loss (Table S29); the terms are listed for any re-run.
5. Spelling, syntax and line numbers	Strings were checked against the exports: the PubMed string with its limits reproduces exactly 426 records; the other three counts match the exports. No spelling errors found.	British and American spellings are not both covered (neighbour, behaviour are absent from the strings; only American forms were used where it matters). Hyphenated forms (life-cycle, greenhouse-gas) rely on platform tokenisation.	Minor.
6. Limits and filters	English language, journal articles only, publication years 2010 to 2026, applied in all four databases.	The 2010 lower limit was not justified in advance and excludes early neural network work on livestock; the journal article limit excludes conference papers, consistent with criterion E7.	Temporal conclusions apply to the 2010 to 2026 window (addressed under the comment on lines 167 to 168).

Table S31. The 33 potentially eligible or borderline records in the two samples that are absent from all four record sets of the original search (identifier: PubMed PMID or OpenAlex work ID).

Source	Identifier	Year	Title	Decision
OpenAlex	W2140544245	2011	Response surface and neural network models for performance of broiler chicks fed diets varying in digestible protein and critical amino acids from 11 to 17 days of age	borderline
OpenAlex	W2179571046	2013	Bootstrapped neural network models for analyzing the responses of broiler chicks to dietary protein and branched chain amino acids	borderline
OpenAlex	W2081956134	2015	Predicting nitrogen excretion from cattle	borderline
OpenAlex	W2801963227	2018	Analysis of land-use/land-cover changes in a livestock landscape dominated by traditional silvopastoral systems: a methodological approach	borderline

OpenAlex	W2797997284	2018	Assessing the Spatial and Occupation Dynamics of the Brazilian Pasturelands Based on the Automated Classification of MODIS Images from 2000 to 2016	eligible
OpenAlex	W2995699160	2019	Applied improved RBF neural network model for predicting the broiler output energies	eligible
OpenAlex	W2969335832	2019	Using machine learning to estimate herbage production and nutrient uptake on Irish dairy farms	eligible
OpenAlex	W3141950031	2020	Construction Method and Performance Test of Prediction Model for Laying Hen Breeding Environmental Quality Evaluation	borderline
OpenAlex	W3037067867	2020	Field and crop specific manure application on a dairy farm based on historical data and machine learning	eligible
OpenAlex	W3212896134	2021	A Design of a Solar Fermentation System on Chicken Manure by Fuzzy Logic Temperature Control	eligible
OpenAlex	W3120812016	2021	Utilising grassland management and climate data for more accurate prediction of herbage mass using the rising plate meter	borderline
OpenAlex	W4280559920	2022	Assessing the utility of Sentinel-2 MSI in mapping an encroaching Serephium plumosum in South African rangeland	borderline
OpenAlex	W4304185232	2022	Using Near-Infrared Spectroscopy and Stacked Regression for the Simultaneous Determination of Fresh Cattle and Poultry Manure Chemical Properties	eligible
OpenAlex	W4388205652	2023	Exploring soil property spatial patterns in a small grazed catchment using machine learning	borderline
OpenAlex	W4319456044	2023	From crowd to herd counting: How to precisely detect and count African mammals using aerial imagery and deep learning?	eligible
OpenAlex	W4401958681	2024	Identification of Pasture Degradation Using Remote Sensing Data and Machine Learning: A Case Study of Obichnik	eligible
OpenAlex	W4411939654	2025	ANALYSIS OF FUZZY K-NEAREST NEIGHBORS WITH KNN AND FUZZY LOGIC APPROACH ON DUCK BROILER PRODUCTION	borderline
OpenAlex	W4412763772	2025	Heavy metals bioavailability prediction in swine manure composting: Development of ensemble machine learning models	eligible
OpenAlex	W4412862713	2025	Kinetic analysis and prediction modeling by advanced machine learning of pyrolysis of dairy cattle manure from conventional and organic systems	eligible
OpenAlex	W4416668260	2025	Nutrient–response modeling with a single and interpretable artificial neuron	borderline
OpenAlex	W7134163906	2026	Bi-objective optimization of hybrid renewable energy systems with ice storage vs. battery storage for sustainable dairy farming	borderline
OpenAlex	W7163321104	2026	Development of a machine learning-based livestock odour dispersion forecasting system	eligible
OpenAlex	W7122390081	2026	Prediction of Ammonia Mitigation Efficiency in Sodium Bisulfate-Treated Broiler Litter Using Artificial Neural Networks	eligible

OpenAlex	W7126182996	2026	Reclaiming West Virginia gas wells for agrivoltaics: a fuzzy logic approach	borderline
PubMed	20952719	2010	The integration of broiler chicken threonine responses data into neural network models.	borderline
PubMed	30602245	2019	Attenuation of tetracyclines during chicken manure and bagasse co-composting: Degradation, kinetics, and artificial neural network modeling.	borderline
PubMed	33445008	2021	Response surface methodology and artificial neural network modelling for enhancing maturity parameters during vermicomposting of floral waste.	borderline
PubMed	35724572	2022	Evaluation of a cascade artificial neural network for modeling and optimization of process parameters in co-composting of cattle manure and municipal solid waste.	eligible
PubMed	36055837	2022	Predicting dry matter intake in Canadian Holstein dairy cattle using milk mid-infrared reflectance spectroscopy and other commonly available predictors via artificial neural networks.	borderline
PubMed	36611774	2022	Prediction of Ammonia Concentration in a Pig House Based on Machine Learning Models and Environmental Parameters.	eligible
PubMed	37317090	2023	Rumen Microbiota Predicts Feed Efficiency of Primiparous Nordic Red Dairy Cows.	eligible
PubMed	38823283	2024	Feed intake in housed dairy cows: validation of a three-dimensional camera-based feed intake measurement system.	borderline
PubMed	38475164	2024	Structure Optimization and Data Processing Method of Electronic Nose Bionic Chamber for Detecting Ammonia Emissions from Livestock Excrement Fermentation.	eligible

Note. Borderline means that one eligibility criterion could not be settled from the abstract (most often whether a feed efficiency or nutrient response outcome counts as a sustainability outcome, or whether the learning method is the core of the study). Reasons are given in the workbook.

S13. Canonicalization rules, recorded judgement overrides and a check of the derived variables

This section states the rules by which the free text extraction fields were turned into the canonical variables, counts the entries of the cleaning log by kind, lists every judgement override, and reports a recount of countries under alternative conventions. The recount was specified in writing on 26 October 2026 before it was run. The rules, every log entry with its

class, every code and every count are held in a canonicalization workbook available from the authors on request; the mapping code is released as canonical_rules.py with the analysis materials.

Rules. Three kinds of rule were used. First, recoding in place: species, geographic scope, production system, method category, validation method, model transparency and reproducibility were recoded to the value sets written in the workbook codebook (Table S12); the recoded value replaced the extracted one, and the original value and the reason were written to the cleaning log. Second, lookup tables: algorithm family, application domain, outcome unit family and sample size bin were assigned by a map sheet with one row per study that pairs the extracted value with its canonical value. Third, formulas: model purpose is the first word of the extracted field, with compound entries coded Multiple; country is the first country listed in the extracted field after sub-national detail in brackets is removed and exonyms are standardized (for example USA to United States), and regional or multi-country labels (Global, International, East Africa, Eastern Africa, Multiple European countries, European Union and United Kingdom, Mongolia and Inner Mongolia, China) are kept as labels; input structure is coded by hand under the definitions given below; and the Global North or South region of a study follows the Country Region Map of the workbook. Apart from the judgement overrides below, every canonical value follows from these rules and tables; the hand coding of input structure is itself a table with one row per study.

Judgement overrides. The cleaning log holds 279 entries (Table S32). Of these, 117 are mechanical normalizations (spelling or format unified, bracketed detail moved to the notes column), 40 are rule based collapses to a canonical value (for example several validation methods to Multiple, poultry subtypes to Poultry), 100 are coding, flag, exclusion or audit entries, and 22 are judgement overrides, in which the cleaning reviewer replaced an extracted

value by a different canonical value on the basis of the study rather than of a written rule. The 22 overrides (Table S33) concern five fields: geographic scope (6), production system (5), method category (4), reproducibility (4) and primary species (3); 17 concern studies in the final corpus and 5 concern studies excluded under amendments 6 and 9 (LopezAndres_2018_001, Navon_2013_001, Zhang_2022_002), whose entries are kept as audit trail.

Input structure, hand coding. Input structure was coded by hand for all 165 studies into four categories from the input variables, data source and primary algorithm fields, under written definitions (imagery or spectral inputs; ordered sequences in time whose order the model uses; both; everything else as tabular measurements). The corpus has 86 tabular, 48 imagery or spectral, 16 sequential or temporal and 15 imagery and temporal studies. Table S21, Fig. 4 and Section 3.3 of the main text use this coding. The code of every study is given in the canonicalization workbook, with a column for the check by a second author (P.V.).

Country (Table S34). Under the published convention each study counts once under the first country listed: the 165 studies take 50 first-listed values, 43 countries and 7 regional or multi-country labels (one study each). Ten studies name more than one country and are counted under the first named, which is the country where the data were collected when the field distinguishes data from modelling locations; 104 studies carried sub-national detail in brackets that was removed, and 11 carried an exonym that was standardized. Two alternative conventions were applied: fractional, in which each named country receives an equal share of a study, and any mention, in which each named country counts once. The four leading countries and their order are the same under all three conventions (China, United States, Brazil, Türkiye), and the same five lead under the first-listed and fractional conventions; when every mention counts, South Korea shares fifth place with Canada. The number of countries named at least once rises from 43

to 46 (Denmark, Finland, Sweden appear only as second or later countries), and ranks five to eight are shared, with ties, among South Korea, Germany, Canada, Australia and the United Kingdom. The sentence of Section 3.2 of the main text that spoke of research originating in 50 countries has been reworded accordingly, and Fig. 2b and Figure S5 are labelled as first-listed country.

Table S32. Entries of the cleaning log of the analysis workbook by class (n = 279 entries).

Class	What it covers	Entries	Fields concerned (entries)
Mechanical normalisation	Spelling or format unified; bracketed detail moved to the notes column; exonyms standardized	117	Extractor Notes (36); Production System (18); Model Transparency (17); Validation Method (14); Reproducibility (13); Primary Species (11); Country (8)
Rule-based collapse	Several values collapsed to one canonical value by a written rule (for example several validation methods to Multiple, poultry subtypes to Poultry, codebook compliance recodes)	40	Validation Method (24); Primary Species (10); Measurement Level (5); 1 other field (1 entry)
Judgement override	An extracted value replaced by a different canonical value on the basis of the study rather than of a written rule (listed in Table S33)	22	Geographic Scope (6); Production System (5); ML Category (4); Reproducibility (4); Primary Species (3)
Coding, flag, exclusion or audit entry	Functional decision support codes, capability flags, exclusions and whole record notes added or reviewed after extraction	100	DS Functional Coding F1 to F5 (raw) (31); VAL_external (new flag) (28); DS Functional Coding (12); Admitted_via_goat_rule (new flag) (7); SensB_a / SensB_b / SensB_c (new, Definition B) (7); Whole record (all sheets) (4); 8 other fields (11 entries)
**Total**		**279**	

Classes were assigned from the Reason column of the cleaning log by the rule in canonical_rules.py; the classified log is in the canonicalization workbook (available on request). Field names are the column labels of the workbook.

Table S33. The 22 judgement overrides recorded in the cleaning log: entries in which the cleaning reviewer replaced an extracted value by a different canonical value on the basis of the study rather than of a written rule.

Study	Field	Extracted value	Canonical value	Reason recorded	In final corpus
Du_2025_001	Primary Species	Cattle	Mixed	Cattle disambiguation to Mixed	Yes
Du_2025_001	Geographic Scope	Global	International	Geographic Scope override	Yes

Lo_2022_002	Primary Species	Cattle	Mixed	Cattle disambiguation to Mixed	Yes
Zhang_2022_002	Reproducibility	Methods clear (data available from corresponding author upon request, code not publicly shared)	Methods clear	Reproducibility judgment	No
Fang_2025_001	Reproducibility	Methods clear (GEE-based workflow described in detail; data products available via supplementary materials but code not explicitly shared in main text)	Code-data available	Reproducibility judgment	Yes
Yang_2024_001	Reproducibility	Methods clear (data not shared per author statement)	Methods clear	Reproducibility judgment	Yes
Lei_2020_001	Geographic Scope	Local	Single farm	Geographic Scope override	Yes
LopezAndres_2018_001	Production System	Standard indoor (controlled environment)	Intensive	Production System override	No
LopezAndres_2018_001	ML Category	Optimization	Hybrid	ML Category override	No
Jaman_2023_001	Production System	NR	Not specified	Production System override	Yes
Jaman_2023_001	ML Category	Neural Networks	Traditional ML	ML Category override	Yes
Alemu_2025_001	Geographic Scope	Single country (multi-flock)	Multi-farm	Geographic Scope override	Yes
Alemu_2025_001	ML Category	Neural Networks	Traditional ML	ML Category override	Yes
Zhou_2025_001	Geographic Scope	Single farm (4 pens)	Single farm	Geographic Scope override	Yes
Zhou_2025_001	Reproducibility	Dataset from Korea AI Hub (not publicly available); code not shared	Methods clear	Reproducibility (data not public) to Methods clear	Yes
Liu_2016_001	Geographic Scope	Single lab	Not specified	Geographic Scope override	Yes
Liu_2016_001	Production System	NR (rumen-fistulated research animals for inoculum only)	Not specified	Production System override	Yes
McParland_2024_001	ML Category	Neural Networks	Traditional ML	ML Category override	Yes
Nakashima_2025	Production System	Rotational grazing (day-night system)	Pasture-based	Production System override	Yes

Duan_2022	Production System	Nomadic pastoralism (alpine rangelands)	Extensive	Production System override	Yes
Navon_2013_001	Primary Species	Cattle	Mixed	Cattle disambiguation to Mixed	No
Navon_2013_001	Geographic Scope	Multi-site	International	Geographic Scope override	No

Entries for LopezAndres_2018_001, Navon_2013_001, Zhang_2022_002 concern studies excluded under amendments 6 and 9 and are retained as audit trail. Four of the 22 entries (Zhang_2022_002, Fang_2025_001, Yang_2024_001, Zhou_2025_001) resolve a reproducibility rating whose extracted text was qualified; the others reassign a study to a different category of the same field.

Table S34. The eight leading countries under three counting conventions (n = 165 studies).

Rank	First listed (as published)	Fractional	Any mention
1	China (39)	China (39.8)	China (41)
2	United States (17)	United States (16.0)	United States (17)
3	Brazil (12)	Brazil (12.0)	Brazil (12)
4	Türkiye (8)	Türkiye (8.8)	Türkiye (10)
5	South Korea (6)	South Korea (6.0)	Canada (6)
6	Germany (5)	Germany (5.0)	South Korea (6)
7	Australia (4)	Canada (4.3)	Germany (5)
8	Canada (4)	Australia (4.0)	United Kingdom (5)
**Countries named; regional labels**	**43; 7**	**46; 7**	**46; 7**

First listed: each study counts once under the first country named. Fractional: each named country receives 1/k of a study naming k countries. Any mention: each named country counts once per study. Regional or multi-country labels (one study each: Global, International, East Africa, Eastern Africa, Multiple European countries, European Union and United Kingdom, Mongolia and Inner Mongolia, China) are counted as labels and not split. The 10 studies naming more than one country: Ali_2021_001 (Mauritania, Senegal, Türkiye); Ellis_2020_001 (Ireland, Spain, Canada); Liu_2016_001 (Mexico, Spain, China); Monteiro_2024_001 (United States, Canada); Peng_2023_001 (Netherlands, United Kingdom, Italy, Denmark); Senol_2024_001 (Egypt, Türkiye); Shadpour_2022_001 (Canada, Denmark); Wallace_2019_001 (United Kingdom, Italy, Sweden, Finland); Xie_2022_001 (United States, China); Xing_2025_001 (France, United Kingdom).

S14. Independent second coding of the functional flags and of the decision support claim

A second coder independently coded all 165 included studies on the six functional flags F1 to F6 from the full texts, under the final rubric (Table S16) and blind to the first coding, in a workbook that contained no earlier code (available from the authors on request). The second coder worked under the written rubric and the coding was checked by P.V. Final status was derived from the flags by the rule of Section 2.7 of the main text: Yes if F1 = Yes and F2 or F3 = Yes, Unclear if the information was insufficient, otherwise No. Agreement is reported as percent agreement and Cohen's kappa over the three categories Yes, Unclear and No, and over Yes against not Yes (Table S35). Disagreements were not reconciled: the first coding, which was checked against every full text (Section S2, amendment 5), is the analysis coding; every study on which the codings differ is listed in Table S36, and the counts under the second coding are given alongside. Under the second coding 27 studies deliver decision support and 5 are unclear, against 26 and 4 under the first; the gap reported in the main text does not depend on the coder.

Table S35. Agreement between the first and the blind second coding of the functional flags and the final status (n = 165 studies).

Flag	First coding Yes	Second coding Yes	Agreement, three categories (%)	Kappa, three categories	Agreement, Yes against not Yes (%)	Kappa, Yes against not Yes
F1 Names a decision	33	38	95.2	0.86	95.8	0.87
F2 Compares alternatives	27	25	98.8	0.96	98.8	0.95
F3 Recommends or controls	24	25	97.6	0.92	99.4	0.98
F4 Accessible tool	6	8	98.8	0.85	98.8	0.85
F5 Evaluated in use	4	2	98.8	0.66	98.8	0.66
F6 Outcome measured	2	2	100.0	1.00	100.0	1.00
**Final status**	**26**	**27**	**97.6**	**0.92**	**99.4**	**0.98**

Three categories: Yes, Unclear and No (F4 to F6 take only Yes or No). Final status cross-tabulation, first coding (rows) by second coding (columns), in the order Yes, Unclear, No: Yes 26, 0, 0; Unclear 0, 3, 1; No 1, 2, 132. Kappa for F5 is lowest because only 4 studies meet it, so a single difference weighs heavily.

Table S36. The 13 studies on which the first and the second coding of the functional flags differ, with the final status under each coding.

Study	Flags that differ (first coding to second coding)	Status, first coding	Status, second coding
Bhattad (2025; Bhattad_2025_001)	F4: No to Yes	No	No
Correa-Luna (2024; CorreaLuna_2024_001)	F5: Yes to No	No	No
Duan (2022; Duan_2022)	F1: No to Yes; F3: No to Unclear	No	Unclear
Fernandez (2023; Fernandez_2023_001)	F1: Yes to No	No	No
Handan-Nader (2019; HandanNader_2019_001)	F1: No to Yes; F3: No to Yes	No	Yes
Li (2024; Li_2024_001)	F1: No to Yes; F3: No to Unclear	No	Unclear
Monteiro (2024; Monteiro_2024_001)	F1: No to Yes	No	No
Nakashima (2025; Nakashima_2025)	F1: No to Yes	No	No
Ogungbuyi (2023; Ogungbuyi_2023_001)	F5: Yes to No	No	No
Qi (2026; Qi_2026_001)	F1: Unclear to No; F2: Yes to No	Unclear	No
Silveira (2023; Silveira_2023_005)	F1: Unclear to Yes	Unclear	Unclear
Simion (2025; Simion_2025_001)	F4: No to Yes	No	No
Xing (2025; Xing_2025_001)	F2: Yes to Unclear; F3: Unclear to No	Unclear	Unclear

The final status differs for 4 studies: Duan (2022) No in the first coding and Unclear in the second; Handan-Nader (2019) No in the first coding and Yes in the second; Li (2024) No in the first coding and Unclear in the second; Qi (2026) Unclear in the first coding and No in the second. The first coding is used in every analysis of the main text.

Decision support claim (Tables S39 and S40). The decision support claim field (column AF, Table S11) was also coded a second time for all 165 studies, by a second coder working from the full text of each study as published (title, abstract, introduction, discussion and concluding section) under a written rule that restates the extraction guide question: does the paper explicitly

aim to support farmer or manager decisions? The rule and both codes, with the passage on which each second code rests, are held in a workbook available from the authors on request; the differing studies and the passages are listed in Table S40. Yes was given when the paper states, anywhere in its text, that the study, model, data or output is meant to support, inform or guide decisions, management, planning, operation, mitigation strategies or policy and names the user or the kind of decision; hedged wording counts when the use and the user are named, and a study whose stated aim is the optimum operating or management setting of a process counts as Yes, because its output is a recommended setting for an operator. Partial was given when decision relevance is indirect or weak: a general statement that the information matters for management or policy, a use placed on future work or on a tool still to be built, or a single passing remark in a paper otherwise framed as prediction, measurement or understanding. No was given when no statement links the output to any decision, management, planning, operating or policy use. The second coder was blind to the first coding for 147 of the 165 studies. For 18 studies (marked with a dagger in Table S40 where they appear among the 64) the first code had been seen before the second was made: a first attempt at this coding, made from the title, abstract and concluding section only, had been checked against the first coding on a random sample of 14 differing studies and found to miss statements of intended use made in introductions and discussions, and the delivering studies examined in that check had also been compared; that first attempt was discarded, and the coding reported here was made afresh from the full text under the rule above, which was written after the check. Disagreements were not reconciled: the first coding remains the analysis coding, every study on which the codings differ is listed in Table S40 with the passage on which the second code rests, and the rates under the second coding are given alongside.

Results (Table S39). The two codings agree on 101 of 165 studies (61.2%; Cohen's kappa 0.24 over the three categories; 66.7% and kappa 0.28 for explicit claim against the rest; 90.3% and kappa 0.38 for any claim against none). The second coding is the more inclusive at the explicit level: 119 explicit claimants (72.1%; 95% CI 64.8 to 78.4) and 147 studies making some claim (89.1%; 95% CI 83.4 to 93.0), against 96 (58.2%) and 155 (93.9%) under the first coding. Of the 64 differences, 37 move a study from Partial to Yes, 11 from Yes to Partial, 7 from Partial to No, 5 from Yes to No, 2 from No to Yes and 2 from No to Partial; 48 of the 64 lie on the line between an explicit and a partial claim, and the codings agree on 149 studies as to whether any claim is made. Against the 26 delivering studies, the explicit claim rate under the second coding exceeds delivery by 56.4 percentage points (95% CI 48.8 to 63.9; 93 studies claim without delivering and none delivers without an explicit claim) and the inclusive rate by 73.3 points (95% CI 66.6 to 80.1), against 42.4 and 78.2 points under the first coding; the explicit rate exceeds delivery in 10 of the 11 application domains under both codings. All 26 delivering studies are explicit claimants under the second coding (19 explicit and 7 partial under the first). Five of the 7 (Okwu (2023; Okwu_2023_001); Olatunji (2023; Olatunji_2023_001); Reza (2022; Reza_2022_001); Şenol (2024; Senol_2024_001); Tuan Yusof (2014; TuanYusof_2014_001)) are bench-scale optimizations of anaerobic digestion or manure treatment whose stated aim is the optimum operating setting of the process, which the second rule counts as a claim addressed to an operator; the other two (Jeong (2022; Jeong_2022_001); Vaysi (2024; Vaysi_2024_001)) state a policy use of their estimates that the first coding read as partial. The difference between the codings therefore lies mainly in how far a hedged or implicit statement of intended use counts as an explicit claim, not in whether a claim is made; the main text reports the agreement next to the claim rate (Sections 3.7.1 and 4.5).

Table S39. Agreement between the first coding of the decision support claim (full text, at extraction) and the second coding (full text, independent), n = 165 studies: cross-tabulation, first coding in rows, second coding in columns.

First coding	Second coding: Yes	Second coding: Partial	Second coding: No	Total
Yes	80	11	5	96
Partial	37	15	7	59
No	2	2	6	10
**Total**	**119**	**28**	**18**	**165**

Agreement 101 of 165 (61.2%), kappa 0.24 over the three categories; explicit claim against the rest 66.7%, kappa 0.28; any claim against none 90.3%, kappa 0.38. Explicit claim rate 58.2% under the first coding and 72.1% under the second; inclusive rate 93.9% and 89.1%.

Table S40. The 64 studies on which the first and the second coding of the decision support claim differ, with the passage of the full text on which the second code rests.

Study	First coding	Second coding	Basis of the second code
Ali (2021; Ali_2021_001)	Partial	Yes	ANN 'can be successfully implemented as control system for optimizing and maximizing the operational conditions of the real-scale plants'; predictive models for 'better monitoring of biogas installations'
Ashworth (2024; Ashworth_2024_001)†	Partial	Yes	model constructed 'to aid ... prediction for real-time pasture resource management and planning'; 'if a forage and range producer had access ... equations ... could be used'
Bao (2025; Bao_2025_001)	Partial	Yes	abstract: understanding XCH4 variability 'can directly inform livestock management strategies, zoning policies, and national greenhouse gas reduction initiatives'; mitigation should focus on cattle density hotspots
Barczyk (2024; Barczyk_2024_001)	Partial	No	RF gap-filling and partitioning of N2O fluxes; conclusions about measurement method and drivers; no decision use stated
Basak (2022; Basak_2022_001)	Partial	No	CH4 modelling from feed intake and pig mass; recommends regression algorithms and further research only
Basak (2024; Basak_2024_001)	Partial	Yes	'an approach to support farmworkers in estimating ... NH3'; models 'can significantly aid by providing ... real-time guidance'; 'informed decision-making in swine production ... adjusting feed intake and ventilation systems based on the predictive models'
Bi (2024; Bi_2024_001)	Partial	Yes	'we seek to uncover insights that can inform breeding programs, management practices, and policy decisions'; 'robust foundation for informed decision-making'

Bi (2024; Bi_2024_002)†	Partial	Yes	'powerful tool for planning and reducing emissions'; 'we recommend that policymakers utilize the established benchmarks'; 'valuable tool for proactive planning' for stakeholders
Cao (2016; Cao_2016_001)	Partial	No	LS-SVM prediction of biochar yield; 'good reference for the modeling study'; no decision use stated
Cardinale (2022; Cardinale_2022_001)	Partial	Yes	'the Synomics Insights platform could help unlock the host's genetic as well as microbiome potential in precision management and genetic selection programs'; 'genetic selection for low-methane-emitting cows is promising'
Chernysh (2025; Chernysh_2025_001)	No	Yes	'findings aim to inform international researchers and practitioners about scalable, data-driven strategies to optimize anaerobic digestion'; ANN 'tool for optimizing process parameters'
Choo (2025; Choo_2025_001)	Partial	Yes	'predictive tools are urgently needed to support mitigation at the farm level'; 'framework empowers cooperatives and extension agencies to identify high-emitting farms and recommend tailored interventions'; policy planning
Dai (2023; Dai_2023_003)	Yes	No	HANPP mapping method; 'valuable basis for grassland sustainability assessment and disaggregating livestock data'; no decision use stated
Embaby (2025; Embaby_2025_001)	Partial	Yes	'practical tool for evaluating feeding strategies'; 'enables farmers to make informed decisions to adjust their feeding strategies'
Foschi (2025; Foschi_2025_001)	Yes	Partial	user-friendly tool to streamline carbon footprint calculation for LCA practitioners; assessment rather than a stated decision use
Hansen, B.G. (2020; Hansen_2020_001)	Yes	Partial	'findings have implications for dairy farmers and policy makers'; 'relevant for policy makers as it may contribute to improve the acceptance' (implications, not a stated decision use)
Hansen (2025; Hansen_2025_001)	Yes	Partial	models 'may help achieve sector reduction goals and support assessments at the individual farm level' (environmental reporting); 'could only be applied if a lower level of accuracy can be justified'
Hempel (2020; Hempel_2020b_001)	Yes	No	sampling and modelling study; control strategy mentioned only as a hypothetical aside about error measures
Hosseinzadeh-Bandbafha (2015; HosseinzadehBandbafha_2015_001)	Partial	No	descriptive energy and GHG modelling with ANN (read from page images); no decision use stated
Hou (2023; Hou_2023_001)	Yes	Partial	'may have important implications for grassland ecosystem management'; 'helpful for better planning and management' (general implications of an ecological study)
Jakimow (2018;	Yes	Partial	spatial knowledge of pasture management 'needed to better estimate potentials of more

Jakimow_2018_006)			sustainable management'; mapping method, no stated decision use
Jaman (2023; Jaman_2023_001)	Partial	Yes	objective 'to identify the optimum ratio' of co-digestion; ANN 'a more robust system to control the performance and to function as a precursor in commercial applications'
Jeong (2022; Jeong_2022_001)	Partial	Yes	'quantifying dairy CH4 emissions is critical for implementing California's climate change laws'; application to anaerobic digester deployment: identifies large farms and reduction potential; 'improve the return on investment ... of the state's investments in AD'
Kiat (2019; Kiat_2019_001)	Partial	Yes	abstract: 'benchmark information which can be used by the Sarawak government to develop appropriate policies and mitigation strategies'
Kolluru (2023; Kolluru_2023_001)	Partial	Yes	'This database will benefit stakeholders, ..., land managers, and policymakers'; 'assist policymakers and land managers in developing targeted land use, pasture management, and carrying capacity strategies'; hotspots 'can help herders and land managers devise proactive measures'
Küçüktopcu, E. (2021; Kucuktopcu_2021_001)	Yes	No	NH3 estimation model 'valuable in research on environmental protection ... litter management'; recommended as an alternative estimation approach
Liu (2016; Liu_2016_001)	Partial	No	chemometric model of methane and fatty acid networks; no decision use stated
Liu (2025; Liu_2025_001)	Partial	Yes	aim 'providing a scientific basis for optimizing dairy barn environmental management'; 'intelligent prediction tool for optimizing dairy barn environmental management'; 'effective tool for environmental management of dairy barns'
Lush (2018; Lush_2018_001)	Partial	Yes	measuring urination 'necessary ... to inform best management practices for reducing greenhouse gas emissions'; 'clear potential to inform agricultural management practices and policies'
Malek (2024; Malek_2024_001)	Partial	No	grazing cattle map to allow 'agricultural, land use and environmental assessments'; conclusions on data and methods; no decision use stated
Marin (2022; Marin_2022_001)	Partial	Yes	'this study aims to provide useful information for territorial planning'; 'can be promoted as a management tool to identify rangelands and design strategies'
Monteiro (2024; Monteiro_2024_001)	Yes	Partial	microbiome contribution to feed efficiency; possible uses ('better grouping strategies could be elaborated', dietary modification 'might become a possibility') in discussion only
Ocholla (2025; Ocholla_2025_001)	Partial	Yes	'can provide valuable insights for decision-support systems that prioritize identifying ... areas of high livestock density, encroachment, or overgrazing risk'; 'inform early warning systems

			or prioritize areas for field-based surveys'; 'census support'
Okwu (2023; Okwu_2023_001)†	Partial	Yes	models adopted 'to model and optimise biogas production' on substrate ratio and retention time (optimum operating settings); scale-up remark
Olatunji (2023; Olatunji_2023_001)†	Partial	Yes	'These models can be used to optimize the anaerobic digestion process ... at the industrial scale'; 'predicting the optimal process conditions at the strategic planning before loading the digester plants'
Park (2023; Park_2023_001)	Partial	Yes	abstract: models 'can facilitate ... the development of mitigation strategies for NH3-derived air pollutants, odor management, and the monitoring of animal-rearing environments'; 'equipping livestock farmers with crucial insights to guide the necessary interventions'
Park (2024; Park_2024_001)	Partial	Yes	'This approach intends to support farm management in optimizing feeding strategies and mitigating methane emissions'; 'practical tool for ... real-world applications in ruminant nutrition'
Pence (2024; Pence_2024_001)	Partial	Yes	'models can play an important role in improving the accuracy of GHG inventories and supporting data-driven policy decisions'; 'valuable information for policymakers and stakeholders interested in developing targeted strategies'
Pence (2024; Pence_2024_002)	Partial	Yes	'can give researchers and policy planners ideas'; 'studies such as the current study will support Turkey's 2050 climate change strategy and 2030 action plan preparations ... the Climate Change Directorate'
Reza (2022; Reza_2022_001)†	Partial	Yes	aim to optimise the operating parameters (pH, irradiation time, power) of the treatment process; optimum conditions given; 'will further encourage the application ... in pilot and full-scale processes'
Rodriguez (2022; Rodriguez_2022_001)	No	Yes	'emissions can also be regulated by operating the climate control system'; 'predictive systems can be incorporated into climate control systems to automatically optimize concentrations ... and emissions'; conclusion: models 'could be integrated into climate control systems, thus allowing for emission regulation'
Saha (2025; Saha_2025_001)	Partial	Yes	'Accurately mapping AFOs is essential for effective environmental and disease management'; 'facilitates early intervention and enhances monitoring'; 'findings can drive policy changes and direct resources'; 'essential tool'
Sahraei (2025; Sahraei_2025_001)	Partial	Yes	'These models can help farmers make informed decisions to optimize feeding strategies'; 'Practical implications for dairy farm management'; 'allows farmers to make data-driven decisions regarding feeding strategies, cow selection, and breeding programs'

Şenol (2024; Senol_2024_001)†	Partial	Yes	aim 'to optimise and model biogas yields' over biochar dose and digestion time (optimum operating settings); 'recommended that the optimum ... doses ... be used in pilot scale applications'
Şenol (2025; Senol_2025_001)	Partial	Yes	'vital to policymakers in terms of deciding on investment in biogas facilities'; 'Investors may need to prepare a feasibility study ... in Manisa'; 'assist policymakers' efforts'
Shadpour (2022; Shadpour_2022_001)†	Yes	No	prediction of weekly CH4 from milk MIR spectra; no decision, management or breeding use stated; conclusion on validation 'before applying the calibrated models for large-scale prediction'
Shea (2022; Shea_2022_001)	Yes	Partial	understanding manure applications 'could improve our ability to track the risks ... and guide strategies to avoid or mitigate harmful impacts'; conclusion: 'This future work can improve ... development of appropriate policies and practices' (future, general)
Shetty (2023; Shetty_2023_001)†	Partial	No	pilot study bridging a knowledge gap on two effluent systems and predicting E. coli levels; 'we hope that future studies will build upon this pilot study to benefit the multiple stakeholders' (no decision use)
Soto (2024; Soto_2024_001)	Yes	Partial	data product; maps 'necessary for many management, policy, and research purposes' (motivation); 'Further use of these products should demonstrate their usefulness as monitoring, prioritization, and inventory tools for planning and decision-making' (future)
Tuan Yusof (2014; TuanYusof_2014_001)†	Partial	Yes	aim 'to optimize the effects of the different ratios ..., initial pH values ... and different temperatures'; optimal operating conditions given (ratio 80:20, 35 C, pH 7.11) (optimisation of operating settings)
Vaysi (2024; Vaysi_2024_001)	Partial	Yes	'need for long-term pollution reduction policies informed by accurate livestock growth forecasts'; 'inform effective policymaking'; 'This paper proposes several strategies to support the development of Iran's biogas sector'
Wallace (2019; Wallace_2019_001)	Partial	Yes	'propose microbiome-led breeding/genetic programs to provide a sustainable solution'; 'it should be possible to optimize their abundance through selective breeding programs'; heritable microbes 'present primary targets for rumen manipulation'
Wang (2024; Wang_2024_001)	Partial	Yes	'valuable tool for ... guiding the optimization of dietary structures for dairy cows, rapidly screening methane-reducing feed options, and enhancing feeding efficiency'
Wang (2025; Wang_2025_001)	No	Partial	Treponema species 'promising microbial agents for targeted methane mitigation' (candidate probiotics, future intervention); mitigation

			strategies as motivation
Wawrzyniak (2023; Wawrzyniak_2023_001)	Partial	Yes	'Neural modelling, combined with expert knowledge, can be a unique tool for decision support in dairy cow herds'; 'provides valuable insights for decision making in dairy farming'; 'valuable for farmers to plan and optimise manure management strategies'
Wawrzyniak (2023; Wawrzyniak_2023_002)	Yes	No	'ANNs can be an effective tool in supporting the process of forecasting biogas production'; biomethane potential figures; no decision, planning or policy use stated
Werner (2024; Werner_2024_006)†	Yes	Partial	'Mapping these systems is crucial for monitoring land use changes in Brazil, playing a significant role in promoting sustainable agricultural production'; national incentive policy as context; 'mapping techniques to support sustainable ... production systems' (general)
Wu (2025; Wu_2025_004)	Yes	Partial	gridded livestock mapping method; 'fundamental data set upon which sustainable land development ... can be promoted'; 'the data can help with sustainable development of livestock farming' (general)
Yu (2025; Yu_2025_001)	Partial	Yes	'producers can pre-emptively design rations that not only meet the nutritional requirements of animals but also reduce CH4 emissions'; 'provides an artificial predictive tool for cattle farming, enabling pre-emptive diet design'; 'new approach for precise feeding of ruminants'
Zhang (2022; Zhang_2022_001)	Partial	Yes	'This study suggested that targeted planning and management should be carried out'; 'The grazing policy on the TP should be adjusted in a timely manner'; 'we suggested optimizing the management of pasture production and livestock production in areas where the grassland carrying capacity decreases greatly, such as in IC1, IIC2, and ID1'
Zhang (2023; Zhang_2023_001)	No	Partial	prediction for emission inventories; passing remark 'Improved prediction of CH4 emissions can assist researchers and farmers in improving the overall feed efficiency'; biomarkers 'for future research and mitigation strategies'
Zhang (2024; Zhang_2024_001)	Partial	Yes	'provides valuable insights and recommendations for implementing effective carbon reduction policies in China's swine industry'; 'Focus of future policy' section: 'The government should focus on implementing a series of policies ...'; 'theoretical support for the Chinese government to continue and optimize existing policies'
Zhang (2025; Zhang_2025_004)	Yes	Partial	field experiment on grazing and selenium addition; closing recommendation 'we suggest that moderate grazing and human management measures (such as fertilization) should be carried out' in a paper framed as understanding soil

			mineralisation
Zhou (2024; Zhou_2024_001)	Partial	Yes	'to inform stakeholders about these issues, we provided the first annual GDGI'; 'Stocking-rate determination can be optimized by using our grazing-intensity maps'; 'our GDGI maps can contribute to this decision-making processes' (policymakers and stakeholders)

Quoted phrases are from the full text as published; a bracketed remark gives the feature of the text that decided the code. A dagger marks a study among the 18 for which the second coder had seen the first code (see the text above). The first coding is used in every analysis of the main text.

S15. Citation tracking of the included studies

The coverage of the search was tested by a route independent of its terms: backward and forward citation tracking of the 165 included studies. The rule was written on 26 October 2026 before any record was retrieved and is reproduced, together with every candidate record and every screening decision, in a workbook available from the authors on request. The included studies were resolved to OpenAlex records by DOI (165 of 165; one study without a DOI was resolved by title). Backward tracking collected every work in their reference lists (8567 reference links, 7284 unique works); forward tracking collected every work citing them (3033 unique works). After the seeds themselves were removed, 9973 unique candidates remained; 7519 were journal articles published from 2010 onward in English or without a recorded language, and 7404 of these were absent from the 2664 records exported from the four databases on 9 January 2026 (matched by DOI, or by normalised title where the DOI was missing). A random sample of 100 of the 7404 candidates was screened from title and abstract against the eligibility codes of Table 1 of the main text, in the fixed order E1 to E7 and under the same rule as Section S12 (potentially eligible when the codes that can be judged from the abstract are passed; borderline

when one code cannot be settled). The screening was made by a second coder under the written rule and checked by a co-author.

Results (Tables S37 and S38). Of the 100 sampled candidates, 7 were potentially eligible and 9 borderline; the others failed one code, most often E1. Applied to the 7404 candidates, this gives an estimated 518 (254 to 1018) potentially eligible studies missed by the four-database search and reachable through the reference lists and citing papers of the included studies, or 1185 (747 to 1808) counting borderline records. Three of the seven potentially eligible records (milk spectra models of enteric methane and a Sentinel-2 pasture quality model) use partial least squares or similar regressions and pass criterion E1 only through its clause on statistical models (Section S2, amendment 10); the other four use boosted trees, maximum entropy or a transformer model. The expanded re-run of Section S12 estimated 147 (85 to 242) missed studies in OpenAlex by widening the search terms; the two estimates come from different routes and overlapping sets of records and are not additive, but both point the same way: the corpus is a reproducible but not exhaustive sample of the eligible literature. As for Section S12, the corpus was not expanded; the limitation is stated in Sections 2.3 and 4.5 of the main text, and the records found are listed in Table S38 for a future update.

Table S37. Citation tracking of the included studies: from seeds to screened sample (OpenAlex, 26 October 2026).

Step	Count
Included studies (seeds)	165
Seeds resolved to OpenAlex records	165
Reference links from the seeds (backward)	8567
Unique referenced works	7284
Unique works citing a seed (forward)	3033
Unique candidates after removing the seeds	9973
Journal articles, 2010 to 2026, English or language not recorded	7519
Already retrieved by the original search (DOI or title match)	115
Candidates not retrieved by the original search	7404

of which reached backward only / forward only / both	5005 / 2152 / 247
Random sample screened (seed 2026)	100
Potentially eligible in the sample	7
Borderline in the sample	9
Estimated potentially eligible studies among the candidates (95% CI)	518 (254 to 1018)
Estimated potentially eligible or borderline (95% CI)	1185 (747 to 1808)

Wilson 95% intervals for the sampled proportion, applied to the candidate pool. Route: whether a candidate was reached through a reference list of an included study (backward), through a paper citing one (forward), or both.

Table S38. The 16 potentially eligible or borderline records in the screened sample that are absent from all four record sets of the original search (OpenAlex work ID).

Route	OpenAlex ID	Year	Title	Decision
backward	W2753950018	2017	Predicting methane emissions of lactating Danish Holstein cows using Fourier transform mid-infrared spectroscopy of milk	potentially eligible
backward	W2790372122	2018	Predicting enteric methane emission of dairy cows with milk Fourier-transform infrared spectra and gas chromatography–based milk fatty acid profiles	potentially eligible
backward	W3096476223	2020	Understanding spatial variability of forage production in California grasslands: delineating climate, topography and soil controls	potentially eligible
backward	W3169939698	2021	Investigating the potential of Sentinel-2 configuration to predict the quality of Mediterranean permanent grasslands in open woodlands	potentially eligible
backward	W4221076616	2022	Prediction of laying hen house odor concentrations using machine learning models based on small sample data	potentially eligible
backward	W4391821154	2024	Mapping livestock grazing in semi-natural areas in the European Union and United Kingdom	potentially eligible
forward	W7165639379	2026	DeepGrass: A transformer-based approach for enhanced grassland management through Sentinel-2 time series spectral signature and vegetation indices forecasting	potentially eligible
forward	W2914268330	2019	Diagnosis of degraded pastures using an improved NDVI-based remote sensing approach: An application to the Environmental Protection Area of Uberaba River Basin	borderline
backward	W3125022415	2021	Estimation of biogas yields produced from combination of waste by implementing response surface methodology (RSM) and adaptive neuro-fuzzy inference system (ANF	borderline
backward	W3193946833	2021	Spatial patterns and driving factors of aboveground and belowground biomass over the eastern Eurasian steppe	borderline
backward	W3183549867	2021	The trends and projections of greenhouse gas emission by the livestock sector in Malaysia	borderline
backward	W4221118892	2022	Mapping of biogas potential of animal and agricultural wastes in Turkey	borderline

both	W4412047137	2025	Advancing Bioenergy: In-situ and in-silico Approach To Enhance Anaerobic Digestion	borderline
forward	W4406416724	2025	Agroecological mapping of livestock feed resources for sustain productivity and climate resilience in Benin (West Africa)	borderline
forward	W4413453518	2025	Spatiotemporal variability in drivers of grassland degradation and recovery under economic transformation in Mongolia	borderline
both	W7160992663	2026	Dynamic modeling of in vitro ruminal fermentation of agricultural residues using mechanistic and neural network–based approaches under limited experimental info	borderline

Borderline means that one eligibility criterion could not be settled from the abstract. Reasons for every sampled record are given in the workbook.
